# Tempo and rates of diversification in the South American cichlid genus *Apistogramma* (Teleostei: Perciformes: Cichlidae)

**DOI:** 10.1371/journal.pone.0182618

**Published:** 2017-09-05

**Authors:** Christelle Tougard, Carmen R. García Dávila, Uwe Römer, Fabrice Duponchelle, Frédérique Cerqueira, Emmanuel Paradis, Bruno Guinand, Carlos Angulo Chávez, Vanessa Salas, Sophie Quérouil, Susana Sirvas, Jean-François Renno

**Affiliations:** 1 Institut des Sciences de l’Evolution de Montpellier (ISEM), UMR CNRS/UM/EPHE 5554, IRD 226, CIRAD 117, Montpellier, France; 2 Instituto de Investigaciones de la Amazonía Peruana, Laboratorio de Biología y Genética Molecular, Iquitos, Perú; 3 University of Trier, Institute of Biogeography, Department of Geo-Sciences, Trier, Germany; 4 UMR Biologie des Organismes et Ecosystèmes Aquatiques, MNHN, UPMC, CNRS-7208, IRD-207, UCBN, Paris, France; 5 Universidad Nacional Federico Villareal, Facultad de Oceanografía, Pesquería, Ciencias Alimentarias y Acuicultura, Lima, Perú; SOUTHWEST UNIVERSITY, CHINA

## Abstract

Evaluating biodiversity and understanding the processes involved in diversification are noticeable conservation issues in fishes subject to large, sometimes illegal, ornamental trade purposes. Here, the diversity and evolutionary history of the Neotropical dwarf cichlid genus *Apistogramma* from several South American countries are investigated. Mitochondrial and nuclear markers are used to infer phylogenetic relationships between 31 genetically identified species. The monophyly of *Apistogramma* is suggested, and *Apistogramma* species are distributed into four clades, corresponding to three morphological lineages. Divergence times estimated with the Yule process and an uncorrelated lognormal clock dated the *Apistogramma* origin to the beginning of the Eocene (≈ 50 Myr) suggesting that diversification might be related to marine incursions. Our molecular dating also suggests that the Quaternary glacial cycles coincide with the phases leading to *Apistogramma* speciation. These past events did not influence diversification rates in the speciose genus *Apistogramma*, since diversification appeared low and constant through time. Further characterization of processes involved in recent *Apistogramma* diversity will be necessary.

## Introduction

The Amazon drainage system is the aquatic continental ecosystem hosting the highest fish species richness, with 2,500 species already described and some 1,000 yet to be described [1–2]. Approximately two-thirds of the Neotropical freshwater ichthyofauna occur in the Amazon drainage system [1–2]. Human activities have impacted the Amazon biodiversity since at least the pre-Columbian times and this impact has dramatically increased since the 1950s [3]. Local populations depend mainly on freshwater fishes for protein supply, and the ornamental fish trade has also contributed to the decline of some freshwater fish species in the Brazilian and Peruvian Amazon [1, 4–5]. More than anything, large-scale destruction of natural habitats (extensive road–building to allow timber exploitation, mining, gas and petroleum activities, reservoir construction and agro-industrial development) causes collateral damages on rivers, floodplains and wetlands, and thus put a high pressure on aquatic biodiversity [1, 6–10]. There is thus an urgent need to assess the global biodiversity, and especially the Amazon freshwater biodiversity.

Fish species with narrow geographical distributions are particularly threatened. This includes many Neotropical Cichlidae (subfamily Cichlinae), but especially most species of the genus *Apistogramma* Regan, 1913 (>100 species) [11]. *Apistogramma* are small fishes (dwarf cichlids) belonging to the tribe Geophagini, and characterized by a high sexual dimorphism in morphology and colour [12–13]. They occupy nearly the entire Neotropical region east of the Andes [12]. Most *Apistogramma* species have restricted and disconnected geographical distributions in the Amazon, Orinoco and Paraguay drainage systems of lowland tropical rainforests and open savannahs [11–12]. However, a few species, such as *A*. *agassizii* (Steindachner, 1875), *A*. *bitaeniata* Pellegrin, 1936, *A*. *cacatuoides* Hoedeman, 1951, or *A*. *trifasciata* Eigenmann and Kennedy, 1903 are rather ubiquitous, widespread and can be sympatric [11–12]. Species of *Apistogramma* occur in all types of water (clear, black and white waters), ranging from fast-flowing to stagnant waters [11]. They usually inhabit leaf litter on shallow banks of waters ranging from few tens (small streams) to hundreds (rivers) of km [11, 13]. Although probably many *Apistogramma* species still remain undescribed, molecular and morphological data suggest that this genus might be monophyletic and considered as the sister clade of *Taeniacara* Myers, 1935 [11, 14–18]. A cluster analysis based on coloration (notably, of lips, anterior dorsal membrane or during brood-care), as well as external morphological (such as black markings, body and fin shape, pores, dentition) and behavioural (family structure) characters established that all of the 116 *Apistogramma* species investigated could belong to three main groups: the *steindachneri*, *agassizii* and *regani* lineages [11]. A fourth lineage, including only *A*. *diplotaenia* Kullander, 1987, was suggested in a phylogenetic analysis where, however, neither the nuclear and mitochondrial markers nor the species taken into account were conveniently listed [18]. Seasonal or geological water-level fluctuations could have played an important role in *Apistogramma* speciation events by isolating populations and favoring the establishment of reproductive barriers [11]. A recent phylogeographic study on *A*. *caetei* Kullander, 1980 from eastern Amazonia indicated that three genetically different allopatric lineages showed a strong prezygotic isolation through female mate choice [19]. According to Ready et al. [19], the *Apistogramma* species richness could be seriously underestimated if future works reveal that their results are aimed to be indicative of a general trend (see also [20–21]).

Better assessment of the species diversity patterns and of the speciation processes are important conservation issues, notably for fishes subject to overfishing for ornamental trade purposes, such as *Apistogramma*. In this study, the diversity and evolutionary history of the Neotropical dwarf cichlid genus *Apistogramma* are investigated. Most attention was paid to interspecific molecular phylogenies to identify (1) the phylogenetic relationships among the species of *Apistogramma* and (2) putative cryptic diversity in this genus. Their evolutionary history and diversification rates are inferred from partial sequences of the mitochondrial cytochrome *b* and cytochrome *c* oxydase I genes (respectively, cytb and COI) and a nuclear marker used in several studies focused on the phylogeny of Cichlidae [22–26], the Tmo-4C4 single-copy locus (Tmo4C4).

## Materials and methods

### Species sampling and DNA analyses

No animal was killed specifically for the present study. Fishes were stored at the Instituto de Investigaciones de la Amazonia Peruana (IIAP) in Iquitos, Peru. Pieces of muscles and fins were taken from fishes preserved in alcohol. A permit from the Dirreccion Regional de la Produccion del Gobierno Regional de Loreto in Peru was obtained to export tissue samples to France. Documents are available upon request.

Tissue samples of *Apistogramma* were taken from 309 specimens for up to 41 morphologically identified species (or morphospecies) [27], depending on the analyzed dataset (S1 Table). These morphospecies were selected according to the lineages they were found to belong to in a previous cluster analysis [11]. The specimens were deposited in the Laboratorio de Biología y Genética Molecular (IIAP, Iquitos, Peru), the Museo de Historia Natural de la Universidad Nacional Mayor de San Marcos (Lima, Peru), the California Academy of Science (San Francisco, USA), the Field Museum of Natural History (Chicago, USA), and the Staatliches Museum für Tierkunde (Dresden, Germany). Tissues originated from Peru, Brazil, Venezuela, Ecuador and Bolivia. A list of the specimens with catalog numbers is provided in the S2 Table.

Total DNA was extracted from fin clips and muscle pieces preserved in 96% ethanol following standard procedures [28]. The partial Tmo4C4, cytb and COI were classically PCR-amplified (see Table 1 for primer details). Direct sequencing was carried out in both directions to confirm polymorphic sites. Sequences were produced through the technical facilities of the Platform “Genotyping and Sequencing” shared by the “Institut des Sciences de l’Evolution de Montpellier” (ISEM) and the “Centre Méditerranéen de l’Environnement et de la Biodiversité” (CeMEB) (Montpellier, France). Sequences were aligned by hand using MEGA v5.2.2 [29].

**Table 1:** Primers used for PCR-amplification of the Tmo-4C4 nuclear locus and the cytochrome *b* and cytochrome *c* oxidase I genes.

Gene Primer Name	Primer Sequence	Tm (°C)	References
**Tmo-4C4**			
Tmo-f2	5'-ATCTGTGAGGCTGTGAACTA-3'	55	[30]
Tmo-4C4R	5'-CATCGTGCTCCTGGGTGACAAAGT-3'		[31]
**Cytochrome *b* gene**			
ApistoCB1	5’- ATGGCAAWTTTACGAAA-3’	46	this study
CytIntR	5'-GGTGAAGTTGTCTGGGTC-3'		[17]
**Cytochrome *c* oxidase I gene**			
Pros1Fwd	5'-TTCTCGACTAATCACAAAGACATYGG-3'	46	[24]
Pros1Rev	5'-TCAAARAAGGTTGTGTTAGGTTYC-3'		[24]

Phylogenetic analyses were performed on datasets including 287 original sequences of Tmo4C4 as well as 282 and 193 original sequences of cytb and COI, respectively. These datasets were completed with GenBank sequences of *Satanoperca* Günther, 1862, *Crenicara* Steindachner, 1875, *Biotodoma* Eigenmann and Kennedy, 1903, *Gymnogeophagus* Miranda-Ribeiro, 1918, *Taeniacara* Myers, 1935 and *Geophagus* Heckel, 1840, that were used as outgroup. Details about sampling sites for the original sequences, as well as the GenBank accession numbers are given in the S2 Table.

### Phylogenetic analyses and species delimitation

Phylogenetic analyses were conducted on both separate (sequences) and concatenated (sequences or haplotypes) gene datasets through the technical facilities of the Platform “Montpellier Bioinformatics Biodiversity” (MBB) shared by ISEM and CeMEB. In the concatenated dataset, chimera sequences were built from Tmo4C4, cytb and COI sequences of the outgroup species. For instance, no cytb sequence was available for *Crenicara punctulatum* (Günther, 1863) in GenBank, only for *Crenicara* sp. Sequences of Tmo4C4 and COI of *C*. *punctulatum* were thus concatenated with the cytb sequence of *C*. sp. (S2 Table). Phylogenetic trees were reconstructed with a maximum likelihood approach (ML) using PhyML v3.0 [32] and a Bayesian inference (BI) using MrBayes v3.1.2 [33]. Best-fitting models of sequence evolution were identified for each dataset with MrModeltest v2.3 [34]. Node robustness was estimated by bootstrap percentages (BP) in ML after 1000 replicates, whereas Bayesian posterior probabilities (PP) were obtained from the 50% majority rule tree consensus after a burn-in stage of 25,000. In BI, three independent runs of five Markov chain Monte Carlo (MCMC) samplings were also performed for five million generations with trees sampled every 100 generations. Alternative hypotheses of *Apistogramma* lineage relationships were compared with the Shimodaira-Hasegawa Test [35] as implemented in PAUP*4.010b [36].

**Fig 1:**
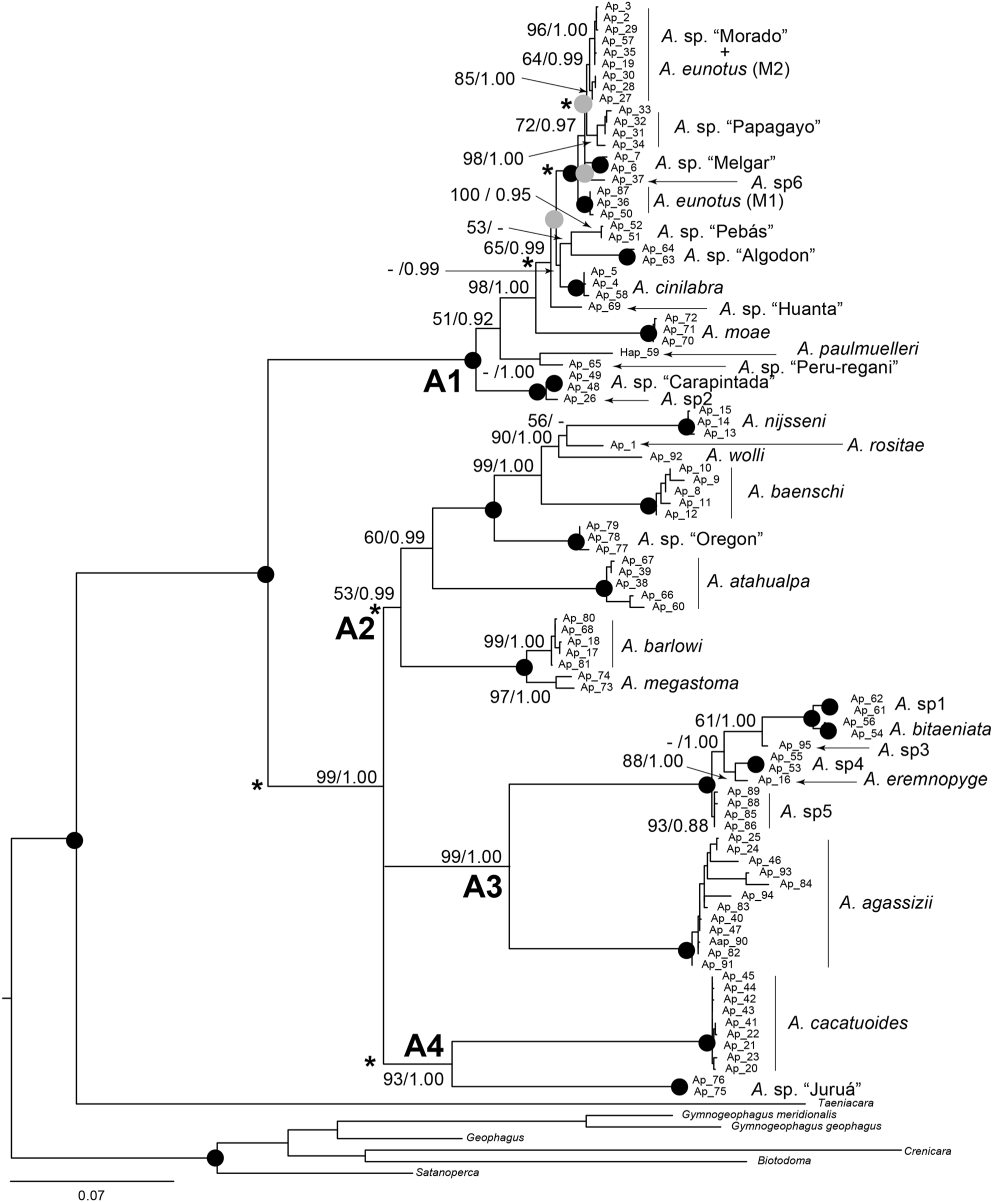
Maximum likelihood tree reconstructed from the *Apistogramma* concatenated haplotype dataset of the mitochondrial cytochrome *b* and cytochrome *c* oxydase I genes and the Tmo-4C4 nuclear locus. Haplotypes are detailed in the S2 Table. Numbers at nodes are for bootstrap percentages (≥ 50%) and posterior probabilities (≥ 0.85). Black circles indicate nodes with BP = 100% and PP = 1.00, while grey circles are for nodes with a weak support (BP < 50% and PP < 0.85). Nodes with “-”are weakly supported in maximum likelihood approach or Bayesian inference. Branches with “*” indicate short internal branches not significantly different from zero.

Species delimitation tests were performed using a multi-locus coalescent-based method implemented in BPP v3.3 [37–38]. This method takes into account incomplete lineage sorting due to ancestral polymorphism and gene vs species tree conflicts. It allows the joint estimation of Bayesian species delimitation and species tree. To validate the genetically identified species, two initial hypotheses were used: one based on a species tree reconstructed from consensus sequences obtained for each morphospecies, and the other based on the species tree (excluding the outgroup) obtained in the frame of divergence time estimates with StarBEAST2 [39] (see next section). To underline putative cryptic diversity, species delimitation tests were then performed on the species validated by BPP that included more than ten individuals. The topology of the guide tree for each species tested was extracted from the haplotype tree (Fig 1). In all cases, the analyses were based on the concatenated dataset. Several combinations of priors for ancestral population size (*θ*s: α = 1 or 2; β = 10, 20, 100, 200, 2000) and root age (τ_0_: α = 1 or 2; β = 10, 20, 100, 200, 2000) were tested. For each test, the other priors were the following: speciesdelimitation = 1, speciestree = 1, speciesmodelprior = 1, algorithm = 0, finetune ε = 2, usedata = 1, locusrate = 1, heredity = 2 (scalar values = 1 for nuclear marker and 0.25 for mitochondrial marker) and cleandata = 1. Finetune variables were automatically adjusted, and swapping rates for each parameter were checked for recommended values (0.10–0.80) [40]. Each analysis was run twice to confirm consistency between runs.

Intra- and intergroup genetic divergences were estimated by the K80 distance with MEGA. As in López-Fernández et al. [17], an internal branch test was performed with MEGA on the concatenated dataset to determine whether short internal branches in the phylogeny were resolved relationships or polytomies. The neighbour-joining method was used to build a tree under the K80 model, with and without a gamma distribution (G).

### Molecular dating estimates

Two scientific schools exist about the origin of the Neotropical cichlids: either related to the Gondwana tectonic fragmentation [41–45], or after trans-Atlantic dispersal from Africa [46–49]. Several attempts of molecular dating were undertaken on the sole base of the fossil record [47, 50–51]. However, the use of different calibration points, datasets, molecular markers and analytical approaches has provided different divergence time estimates for the Cichlinae and Geophagini. The oldest Neotropical cichlids known from the fossil record are from the Lumbrera Formation in Argentina [52–54]. Sedimentological, paleontological, and geochemical dating studies suggest a Middle Eocene age (47.8–41.2 Myr; Lutetian) for the uppermost section of the Lower Lumbrera Formation where a species assigned to the Geophagini, *Gymnogeophagus eocenicus* Malabarba et al., 2010, was discovered [53–55]. The occurrence of this fossil predates the hypothesis of a Neotropical cichlid origin after a trans-Atlantic dispersal around 29.2 Myr (34.8–25.5 Myr) [47].

The date of 44.5 ± 3.3 Myr for the occurrence of *G*. *eocenicus* in the Lower Lumbrera Formation [54] was thus used as fossil calibration point. However, from a phylogenetic standpoint, *G*. *eocenicus* has been shown to be possibly nested within the living genus *Gymnogeophagus* [53], and thus does not represent the most recent common ancestor of *Gymnogeophagus*. According to a morphometric analysis, *G*. *eocenicus* seems to be the sister species of *G*. *rhabdotus* (Hensel, 1870) and *G*. *balzanii* (Perugia, 1891) [53]. Sequences of these two latter species were available in GenBank only for cytb. In order to correctly place this calibration point for the concatenated dataset, a first analysis was thus run for a cytb sub-dataset corresponding to the specimens included in the concatenated dataset, as well as *G*. *rhabdotus* and *G*. *balzanii*. A second analysis was then run for the same cytb sub-dataset, but by removing *G*. *rhabdotus* and *G*. *balzanii*. Priors of the analyses are provided below. An estimation of the Geophagini root age by McMahan et al. [51], 51 Myr (64–40 Myr), was used as an additional calibration point. These authors used both mitochondrial and nuclear markers, as in the present study, and the oldest known fossil for several cichlid clades as calibration points rather than biogeographic hypotheses (i.e. ages related to the tectonic fragmentation of Gondwana).

To estimate the time to the most recent common ancestor (TMRCA) for the *Apistogramma* species, Bayesian coalescent analyses were conducted at MBB. Analyses were performed for two speciation models (Yule and birth-death) with three molecular clocks (strict, relaxed uncorrelated lognormal and relaxed uncorrelated exponential clocks) using StarBEAST2 [39]. With Tracer v.1.6 [56], speciation models and clocks were compared using the Akaike’s information criterion through MCMC (AICM) [57] to test which of them best fit our data. Normally distributed priors were used for node calibration points: the *Gymnogeophagus gymnogenys* (Hensel, 1870) / *G*. *meridionalis* Reis and Malabarba, 1988 node calibrated with the age of *G*. *eocenicus* (mean = 44.5, Sigma = 2); the estimation of the Geophagini root age (mean = 52, Sigma = 7.3). StarBEAST analyses were performed with five independent runs of 100 million generations with the first 10% removed as burn-in (see S1 File for details). Markov chain convergence was ascertained by visual inspection of the traces, while the stability of each run was measured using the effective sample size (ESS > 200 for all parameters) using Tracer. Results of the independent convergent runs were combined with LogCombiner v2.4.4 [58] to estimate TMRCA and 95% confidence intervals. A consensus tree was generated using TreeAnnotator v2.4.4 [58] with mean node heights as node heights option and maximum clade credibility as target tree type option.

### Diversification rates

Diversification rates were estimated using BayesRate v1.63b [59] for the entire tree and for the lineages of *Apistogramma* as defined in the introduction from morphological and/or molecular data [11, 18]. Marginal likelihoods via the thermodynamic integration were calculated to select the best-fitting model of diversification between the pure-birth or birth-death processes, under the following parameters: 100,000 MCMC iterations per three chains for 1,000 randomly sub-sampled posterior species trees obtained with StarBEAST and excluding the outgroup. Marginal likelihoods were then compared using the AICM, as previously mentioned. Speciation (λ), extinction (μ) and diversification (λ–μ) rates through time were finally estimated with the selected model and previously mentioned parameters. The results were visualized with Tracer.

A lineages through time (LTT) plot was used to summarize the accumulation of diversity across evolutionary time for a given phylogeny. It was thus constructed with the ltt.plot function of the ape package [60–61] for R v3.3.3 [62] from the species tree (without outgroup) obtained with StarBEAST and TreeAnnotator. Predicted LTT curves (λ and μ from the BayesRate analyses) were obtained with the LTT function of ape [63], and compared to the observed LTT plot.

## Results

### Phylogenetic relationships and species delimitation

All new sequences were deposited in the ENA database under the accession numbers LN678825-LN678947 and LT617356-LT617520 for Tmo4C4, LN678702-LN678824 and LT617119-LT617280 for cytb, and LN678948-LN679066 and LT617281-LT617355 for COI (S2 Table).

For the separated datasets, the full alignments represented: 293 positions for Tmo4C4 with 28 phylogenetically informative sites (PIS) within the 333 *Apistogramma* sequences; 669 positions and 326 PIS for 315 cytb sequences; 583 positions and 265 PIS for 207 COI sequences. The concatenated dataset, which includes Tmo4C4+cytb+COI sequences of 180 individuals corresponding to 56% of the sampled specimens for, respectively, 19, 60 and 61 haplotypes, was thus 1545 nucleotides long and it has 583 PIS within the *Apistogramma* sequences.

The best-fitting models of nucleotide substitution were the K80 model [64] with a proportion of invariable sites (I) and a gamma distribution (G) for Tmo4C4, whereas the GTR model [65] +I+G was selected for cytb, COI and the concatenated dataset in ML. On the other hand, a mixed-model analysis (K80+I+G and GTR+I+G) was performed in BI for the concatenated dataset. Based on the subset of *Apistogramma* species included in the present study, the monophyly of *Apistogramma* was highly supported in all tree topologies, except for the one obtained in BI for the Tmo4C4 (Fig 1 and S1–S4 Figs): BP between 87% (COI) and 100% (Tmo4C4+cytb+COI); PP = 1.00. In trees reconstructed from cytb and concatenated datasets, the individuals were clustered into four monophyletic groups with weak to high support values: A1, 99% < BP < 100% and PP = 1.00; A2, less than 50% < BP < 100% and 0.81 < PP < 0.99; A3, 85% < BP < 99% and PP = 1.00; A4, 57% < BP < 94% and 0.97 < PP < 1.00. The A2, A3 and A4 clades grouped together in a trichotomic clade (88% < BP < 99% and PP = 1.00). Alternative relationships (A2+A4/A3, A3+A4/A2 or A2+A3/A4) between these three latter groups were investigated with the Shimodaira-Hasegawa Test [35]. The best ML tree differed from the tree presented in Fig 1 by placing the A2 group as the sister group of A3+A4. However, the other relationships tested (A2+A4/A3 and A2+A3/A4) were not significantly worse than the best ML tree at the 5% confidence level (0.17 < *P* < 0.57). In COI trees, two clades were identified: A1, BP = 99% and PP = 1.00; A2+A3+A4, BP = 84% and PP = 1.00. In Tmo4C4 trees, the phylogenetic relationships inside the *Apistogramma* group remained unresolved. Some individuals belonging to some undescribed morphospecies could not be attributed to a genetically identified species. They were named *A*. spx in all present figures and tables.

Since the genetically identified species did not fully match the morphospecies, two initial hypotheses were used in the frame of the species delimitation tests. The “morphospecies” hypothesis was not validated by the multi-locus coalescent-based method with BPP from the concatenated dataset. The BPP analyses rather suggest 26 putative species (*θ*s: α = 2 and β = 200; τ_0_: α = 2 and β = 2000; PP = 0.97) corresponding to most morphospecies except three groups. The “StarBEAST species tree” hypothesis was, on the other hand, validated (*θ*s: α = 2 and β = 2000; τ_0_: α = 2 and β = 2000; PP = 1.00). Among the valid species, six included more than ten individuals: *A*. *agassizii*, *A*. *barlowi* Römer and Hahn, 2008, *A*. *cacatuoides*, *A*. *cinilabra* Römer et al., 2011, *A*. *eunotus* Kullander, 1981, and *A*. sp “Morado”. For four of them (*A*. *barlowi*, *A*. *cacatuoides*, *A*. *cinilabra*, *A*. *eunotus*), the BPP analyses suggested one putative species with high posterior probabilities (*θ*s: α = 2 and β = 20; τ_0_: α = 2 and β = 200; 0.84 < PP < 1.00), while the BPP analyses suggested seven (*A*. sp “Morado”) or nine (*A*. *agassizii*) putative species, but with low posterior probabilities (*θ*s: α = 2 and β = 2000; τ_0_: α = 2 and β = 2000; PP = 0.32 and 0.41, respectively).

Intragroup genetic divergence ranged from 0% to 1.4% for the 31 genetically identified species from the concatenated dataset (Fig 1 and S1 Fig), while intergroup genetic divergence ranged from 1.1% to 26.7% for these species (S3 Table). The A1 group is characterized by shorter internal branches than the A2, A3 and A4 groups. However, the internal branch test indicates that most branches are significantly different from zero with length confidence probabilities higher than 95% for most interspecific branches (Fig 1) [29, 66].

### Divergence time estimates

**Fig 2:**
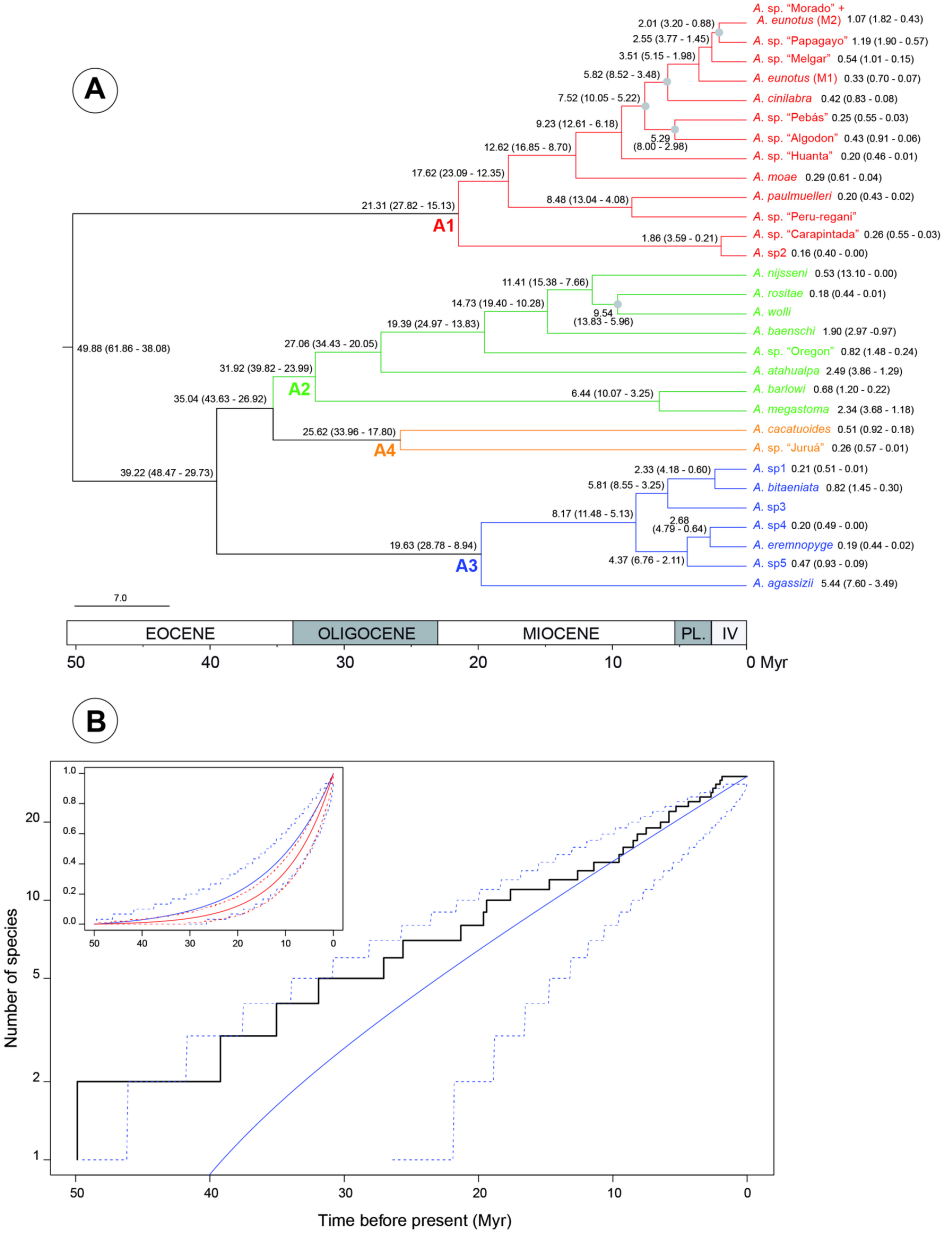
Chronogram showing the divergence time estimates (A) and lineages through time plot (B) of all the *Apistogramma* species taken into account in the present study. A: values at nodes and with species names reflect the time (in Myr) to the most recent common ancestor and, in brackets, the 95% confidence intervals. Values with species names are divergence times estimated from the mitochondrial dataset. Grey circles are for nodes with posterior probabilities < 0.85. PL. and IV are for the Pliocene and the Quaternary, respectively. B: the x-axis represents the time before present in Myr, while the y-axis is the number of species (*N*) on a logarithmic scale. The black line is for LTT plot constructed from the species tree (A), while the blue lines are for the predicted LTT curve obtained with λ = 0.072 (dashed lines are the 95% prediction interval). The insert shows two predicted LTT curves with λ = 0.072 and *N* = 30 (blue), and λ = 0.103 and *N* = 100 (red). The two curves were standardized to be compared.

Bayesian coalescent analyses were conducted under the GTR+I+G model from the concatenated cytb and COI dataset and the K80+I+G model for the Tmo4C4 dataset. The AICM suggested that the Yule model of speciation and the relaxed uncorrelated lognormal clock were significantly best suited to our dataset. *A*. sp6 was removed from the dataset because only mitochondrial markers were sequenced for one individual. Results of three of the five independent runs converged and were thus combined with LogCombiner for further analyses (Fig 2A).

The split between the A1 and the other groups of *Apistogramma* from their most recent common ancestor took place at the beginning of the Eocene (≈ 50 Myr), while the split between the A2, A3 and A4 groups seems to have occurred at the end of the Eocene (≈ 39 Myr). The four identified clades or lineages began to diversify from the beginning of the Oligocene (from ≈ 32 Myr for A2 to ≈ 20 Myr for A3 and A4). The extant species included in our dataset originated within the Pleistocene (from 2.49 to 0.16 Myr).

### Diversification rates

The AICM provided a strong support for the pure-birth process in diversification estimates. The net diversification rate detected with this model was 0.072 ± 0.017 (λ = 0.072 ± 0.017 and μ = 0) event/Myr. The estimate of the diversification rate was found slightly higher (0.103 ± 0.020) when the proportion of species included in the phylogeny was set to 33%. The diversification rates for each lineage are as follows: 0.184 ± 0.045 event/Myr for A1; 0.092 ± 0.027 event/Myr for A2; 0.337 ± 0.121 event/Myr for A3. No rate of diversification was estimated for the clade A4 because BayesRate does not allow this for a clade with less than three taxa.

In agreement with the above results, the observed LTT plot (Fig 2B) exhibited a pattern of constant diversification through time. The predicted LTT curve calculated with the estimated parameters showed a good fit with the observed plot, particularly for the recent times but a bit less deeper in time, though the logarithmic scale magnified the differences. Interestingly, the predicted LTT curves calculated with the two above estimates of λ (considering missing species or not) were very similar and their prediction intervals overlapped widely (insert of Fig 2B).

## Discussion

The aims of the present study were, first, to investigate the phylogeny of the genus *Apistogramma*, a speciose group of Neotropical cichlids suffering from ornamental trade, second to better understand the origin and tempo of diversification in this genus. An extended number of morphospecies (up to 41) were included in the present molecular analyses compared to previous published studies (between 1 and 4) [14–17, 22–25, 43, 49, 51–52]. The use of only two mitochondrial (cytb and COI) and one nuclear (Tmo4C4) markers should be seen here as a limitation of our study. Nevertheless, our phylogeny based on these markers but including several representatives of the major *Apistogramma* lineages and species sampled appears better resolved than previous ones.

### Phylogeny of *Apistogramma*

The monophyly of the genus *Apistogramma* was already established from morphological (external characters and osteology) and/or molecular (mitochondrial cytb, ND4, 16S and nuclear RAG2, Tmo-M27, Tmo-4C4 genes) datasets focused on the Geophagini [15–17]. Considering a larger and more evenly distributed number of species, the present study also suggests monophyly of the genus. Three of the four clades identified here were found to correspond to species groups described on the basis of colour, morphological and behavioural characters as well as unspecified molecular markers [11, 18]. The fourth clade could not be identified because it was not possible to include cytb, COI or Tmo4C4 sequences of its representatives (*A*. *diplotaenia*) in our datasets. Indeed, the A1 lineage includes species of the *regani* lineage, while the A2 and A3 lineages cluster species of the *steindachneri* and *agassizii* lineages, respectively. Species found in the A4 group, and especially *A*. *cacatuoides*, are included in the *steindachneri* lineage by Römer [11], while they are included in the *agassizii* lineage by Miller and Schliewen [18]. In the present study, the phylogenetic relationships between the A2, A3 and A4 lineages remain unresolved. Alternative hypotheses regarding the sister group relationship of A4 with A2 and A3 do not allow to favor one hypothesis rather than another. More data are required to confirm or refute the phylogenetic position of the A4 group.

Discrepancies among studies were also found within *Apistogramma* lineages. For instance, *A*. *barlowi* and *A*. *nijsseni* Kullander, 1979 are considered as species of the *cacatuoides* complex in Römer [11], whereas they are not closely related to this complex in the present study. Likewise, *A*. *nijsseni* and *A*. *atahualpa* Römer, 1997 are included in the *agassizii* lineage in the phylogeny of Miller and Schliewen [18], whereas both species are clustered with species of the *steindachneri* lineage in both Römer [11] and the present study. On the basis of morphological data, Britzke et al. [67] included *A*. sp. “Papagayo” and *A*. sp. “Pebás” in *A*. *ortegai* Britzke et al., 2014. However, in the present study (Fig 1 and S1, S3 and S4 Figs), *A*. sp. “Papagayo” and *A*. sp. “Pebás” are not sister species. This means that one of these clades, at least, is not a sub-population or a sub-species of *A*. *ortegai*, and it might be considered as a different species if samples of *A*. *ortegai* could have been included in the present dataset. Lastly, some individuals with the *eunotus* morphotype are clustered with *A*. sp. “Morado” individuals, while other individuals with the *eunotus* morphotype are combined in another monophyletic group. This pattern might be the result of one of the following scenarios: (1) the fixation of *A*. sp. “Morado” haplotypes in some *A*. *eunotus* populations by introgressive hybridization of sympatric populations from secondary contact [68–69]; (2) an incomplete lineage sorting during past speciation events [70–71]; (3) improper taxonomic identification of specimens studied. These processes are difficult to distinguish based on phylogenetic reconstructions. A further investigation is needed with more appropriate genetic markers (e.g., microsatellites or RAD-sequencing markers).

Our sequence-based phylogenetic study of 31 *Apistogramma* genetic species underlined widespread genetic variation within this genus. Because the intragroup upper bound (1.4%) and the intergroup lower bound (1.1%) of the range of the estimated genetic divergences are overlapping, genetic distances seem to be an uncertain criterion for delimiting closely related species [72]. Methods such as the Automatic Barcode Gap Discovery [73] could be used for this purpose but require a large number of individuals per taxon. Hereby, a multi-locus coalescent-based method was preferred to validate the genetically identified species and to evaluate putative cryptic diversity. This method suggests the occurrence of 30 putative species in the concatenated dataset. These species were found to correspond to the genetically identified species (excluding *A*. sp6) (Figs 1 and 2 and S1 Fig). Among the six species with a number of sampled individuals greater than ten, no cryptic diversity was underlined in *A*. *barlowi*, *A*. *cacatuoides*, *A*. *cinilabra* or *A*. *eunotus*. Putative cryptic diversity is however weakly supported in *A*. *agassizii* and *A*. sp. “Morado”. Such genetic diversity might reflect large phenotypic variation. For instance, *A*. *agassizii* is characterized by phenotypic plasticity for colour, patterns and body proportions, and several attempts to create new species have been proposed [12]. This kind of species with patchy isolated populations distributed in the Amazon basin could represent likely sources of investigation on cryptic diversity, and deserve more attention through phylogeographic studies or population genetics / genomics approaches.

### Evolutionary history of the genus *Apistogramma*

The fossil record and the molecular phylogenetic evidence suggest that most lineages of freshwater fishes currently dominating Neotropical ecosystems originated by the Late Cretaceous, and started their diversification before or during the Early Paleogene [42, 74–76]. For the origin of the Cichlinae, several time estimates were proposed: at 140–120 Myr if related to the breakup of Gondwana [41–45]; 124 Myr (146–104 Myr) [50] on the basis of the same biogeographic hypothesis and the fossil record; 82 Myr (89–74 Myr) [48] or 63 Myr (74–54 Myr) [51] only on the basis of fossil calibration points. Based on paleontological and relaxed molecular-clock estimates, Friedman et al. [47] consider this origin as much younger (34.8–25.5 Myr). This latter hypothesis implies that the origin of Neotropical cichlid fish should be posterior to the origin of the oldest Neotropical fossil cichlids (between 47.8 and 41.2 Myr for *Proterocara argentina*, *Gymnogeophagus eocenicus*, and *Plesioheros chauliodus* from the Lumbrera Formation) [54–55]. These diverse approaches generated various interpretations for the origin of the Geophagini (between 107 Myr and 52 Myr) [48, 50–51], the split between the genera *Taeniacara* and *Apistogramma* (between 70 Myr and 33 Myr) [48, 50] or the *Apistogramma* origin (between 52 Myr and 15 Myr) [48, 50]. The dates obtained here for the Geophagini and *Apistogramma* clades are included in their previously estimated range of divergence times.

In Amazonia, major marine regressions are known notably for the Paleocene (≈ 59–55 Myr) and the Early Eocene (≈ 43–42 Myr) [74, 77]. According to our molecular dating analyses, early stages of the *Apistogramma* diversification seem to have occurred between marine regressions. The ancestral *Apistogramma* populations thus isolated because of higher salinity levels might have initiated allopatric differentiation leading to the A2 and A4 lineages during the Oligocene. On the basis of the same hypothesis, the differentiation of the A1 and A3 lineages seem to have occurred at the beginning of the first major Neogene marine incursion (≈ 20–17 Myr) [77–79]. The present-day fluvial system started then to develop and was fully established at approximately 6.8 Myr [80]. During the latest Neogene (7–2.5 Myr), lowland aquatic habitats became better-delineated drainage systems rather than a system of more or less connected wetlands and lakes. During the Quaternary, these aquatic ecosystems were strongly affected by the glacial cycles. Especially cooler temperatures (a decrease of 8°C in the Andes and of 4–5°C in Amazonian lowland) [81–82] induced, for instance, increase/decrease of seasonal water levels, sediment supply, lake-level variations, stream capture or dispersal-based habitation [75, 83]. Moreover, glacial sea level lowering induced large lateral shifts of major rivers in Early Pleistocene and a cyclic process of vertical erosion, flooding and filling in the Amazon trunk during the Middle and Late Pleistocene [83–84]. These Quaternary glacial cycles could also be at the origin of recent events of differentiation leading to the speciation of several genetically identified *Apistogramma* species analyzed in the present study. Quaternary sea-level oscillations and deposition of Andean sediments could be at the origin of Amazonian *várzeas* (freshwater swamp or flooded forests), *ria* (river with typical lake features because of flow velocity reduction) [84–86], and probably oxbow lakes (meander cut-off from the main river stem) considered as favourable environments for allopatric speciation [87–90].

### Diversification in the genus *Apistogramma*

The observed LTT plot did not evidence for substantial shifts in the *Apistogramma* diversification, even if the rate of diversification for the A3 lineage was found slightly higher compared to the other lineages (0.337 vs 0.184 and 0.092 event/Myr). High estimates of the diversification rates could be strongly linked to the species richness of a clade [91–93]. However, the A3 lineage is not the most species-rich clade in our dataset. The rates of diversification presented here (0.072 or 0.103) for the genus *Apistogramma* is close to the rates observed in the Cichlidae (0.069) [51] and the Percomorpha (0.081) [94–96], but slightly higher than the rate of the Teleostei as a whole (0.041) [95]. According to McMahan et al. [51], only the Heroini were found to potentially have elevated diversification rates relative to the other Cichlinae.

Adaptive radiation is often invoked to explain the species diversification in the Cichlidae as in the East African cichlid species characterized by ecomorphological and colour variations (e.g. [97–100]) or in the Neotropical crater lake (e.g. [101–104]) and riverine [50, 105–106] cichlids. According to López-Fernández et al. [17], short branches at the base of the Geophagini clade suggest a possible early burst of evolutionary divergence considered as a pattern of adaptive radiation [107]. Compared to the Geophagini, the *Apistogramma* phylogenetic tree displays few basal short internal branches compared to the terminal branches. Moreover, the statistical support of the cluster generated by these internal branches was not significant, and the relationships remain thus poorly supported. This suggests a lack of phylogenetic resolution rather than fast lineage differentiation. This is largely supported by the overlapping prediction intervals of the LTT curves with different parameters and sampling scenarios. Short internal branches observed in the A1 lineage might indicate however a possible recent and rapid speciation event, or an ongoing speciation process, as also suggested by the overlap between the intra- and intergroup genetic divergences (S3 Table). Overall, the observed tree topology and LTT plot (constant diversity through time) do not seem compatible with an early burst of species diversification as postulated for the Geophagini [17, 50]. Paleogene and/or Neogene marine incursions, the establishment of the modern Amazon drainage system during the Miocene and the Quaternary glacial cycles might be as well at the origin of several vicariant events in *Apistogramma*. Results from the present work are however not sufficient to deeply investigate either of these hypotheses. New phylogenetic analyses on an extended dataset, the genotyping of new marker sets or eventually other appropriate approaches (e.g., behavioural studies) should be performed to confirm or to infirm these hypotheses.

## Supporting information

S1 Fig Maximum-likelihood tree reconstructed from the *Apistogramma* concatenated sequence dataset of the mitochondrial cytochrome *b* and cytochrome *c* oxydase I genes and the Tmo-4C4 nuclear locus. Sequences are reported in the S2 Table. Numbers at nodes are for bootstrap percentages (≥ 50%) and posterior probabilities (≥ 0.85). Black circles indicates nodes with BP = 100% and PP = 1.00, while grey circles are for nodes with a weak support (BP < 50% and PP < 0.85). Nodes with “-”are weakly supported in maximum-likelihood approach or Bayesian inference.
(TIF)

S2 Fig Simplified maximum-likelihood tree reconstructed from the *Apistogramma* sequence dataset of the Tmo-4C4 nuclear locus. Sequences are reported in the S2 Table. Numbers at nodes are for bootstrap percentages (≥ 50%) and posterior probabilities (≥ 0.85). Grey circles are for nodes with a weak support (BP < 50% and PP < 0.85). Grey circles are for nodes with a weak support (BP < 50% and PP < 0.85). Nodes with “-”are weakly supported in maximum-likelihood approach or Bayesian inference.
(TIF)

S3 Fig Simplified maximum-likelihood tree reconstructed from the *Apistogramma* sequence dataset of the mitochondrial cytochrome *b* gene. Sequences are reported in the S2 Table. Numbers at nodes are for bootstrap percentages (≥ 50%) and posterior probabilities (≥ 0.80). Black circles indicates nodes with BP = 100% and PP = 1.00, while grey circles are for nodes with a weak support (BP < 50% and PP < 0.80). Nodes with “-”are weakly supported in maximum-likelihood approach or Bayesian inference.
(TIF)

S4 Fig Simplified maximum-likelihood tree reconstructed from the *Apistogramma* sequence dataset of the mitochondrial cytochrome *c* oxydase I gene. Sequences are reported in the S2 Table. Numbers at nodes are for bootstrap percentages (≥ 50%) and posterior probabilities (≥ 0.85). Black circles indicates nodes with BP = 100% and PP = 1.00, while grey circles are for nodes with a weak support (BP < 50% and PP < 0.85). Nodes with “-”are weakly supported in maximum-likelihood approach or Bayesian inference.
(TIF)

S1 Table Number of morphospecies and phylogenetic clades for each marker and the concatenated dataset. (PDF)

S2 Table Detailed list of the *Apistogramma* labels and sampling localities. Accession numbers for sequences produced in the frame of the present study and previously submitted to GenBank are provided for the cytochrome *b* and cytochrome *c* oxydase I genes and the Tmo-4C4 nuclear locus. Haplotypes from concatenated mitochondrial and nuclear markers are also listed. The tissue provider or references for sequences from GenBank are indicated. GenBank sequences of a given genus with * were combined and used as outgroup for the concatenated analysis.
(PDF)

S3 Table Genetic distance within and between *Apistogramma* species and the outgroup. (PDF)

S1 File XML file generated by BEAUti v2.4.4 in order to run the StarBEAST2 analysis with BEAST v2.4.4. (PDF)
