# Supplementary figures and images for: Tempo and rates of diversification in the South American cichlid genus Apistogramma (Teleostei: Perciformes: Cichlidae)
Source: PLoS One. 2017 Sep 5;12(9):e0182618. doi: 10.1371/journal.pone.0182618 (PMC5584756; doi:10.1371/journal.pone.0182618)

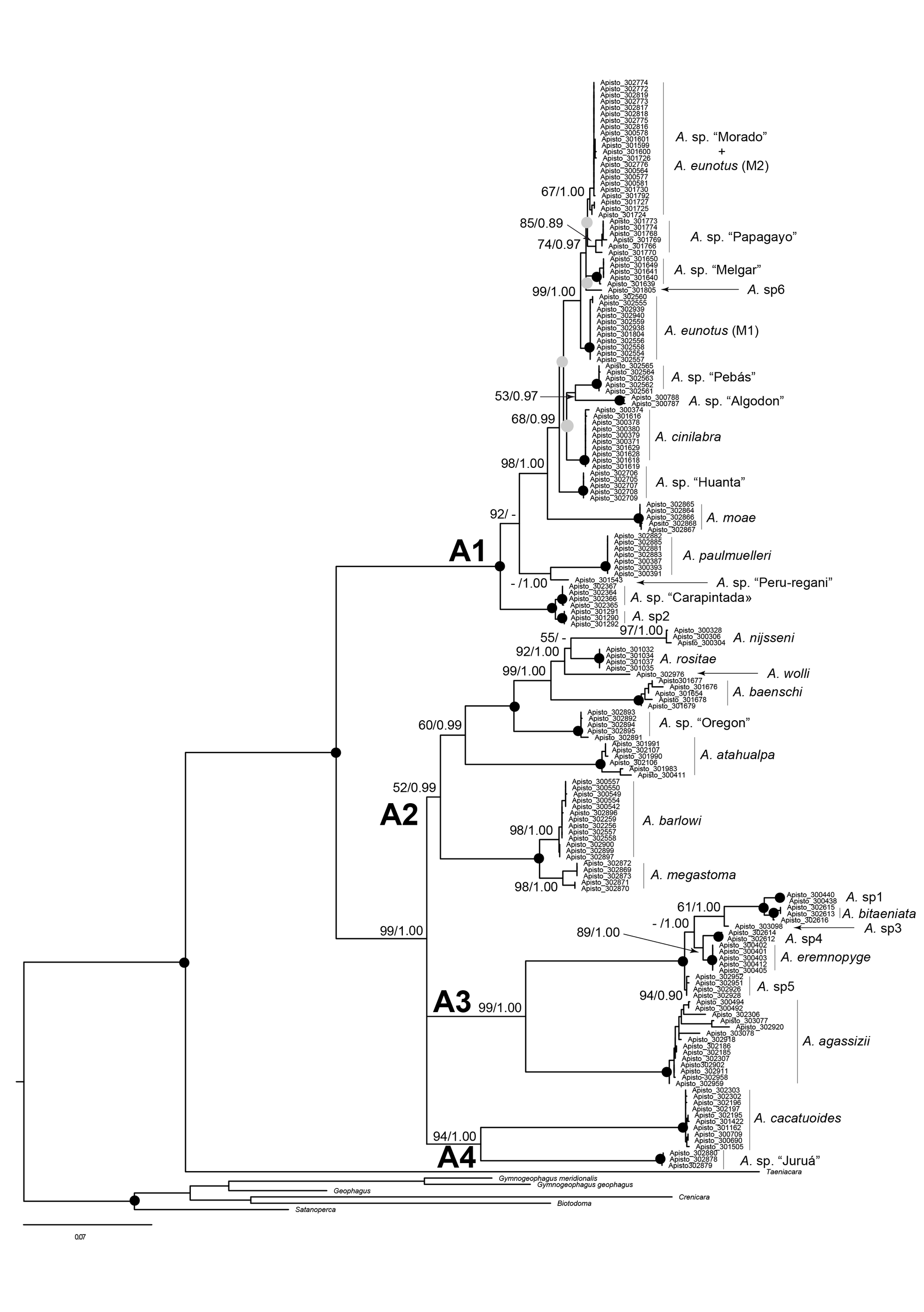

Supplement: S1 Fig — Sequences are reported in the S2 Table. Numbers at nodes are for bootstrap percentages (≥ 50%) and posterior probabilities (≥ 0.85). Black circles indicates nodes with BP = 100% and PP = 1.00, while grey circles are for nodes with a weak support (BP < 50% and PP < 0.85). Nodes with “-”are weakly supported in maximum-likelihood approach or Bayesian inference. (TIF) [file pone.0182618.s001.tif]

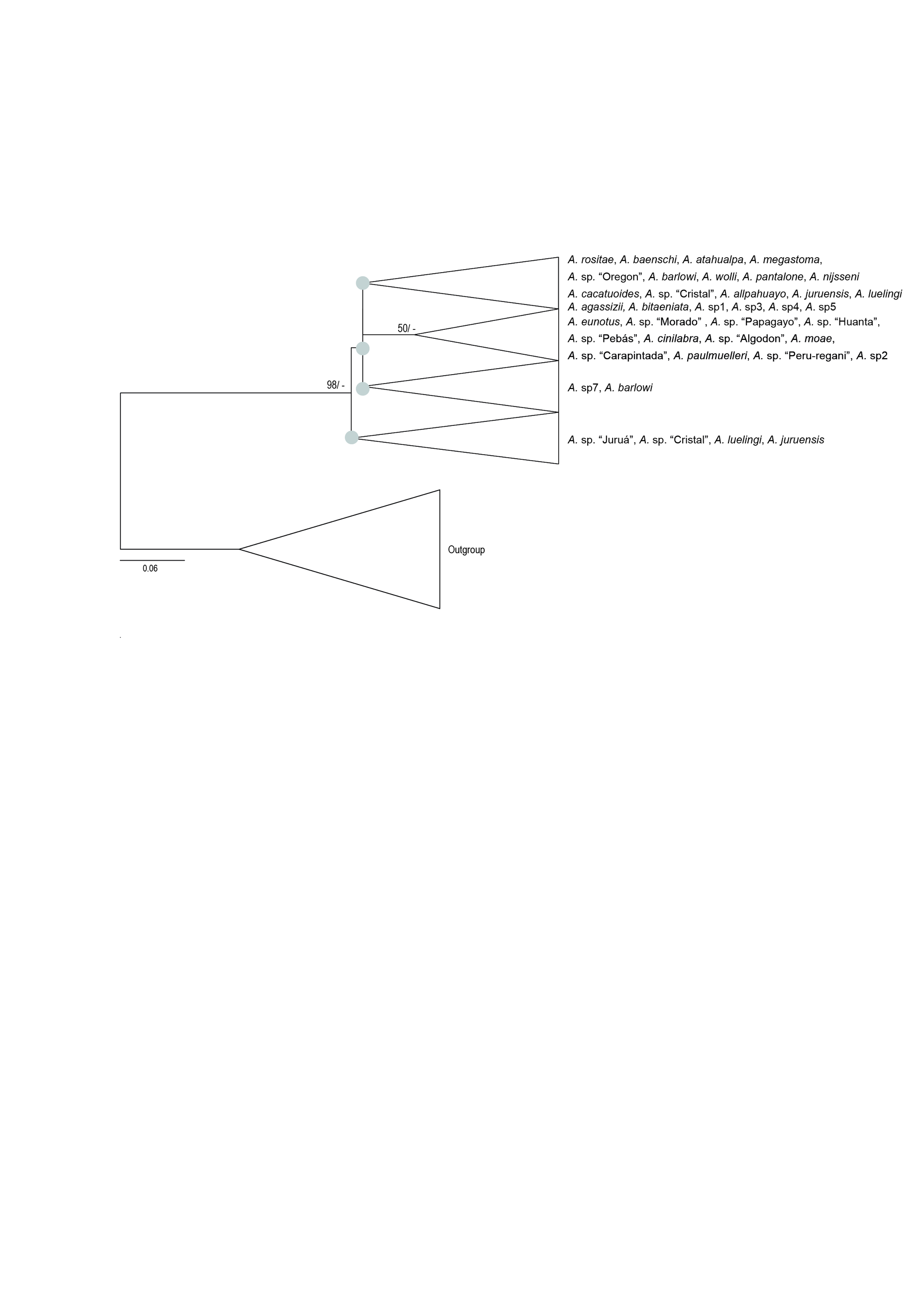

Supplement: S2 Fig — Sequences are reported in the S2 Table. Numbers at nodes are for bootstrap percentages (≥ 50%) and posterior probabilities (≥ 0.85). Grey circles are for nodes with a weak support (BP < 50% and PP < 0.85). Grey circles are for nodes with a weak support (BP < 50% and PP < 0.85). Nodes with “-”are weakly supported in maximum-likelihood approach or Bayesian inference. (TIF) [file pone.0182618.s002.tif]

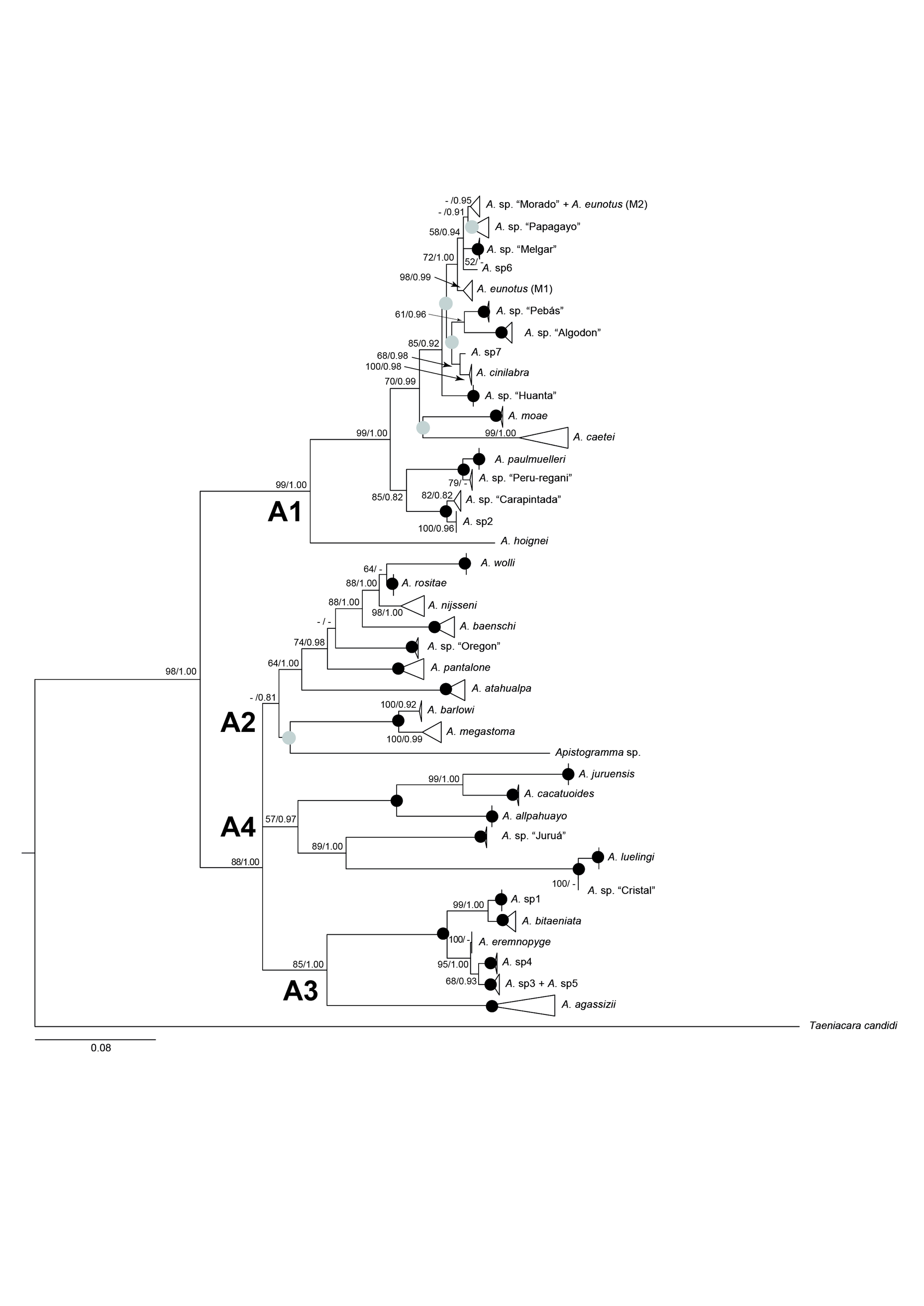

Supplement: S3 Fig — Sequences are reported in the S2 Table. Numbers at nodes are for bootstrap percentages (≥ 50%) and posterior probabilities (≥ 0.80). Black circles indicates nodes with BP = 100% and PP = 1.00, while grey circles are for nodes with a weak support (BP < 50% and PP < 0.80). Nodes with “-”are weakly supported in maximum-likelihood approach or Bayesian inference. (TIF) [file pone.0182618.s003.tif]

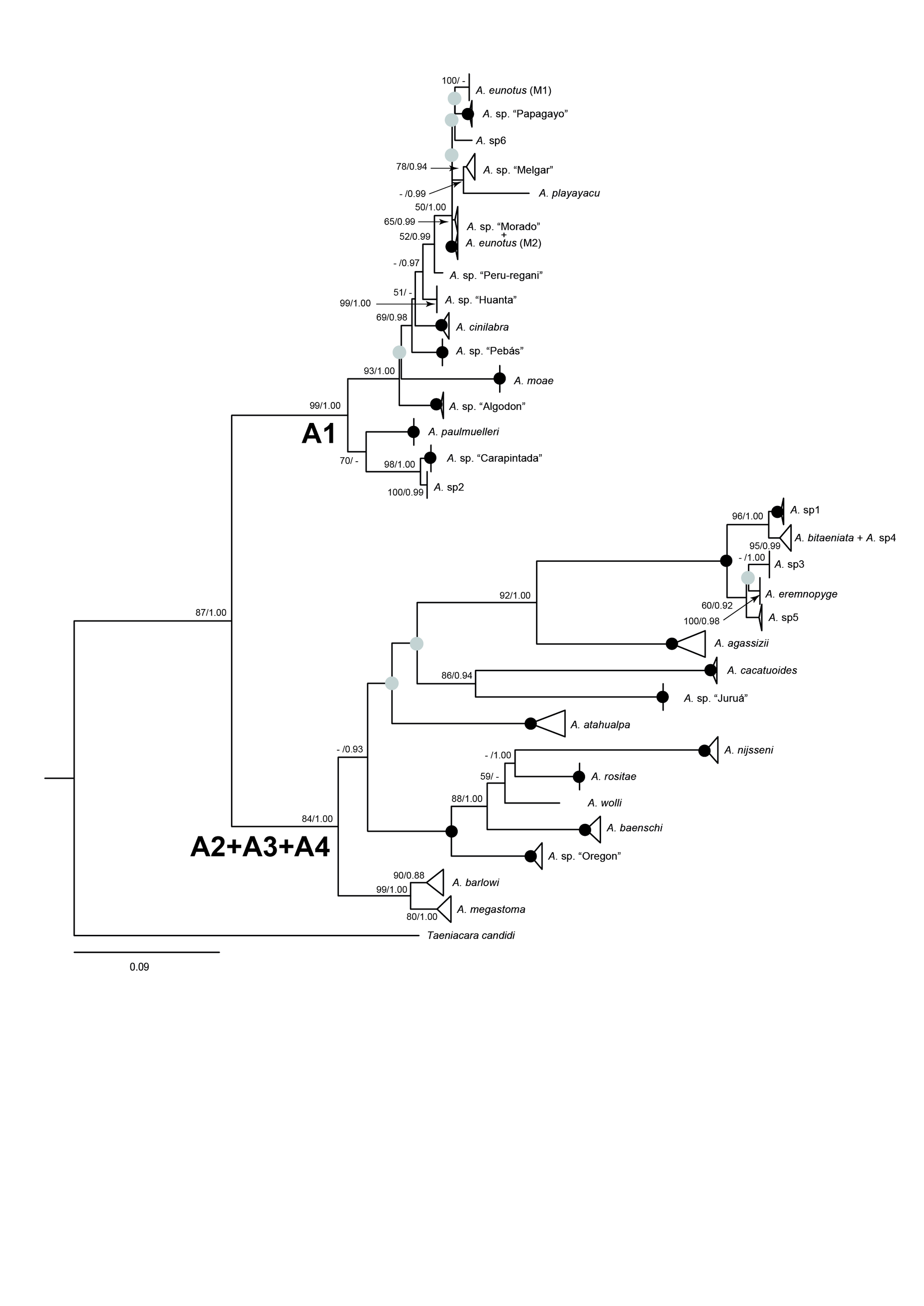

Supplement: S4 Fig — Sequences are reported in the S2 Table. Numbers at nodes are for bootstrap percentages (≥ 50%) and posterior probabilities (≥ 0.85). Black circles indicates nodes with BP = 100% and PP = 1.00, while grey circles are for nodes with a weak support (BP < 50% and PP < 0.85). Nodes with “-”are weakly supported in maximum-likelihood approach or Bayesian inference. (TIF) [file pone.0182618.s004.tif]
