# Supplementary material for: Tempo and rates of diversification in the South American cichlid genus Apistogramma (Teleostei: Perciformes: Cichlidae)
Source: PLoS One. 2017 Sep 5;12(9):e0182618. doi: 10.1371/journal.pone.0182618 (PMC5584756; doi:10.1371/journal.pone.0182618)
Supplement: S1 Table — (PDF) [file pone.0182618.s005.pdf]

Dataset	Morphospecies	Phylogenetic Clades
Tmo-4C4	41	-
Cytochrome *b*	41	39
Cytochrome *c* Oxidase I	31	31
Concatenated dataset	30	31
