# Supplementary material for: Tempo and rates of diversification in the South American cichlid genus Apistogramma (Teleostei: Perciformes: Cichlidae)
Source: PLoS One. 2017 Sep 5;12(9):e0182618. doi: 10.1371/journal.pone.0182618 (PMC5584756; doi:10.1371/journal.pone.0182618)
Supplement: S2 Table — Accession numbers for sequences produced in the frame of the present study and previously submitted to GenBank are provided for the cytochrome b and cytochrome c oxydase I genes and the Tmo-4C4 nuclear locus. Haplotypes from concatenated mitochondrial and nuclear markers are also listed. The tissue provider or references for sequences from GenBank are indicated. GenBank sequences of a given genus with * were combined and used as outgroup for the concatenated analysis. (PDF) [file pone.0182618.s006.pdf]

S2 Table Detailed list of the *Apistogramma* labels and sampling localities. Accession numbers for sequences produced in the frame of the present study and previously submitted to GenBank are provided for the cytochrome *b* and cytochrome *c* oxydase I genes and the Tmo-4C4 nuclear locus. Haplotypes from concatenated mitochondrial and nuclear markers are also listed. The tissue provider or references for sequences from GenBank are indicated. GenBank sequences of a given genus with * were combined and used as outgroup for the concatenated analysis.

Phylogenetic clades	Locality	Label	Tmo4C4	cytb	COI	Haplotypes	Tissue provider/References
	River Amazon, Quebrada Oran, Loreto Peru	300492	LN678884	LN678759	LN679002	Ap_24	IIAP[a]
		300494	LN678885	LN678760	LN679003	Ap_25	IIAP
		302185	LN678886	LN678761	LN679004	Ap_40	IIAP
	River Amazon, Pevas, Loreto, Peru	302186	LN678887	LN678762	LN679005	Ap_40	IIAP
		302306	-	LN678763	LN679006	Ap_46	IIAP
		302307	-	LN678764	LN679007	Ap_47	IIAP
		302919	LT617356	-	LT617281	-	IIAP
	River Amazon, Quebrada Tacsha Mendoza, Loreto, Peru 05°20'25.10"S 73°50'12.80"W	302935	LT617357	-	-	-	IIAP
		302936	LT617358	-	-	-	IIAP
		302996	LT617359	LT617119	-	-	IIAP
		303077	LT617360	LT617120	LT617282	Ap_93	IIAP
	River Amazon, Quebrada Orancillo, Loreto, Peru 03°2'13.50"S 72°30'45.70"W	302920	LT617361	LT617121	LT617283	Ap_84	IIAP
		302934	LT617362	LT617122	-	-	IIAP
		302968	LT617363	LT617123	-	-	IIAP
		302990	LT617364	-	-	-	IIAP
	River Amazon, Quebrada Roca Eterna "B", Loreto, Peru	303126	LT617365	LT617124	-	-	IIAP
		303127	LT617366	LT617125	-	-	IIAP
Apistogramma agassizii (Steindachner, 1875)	River Ucayali, Quebrada Ramiro, Loreto Peru 03°53'46.80"S 73°39'52.10"W	302902	LT617367	LT617126	LT617284	Ap_82	IIAP
		302958	LT617368	LT617127	LT617285	Ap_90	IIAP
		302959	LT617369	LT617128	LT617286	Ap_91	IIAP
	River Ucayali, Quebrada Pradera, Loreto Peru 04°54'24.10"S 73°39'47.50"W	302911	LT617370	LT617129	LT617287	Ap_82	IIAP
		303023	LT617371	LT617130	-	-	IIAP
	River Tapiche, Quebrada Antonia, Loreto, Peru 05°20'25.10"S 73°50'12.80"W	302918	LT617372	LT617131	LT617288	Ap_83	IIAP
	River Ucayali, Quebrada Búfalo, Loreto, Peru 04°53'59.10"S 73°39'33.54"W	303021	LT617373	LT617132	-	-	IIAP
		303024	LT617374	LT617133	-	-	IIAP
		303112	LT617375	LT617134	-	-	IIAP
	River Tapiche, Quebrada Carbón, Loreto, Peru 05°20'02.10"S 73°51'20.50"W	303078	LT617376	LT617135	LT617289	Ap_94	IIAP
		303134	LT617377	LT617136	-	-	IIAP
	River Ucayali, Quebrada Rain, Loreto, Peru 04°54'48.30"S 73°39'42.90"W	303084	LT617378	LT617137	-	-	IIAP
		303085	LT617379	LT617138	-	-	IIAP
		303111	LT617380	LT617139	-	-	IIAP
	River Nanay, Quebrada Shushuna, Loreto, Peru 03°48'59.90"S 73°20'44.80"W	300921	LT617381	-	-	-	IIAP
		300922	LT617382	LT617140	-	-	IIAP
	-	303137	LT617383	LT617141	-	-	IIAP
		302158	LT617389	LT617146	-	-	IIAP
	River Nanay, Reserve Allpahuayo, Loreto, Peru 03°59'11.10"S 73°25'51.40"W	302159	LT617390	LT617147	-	-	IIAP
		302160	LT617391	-	-	-	IIAP
		302161	LT617392	LT617148	-	-	IIAP
A. allpahuayo Römer et al., 2012		302162	LT617393	LT617149	-	-	IIAP
		302164	LT617394	LT617150	-	-	IIAP
	River Itaya, Loreto, Peru	302165	LT617395	LT617151	-	-	IIAP
		302166	LT617396	LT617152	-	-	IIAP
		302168	LT617397	LT617153	-	-	IIAP
	Allpahuayo Mishana Forest Reserve, Loreto, Peru 03°58'51.10"S 73°25'21.10"W	MTD F 32696 PT	LT617398	LT617154	-	-	UR
		302106	LN678876	LN678751	LN678997	Ap_38	IIAP
		302107	LT617399	LN678752	LN678998	Ap_39	IIAP
		302108	LT617400	LN678753	LN678999	-	IIAP
	River Amazon, Pevas, Loreto, Peru 03°17'46"S 72°00'00.10"W	302109	LN678877	-	LN679000	-	IIAP
		302171	LN678879	LN678754	-	-	IIAP
		302172	LN678880	LN678755	-	-	IIAP
		302173	LN678881	LN678756	-	-	IIAP
		302174	LN678882	LN678757	-	-	IIAP
A. atahualpa Römer, 1997		302175	LN678883	LN678758	LN679001	-	IIAP
		300411	LT617401	LT617156	LT617292	Ap_60	IIAP
		301604	LT617402	LT617157	-	-	IIAP
	River Nanay, Road Iquitos-Nauta Km 22, Loreto, Peru	301605	LT617403	-	-	-	IIAP
		301606	LT617404	LT617158	-	-	IIAP
		301607	LT617405	LT617159	-	-	IIAP
		301982	LT617406	LT617160	-	-	IIAP
	River Nanay, Loreto, Peru 03°52'56.30"S 73°23'19.20"W	301983	LT617407	LT617161	LT617293	Ap_66	IIAP
		301989	LT617408	-	LT617294	-	IIAP
		301990	LT617409	LT617162	LT617295	Ap_67	IIAP
		301991	LT617410	LT617163	LT617296	Ap_39	IIAP
		301654	LN678840	LN678716	LN678963	Ap_8	IIAP
A. baenschi Römer et al., 2004		301676	LN678841	LN678717	LN678964	Ap_9	IIAP
	Yurimaguas, Loreto, Peru	301677	LN678842	LN678718	LN678965	Ap_10	IIAP
		301678	LN678843	LN678719	LN678966	Ap_11	IIAP
		301679	LN678844	LN678720	LN678967	Ap_12	IIAP
		300542	LN678855	LN678730	LN678976	Ap_17	IIAP
	River Amazon, Roca Eterna, Quebrada Agua Negrilla Loreto, Peru	300549	LT617411	LN678731	LN678977	Ap_18	IIAP
		300550	LN678856	LN678732	LN678978	Ap_17	IIAP
		300554	LN678857	LN678733	LN678979	Ap_17	IIAP
		300557	LN678858	LN678734	LN678980	Ap_17	IIAP
		302256	LN678859	LN678735	LN678981	Ap_68	IIAP
	River Amazon, Roca Eterna, Loreto, Peru 03°26'37.60"S 72°30'54.20"W	302257	LN678860	LN678736	LN678982	Ap_68	IIAP
		302258	LN678861	LN678737	LN678983	Ap_68	IIAP
		302259	LN678862	LN678738	LN678984	Ap_68	IIAP
		302260	LN678863	LN678739	LN678985	-	IIAP
		302896	LT617412	LT617164	LT617297	Ap_80	IIAP
		302897	LT617413	LT617165	LT617298	Ap_81	IIAP
A. barlowi Römer & Hahn, 2008	River Amazon, Roca Eterna, Loreto, Peru	302898	LT617414	LT617166	LT617299	-	IIAP
		302899	LT617415	LT617167	LT617300	Ap_81	IIAP
		302900	LT617416	LT617168	LT617301	Ap_81	IIAP
	-	303150	LT617417	LT617169	-	-	IIAP
		303151	LT617418	LT617170	-	-	IIAP
		303152	LT617419	LT617171	-	-	IIAP
	River Amazon, Pevas, Loreto, Peru	303153	LT617420	LT617172	-	-	IIAP
		303154	LT617421	LT617173	-	-	IIAP
		303155	LT617422	LT617174	-	-	IIAP
	River Amazon, Loreto, Peru	303128	LT617423	-	LT617302	-	IIAP
	River Amazon, Pevas, Loreto, Peru 03°17'46"S 72°00'00.10"W	302110	LN678878	-	LT617303	-	IIAP
	River Ampiyacu, Loreto, Peru	MUSM 43060	LT617424	-	LT617304	-	UR
	03°10'S 71°55'W	MUSM 52455	LT617425	LT617175	-		UR
	River Amazon, Pevas, Loreto, Peru 03°18'60"S 72°50'84.10"W	302613	LN678897	LN678776	LN679018	Ap_54	IIAP
		302615	LN678899	LN678778	LN679019	Ap_54	IIAP
A. bitaeniata Pellegrin, 1936		302616	LN678900	LN678779	LN679020	Ap_56	IIAP
	River Yanasyacu, Tributary of the River Amazon	303122	LT617426	LT617176	-	-	IIAP
	Pueblo Brillo Nuevo, Loreto, Peru	303146	LT617427	LT617177	-	-	IIAP
	-	UMMZ243211	DQ119243	-	DQ119214	-	Chakrabarty (2006)
	River Amazon, Roca Eterna, Quebrada Chontilla, Loreto, Peru	300690	LN678888	LN678765	LN679008	Ap_20	IIAP
	03°26'41.30"S 72°32'10.90"W	300709	LN678889	LN678766	LN679009	Ap_20	IIAP
	Quebrada Felipe Caño, Loreto, Peru 03°48'59.60"S 73°18'25.70"W	301162	LN678890	LN678767	LN679010	Ap_21	IIAP
	River Itaya, Quebrada Huillacocha, Loreto, Peru 03°46'31.60"S 73°14'52.10"W	301422	LN678891	LN678768	LN679011	Ap_22	IIAP
A. cacatuoides Hoedeman, 1951	Quebrada Horizonte, Loreto, Peru 04°02'29.70"S 73°28'04.60"W	301505	LN678892	LN678769	LN679012	Ap_23	IIAP
		302195	LN678893	LN678770	LN679013	Ap_41	IIAP
		302196	LN678894	LN678771	LN679014	Ap_42	IIAP
	River Amazon, Pevas, Loreto, Peru	302197	LN678895	LN678772	LN679015	Ap_43	IIAP
		302302	-	LN678773	LN679016	Ap_44	IIAP
		302303	-	LN678774	LN679017	Ap_45	IIAP
		301616	LN678830	LN678706	LN678953	Ap_4	IIAP
		301618	LN678831	LN678707	LN678954	Ap_5	IIAP
	River Itaya, Road Iquitos-Nauta km 78, Loreto, Peru	301619	LN678832	LN678708	LN678955	Ap_5	IIAP
		301628	LN678833	LN678709	LN678956	Ap_5	IIAP
		301629	LN678834	LN678710	LN678957	Ap_5	IIAP
		300371	LT617428	LT617178	LT617305	Ap_5	IIAP
		300374	LT617429	LT617179	LT617306	Ap_58	IIAP
A. cinilabra Römer et al., 2011	River Itaya, Cocha Rother, Loreto, Peru	300378	LT617430	LT617180	LT617307	Ap_5	IIAP
		300379	LT617431	LT617181	LT617308	Ap_5	IIAP
		300380	LT617432	LT617182	LT617309	Ap_5	IIAP
		301061	LT617433	LT617183	-	-	IIAP
		301062	LT617434	LT617184	-	-	IIAP
	River Itaya, Road Iquitos-Nauta km 78, Loreto, Peru	301063	LT617435	LT617185	-	-	IIAP
		301064	LT617436	LT617186	-	-	IIAP
		301065	LT617437	LT617187	-	-	IIAP
		300401	LN678850	LN678725	LN678971	Ap_16	IIAP
A. eremnopyge Ready & Kullander, 2004		300402	LN678851	LN678726	LN678972	Ap_16	IIAP
	River Itaya, Cocha Diamente 3, Loreto, Peru	300403	LN678852	LN678727	LN678973	Ap_16	IIAP
		300405	LN678853	LN678728	LN678974	Ap_16	IIAP
		300412	LN678854	LN678729	LN678975	Ap_16	IIAP
		301791	LN678914	LN678790	-	-	IIAP
	Tributary of the River Morona, San Pablo, Loreto, Peru	301793	LN678915	LN678791	LN679032	Ap_36	IIAP
		301804	LN678916	LN678792	LN679033	Ap_36	IIAP
		302554	LN678917	LN678794	LN679035	Ap_36	IIAP
		302555	LN678918	LN678795	LN679036	Ap_50	IIAP
	River Amazon, Pevas, Loreto, Peru 03°29'77.30"S 72°03'69.60"W	302556	LN678919	LN678796	LN679037	Ap_36	IIAP
		302557	LN678920	LN678797	LN679038	Ap_36	IIAP
A. eunotus (M1) Kullander, 1981		302558	LN678921	LN678798	LN679039	Ap_36	IIAP
	River Amazon, Roca Eterna, Loreto Peru	301710	LT617438	LT617188	-	-	IIAP
		302938	LT617439	LT617189	LT617310	Ap_36	IIAP
	Natural Reserve Pacaya Samiria, Loreto, Peru	302939	-	LT617190	LT617311	Ap_87	IIAP
		302940	LT617440	LT617191	LT617312	Ap_36	IIAP
		302941	-	-	LT617313	-	IIAP
	River Amazon, Loreto, Peru	302559	LT617441	LT617192	LT617314	Ap_36	IIAP
		302560	LT617442	LT617193	LT617315	Ap_50	IIAP
	River Itaya, Loreto, Peru	302167	LT617443	LT617194	-	-	IIAP
	River Amazon, Roca Eterna, Quebrada Chontilla, Loreto, Peru	301599	LN678922	LN678799	LN679040	Ap_2	IIAP
A. eunotus (M2) Kullander, 1981	03°26'44"S 72°32'13"W	301600	LN678923	LN678800	LN679041	Ap_3	IIAP
		301601	LN678924	LN678801	LN679042	Ap_2	IIAP
	Tributary of the River Morona, San Pablo, Loreto, Peru	301792	LN678925	LN678802	LN679043	Ap_35	IIAP
		302859	LT617444	LT617195	-	-	IIAP
A. juruensis Kullander, 1986		302860	LT617445	LT617196	-	-	IIAP
	River Juruá, Brazil	302861	LT617446	LT617197	-	-	IIAP

Kullander, 1986		302862	LT617447	LT617198	-	-	IIAP
		302863	LT617448	LT617199	-	-	IIAP
A. luelingi Kullander, 1976	Tributary of River Maderira, Puerto Maldonado, Bolivia	302665	LT617449	LT617200	-	-	IIAP
		302666	LT617450	LT617201	-	-	IIAP
		302667	LT617451	LT617202	-	-	IIAP
		302668	LT617452	LT617203	-	-	IIAP
		302669	LT617453	LT617204	-	-	IIAP
A. megastoma Römer et al., 2017	River Jutai, Brazil	301019	LT617500	LT617255	-	-	IIAP
		301020	LT617501	LT617256	-	-	IIAP
		302869	LT617502	LT617257	LT617339	Ap_73	IIAP
		302870	LT617503	LT617258	LT617340	Ap_74	IIAP
		302871	LT617504	LT617259	LT617341	Ap_74	IIAP
		302872	LT617505	LT617260	LT617342	Ap_73	IIAP
		302873	LT617506	LT617261	LT617343	Ap_73	IIAP
A. moae Kullander, 1980	River Moa, Tributary of the River Juruá, Brazil	302864	LT617454	LT617205	LT617316	Ap_70	IIAP
		302865	LT617455	LT617206	LT617317	Ap_70	IIAP
		302866	LT617456	LT617207	LT617318	Ap_70	IIAP
		302867	LT617457	LT617208	LT617319	Ap_71	IIAP
		302868	LT617458	LT617209	LT617320	Ap_72	IIAP
A. nijsseni Kullander, 1979	River Ucayali, Loreto, Peru 04°55'04"S 73°33'32.20"W	300292	LN678845	LN678721	-	-	IIAP
		300304	LN678846	LN678722	LN678968	Ap_13	IIAP
		300306	LN678847	LN678723	LN678969	Ap_14	IIAP
		300328	LN678848	LN678724	LN678970	Ap_15	IIAP
		300361	LN678849	-	-	-	IIAP
	Road from Genaro Herrera to Los Angamos, Loreto Peru 04°56'25"S 73°30'24"W	MTD F uncatalogued	LT617459	LT617210	-	-	UR
A. pantalone Römer et al., 2006	-	303156	LT617473	LT617224	-	-	IIAP
		303157	LT617474	LT617225	-	-	IIAP
		303158	LT617475	LT617226	-	-	IIAP
		303159	LT617476	LT617227	-	-	IIAP
		303160	LT617477	LT617228	-	-	IIAP
	Small tributary of River Tígre, Loreto, Peru 03°41'20"S 74°35'37"W	MUSM 43063	LT617478	LT617229	-	-	UR
		MUSM uncatalogued	LT617479	LT617230	-	-	UR
A. paulmuelleri Römer et al., 2013	River Itaya, Cocha Apache, Loreto, Peru	300387	LT617483	LT617234	LT617328	Ap_59	IIAP
		300391	LT617484	LT617235	LT617329	Ap_59	IIAP
		300393	LT617485	LT617236	LT617330	Ap_59	IIAP
	River Amazon, Loreto, Peru 04°23'29.10"S 73°34'08.90"W	MUSM 41810 PT_1	-	LT617237	-	-	IIAP
		MTD F 32637 PT_1	LT617486	LT617238	-	-	IIAP
		MUSM 41810 PT_2	-	LT617239	-	-	IIAP
		MUSM 41811 PT	-	LT617240	-	-	IIAP
		CAS 233864 PT	-	LT617241	-	-	IIAP
	River Itaya, Carretera Iquitos-Nauta, Loreto, Peru	MTD F 32637 PT_2	LT617487	LT617242	LT617331	Ap_59	IIAP
		MTD F 32637 PT_3	LT617488	LT617243	LT617332	Ap_59	IIAP
		MUSM 41809 PT	LT617489	LT617244	LT617333	Ap_59	IIAP
		CAS 233863 PT	LT617490	LT617245	LT617334	-	IIAP
		MTD F 32636 PT	LT617491	LT617246	LT617335	Ap_59	IIAP
A. playayacu Römer et al., 2011	River Napo system, Quebrada Playayacu Quebrada entering Limoncocha, Ecuador 00°22'S 76°36'W	FMNH 101589	-	-	LT617327	-	UR
A. rositae Römer et al., 2006	River Santiago, near the frontier between Peru and Ecuador	301032	LN678825	LN678702	LN678948	Ap_1	IIAP
		301034	LN678826	LN678703	LN678949	Ap_1	IIAP
		301035	LN678827	LN678704	LN678950	Ap_1	IIAP
		301037	LN678828	LN678705	LN678951	Ap_1	IIAP
		301038	LN678829	-	LN678952	-	IIAP
A. wolli Römer et al., 2015	River Napo, Loreto, Peru	302976	LT617480	LT617231	LT617326	Ap_92	IIAP
		302977	LT617481	LT617232	-	-	IIAP
		303011	LT617482	LT617233	-	-	IIAP
A. sp. "Algodon"	River Ampiyacu, Tributary of the River Amazon Loreto, Peru	300663	LT617384	-	-	-	IIAP
		300787	LT617385	LT617142	LT617290	Ap_63	IIAP
		300788	LT617386	LT617143	LT617291	Ap_64	IIAP
		300789	LT617387	LT617144	-	-	IIAP
		300790	LT617388	LT617145	-	-	IIAP
A. sp. "Carapintada"	River Morona, San Pablo, Loreto, Peru	301784	-	LN678746	-	-	IIAP
		301785	LN678871	-	-	-	IIAP
	River Amazon, San Pablo, Loreto, Peru 04º02'42"S 71º05'24.60"W	302364	LN678872	LN678747	LN678993	Ap_48	IIAP
		302365	LN678873	LN678748	LN678994	Ap_49	IIAP
		302366	LN678874	LN678749	LN678995	Ap_48	IIAP
		302367	LN678875	LN678750	LN678996	Ap_48	IIAP
A. sp. "Cristal"	Madre de Dios, Peru	302192	LT617492	LT617247	-	-	IIAP
		302193	LT617493	LT617248	-	-	IIAP
		302194	LT617494	LT617249	-	-	IIAP
A. sp. "Huanta"	River Amazon, Pevas, Loreto, Peru 03°29'77.30"S 72°03'69.60"W	302451	LN678901	-	-	-	IIAP
		302452	LN678902	-	LN679021	-	IIAP
		302453	LN678903	-	-	-	IIAP
		302705	LN678904	LN678780	LN679022	Ap_69	IIAP
		302706	LN678905	LN678781	LN679023	Ap_69	IIAP
		302707	LN678906	LN678782	LN679024	Ap_69	IIAP
		302708	LN678907	LN678783	LN679025	Ap_69	IIAP
		302709	LN678908	LN678784	LN679026	Ap_69	IIAP
A. sp. "Juruá"	River Juruá, Brazil	302876	LT617495	LT617250	-	-	IIAP
		302877	LT617496	LT617251	-	-	IIAP
		302878	LT617497	LT617252	LT617336	Ap_75	IIAP
		302879	LT617498	LT617253	LT617337	Ap_76	IIAP
		302880	LT617499	LT617254	LT617338	Ap_75	IIAP
A. sp. "Melgar"	River Ampiyacu, Pevas-Oran area, Loreto, Peru	301639	LN678835	LN678711	LN678958	Ap_6	IIAP
		301640	LN678836	LN678712	LN678959	Ap_7	IIAP
		301641	LN678837	LN678713	LN678960	Ap_7	IIAP
		301649	LN678838	LN678714	LN678961	Ap_7	IIAP
		301650	LN678839	LN678715	LN678962	Ap_7	IIAP
A. sp. "Morado"	River Amazon, Roca Eterna, Quebrada Alvin, Loreto, Peru 03°25'47"S 72°29'06"W	300564	LN678926	LN678803	LN679044	Ap_19	IIAP
		300567	LN678927	-	LN679045	-	IIAP
		300577	LN678928	LN678804	LN679046	Ap_19	IIAP
		300578	LN678929	LN678805	LN679047	Ap_19	IIAP
		300581	LN678930	LN678806	LN679048	Ap_19	IIAP
	River Amazon, Pevas, Loreto, Peru	301724	LN678931	LN678807	LN679049	Ap_27	IIAP
		301725	-	LN678808	LN679050	Ap_28	IIAP
		301726	LN678932	LN678809	LN679051	Ap_29	IIAP
		301727	LN678933	LN678810	LN679052	Ap_30	IIAP
		301730	LN678934	LN678811	LN679053	Ap_19	IIAP
		301711	LT617465	LT617216	-	-	IIAP
		301716	LT617466	LT617217	-	-	IIAP
		301733	LT617467	LT617218	-	-	IIAP
		301734	LT617468	LT617219	-	-	IIAP
		301735	LT617469	LT617220	-	-	IIAP
		301736	LT617470	LT617221	-	-	IIAP
		301737	LT617471	LT617222	-	-	IIAP
		301749	LT617472	LT617223	-	-	IIAP
	River Amazon, Roca Eterna, Loreto, Peru 03°27'33.40"S 72°31'30.60"W	302772	LN678935	LN678812	LN679054	Ap_19	IIAP
		302773	LN678936	LN678813	LN679055	Ap_19	IIAP
		302774	-	LN678814	LN679056	Ap_57	IIAP
		302775	LN678937	LN678815	LN679057	Ap_19	IIAP
		302776	LN678938	LN678816	LN679058	Ap_19	IIAP
	River Amazon, Roca Eterna, Loreto, Peru 03°25'45.90"S 72°30'43.20"W	302816	LN678939	LN678817	LN679059	Ap_19	IIAP
		302817	LN678940	LN678818	LN679060	Ap_19	IIAP
		302818	LN678941	LN678819	LN679061	Ap_19	IIAP
		302819	LN678942	LN678820	LN679062	Ap_19	IIAP
		302820	LN678943	LN678821	-	-	IIAP
A. sp. "Oregon"	River Itaya, Loreto, Peru	302891	LT617460	LT617211	LT617321	Ap_77	IIAP
		302892	LT617461	LT617212	LT617322	Ap_78	IIAP
		302893	LT617462	LT617213	LT617323	Ap_79	IIAP
		302894	LT617463	LT617214	LT617324	Ap_79	IIAP
		302895	LT617464	LT617215	LT617325	Ap_79	IIAP
A. sp. "Papagayo"[c]	River Amazon, Pevas, Loreto, Peru	301766	LN678864	LN678740	LN678986	Ap_31	IIAP
		301767	LN678865	-	LN678987	-	IIAP
		301768	LN678866	LN678741	LN678988	Ap_32	IIAP
		301769	LN678867	LN678742	LN678989	Ap_33	IIAP
		301770	LN678868	LN678743	LN678990	Ap_34	IIAP
		301773	LN678869	LN678744	LN678991	Ap_32	IIAP
		301774	LN678870	LN678745	LN678992	Ap_32	IIAP
A. sp. "Pebás"[c]	River Amazon, Pevas, Loreto, Peru 03°17'23.50"S 71°50'7.50"W	302561	LN678909	LN678785	LN679027	Ap_51	IIAP
		302562	LN678910	LN678786	LN679028	Ap_51	IIAP
		302563	LN678911	LN678787	LN679029	Ap_51	IIAP
		302564	LN678912	LN678788	LN679030	Ap_52	IIAP
		302565	LN678913	LN678789	LN679031	Ap_51	IIAP
A. sp. "Peru-regani"	River Nanay, Quebrada Huacamayo, Loreto, Peru 03°52'40.20"S 73°52'48.50"W	301543	LT617507	LT617262	LT617344	Ap_65	IIAP
		301544	-	LT617263	-	-	IIAP
		302152	-	LT617264	-	-	IIAP
		302153	-	LT617265	-	-	IIAP
	River Nanay, Quebrada Huacamayo, Loreto, Peru	302154	LT617508	LT617266	-	-	IIAP
		302155	-	LT617267	-	-	IIAP
		302156	LT617509	LT617268	-	-	IIAP
A. sp1	River Napo, Quebrada Commandancia, Loreto, Peru 03°24'99.30"S 73°06'17"W	300438	LT617510	LT617269	LT617345	Ap_61	IIAP
		300440	LT617511	LT617270	LT617346	Ap_62	IIAP
A. sp2	River Orosa, Pevas, Loreto, Peru	301290	LN678944	LN678822	LN679063	Ap_26	IIAP
		301291	LN678945	LN678823	LN679064	Ap_26	IIAP
		301292	LN678946	LN678824	LN679065	Ap_26	IIAP
	River Morona, San Pablo, Loreto, Peru	301783	LN678947	-	LN679066	-	IIAP
A. sp3	River Manati, Loreto, Peru	303098	LT617512	LT617271	LT617347	Ap_95	IIAP
		303113	LT617513	-	LT617348	-	IIAP
A. sp4	River Amazon, Pevas, Loreto, Peru 03°18'60"S 72°50'84.10"W	302612	LN678896	LN678775	LT617349	Ap_53	IIAP
		302614	LN678898	LN678777	LT617350	Ap_55	IIAP
A. sp5	River Nanay, Tascha Nanay, Loreto, Peru 03°54'05.90"S 73°43'22.10"W	301248	LT617514	-	-	-	IIAP
		301251	LT617515	LT617272	-	-	IIAP
	River Nanay, Cocha Nuevo, Loreto, Peru	302926	LT617516	LT617273	LT617351	Ap_85	IIAP
		302928	-	LT617274	LT617352	Ap_86	IIAP
	River Manati, Loreto, Peru	302951	LT617517	LT617275	LT617353	Ap_88	IIAP
		302952	LT617518	LT617276	LT617354	Ap_89	IIAP
	River Napo, Mouth of the River Amazon, Loreto, Peru	302998	LT617519	LT617277	-	-	IIAP
		302999	LT617520	LT617278	-	-	IIAP
A. sp6	Tributary of the River Morona, San Pablo, Loreto, Peru	301805	-	LN678793 LT617279	LT617355	Ap_37	IIAP
A. sp7	River Itaya, Loreto, Peru 04°05'56.20" 073°2'12.80"	301911	-	LT617280	-	-	IIAP
	River Jeju, Bragantina, Brazil	-	-	AJ557085	-	-	Ready et al. (2006)[b]
	River Capim, Bragantina, Brazil	-	-	AJ557095	-	-	Ready et al. (2006)

A. caetei	River Marapanim, Bragantina, Brazil	-	-	AJ557107	-	-	Ready et al. (2006)
Kullander, 1980	River Quatipuru, Bragantina, Brazil	-	-	AJ557121	-	-	Ready et al. (2006)
	River Piquioira, Bragantina, Brazil	-	-	AJ557127	-	-	Ready et al. (2006)
	-	-	EF470866	-	-	-	Genner et al. (2007)
A. hoignei Meinken, 1965	Cano Maporal, Venezuela	-	-	GU736927	-	-	López-Fernández et al. (2010)
Apistogramma sp.	-	-	AY662850	AF370656	-	-	Sparks & Smith (2004) / Farias et al. (2001)
**Outgroup**							
Acarichthys heckelii			AY662848				Sparks & Smith (2004)
Biotodoma cupido				GU736928			López-Fernández et al. (2010)
Biotodoma wavrini			EU888076*	AF370657*	EU888075*		Smith et al. (2008)/Farias et al. (2001)/Smith et al. (2008)
Crenicara punctulatum			EU888069*		EU888068*		Smith et al. (2008)
Crenicara sp.				AF370655*			Farias et al. (2001)
Dicrossus sp.			AY662855				Sparks & Smith (2004)
Geophagus abalios				GU736939			López-Fernández et al. (2010)
Geophagus brasiliensis			EF470869*	AF370659*	JN988868		Genner et al. (2007)/Farias et al. (2001)/Pereira et al. (2013)
			EU888082				Smith et al. (2008)
Geophagus dicrozoster				GU736941			López-Fernández et al. (2010)
Geophagus grammepareius				GU736942			López-Fernández et al. (2010)
Geophagus harreri				GU736943			López-Fernández et al. (2010)
Geophagus proximus					GU701784		Pereira et al. (2013)
					GU701786		Pereira et al. (2013)
					JN988869*		Pereira et al. (2013)
Geophagus steindachneri			DQ119246	AF370660			Chakrabarty (2006)/Farias et al. (2001)
Geophagus surinamensis				GU736944	JN026710		López-Fernández et al. (2010)/April et al. (2011)
Geophagus taeniopareius				GU736946			López-Fernández et al. (2010)
Gymnogeophagus balzanii				GU736950			López-Fernández et al. (2010)
Gymnogeophagus gymnogenys			GU817313*	AF370661*	EU888086*		Chakrabarty & Albert (unp.)/Farias et al. (2001)/Smith et al. (2008)
			EU888087				Smith et al. (2008)
Gymnogeophagus labiatus				AF370662			Farias et al. (2001)
Gymnogeophagus meridionalis				DQ448271*	JX111759*		Pereyra & Garcia (2008)/Rosso et al. (2012)
Gymnogeophagus rhabdotus				GU736951			López-Fernández et al. (2010)
Gymnogeophagus setequedas				GU736952			López-Fernández et al. (2010)
Mikrogeophagus altispinosus				GU736953	EU888090		López-Fernández et al. (2010)/Smith et al. (2008)
Mikrogeophagus ramirezi				GU736954			López-Fernández et al. (2010)
Satanoperca acuticeps				AF370663*			Farias et al. (2001)
Satanoperca daemon				GU736955			López-Fernández et al. (2010)
Satanoperca jurupari			GU817314*	AB018986			Chakrabarty & Albert (unp.)/Kumazawa et al. (1999)
Satanoperca leucosticta				GU736956	AY263861		López-Fernández et al. (2010)/Sparks & Smith (2004)
Satanoperca mapiritensis				GU736957			López-Fernández et al. (2010)
Satanoperca pappaterra					JN989214*		Pereira et al. (2013)
					JN989213		Pereira et al. (2013)
Taeniacara candidi				AF370665*	EU888093*		Farias et al. (2001)/Smith et al. (2008)

[a] IIAP = Instituto de Investigaciones de la Amazonía Peruana; UR = Uwe Römer

[b] References:

April, J., Mayden, R.L., Hanner, R.H., Bermatchez, L. 2011. Genetic calibration of species diversity among North America's freshwater fishes. Proc. Natl. Acad. Sci. U.S.A.108, 10602-10607.
Chakrabarty, P. 2006. Systematics and historical biogeography of Greater Antillean Cichlidae. Mol. Phylogenet. Evol. 39, 619-627.
Farias, I.P., Orti, G., Sampaio, I., Schneider, H., Meyer, A. 2001. The cytochrome b gene as a phylogenetic marker: the limits of resolution for analyzing relationships among cichlid fishes. J. Mol. Evol. 53, 89-103.
Genner, M.J., Seehausen, O., Lunt, D.H., Joyce, D.A., Shaw, P.W., Carvalho, G.R., Turner, G.F. 2007. Age of cichlids: new dates for ancient lake fish radiations. Mol. Biol. Evol. 24, 1269-1282.
Kumazawa, Y., Yamaguchi, M., Nishida, M. 1999. Mitochondrial molecular clocks and the origin of euteleostean biodiversity: Familial radiation of perciforms may have predated the Cretaceous/Tertiary boundary. In: Kato, M. (Ed), The biology of biodiversity. Springer-Verlag, Tokyo, pp. 35-52.
López-Fernández, H., Winemiller, K.O., Honeycutt, R.L. 2010. Multilocus phylogeny and rapid radiations in Neotropical cichlid fishes (Perciformes: Cichlidae: Cichlinae). Mol. Phylogenet. Evol. 55, 1070-1086.
Pereira, L.H., Hanner, R., Foresti, F., Oliveira, C. 2013. Can DNA barcoding accurately discriminate megadiverse Neotropical freshwater fish fauna? BMC Genet. 14, 20.
Pereyra, S., Garcia, G. 2008. Patterns of genetic differentiation in the *Gymnogeophagus gymnogenys* species complex, a Neotropical cichlid from South America basins. Environ. Biol. Fishes 83, 245-257.
Ready, J. S., Sampaio, I., Schneider, H., Vinson, C., & Dos Santos, T. 2006. Colour forms of Amazonian cichlid fish represent reproductively isolated species. J. Evol. Biol., 19, 1139-1148.
Rosso, J.J., Mabragana, E., Gonzalez Castro, M., Diaz de Astarloa, J. M. 2012. DNA barcoding Neotropical fishes: recent advances from the Pampa Plain, Argentina. Mol. Ecol. Resour. 12, 999-1011.
Smith, W.L., Chakrabarty, P., Sparks, J.S. 2008. Phylogeny, taxonomy, and evolution of Neotropical cichlids (Teleostei: Cichlidae: Cichlinae). Cladistics 24, 625-641.
Sparks, J.S., Smith, W.L. 2004. Phylogeny and biogeography o cichlid fishes (Teleostei: Perciformes: Cichlidae). Cladistics 20, 501-517.
