# Supplementary material for: Tempo and rates of diversification in the South American cichlid genus Apistogramma (Teleostei: Perciformes: Cichlidae)
Source: PLoS One. 2017 Sep 5;12(9):e0182618. doi: 10.1371/journal.pone.0182618 (PMC5584756; doi:10.1371/journal.pone.0182618)
Supplement: S3 Table — (PDF) [file pone.0182618.s007.pdf]

		1	2	3	4	5	6	7	8	9	10	11	12	13	14	15	16	17	18	19	20	21	22	23	24	25	26	27	28	29	30	31	32
1.	*A. agassizii*	**0.014 (0.002)**[a]	0.012[c]	0.014	0.014	0.015	0.015	0.016	0.012	0.016	0.013	0.017	0.016	0.015	0.015	0.014	0.015	0.015	0.016	0.015	0.016	0.016	0.015	0.015	0.015	0.015	0.014	0.015	0.014	0.015	0.014	0.015	0.016
2.	*A. atahualpa*	0.182[b]	**0.009 (0.002)**	0.010	0.010	0.014	0.013	0.016	0.012	0.014	0.011	0.018	0.012	0.015	0.011	0.011	0.015	0.014	0.015	0.015	0.016	0.015	0.013	0.015	0.015	0.016	0.012	0.014	0.012	0.012	0.012	0.014	0.015
3.	*A. baencshi*	0.194	0.158	**0.008 (0.002)**	0.012	0.013	0.014	0.014	0.013	0.015	0.012	0.016	0.010	0.014	0.009	0.010	0.015	0.015	0.015	0.015	0.015	0.015	0.011	0.015	0.016	0.014	0.013	0.015	0.013	0.014	0.014	0.014	0.014
4.	*A. barlowi*	0.187	0.140	0.142	**0.002 (0.001)**	0.015	0.012	0.011	0.015	0.011	0.004	0.014	0.012	0.013	0.012	0.011	0.012	0.013	0.012	0.015	0.011	0.011	0.013	0.011	0.010	0.012	0.014	0.013	0.015	0.014	0.014	0.011	0.013
5.	*A. bitaeniata*	0.174	0.206	0.191	0.194	**0.004 (0.002)**	0.015	0.015	0.006	0.014	0.016	0.020	0.016	0.017	0.013	0.014	0.015	0.018	0.016	0.015	0.015	0.015	0.015	0.014	0.015	0.017	0.007	0.018	0.004	0.005	0.006	0.015	0.015
6.	*A. cacatuoides*	0.213	0.188	0.208	0.173	0.222	**0.002 (0.001)**	0.014	0.012	0.016	0.012	0.015	0.013	0.017	0.012	0.013	0.016	0.017	0.016	0.014	0.015	0.015	0.014	0.015	0.015	0.016	0.014	0.017	0.015	0.014	0.013	0.015	0.015
7.	*A. cinilabra*	0.218	0.203	0.198	0.183	0.253	0.239	**0.001 (0.000)**	0.015	0.005	0.012	0.009	0.016	0.007	0.014	0.015	0.005	0.009	0.004	0.017	0.005	0.004	0.013	0.005	0.006	0.006	0.015	0.009	0.014	0.014	0.014	0.004	0.015
8.	*A. eremnopyge*	0.155	0.199	0.183	0.190	0.053	0.216	0.243	**0.000 (0.000)**	0.013	0.013	0.018	0.015	0.015	0.012	0.013	0.014	0.016	0.015	0.014	0.014	0.014	0.015	0.014	0.014	0.015	0.004	0.015	0.005	0.005	0.004	0.013	0.014
9.	*A. eunotus* (M1)	0.208	0.191	0.197	0.180	0.241	0.250	0.029	0.235	**0.001 (0.000)**	0.0110	0.009	0.017	0.008	0.014	0.016	0.007	0.008	0.006	0.019	0.004	0.003	0.013	0.003	0.005	0.006	0.014	0.009	0.013	0.013	0.013	0.003	0.015
10.	*A. megastoma*	0.180	0.143	0.147	0.037	0.194	0.173	0.187	0.190	0.183	**0.010 (0.002)**	0.014	0.012	0.013	0.012	0.011	0.013	0.014	0.013	0.015	0.012	0.012	0.012	0.012	0.011	0.012	0.013	0.014	0.015	0.014	0.012	0.012	0.014
11.	*A. moae*	0.227	0.217	0.217	0.198	0.267	0.242	0.073	0.261	0.074	0.203	**0.000 (0.000)**	0.017	0.010	0.016	0.017	0.009	0.011	0.009	0.018	0.010	0.009	0.015	0.009	0.009	0.009	0.019	0.011	0.018	0.019	0.018	0.009	0.016
12.	*A. nijsseni*	0.214	0.167	0.114	0.162	0.222	0.226	0.229	0.214	0.230	0.169	0.253	**0.003 (0.001)**	0.017	0.007	0.009	0.016	0.018	0.017	0.014	0.017	0.017	0.011	0.017	0.016	0.018	0.015	0.018	0.015	0.016	0.015	0.017	0.017
13.	*A. paulmuelleri*	0.231	0.201	0.198	0.187	0.244	0.243	0.093	0.243	0.094	0.191	0.111	0.235	**0.000 (0.000)**	0.014	0.016	0.007	0.009	0.008	0.016	0.008	0.008	0.015	0.008	0.008	0.006	0.016	0.008	0.015	0.015	0.015	0.009	0.015
14.	*A. rositae*	0.182	0.126	0.083	0.124	0.182	0.179	0.203	0.173	0.197	0.129	0.212	0.073	0.194	**0.000 (0.000)**	0.006	0.013	0.016	0.014	0.013	0.014	0.014	0.011	0.014	0.014	0.014	0.012	0.016	0.013	0.013	0.013	0.014	0.014
15.	*A. wolli*	0.188	0.151	0.097	0.147	0.201	0.206	0.192	0.200	0.184	0.149	0.207	0.093	0.200	0.062	-	0.015	0.016	0.016	0.014	0.016	0.016	0.010	0.016	0.016	0.016	0.013	0.017	0.014	0.014	0.013	0.015	0.014
16.	*A.*sp. " Algodon"	0.229	0.206	0.201	0.186	0.256	0.246	0.044	0.246	0.045	0.193	0.080	0.226	0.092	0.197	0.189	**0.002 (0.001)**	0.009	0.007	0.018	0.007	0.007	0.014	0.007	0.005	0.006	0.015	0.009	0.013	0.014	0.014	0.007	0.015
17.	*A.* sp. "Carapintada"	0.213	0.189	0.195	0.177	0.236	0.239	0.087	0.240	0.088	0.183	0.103	0.231	0.066	0.194	0.204	0.094	**0.000 (0.000)**	0.009	0.018	0.009	0.009	0.015	0.009	0.009	0.009	0.017	0.003	0.015	0.015	0.016	0.008	0.014
18.	*A.* sp. "Huanta"	0.213	0.202	0.204	0.183	0.247	0.250	0.031	0.244	0.032	0.186	0.076	0.224	0.088	0.193	0.192	0.054	0.088	**0.000 (0.000)**	0.016	0.006	0.005	0.014	0.006	0.006	0.007	0.016	0.009	0.014	0.014	0.015	0.005	0.015
19.	*A.* sp. "Juruá"	0.200	0.170	0.194	0.171	0.212	0.193	0.236	0.202	0.238	0.178	0.251	0.207	0.236	0.171	0.187	0.238	0.242	0.230	**0.001 (0.000)**	0.018	0.017	0.016	0.018	0.018	0.016	0.015	0.018	0.015	0.015	0.014	0.017	0.014
20.	*A.* sp. "Melgar"	0.216	0.200	0.199	0.187	0.250	0.252	0.036	0.244	0.020	0.191	0.084	0.230	0.096	0.199	0.194	0.050	0.095	0.040	0.241	**0.001 (0.001)**	0.003	0.014	0.004	0.006	0.006	0.015	0.009	0.013	0.014	0.014	0.004	0.015
21.	*A.* sp. "Morado"+*A. eunotus* (M2)	0.212	0.195	0.203	0.183	0.248	0.254	0.029	0.238	0.015	0.191	0.078	0.231	0.092	0.201	0.196	0.047	0.089	0.030	0.236	0.016	**0.002 (0.001)**	0.014	0.003	0.006	0.006	0.014	0.009	0.013	0.014	0.014	0.003	0.015
22.	*A.* sp. "Oregon"	0.190	0.140	0.105	0.123	0.195	0.192	0.183	0.182	0.180	0.133	0.199	0.119	0.199	0.092	0.104	0.183	0.190	0.185	0.180	0.186	0.182	**0.002 (0.001)**	0.014	0.013	0.015	0.016	0.015	0.014	0.015	0.015	0.013	0.014
23.	*A.* sp. "Papagayo"	0.213	0.196	0.198	0.180	0.242	0.258	0.036	0.236	0.021	0.186	0.082	0.226	0.093	0.197	0.196	0.049	0.092	0.038	0.234	0.021	0.015	0.177	**0.004 (0.001)**	0.006	0.006	0.014	0.008	0.013	0.013	0.013	0.004	0.014
24.	*A.* sp. "Pebás"	0.212	0.196	0.200	0.182	0.245	0.252	0.033	0.237	0.034	0.191	0.077	0.237	0.093	0.205	0.195	0.044	0.093	0.040	0.234	0.041	0.035	0.186	0.042	**0.000 (0.000)**	0.007	0.014	0.009	0.014	0.014	0.014	0.006	0.014
25.	*A.* sp. "Peru-regani"	0.230	0.195	0.195	0.181	0.238	0.250	0.061	0.238	0.057	0.188	0.092	0.230	0.047	0.197	0.196	0.075	0.078	0.054	0.227	0.059	0.052	0.186	0.057	0.067	-	0.016	0.009	0.015	0.015	0.015	0.006	0.015
26.	*A.* sp1	0.165	0.206	0.189	0.194	0.057	0.227	0.251	0.018	0.236	0.198	0.262	0.222	0.247	0.183	0.204	0.250	0.246	0.250	0.210	0.242	0.241	0.189	0.239	0.242	0.241	**0.001 (0.001)**	0.017	0.005	0.005	0.004	0.014	0.015
27.	*A.* sp2	0.216	0.191	0.196	0.175	0.241	0.242	0.088	0.244	0.092	0.180	0.102	0.236	0.061	0.193	0.205	0.092	0.011	0.089	0.240	0.097	0.091	0.193	0.092	0.091	0.076	0.252	**0.000 (0.000)**	0.015	0.015	0.015	0.009	0.014
28.	*A.* sp3	0.165	0.207	0.190	0.191	0.020	0.219	0.249	0.050	0.239	0.194	0.265	0.224	0.241	0.181	0.199	0.252	0.238	0.246	0.208	0.247	0.243	0.199	0.236	0.242	0.231	0.055	0.243	**0.001 (0.001)**	0.005	0.005	0.013	0.015
29.	*A.* sp4	0.165	0.204	0.195	0.189	0.035	0.220	0.248	0.033	0.237	0.192	0.265	0.219	0.244	0.181	0.198	0.253	0.242	0.248	0.197	0.248	0.244	0.196	0.242	0.24	0.241	0.039	0.247	0.038	-	0.004	0.013	0.014
30.	*A.* sp5	0.159	0.202	0.187	0.190	0.060	0.223	0.246	0.017	0.232	0.192	0.259	0.213	0.241	0.178	0.196	0.250	0.243	0.244	0.202	0.242	0.235	0.186	0.234	0.238	0.236	0.022	0.247	0.055	0.025	**0.002 (0.001)**	0.013	0.014
31.	*A.* sp6	0.208	0.193	0.198	0.181	0.248	0.246	0.036	0.237	0.020	0.189	0.084	0.229	0.102	0.199	0.190	0.052	0.095	0.038	0.229	0.021	0.015	0.178	0.021	0.042	0.061	0.240	0.099	0.238	0.243	0.234	-	0.014
32.	outgroup	0.279	0.272	0.265	0.266	0.286	0.288	0.266	0.279	0.272	0.270	0.280	0.299	0.278	0.263	0.270	0.278	0.273	0.268	0.281	0.272	0.271	0.266	0.271	0.267	0.275	0.286	0.268	0.285	0.28	0.281	0.270	**0.232 (0.011)**

[a] Genetic distance within species (standard error)
[b] Genetic distance between species (below diagonal)
[c] Standard error (above diagonal)
