# Supplementary material for: Tempo and rates of diversification in the South American cichlid genus Apistogramma (Teleostei: Perciformes: Cichlidae)
Source: PLoS One. 2017 Sep 5;12(9):e0182618. doi: 10.1371/journal.pone.0182618 (PMC5584756; doi:10.1371/journal.pone.0182618)
Supplement: S1 File — (PDF) [file pone.0182618.s008.pdf]

<?xml version="1.0" encoding="UTF-8" standalone="no"?><beast
beautitemplate='StarBeast2' beautistatus='noAutoSetClockRate'
namespace="beast.core:beast.evolution.alignment:beast.evolution.tree.coalescent:beast.core.u
til:beast.evolution.nuc:beast.evolution.operators:beast.evolution.sitemodel:beast.evolution.sub
stitutionmodel:beast.evolution.likelihood" required="starbeast2 v0.13.5" version="2.4">

<data
id="Apisto_Combi_Nuc"
name="alignment">
<sequence id="seq_Aaga_Nuc_Apisto_302902"
taxon="Aaga_Nuc_Apisto_302902" totalcount="4"
value="TGTTGGAGAAGCCAGAAGTGTTGCTTTAGTGGTGGTTGTGTCCCGGGAAG
TGACGTTCATGCCCGCCCCACCTGCCGTCTCCCATCAGCATGTGATGGAGTTTGAT
GTGGAGGAGGACGACTCCTCTCGGTCACCATCTCCTCAGGAGATTCTGCTCGAGG
TGGAGCTGGATGAAAACGAAGTCAAAGAGTTTGAGAGGCAGGTGAAGATCATCA
CCATACCCGAGTACACGGCTGACAACAAGAGCATGATCATATCTCTGGACGTGCT
GCCGAGCATTTATGAGGAAGGTG"/>
<sequence id="seq_Aaga_Nuc_Apisto_302911"
taxon="Aaga_Nuc_Apisto_302911" totalcount="4"
value="TGTTGGAGAAGCCAGAAGTGTTGCTTTAGTGGTGGTTGTGTCCCGGGAAG
TGACGTTCATGCCCGCCCCACCTGCCGTCTCCCATCAGCATGTGATGGAGTTTGAT
GTGGAGGAGGAcGACTCCTCTCGGTCACCATCTCCTCAGGAGATTCTGCTCGAGG
TGGAGCTGGATGAAAACGAAGTCAAAGAGTTTGAGAGGCAGGTGAAGATCATCA
CCATACCCGAGTACACGGCTGACAACAAGAGCATGATCATATCTCTGGACGTGCT
GCCGAGCATTTATGAGGAAGGTG"/>
<sequence id="seq_Aaga_Nuc_Apisto_302918"
taxon="Aaga_Nuc_Apisto_302918" totalcount="4"
value="TGTTGGAGAAGCCAGAAGTGTTGCTTTAGTGGTGGTTGTgTCCCGGGAAGT
GACGTTCATGCCCGCCCCACCTGCCGTCTCCCATCAGCATGTGATGGAGTTTGAT
GTGGAGGAGGAcGACTCCTCTCGGTCACCATCTCCTCAGGAGATTCTGCTcGAGGT
GGAGCTGGATGAAAACGAAGTCAAAGAGTTTGAGAgGCAGGTGAAGATCATCAC
CATACCCGAGTACACGgCTGACAACAaGAGCATGATCATATCTCTGGACGTGCTG
CCGAGCATTTATGAGGAAGGTG"/>
<sequence id="seq_Aaga_Nuc_Apisto_302920"
taxon="Aaga_Nuc_Apisto_302920" totalcount="4"
value="TGTTGGAGAAGCCAGAAGTGTTGCTTTAGTGGTGGTTGTGTCCCGGGAAG
TGACGTTCATGCCCGCCCCACCTGCCGTCTCCCATCAGCATGTGATGGAGTTTGAT
GTGGAGGAGGACGACTCCTCTCGGTCACCATCTCCTCAGGAGATTCTGCTCGAGG
TGGAGCTGGATGAAAACGAAGTCAAAGAGTTTGAGAGGCAGGTGAAGATCATCA
CCATACCCGAGTACACGGCTGACAACAAGAGCATGATCATATCTCTGGACGTGCT
GCCGAGCATTTATGAGGAAGGTG"/>
<sequence id="seq_Aaga_Nuc_Apisto_302958"
taxon="Aaga_Nuc_Apisto_302958" totalcount="4"
value="TGTTGGAGAAGCCAGAAGTGTTGCTTTAGTGGTGGTTGTGTCCCGGGAAG
TGACGTTCATGCCCGCCCCACCTGCCGTCTCCCATCAGCATGTGATGGAGTTTGAT
GTGGAGGAGGAcGACTCCTCTCGGTCACCATCTCCTCAGGAGATTCTGCTCGAGG
TGGAGCTGGATGAAAACGAAGTCAAAGAGTTTGAGAgGCAGGTGAAGATCATCA
CCATACCCGAGTACACGGCTGACAACAAGAGCATGATCATATCTCTGGACGTGCT
GCCGAGCATTTATGAGGAAGGTG"/>

<sequence id="seq_Aaga_Nuc_Apisto_302959"
taxon="Aaga_Nuc_Apisto_302959" totalcount="4"
value="TGTTGGAGAAGCCAGAAGTGTTGCTTTAGTGGTGGTTGTATCaCGGGAAGT
GACGTTCATGCCCGCCCCACCTGCCGTCTCCCATCAGCATGTGATGGAGTTTGAT
GTGGAGGAGGATGACTCCTCTCGGTCACCATCTCCTCAGGAGATTCTGCTCGAGG
TGGAGCTGGATGAAAACGAAGTCAAAGAGTTTGAGAAGCAGGTGAAGATCATCa
CCATACCCGAGTACACGGCTGACAACAAGAGCATGATCATATCTCTGGACGTGCT
GCCGAGCATTTATGAGGAAGGTG"/>
<sequence id="seq_Aaga_Nuc_Apisto_303077"
taxon="Aaga_Nuc_Apisto_303077" totalcount="4"
value="TGTTGGAGAAGCCAGAAGTGTTGCTTTAGTGGTGGTTGTGTCCCGGGAAG
TGACGTTCATGCCCGCCCCACCTGCCGTCTCCCATCAGCATGTGATGGAGtttGATG
TGGAGGAGGACGACTCCTCTCGGTCACCATCTCCTCAGGAGATTCTGCTCGAGGT
GGAGCTGGATGAAaaCGAAGTCAAAGAGTTTGAGAGGCAGGTGAAGATCATCaCC
ATACCCGAGTACACGGCTGACAACAAGAGCATGATCATATCTCTGGACGTGCTGC
CGAGCATTTATGAGGAAGGTG"/>
<sequence id="seq_Aaga_Nuc_Apisto_303078"
taxon="Aaga_Nuc_Apisto_303078" totalcount="4"
value="TGTTGGAGAAGCCAGAAGTGTTGCTTTAGTGGTGGTTGTATCCCGGGAAG
TGACGTTCATGCCCGCCCCACCTGCCGTCTCCCATCAGCATGTGATGGAGTTTGAT
GTGGAGGAGGATGACTCCTCTCGGTCACCATCTCCTCAGGAGATTCTGCTCGAGG
TGGAGCTGGATGAAAACGAAGTCAAAGAGTTTGAGAAGCAGGTGAAGATCATCA
CCATACCCGAGTACACGGCTGACAACAAGAGCATGATCATATCTCTGGACGTGCT
GCCGAGCATTTATGAGGAAGGTG"/>
<sequence id="seq_Aaga_Nuc_Apisto_300492"
taxon="Aaga_Nuc_Apisto_300492" totalcount="4"
value="TGTTGGAGAAGCCAGAAGTGTTGCTTTAGTGGTGGTTGTGTCCCGGGAAG
TGACGTTCATGCCCGCCCCACCTGCCGTCTCCCATCAGCATGTGATGGAGTTTGAT
GTGGAGGAGGACgACTCCTCTCGGTCACCATCTCCTCAGGAGATTCTGCTCGAGG
TGGAGCTGGATGAAAACGAAGTCAAAGAGTTTGAGAGGCAGGTGAAGATCATCA
CCATACCcGAGTACACGGCTGACAACAAGAGCATGATCaTATCTCTGGACGTGCT
GCCGAGCATTTATGAGGAAGGTG"/>
<sequence id="seq_Aaga_Nuc_Apisto_300494"
taxon="Aaga_Nuc_Apisto_300494" totalcount="4"
value="TGTTGGAGAAGCCAGAAGTGTTGCTTTAGTGGTGGTTGTGTCCCGGGAAG
TGACGTTCATGCCCGCCCCACCTGCCGTCTCCCATCAGCATGTGATGGAGTTTGAT
GTGGAGGAGGACGACTCCTCTCGGTCACCATCTCCTCAGGAGATTCTGCTCGAGG
TGGAGCTGGATGAAAACGAAGTCAAAGAGTTTGAGAGGCAGGTGAAGATCATCA
CCATACCCGAGTACACGGCTGACAACAAGAGCATGATCATATCTCTGGACGTGCT
GCCGAGCATTTATGAGGAAGGTG"/>
<sequence id="seq_Aaga_Nuc_Apisto_302185"
taxon="Aaga_Nuc_Apisto_302185" totalcount="4"
value="TGTTGGAGAAGCCAGAAGTGTTGCTTTAGTGGTGGTTGTgTCcCGGGAAGT
GACGTTCATGCCCGCCCCACCTGCCGTCTCCCATCAGCATGTGATGGAGTTTGAT
GTGGAGGAGGAcGACTCCTCTCGGTCACCATCTCCTCAGGAGATTCTGCTCGAGG
TGGAGCTGGATGAAAACGAAGTCAAAGAGTTTGAGAgGCAGGTGAAGATCATCA
CCATACCCGAGTACACGGCTGACAACAAGAGCATGATCATATCTCTGGACGTGCT
GCCGAGCATTTATGAGGAAGGTG"/>
<sequence id="seq_Aaga_Nuc_Apisto_302186"
taxon="Aaga_Nuc_Apisto_302186" totalcount="4"

value="TGTTGGAGAAGCCAGAAGTGTTGCTTTAGTGGTGGTTGTGTCCCGGGAAG
TGACGTTCATGCCCGCCCCACCTGCCGTCTCCCATCAGCATGTGATGGAGTTTGAT
GTGGAGGAGGACGACTCCTCTCGGTCACCATCTCCTCAGGAGATTCTGCTCGAGG
TGGAGCTGGATGAAAACGAAGTCAAAGAGTTTGAGAGGCAGGTGAAGATCATCA
CCATACCCGAGTACACGGCTGACAACAAGAGCATGATCATATCTCTGGACGTGCT
GCCGAGCATTTATGAGGAAGGTG"/>
<sequence id="seq_Aalg_Nuc_Apisto_300787"
taxon="Aalg_Nuc_Apisto_300787" totalcount="4"
value="TGTTGGAGAAGCCAGAAGTGTTGCTTTAGTGGTaGTTGTATCACGGGAAGT
GACgTTCATGCCCGCCCCACCTGCCGTCTCCCAtCAGCATGTGATGGAGTTtGATGT
GGAGGAaGATGACTCtTCTCGGTCACCgTCTCCTCAGGAGATTCTGCTGGAaGTGG
AGCTGGATGAAAACGAgGTCAAAGAGTTTGAGAAGCAGGTGAAGATCATCACCA
TACCCGAGTACACagCTGACAACAAGAGCATGATcATATCTCTGGAtGTGCTgCCGA
GCATTTATGAGGAAGGTG"/>
<sequence id="seq_Aalg_Nuc_Apisto_300788"
taxon="Aalg_Nuc_Apisto_300788" totalcount="4"
value="TGTTGGAGAAGCCAGAAGTGTTGCTTTAGTGGTAGTTGTATCACGGGAAG
TGACATTCATGCCCGCCCCACCTGCCGTCTCCCACCAGCATGTGATGGAGTTTGA
TGTGGAGGAAGATGACTCTTCTCGGTCACCGTCTCCTCAGGAGATTCTGCTGGAA
GTGGAGCTGGATGAAAACGAgGTCAAAGAGTTTGAGAAGCAGGTGAAGATCATC
ACCATACCCGAGTACACaGCTGACAACAAGAGCATGATCATATCTCTGGATGTGC
TaCCGAGCATTTATGAGGAAGGTG"/>
<sequence id="seq_Aata_Nuc_Apisto_300411"
taxon="Aata_Nuc_Apisto_300411" totalcount="4"
value="TGTTGGAGAAGCCAGAAGTGTTGCTTTAGTGGTGGTTGTGTCCCGGGAAG
TGACGTTCATGCCAGCCCCACCTGCCGTCTCCCATCAGCATGTGATGGAGTTTGA
TGTGGAGGAGGATGACTCCTCTCGGTCACCATCTCCTCAGGAGATTCTGCTCGAG
GTGGAGCTGGATGAAAACGAAGTCAAAGAGTTTGAGAAGCAGGTGAAGATCATC
ACCATACCCGAGTACACGGCTGACAACAAGAGCATGATCATATCTCTGGACGTGC
TGCCGAGCATTTATGAGGAAGGTG"/>
<sequence id="seq_Aata_Nuc_Apisto_301983"
taxon="Aata_Nuc_Apisto_301983" totalcount="4"
value="TGTTGGAGAAGCCAGAAGTGTTGCTTTAGTGGTGGTTGTATCaCGGGAAGT
GACGTTCATGCCCGCCCCACCTGCCGTCTCCCATCAGCATGTGATGGAGTTTGAT
GTGGAGGAGGATGACTCCTCTCGGTCACCATCTCCTCAGGAGATTCTGCTCGAGG
TGGAGCTGGATGAAAACGAAGTCAAAGAGTTTGAGAAGCAGGTGAAGATCATCA
CCATACCCGAGTACACGGCTGACAACAAGAGCATGATCATATCTCTGGACGTGCT
GCCGAGCATTTATGAGGAAGGTG"/>
<sequence id="seq_Aata_Nuc_Apisto_301990"
taxon="Aata_Nuc_Apisto_301990" totalcount="4"
value="TGTTGGAGAAGCCAGAAGTGTTGCTTTAGTGGTGGTTGTGTCCCGGGAAG
TGACGTTCATGCCAGCCCCACCTGCCGTCTCCCATCAGCATGTGATGGAGTTTGA
TGTGGAGGAGGATGACTCCTCTCGGTCACCATCTCCTCAGGAGATTCTGCTCGAG
GTGGAGCTGGATGAAAACGAAGTCAAAGAGTTTGAGAAGCAGGTGAAGATCATC
ACCATACCCGAGTACACGGCTGACAACAAGAGCATGATCATATCTCTGGACGTGC
TGCCGAGCATTTATGAGGAAGGTG"/>
<sequence id="seq_Aata_Nuc_Apisto_301991"
taxon="Aata_Nuc_Apisto_301991" totalcount="4"
value="TGTTGGAGAAGCCAGAAGTGTTGCTTTAGTGGTGGTTGTGTCCCGGGAAG
TGACGTTCATGCCAGCCCCACCTGCCGTCTCCCATCAGCATGTGATGGAGTTTGA

TGTGGAGGAGGATGACTCCTCTCGGTCACCATCTCCTCAGGAGATTCTGCTCGAG
GTGGAGCTGGATGAAAACGAAGTCAAAGAGTTTGAGAAGCAGGTGAAGATCATC
ACCATACCCGAGTACACGGCTGACAACAAGAGCATGATCATATCTCTGGACGTGC
TGCCGAGCATTTATGAGGAAGGTG"/>
<sequence id="seq_Aata_Nuc_Apisto_302106"
taxon="Aata_Nuc_Apisto_302106" totalcount="4"
value="TGTTGGAGAAGCCAGAAGTGTTGCTTTAGTGGTGGTTGTATCACGGGAAG
TGACGTTCATGCCCGCCCCACCTGCCGTCTCCCATCAGCATGTGATGGAGTTTGAT
GTGGAGGAGGATGACTCCTCTCGGTCACCATCTCCTCAGGAGATTCTGCTCGAGG
TGGAGCTGGATGAAAACGAAGTCAAAGAGTTTGAGAAGCAGGTGAAGATCATCA
CCATACCTGAGTACACGGCTGACAACAAGAGCATGATCATATCTCTGGACGTGCT
GCCGAGCATTTATGAGGAAGGTG"/>
<sequence id="seq_Aata_Nuc_Apisto_302107"
taxon="Aata_Nuc_Apisto_302107" totalcount="4"
value="TGTTGGAGAAGCCAGAAGTGTTGCTTTAGTGGTGGTTGTGTCCCGGGAAG
TGACGTTCATGCCAGCCCCACCTGCCGTCTCCCATCAGCATGTGATGGAGTTTGA
TGTGGAGGAGGATGACTCCTCTCGGTCACCATCTCCTCAGGAGATTCTGCTCGAG
GTGGAGCTGGATGAAAACGAAGTCAAAGAGTTTGAGAAGCAGGTGAAGATCATC
ACCATACCCGAGTACACGGCTGACAACAAGAGCATGATCATATCTCTGGACGTGC
TGCCGAGCATTTATGAGGAAGGTG"/>
<sequence id="seq_Abae_Nuc_Apisto_301654"
taxon="Abae_Nuc_Apisto_301654" totalcount="4"
value="TGTTGGAGAAGCCAGAAGTGTTGCTTTAGTGGTGGTTGTATCACGGGAAG
TGACGTTCATGCCCGCCCCACCTGCCGTCTCCCATCAGCATGTGATGGAGTTTGAT
GTGGAGGAGGATGACTCCTCTCGGTCACCATCTCCTCAGGAGATTCTGCTCGAGG
TGGAGCTGGATGAAAACGAAGTCAAAGAGTTTGAGAAGCAGGTGAAGATCATCA
CCATACCCGAGTACACGGCTGACAACAAGAGCATGATCATATCTCTGGACGTGCT
GCCGAGCATTTATGAGGAAGGTG"/>
<sequence id="seq_Abae_Nuc_Apisto_301676"
taxon="Abae_Nuc_Apisto_301676" totalcount="4"
value="TGTTGGAGAAGCCAGAAGTGTTGCTTTAGTGGTGGTTGTATCACGGGAAG
TGACGTTCATGCCCGCCCCACCTGCCGTCTCCCATCAGCaTGTGATGGAGTTTGAT
GTGGAGGAGGATGACTCCTCTCGGTCACCATCTCCTCaGGAGATTCTGCTCGAGG
TGGAGCTGGATGAAAACGAAGTCAAAGAGTTTGAGAAGCaGGTGAAGATCATCA
CCATACCCGAGTACACGGCTGACAACAAGAGCATGATCATATCTCTGGACGTGCT
GCCGAGCATTTAtGAGGAAGGTG"/>
<sequence id="seq_Abae_Nuc_Apisto_301677"
taxon="Abae_Nuc_Apisto_301677" totalcount="4"
value="TGTTGGAGAAGCCAGAAGTGTTGCTTTAGTGGTGGTTGTATCACGGGAAG
TGACGTTCATGCCCGCCCCACCTGCCGTCTCCCATCAGCATGTGATGGAGTTTGAT
GTGGAGGAGGATGACTCCTCTCGGTCACCaTCTCCTCAGGAGATTCTGCTCGAGG
TGGAGCTGGATGAAAACGAAGTcaAAGAGTTTGAGAAGCAGGTGAAGATCATCAC
CATACCCGAGTACACGGCTGACAACAAGAGCATGATCATATCTCTGGACGTGCTG
CCGAGCATTTATGAGGAAGGTG"/>
<sequence id="seq_Abae_Nuc_Apisto_301678"
taxon="Abae_Nuc_Apisto_301678" totalcount="4"
value="TGTTGGAGAAGCCAGAAGTGTTGCTTTAGTGGTGGTTGTATCACGGGAAG
TGACGTTCATGCCCGCCCCACCTGCCGTCTCCCATCAGCATGTGATGGAGTTTGAT
GTGGAGGAGGATGACTCCTCTCGGTCACCATCTCCTCAGGAGATTCTGCTCGAGG
TGGAGCTGGATGAAAACGAAGTCAAAGAGTTTGAGAAGCAGGTGAAGATCATCA

CCATACCCGAGTACACGGCTGACAACAAGAGCATGATCATATCTCTGGACGTGCT
GCCGAGCATTTACGAGGAAGGTG"/>
<sequence id="seq_Abae_Nuc_Apisto_301679"
taxon="Abae_Nuc_Apisto_301679" totalcount="4"
value="TGTTGGAGAAGCCAGAAGTGTTGCTTTAGTGGTGGTTGTATCACGGGAAG
TGACGTTCATGCCCGCCCCACCTGCCGTCTCCCATCAGCATGTGATGGAGTTTGAT
GTGGAGGAGGATGACTCCTCTCGGTCACCATCTCCTCAGGAGATTCTGCTCGAGG
TGGAGCTGGATGAAAACGAAGTCAAAGAGTTTGAGAAGCAGGTGAAGATCATCA
CCATACCCGAGTACACGGCTGACAACAAGAGCATGATCATATCTCTGGACGTGCT
GCCGAGCATTTATGAGGAAGGTG"/>
<sequence id="seq_Abar_Nuc_Apisto_302256"
taxon="Abar_Nuc_Apisto_302256" totalcount="4"
value="TGTTGGAGAAGCCAGAAGTGTTGCTTTAGTGGTGGTTGTATCACGGGAAG
TGACGTTCATGCCCGCCCCACCTGCCGTCTCCCATCAGCATGTGATGGAGTTTGAT
GTGGAGGAGGATGACTCCTCTCGGTCACCATCTCCTCAGGAGATTCTGCTCGAGG
TGGAGCTGGATGAAAACGAAGTCAAAGAGTTTGAGAAGCAGGTGAAGATCATCA
CCATACCCGAGTACACGGCTGACAACAAGAGCATGATCATATCTCTGGACGTGCT
GCCGAGCATTTATGAGGAAGGTG"/>
<sequence id="seq_Abar_Nuc_Apisto_302257"
taxon="Abar_Nuc_Apisto_302257" totalcount="4"
value="TGTTGGAGAAGCCAGAAGTGTTGCTTTAGTGGTGGTTGTATCACGGGAAG
TGACGTTCATGCCCGCCCCACCTGCCGTCTCCCATCAGCATGTGATGGAGTTTGAT
GTGGAGGAGGATGACTCCTCTCGGTCACCATCTCCTCAGGAGATTCTGCTCGAGG
TGGAGCTGGATGAAAACGAAGTCAAAGAGTTTGAGAAGCAGGTGAAGATCATCA
CCATACCCGAGTACACGGCTGACAACAAGAGCATGATCATATCTCTGGACGTGCT
GCCGAGCATTTATGAGGAAGGTG"/>
<sequence id="seq_Abar_Nuc_Apisto_302258"
taxon="Abar_Nuc_Apisto_302258" totalcount="4"
value="TGTTGGAGAAGCCAGAAGTGTTGCTTTAGTGGTGGTTGTATCACGGGAAG
TGACGTTCATGCCCGCCCCACCTGCCGTCTCCCATCAGCATGTGATGGAGTTTGAT
GTGGAGGAGGATGACTCCTCTCGGTCACCATCTCCTCAGGAGATTcTGCTCGAGG
TGGAGCTGGATGAAAACGAAGTCAAAGAGTTTGAGAAGCAGGTGAAGATCATCA
CCATACCCGAGTACACGGCTGACAACAAGAGCATGATCATATCTCTGGACGTGCT
GCCGAGCATTTATGAGGAAGGTG"/>
<sequence id="seq_Abar_Nuc_Apisto_302259"
taxon="Abar_Nuc_Apisto_302259" totalcount="4"
value="TGTTGGAGAAGCCAGAAGTGTTGCTTTAGTGGTGGTTGTATCACGGGAAG
TGACGTTCATGCCCGCCCCACCTGCCGTCTCCCATCAGCATGTGATGGAGTTTGAT
GTGGAGGAGGATGACTCCTCTCGGTCACCATCTCCTCAGGAGATTcTGCTCGAGG
TGGAGCTGGATGAAAACGAAGTCAAAGAGTTTGAGAAGCAGGTGAAGATCATCA
CCATACCCGAGTACACGGCTGACAACAAGAGCATGATCATATCTCTGGACGTGCT
GCCGAGCATTTATGAGGAAGGTG"/>
<sequence id="seq_Abar_Nuc_Apisto_302896"
taxon="Abar_Nuc_Apisto_302896" totalcount="4"
value="TGTTGGAGAAGCCAGAAGTGTTGCTTTAGTGGTGGTTGTATCACGGGAAG
TGACGTTCATGCCCGCCCCACCTGCCGTCTCCCATCAGCATGTGATGGAGTTTGAT
GTAGAGGAGGATGACTCCTCTCGGTCACCATCTCCTCAGGAGATTCTGCTCGAGG
TGGAGCTGGATGAAAACGAAGTCAAAGAGTTTGAGAAGCAGGTGAAGATCATCA
CCATACCCGAGTACACGGCTGACAACAAGAGCATGATCATATCTCTGGACGTGCT
GCCGAGCATTTATGAGGAAGGTG"/>

<sequence id="seq_Abar_Nuc_Apisto_302897"
taxon="Abar_Nuc_Apisto_302897" totalcount="4"
value="TGTTGGAGAAGCCAGAAGTGTTGCTTTAGTGGTGGTTGTATCACGGGAAG
TGACGTTCATGCCCGCCCCACCTGCCGTCTCCCATCAGCATGTGATGGAGTTTGAT
GTgGAGGAGGATGACTCCTCTCGGTCACCATCTCCTCAGGAGATTCTGCTCGAGG
TGGAGCTGGATGAAAACGAAGTCAAAGAGTTTGAGAAGCAGGTGAAGATCATCA
CCATACCCGAGTACACGGCTGACAACAAGAGCATGATCATATCTCTGGACGTGCT
GCCGAGCATTTATGAGGAAGGTG"/>
<sequence id="seq_Abar_Nuc_Apisto_302899"
taxon="Abar_Nuc_Apisto_302899" totalcount="4"
value="TGTTGGAGAAGCCAGAAGTGTTGCTTTAGTGGTGGTTGTATCACGGGAAG
TGACGTTCATGCCCGCCCCACCTGCCGTCTCCCATCAGCATGTGATGGAGTTTGAT
GTGGAGGAGGATGACTCCTCTCGGTCACCATCTCCTCAGGAGATTCTGCTCGAGG
TGGAGCTGGATGAAAACGAAGTCAAAGAGTTTGAGAAGCAGGTGAAGATCATCA
CCATACCCGAGTACACGGCTGACAACAAGAGCATGATCATATCTCTGGACGTGCT
GCCGAGCATTTATGAGGAAGGTG"/>
<sequence id="seq_Abar_Nuc_Apisto_302900"
taxon="Abar_Nuc_Apisto_302900" totalcount="4"
value="TGTTGGAGAAGCCAGAAGTGTTGCTTTAGTGGTGGTTGTATCACGGGAAG
TGACGTTCATGCCCGCCCCACCTGCCGTCTCCCATCAGCATGTGATGGAGTTTGAT
GTgGAGGAGGATGACTCCTCTCGGTCACCATCTCCTCAGGAGATTCTGCTCGAGG
TGGAGCTGGATGAAAACGAAGTCAAAGAGTTTGAGAAGCAGGTGAAGATCATCA
CCATACCCGAGTACACGGCTGACAACAAGAGCATGATCATATCTCTGGACGTGCT
GCCGAGCATTTATGAGGAAGGTG"/>
<sequence id="seq_Abar_Nuc_Apisto_300542"
taxon="Abar_Nuc_Apisto_300542" totalcount="4"
value="TGTTGGAGAAGCCAGAAGTGTTGCTTTAGTGGTGGTTGTATCACGGGAAG
TGACGTTCATGCCCGCCCCACCTGCCGTCTCCCATCAGCATGTGATGGAGTTTGAT
GTGGAGGAGGATGACTCCTCTCGGtCaCCaTCTCCTCAGGAGATTCTGCTCGAGGT
GGAGCTGGATgAAAACGAAGTcaAAGAGTTTgAGAAGCAGGTgAAGATcaTCACCA
TACCCGAgTAcACgGCTgACAACAAGAGCATGATCATATCTCTGGACGTGCTGCCG
AGCATTTATGAGGAAGGTG"/>
<sequence id="seq_Abar_Nuc_Apisto_300549"
taxon="Abar_Nuc_Apisto_300549" totalcount="4"
value="TGTTGGAGAAGCCAGAAGTGTTGCTTTAGTGGTGGTTGTATCACGGGAAG
TGACGTTCATGCCCGCCCCACCTGCCGTCTCCCATCAGCATGTGATGGAGTTTGAT
GTAGAGGAGGATGACTCCTCTCGGTCACCaTCTCCTCaGGAGATTCTGCTCGAGGT
GGAGCTGGATGAAAACGAAGTCAAAGAGTTTGAGAaGCAGGTGAAGATCATCAC
CATACCCGAgTACACGGCTgACAACAAGAGCATGATCATATCTCTGGACGTGCTG
CCGAGCATTTATGAGGAAGGTG"/>
<sequence id="seq_Abar_Nuc_Apisto_300550"
taxon="Abar_Nuc_Apisto_300550" totalcount="4"
value="TGTTGGAGAAGCCAGAAGTGTTGCTTTAGTGGTGGTTGTATCACGGGAAG
TGACGTTCATGCCCGCCCCACCTGCCGTCTCCCATCAGCATGTGATGGAGTTTGAT
GTgGAGGAGGATGACTCCTCTCGGTCACCaTCTCCTCAGGAGATTCTGCTCGAGGT
GGAGCTGGATGAAAACGAAGTCAAAGAGTTtGAGAAGCAGGtGAAGATCaTCACC
ATACCCGAgTAcACgGCTgACAAcAAGAgCATGATCATATCTCTGGACGTGCTGCCG
AGCATTTATGAGGAAGGTG"/>
<sequence id="seq_Abar_Nuc_Apisto_300554"
taxon="Abar_Nuc_Apisto_300554" totalcount="4"

value="TGTTGGAGAAGCCAGAAGTGTTGCTTTAGTGGTGGTTGTATCACGGGAAG
TGACGTTCATGCCCGCCCCACCTGCCGTCTCCCATCAGCATGTGATGGAGTTTGAT
GTGGAGGAGGATGACTCCTCTCGGTCACCATCTCCTCAGGAGATTCTGCTCGAGG
TGGAGCTGGATGAAAACGAAGTCAAAGAGTTTGAGAAGCAGGTGAAGATCATCA
CCATACCCGAGTACACGGCTGACAACAAGAGCATGATCATATCTCTGGACGTGCT
GCCGAGCATTTATGAGGAAGGTG"/>
<sequence id="seq_Abar_Nuc_Apisto_300557"
taxon="Abar_Nuc_Apisto_300557" totalcount="4"
value="TGTTGGAGAAGCCAGAAGTGTTGCTTTAGTGGTGGTTGTATCACGGGAAG
TGACGTTCATGCCCGCCCCACCTGCCGTCTCCCATCAGCATGTGATGGAGTTTGAT
GTGGAGGAGGATGACTCCTCTCGGTCACCATCTCCTCAGGAGATTCTGCTCGAGG
TGGAGCTGGATGAAAACGAAGTCAAAGAGTTTGAGAAGCAGGTGAAGATCATCA
CCATACCCGAGTACACGGCTGACAACAAGAGCATGATCATATCTCTGGACGTGCT
GCCGAGCATTTATGAGGAAGGTG"/>
<sequence id="seq_Abit1_Nuc_Apisto_300438"
taxon="Abit1_Nuc_Apisto_300438" totalcount="4"
value="TGTTGGAGAAGCCAGAAGTGTTGCTTTAGTGGTGGTTGTGTCCCGGGAAG
TGACGTTCATGCCCGCCCCACCTGCCGTCTCCCATCAGCATGTGATGGAGTTTGAT
GTGGAGGAGGATGACTCCTCTCGGTCACCATCTCCTCAGGAGATTCTGCTCGAGG
TGGAGCTGGATGAAAACGAAGTCAAAGAGTTTGAGAGGCAGGTGAAGATCATCA
CCATACCCGAGTACACAGCTGACAACAAGAGCATGATCATATCTCTgGACGTGCT
GCCGAGCATTTATGAGGAAGGTG"/>
<sequence id="seq_Abit1_Nuc_Apisto_300440"
taxon="Abit1_Nuc_Apisto_300440" totalcount="4"
value="TGTTGGAGAAGCCAGAAGTGTTGCTTTAGTGGTGGTTGTGTCCCGGGAAG
TGACGTTCATGCCCGCCCCACCTGCCGTCTCCCATCAGCATGTGATGGAGTTTGAT
GTGGAGGAGGATGACTCCTCTCGGTCACCATCTCCTCAGGAGATTCTGCTCGAGG
TGGAGCTGGATGAAAACGAAGTCAAAGAGTTTGAGAGGCAGGTGAAGATCATCA
CCATACCCGAGTACACtGCTGACAACAAGAGCATGATCATATCTCTGGACGTGCT
GCCGAGCATTTATGAGGAAGGTG"/>
<sequence id="seq_Abit3_Nuc_Apisto_303098"
taxon="Abit3_Nuc_Apisto_303098" totalcount="4"
value="TGTTGGAGAAGCCAGAAGTGTTGCTTTAGTGGTGGTTGTGTCCCGGGAAG
TGACGTTCATGCCCGCCCCACCTGCCGTCTCCCATCAGCATTTGATGGAGTTTGAT
GTGGAGGAGGATGACTCCTCTCGGTCACCATCTCCTCAGGAGATTCTGCTCGAGG
TGGAGCTGGATGAAAACGAAGTCAAAGAGTTTGAGAGGCAGGTGAAGATCATCA
CCATACCCGAGTACACTGCTGACAACAAGAGCATGATCATATCTCTGGACGTGCT
GCCGAGCATTTATGAGGAAGGTG"/>
<sequence id="seq_Abit4_Nuc_Apisto_302612"
taxon="Abit4_Nuc_Apisto_302612" totalcount="4"
value="TGTTGGAGAAGCCAGAAGTGTTGCTTTAGTGGTGGTTGTGTCCCGGGAAG
TGACGTTCATGCCCGCCCCACCTGCCGTCTCCCATCAGCATGTGATGGAGTTTGAT
GTGGAGGAGGATGACTCCTCTCGGTCACCATCTCCTCAGGAGATTcTGCTcGAGGT
GGAGCTGGATGAAAACGAAGTCAAAGAGTTTGAGAGGCAGGTGAAGATCATCAC
CATACCcGAGTACACtGCTGACAACAAGAGCATGATCATATCTCTGGACGTgCTGC
CGAGCATTTATGAGGAAGGTG"/>
<sequence id="seq_Abit4_Nuc_Apisto_302614"
taxon="Abit4_Nuc_Apisto_302614" totalcount="4"
value="TGTTGGAGAAGCCAGAAGTGTTGCTTTAGTGGTGGTTGTGTCCCgGGAAGT
GACGTTCATGCCCGCCCCACCTGCCGTCTCCCATCAGCATGTGATGGAGTTTGAT

GTGGAGGAGGATGACTCCTCTCGGTCACCATCTCCTCAGGAGATTCTGCTcGAGG
TGGAGCTGGATGAAAACGAAGTCAAAGAGTTTGAGAGGCAGGTGAAGATCATCA
CCATAcCCGaGTACACTGCTGACAACAAGAGCATGATCATATCTCTGGACGTGCTG
CCGAGCATTTATGAGGAAGGTG"/>
<sequence id="seq_Abit5_Nuc_Apisto_302926"
taxon="Abit5_Nuc_Apisto_302926" totalcount="4"
value="TGTTGGAGAAGCCAGAAGTGTTGCTTTAGTGGTGGTTGTATCACGGGAAG
TGACGTTCATGCCCGCCCCACCTGCCGTcTCCCATCAGCATGTGATGGAGTTTGAT
GTGGAGGAGGATGACTCCTCTCGGTCACCATCTCCTCAGGAGATTCTGCTCGAGG
TGGAGCTGGATGAAAACGAAGTCAAAGAGTTTGAGAAGCAGGTGAAGATCATCa
CCATACCCGAGTACACGGCTGACAACAAGAGCATGATCATATCTCTGGACGTGCT
GCCGAGCATTTATGAGGAAGGTG"/>
<sequence id="seq_Abit5_Nuc_Apisto_302951"
taxon="Abit5_Nuc_Apisto_302951" totalcount="4"
value="TGTTGGAGAAGCCAGAAGTGTTGCTTTAGTGGTGGTTGTATCACGGGAAG
TGACGTTCATGCCCGCCCCACCTGCCGTcTCCCATCAGCATGTGATGGAGTTTGAT
GTGGAGGAGGATGACTCCTCTCGGTCACCATCTCCTCAGGAGATTCTGCTCGAGG
TGGAGCTGGATGAAAACGAAGTCAAAGAgTTTGAGAAGCAGGTGAAGATCATCaC
CATACCCGAGTACACGGCTGACAACAAGAGCATGATCATATCTCTGGACGTGCTG
CCGAGCATTTATGAGGAAGGTG"/>
<sequence id="seq_Abit5_Nuc_Apisto_302952"
taxon="Abit5_Nuc_Apisto_302952" totalcount="4"
value="TGTTGGAGAAGCCAGAAGTGTTGCTTTAGTGGTGGTTGTATCACGGGAAG
TGACGTTCATGCCCGCCCCACCTGCCGTCTCCCATCAGCATGTGATGGAGTTTGAT
GTGGAGGAGGATGACTCCTCTCGGTCACCATCTCCTCAGGAGATTcTGCTCGAGG
TGGAGCTGGATGAAAACGAAGTCAAAGAGTTTGAGAAGCAGGTGAAGATCATCa
CCATACCCGAGTACaCGGCTGACAACAAGAGCATGATCATATCTCTGGACGTGCT
GCCGAGCATTTATGAGGAAGGTG"/>
<sequence id="seq_Abit_Nuc_Apisto_302613"
taxon="Abit_Nuc_Apisto_302613" totalcount="4"
value="TGTTGGAGAAGCCAGAAGTGTTGCTTTAGTGGTGGTTGTGTCCCGGGAAG
TGACGTTCATGCCCGCCCCACCTGCCGTCTCCCATCAGCATGTGATGGAGTTTGAT
GTGGAGGAGGATGACTCCTCTCGGTCACCATCTCCTCAGGAGATTcTGCTcGAGGT
GGAGCTGGATGAAAACGAAGTCAAAGAGTTTGAGAGGCAGGTGAAGATCATCAC
CATACCcGAGTACACtGCTGACAACAAGAGCATGATCATATCTCTgGACGTgcTGCC
GAGCATTTATGAGGAAGGTG"/>
<sequence id="seq_Abit_Nuc_Apisto_302615"
taxon="Abit_Nuc_Apisto_302615" totalcount="4"
value="TGTTGGAGAAGCCAGAAGTGTTGCTTTAGTGGTGGTTGTGTCCCGGGAAG
TGACGTTCATGCCCGCCCCACCTGCCGTCTCCCATCAGCATGTGATGGAGTTTGAT
GTGGAGGAGGATGACTCCTCTCGGTCACCATCTCCTCAGGAGATTCTGCTcGAGG
TGGAGCTGGATGAAAACGAAGTCAAAGAGTTTGAGAGGCAGGTGAAGATcATCA
CCATAcCCGAGTACACTGCTGACAACAAGAGCATGATCATATCTCTGGACGTGCT
GCCGAGCATTTATGAGGAAGGTG"/>
<sequence id="seq_Abit_Nuc_Apisto_302616"
taxon="Abit_Nuc_Apisto_302616" totalcount="4"
value="TGTTGGAGAAGCCAGAAGTGTTGCTTTAGTGGTGGTTGTGTCCCGGGAAG
TGACGTTCATGCCCGCCCCACCTGCCGTCTCCCATCAGCATGTGATGGAGTTTGAT
GTGGAGGAGGATGACTCCTCTCGGTCACCATCTCCTCAGGAGATTCTGCTcGAGG
TGGAGCTGGATGAAAACGAAGTCAAAGAGTTTGAGAGGCAGGTGAAGATCATCA

CCATACCCGAGTACACTGCTGACAACAAGAGCATGATCATATCTCTGGACGTGCT
GCCGAGCATTTATGAGGAAGGTG"/>
<sequence id="seq_Acac_Nuc_Apisto_300690"
taxon="Acac_Nuc_Apisto_300690" totalcount="4"
value="TGTTGGAGAAGCCAGAAGTGTTGCTTTAGTGGTGGTTGTATCACGGGAAG
TGACGTTCATGCCCGCCCCACCTGCCGTCTCCCATCAGCATGTGATGGAGTTTGAT
GTGGAGGAGGATGACTCCTCTCGGTCACCATCTCCTCAGGAGATTCTGCTCGAGG
TGGAGCTGGATGAAAACGAAGTCAAAGAGTTTGAGAAGCAGGTGAAGATCATCA
CCATACCCGAGTACACGGCTGACAACAAGAGCATGATCATATCTCTGGACGTGCT
GCCGAGCATTTATGAGGAAGGTG"/>
<sequence id="seq_Acac_Nuc_Apisto_300709"
taxon="Acac_Nuc_Apisto_300709" totalcount="4"
value="TGTTGGAGAAGCCAGAAGTGTTGCTTTAGTGGTGGTTGTATCACGGGAAG
TGACGTTCATGCCCGCCCCACCTGCCGTCTCCCATCAGCATGTGATGGAGTTTGAT
GTGGAGGAGGATGACTCCTCTCGGTCACCATCTCCTCAGGAGATTCTGCTCGAGG
TGGAGCTGGATGAAAACGAAGTCAAAGAGTTTGAGAAGCAGGTGAAGATCATCA
CCATACCCGAGTACACGGCTGACAACAAGAGCATGATCATATCTCTGGACGTGCT
GCCGAGCATTTATGAGGAAGGTG"/>
<sequence id="seq_Acac_Nuc_Apisto_301162"
taxon="Acac_Nuc_Apisto_301162" totalcount="4"
value="TgTtGGAGAAGCCAGAAGTGTTGCTTTAGTGGTGGTTGTATCACGGGAAGT
GACGTTCATGCCCGCCCCACCTGCCGTCTCCCATCAGCATGTGATGGAGTTTGAT
GTGGAGGAGGATGACTCCTCTCGGTCACCATCTCCTCAGGAGATTCTGCTCGAGG
TGGAGCTGGATGAAAACGAAGTCAAAGAGTTTGAGAAGCAGGTGAAGATCATCA
CCATACCCGAGTACACGGCTGACAACAAGAGCATGATCATATCTCTGGACGTGCT
GCCGAGCATTTATGAGGAAGGTG"/>
<sequence id="seq_Acac_Nuc_Apisto_301422"
taxon="Acac_Nuc_Apisto_301422" totalcount="4"
value="TGTTGGAGAAGCCAGAAGTGTTGCTTTAGTGGTGATTGTATCACGGGAAG
TGACGTTCATGCCCGCCCCACCTGCCGTCTCCCATCAGCATGTGATGGAGTTTGAT
GTGGAGGAGGATGACTCCTCTCGGTCACCATCTCCTCAGGAGATTCTGCTCGAGG
TGGAGCTGGATGAAAACGAAGTCAAaGAGTTTGAGAAGCAGGtGAAGATcaTCAC
CATACCCGAgTACACGgCTgACAACAAGAgCATGATcATATCTCTGGACGTGCTGC
CGAGCATTTATGAGGAAGGTG"/>
<sequence id="seq_Acac_Nuc_Apisto_301505"
taxon="Acac_Nuc_Apisto_301505" totalcount="4"
value="TGTTGGAGAAGCCAGAAGTGTTGCTTTAGTGGTGATTGTATCACGGGAAG
TGACGTTCATGCCCGCCCCACCTGCCGTCTCCCATCAGCATGTGATGGAGTTTGAT
GTGGAGGAGGATGACTCCTCTCGGTCACCATCTCCTCAGGAGATTCTGCTCGAGG
TGGAGCTGGATGAAAACGAAGTCAAAGAGTTTGAGAAGCAGGTGAAGATCATCA
CCATACCCGAGTACACGGCTGACAACAAGAGCATGATCATATCTCTGGACGTGCT
GCCGAGCATTTATGAGGAAGGTG"/>
<sequence id="seq_Acac_Nuc_Apisto_302195"
taxon="Acac_Nuc_Apisto_302195" totalcount="4"
value="TGTTGGAGAAGCCAGAAGTGTTGCTTTAGTGGTGGTTGTATCACGGGAAG
TGACGTTCATGCCCGCCCCACCTGCCGTCTCCCATCAGCATGTGATGGAGTTTGAT
GTgGAGGAGGATGACTCCTCTCGGTCACCATCTCCTCAGGAGATTCTGCTCGAGG
TGGAGCTGGATGAAAACGAAGTCAAAGAGTTTGAGAAGCAGGTGAAGATCATCA
CCATACCCGAGTACACGGCTGACAACAAGAGCATGATCATATCTCTGGACGTGCT
GCCGAGCATTTATGAGGAAGGTG"/>

<sequence id="seq_Acac_Nuc_Apisto_302196"
taxon="Acac_Nuc_Apisto_302196" totalcount="4"
value="TGTTGGAGAAGCCAGAAGTGTTGCTTTAGTGGTGGTTGTATCACGGGAAG
TGACGTTCATGCCCGCCCCACCTGCCGTCTCCCATCAGCATGTGATGGAGTTTGAT
GTGGAGGAGGATGACTCCTCTCGGTCACCATCTCCTCAGGAGATTCTGCTCGAGG
TGGAGCTGGATGAAAACGAAGTCAAAGAGTTTGAGAAGCAGGTGAAGATCATCA
CCATACCCGAGTACACGGCTGACAACAAGAGCATGATCATATCTCTGGACGTGCT
GCCGAGCATTTATGAGGAAGGTG"/>
<sequence id="seq_Acac_Nuc_Apisto_302197"
taxon="Acac_Nuc_Apisto_302197" totalcount="4"
value="TGTTGGAGAAGCCAGAAGTGTTGCTTTAGTGGTGgTTGTATCACGGGAAGT
GACGTTCATGCCCGCCCCACCTGCCGTCTCCCATCAGCATGTGATGGAGTTTGAT
GTGGAGGAGGATGACTCCTCTCGGTCACCATCTCCTCAGGAGATTcTGCTCGAGG
TGGAGCTGGATGAAAACGAAGTCAAAGAGTTTGAGAAGCAGGTGAAGATCATCA
CCATACCCGAGTACACGGCTGACAACAAGAGCATGATCATATCTCtgGACGTGCT
GCCGAGCATTTATGAGGAAGGTG"/>
<sequence id="seq_Acar_Nuc_Apisto_302364"
taxon="Acar_Nuc_Apisto_302364" totalcount="4"
value="TGTTGGAGAAGCCAGAAGTGTTGCTTTAGTGGTAGTCGTATCACGGGAAG
TGACATTCATGCCCGCCCCACCTGCCGTCTCCCATCAGCATGTGATGGAGTTCGA
TGTGGAGGAAGATGACTCTTCTCGGTCACCGTCTCCTCAGGAGATTcTGCTGGAA
GTGGAGCTGGATGAAAACGAGGTCAAAGAGTTTGAGAAGCAGGTGAAGATCATC
ACCATACCCGAGTACACAGCTGACAACAAGAGCATGATCATATCTCTGGATGTGC
TACCGAGCATTTATGAGGAAGGTG"/>
<sequence id="seq_Acar_Nuc_Apisto_302365"
taxon="Acar_Nuc_Apisto_302365" totalcount="4"
value="TGTTGGAGAAGCCAGAAGTGTTGCTTTAGTGGTAGTCGTATCACGGGAAG
TGACATTCATGCCCGCCCCACCTGCCGTCTCCCATCAGCATGTGATGGAGTTCGA
TGTGGAGGAAGATGACTCTTCTCGGTCACCGTCTCCTCAGGAGATTCTGCTGGAA
GTGGAGCTGGATGAAAACGAGGTCAAAGAGTTTGAGAAGCAGGTGAAGATCATC
ACCATACCCGAGTACACAGCTGACAACAAGAGCATGATCATATCTCTGGATGTGC
TACCGAGCATTTATGAGGAAGGTG"/>
<sequence id="seq_Acar_Nuc_Apisto_302366"
taxon="Acar_Nuc_Apisto_302366" totalcount="4"
value="TGTTGGAGAAGCCAGAAGTGTTGCTTTAGTGGTAGTCGTATCACGGGAAG
TGACATTCATGCCCGCCCCACCTGCCGTCTCCCATCAGCATGTGATGGAGTTCGA
TGTGGAGGAAGATGACTcTTCTCGGTCACCGTCTCCTCAGGAGATTcTGCTGGAAG
TGGAGCTGGATGAAAACGAGGTCAAAGAGTTTGAGAAGCAGGTGAAGATCATCA
CCATACCCGAGTACACAGCTGACAACAAGAGCATGATCATATCTCTGGATGTGCT
ACCGAGCATTTATGAGGAAGGTG"/>
<sequence id="seq_Acar_Nuc_Apisto_302367"
taxon="Acar_Nuc_Apisto_302367" totalcount="4"
value="TGTTGGAGAAGCCAGAAGTGTTGCTTTAGTGGTAGTCGTATCACGGGAAG
TGACATTCATGCCCGCCCCACCTGCCGTCTCCCATCAGCATGTGATGGAGTTCGA
TGTGGAGGAAGATGACTcTTCTCGGTCACCGTCTCCTCAGGAGATTcTGCTGGAAG
TGGAGCTGGATGAAAACGAGGTCAAAGAGTTTGAGAAGCAGGTGAAGATCATCa
CCATACCCGAGTACACAgCTGACAACAAGAGCATGATcaTaTCTCTGGATGTGCTA
CCGAGCATTTATGAGGAAGGTG"/>
<sequence id="seq_Acin_Nuc_Apisto_301616"
taxon="Acin_Nuc_Apisto_301616" totalcount="4"

value="TGTTGGAGAAGCCAGAAGTGTTGCTTTAGTGGTAGTTGTATCACGGGAAG
TGACATTCATGCCCGCCCCACCTGCCGTCTCCCACCAGCATGTGATGGAGTTCGA
TGTGGAGGAAGATGACTCTTCTCGGTCACCGTCTCCTCAGGAGATTCTGCTGGAA
GTGGAGCTGGATGAAAACGAGGTCAAAGAGTTTGAGAAGCAGGTGAAGATCATC
ACCATACCCGAGTACACAGCTGACAACAAGAGCATGATCATATCTCTGGATGTGC
TACCGAGCATTTATGAGGAAGGTG"/>
<sequence id="seq_Acin_Nuc_Apisto_301618”
taxon="Acin_Nuc_Apisto_301618” totalcount="4"
value="TGTTGGAGAAGCCAGAAGTGTTGCTTTAGTGGTAGTTGTATCACGGGAAG
TGACATTCATGCCCGCCCCACCTGCCGTCTCCCACCAGCATGTGATGGAGTTCGA
TGTGGAGGAAGATGACTCTTCTCGGTCACCGTCTCCTCAGGAGATTcTGCTGGAA
GTGGAGCTGGATGAAAACGAGGTCAAAGAGTTTGAGAAGCAGGTGAAGATCATC
ACCATACCCGAGTACACAGCTGACAACAAGAGCATGATCATATCTCTGGATGTGC
TACCGAGCATTTATGAGGAAGGTG"/>
<sequence id="seq_Acin_Nuc_Apisto_300371"
taxon="Acin_Nuc_Apisto_300371" totalcount="4"
value="TGTTGGAGAAGCCAGAAGTGTTGCTTTAGTGGTAGTTGTATCACGGGAAG
TGACATTCATGCCCGCCCCACCTGCCGTCTCCCACCAGCATGTGATGGAGTTCGA
TGTGGAGGAAGATGACTCTTCTCGGTCACCGTCTCCTCAGGAGATTCTGCTGGAA
GTGGAGCTGGATGAAAACGAGGTCAAAGAGTTTGAGAAGCAGGTGAAGATCATC
ACCATACCCGAGTACACAGCTGACAACAAGAGCATGATCATATCTCTGGATGTGC
TACCGAGCATTTATGAGGAAGGTG"/>
<sequence id="seq_Acin_Nuc_Apisto_301619”
taxon="Acin_Nuc_Apisto_301619” totalcount="4"
value="TGTTGGAGAAGCCAGAAGTGTTGCTTTAGTGGTAGTTGTATCACGGGAAG
TGACATTCATGCCCGCCCCACCTGCCGTCTCCCACCAGCATGTGATGGAGTTCGA
TGTGGAGGAAGATGACTCTTCTCGGTCACCGTCTCCTCAGGAGATTcTGCTGGAA
GTGGAGCTGGATGAAAACGAGGTCAAAGAGTTTGAGAAGCAGGTGAAGATCATC
ACCATACCCGAGTACACAGCTGACAACAAGAGCATGATCATATCTCTGGATGTGC
TACCGAGCATTTATGAGGAAGGTG"/>
<sequence id="seq_Acin_Nuc_Apisto_300374"
taxon="Acin_Nuc_Apisto_300374" totalcount="4"
value="TGTTGGAGAAGCCAGAAGTGTTGCTTTAGTGGTAGTTGTATCACGGGAAG
TGACATTCATGCCCGCCCCACCTGCCGTCTCCCACCAGCATGTGATGGAGTTCGA
TGTGGAGGAAGATGACTCTTCTCGGTCACCGTCTCCTCAGGAGATTCTGCTGGAA
GTGGAGCTGGATGAAAACGAGGTCAAAGAGTTTGAGAAGCAGGTGAAGATCATC
ACCATACCCGAGTACACAGCTGACAACAAGAGCATGATCATATCTCTGGATGTGC
TACCGAGCATTTATGAGGAAGGTG"/>
<sequence id="seq_Acin_Nuc_Apisto_300378"
taxon="Acin_Nuc_Apisto_300378" totalcount="4"
value="TGTTGGAGAAGCCAGAAGTGTTGCTTTAGTGGTAGTTGTATCACGGGAAG
TGACATTCATGCCCGCCCCACCTGCCGTCTCCCACCAGCATGTGATGGAGTTCGA
TGTGGAGGAAGATGACTCTTCTCGGTCACCGTCTCCTCAGGAGATTCTGCTGGAA
GTGGAGCTGGATGAAAACGAGGTCAAAGAGTTTGAGAAGCAGGTGAAGATCATC
ACCATACCCGAGTACACAGCTGACAACAAGAGCATGATCATATCTCTGGATGTGC
TACCGAGCATTTATGAGGAAGGTG"/>
<sequence id="seq_Acin_Nuc_Apisto_300379"
taxon="Acin_Nuc_Apisto_300379" totalcount="4"
value="TGTTGGAGAAGCCAGAAGTGTTGCTTTAGTGGTAGTTGTATCACGGGAAG
TGACATTCATGCCCGCCCCACCTGCCGTCTCCCACCAGCATGTGATGGAGTTCGA

TGTGGAGGAAGATGACTCTTCTCGGTCACCGTCTCCTCAGGAGATTCTGCTGGAA
GTGGAGCTGGATGAAAACGAGGTCAAAGAGTTTGAGAAGCAGGTGAAGATCATC
ACCATACCCGAGTACACAGCTGACAACAAGAGCATGATCATATCTCTGGATGTGC
TACCGAGCATTTATGAGGAAGGTG"/>
<sequence id="seq_Acin_Nuc_Apisto_300380"
taxon="Acin_Nuc_Apisto_300380" totalcount="4"
value="TGTTGGAGAAGCCAGAAGTGTTGCTTTAGTGGTAGTTGTATCACGGGAAG
TGACATTCATGCCCGCCCCACCTGCCGTCTCCCACCAGCATGTGATGGAGTTCGA
TGTGGAGGAAGATGACTCTTCTCGGTCACCGTCTCCTCAGGAGATTCTGCTGGAA
GTGGAGCTGGATGAAAACGAGGTCAAAGAGTTTGAGAAGCAGGTGAAGATCATC
ACCATACCCGAGTACACAGCTGACAACAAGAGCATGATCATATCTCTGGATGTGC
TACCGAGCATTTATGAGGAAGGTG"/>
<sequence id="seq_Acin_Nuc_Apisto_301628”
taxon="Acin_Nuc_Apisto_301628” totalcount="4"
value="TGTTGGAGAAGCCAGAAGTGTTGCTTTAGTGGTAGTTGTATCACGGGAAG
TGACATTCATGCCCGCCCCACCTGCCGTCTCCCACCAGCATGTGATGGAGTTCGA
TGTGGAGGAAGATGACTCTTCTCGGTCACCGTCTCCTCAGGAGATTCTGCTGGAA
GTGGAGCTGGATGAAAACGAGGTCAAAGAGTTTGAGAAGCAGGTGAAGATCATC
ACCATACCCGAGTACACAGCTGACAACAAGAGCATGATCATATCTCTGGATGTGC
TACCGAGCATTTATGAGGAAGGTG"/>
<sequence id="seq_Acin_Nuc_Apisto_301629”
taxon="Acin_Nuc_Apisto_301629” totalcount="4"
value="TGTTGGAGAAGCCAGAAGTGTTGCTTTAGTGGTAGTTGTATCACGGGAAG
TGACATTCATGCCCGCCCCACCTGCCGTCTCCCACCAGCATGTGATGGAGTTCGA
TGTGGAGGAAGATGACTCTTCTCGGTCACCGTCTCCTCAGGAGATTCTGCTGGAA
GTGGAGCTGGATGAAAACGAGGTCAAAGAGTTTGAGAAGCAGGTGAAGATCATC
ACCATACCCGAGTACACAGCTGACAACAAGAGCATGATCATATCTCTGGATGTGC
TACCGAGCATTTATGAGGAAGGTG"/>
<sequence id="seq_Aere_Nuc_Apisto_300401"
taxon="Aere_Nuc_Apisto_300401" totalcount="4"
value="TGTTGGAGAAGCCAGAAGTGTTGCTTTAGTGGTGGTTGTGTCCCGGGAAG
TGACGTTCATGCCCGCCCCACCTGCCGTCTCCCATCAGCaTGTGATGGAGTTTGAT
GTGGAGGAGGATGACTCCTCTCGGTCaCCaTCTCCTCaGGAGATTCTGCTCGAGGT
GGAGCTGGATGAAAACGAAGTCAAAGAGTTTGAGAGGCAGGTGAAGATCATCAC
CATACCCGAGTAcACTGCTgACAAcAAGAgCATGATCATATCTCTGGACGTGCTGC
CGAGCATTTATGAGGAAGGTG"/>
<sequence id="seq_Aere_Nuc_Apisto_300402"
taxon="Aere_Nuc_Apisto_300402" totalcount="4"
value="TGTTGGAGAAGCCAGAAGTGTTGCTTTAGTGGTGGTTGTGTCCCGGGAAG
TGACGTTCATGCCCGCCCCACCTGCCGTCTCCCATCAGCATGTGATGGAGTTTGAT
GTGGAGGAGGATGACTCCTCTCGGTCACcaTCTCCTCaGGAGATTCTGCTCGAGGT
GGAGCTGGATGAAAACGAAGtCAAAGAGTTTGAGAGGCAGGTGAaGATcaTCACC
ATACCCGAgTAcACTgCTGaCAACaAGAgCATGATCATATCTCTGGACGTGCTGCCG
AGCATTTATGAGGAAGGTG"/>
<sequence id="seq_Aere_Nuc_Apisto_300403"
taxon="Aere_Nuc_Apisto_300403" totalcount="4"
value="TGTTGGAGAAGCCAGAAGTGTTGCTTTAGTGGTGGTTGTGTCCCGGGAAG
TGACGTTCATGCCCGCCCCACCTGCCGTCTCCCATCAGCaTGTGATGGAGTTTGAT
GTGGAGGAGGATGACTCCTCTCGGTCACCaTCTCCTCAGGAGATTCTGCTCGAGG
TGGAGCTGGATGAAAACGAAGTCAAAGAGTTTGAGAGGCAGGTGAAGAtCATCA

CCATACCCGAGTAcACTGCTGACAACAAGAGCATGATCATATCTCTGGACGTGCT
GCCGAGCATTTATGAGGAAGGTG"/>
<sequence id="seq_Aere_Nuc_Apisto_300405"
taxon="Aere_Nuc_Apisto_300405" totalcount="4"
value="TGTTGGAGAAGCCAGAAGTGTTGCTTTAGTGGTGGTTGTGTCCCGGGAAG
TGACGTTCATGCCCGCCCCACCTGCCGTCTCCCATCAGCATGTGATGGAGTTTGAT
GTGGAGGAGGATGACTCCTCTCGGTCACCATCTCCTCAGGAGATTCTGCTCGAGG
TGGAGCTGGATGAAAACGAAGTCAAAGAGTTTGAGAGGCAGGTGAAGATCATCA
CCATACCCGAGTACACTGCTGACAACAAGAGCATGATCATATCTCTGGACGTGCT
GCCGAGCATTTATGAGGAAGGTG"/>
<sequence id="seq_Aere_Nuc_Apisto_300412"
taxon="Aere_Nuc_Apisto_300412" totalcount="4"
value="TGTTGGAGAAGCCAGAAGTGTTGCTTTAGTGGTGGTTGTGTCCCGGGAAG
TGACGTTCATGCCCGCCCCACCTGCCGTCTCCCATCAGCATGTGATGGAGTTTGAT
GTGGAGGAGGATGACTCCTCTCGGTCACCATCTCCTCAGGAGATTCTGCTCGAGG
TGGAGCTGGATGAAAACGAAGTCAAAGAGTTTGAGAGGCAGGTGAAGATCATCA
CCATACCCGAGTACACTGCTGACAACAAGAGCATGATCATATCTCTGGACGTGCT
GCCGAGCATTTATGAGGAAGGTG"/>
<sequence id="seq_Aeun_Nuc_Apisto_302554"
taxon="Aeun_Nuc_Apisto_302554" totalcount="4"
value="TGTTGGAGAAGCCAGAAGTGTTGCTTTAGTGGTAGTTGTATCACGGGAAG
TGACATTCATGCCCGCCCCACCTGCCGTCTCCCACCAGCATGTGATGGAGTTCGA
TGTGGAGGAAGATGACTCTTCTCGGTCACCGTCTCCTCAGGAGATTcTGCTGGAA
GTGGAGCTGGATGAAAACGAGGTCAAAGAGTTTGAGAAGCAGGTGAAGATCATC
ACCATACCCGAGTACACAGCTGACAACAAGAGCATGATCATATCTCTGGATGTGC
TACCGAGCATTTATGAGGAAGGTG"/>
<sequence id="seq_Aeun_Nuc_Apisto_302555"
taxon="Aeun_Nuc_Apisto_302555" totalcount="4"
value="TGTTGGAGAAGCCAGAAGTGTTGCTTTAGTGGTAGTTGTATCACGGGAAG
TGACATTCATGCCCGCCCCACCTGCCGTCTCCCACCAGCATGTGATGGAGTTCGA
TGTGGAGGAAGATGACTCTTCTCGGTCACCGTCTCCTCAGGAGATTcTGCTGGAA
GTGGAGCTGGATGAAAACGAGGTCAAAGAGTTTGAGAAGCAGGTGAAGATCATC
ACCATACCCGAGTACACAGCTGACAACAAGAGCATGATCATATCTCTGGATGTGC
TACCGAGCATTTATGAGGAAGGTG"/>
<sequence id="seq_Aeun_Nuc_Apisto_302556"
taxon="Aeun_Nuc_Apisto_302556" totalcount="4"
value="TGTTGGAGAAGCCAGAAGTGTTGCTTTAGTGGTAGTTGTATCACGGGAAG
TGACATTCATGCCCGCCCCACCTGCCGTCTCCCACCAGCATGTGATGGAGTTCGA
TGTGGAGGAAGATGACTCTTCTCGGTCACCGTCTCCTCAGGAGATTcTGCTGGAA
GTGGAGCTGGATGAAAACGAGGTCAAAGAGTTTGAGAAGCAGGTGAAGATCATC
ACCATACCCGAGTACACAGCTGACAACAAGAGCATGATCATATCTCTGGATGTGC
TACCGAGCATTTATGAGGAAGGTG"/>
<sequence id="seq_Aeun_Nuc_Apisto_302557"
taxon="Aeun_Nuc_Apisto_302557" totalcount="4"
value="TGTTGGAGAAGCCAGAAGTGTTGCTTTAGTGGTAGTTGTATCACGGGAAG
TGACATTCATGCCCGCCCCACCTGCCGTCTCCCACCAGCATGTGATGGAGTTCGA
TGTGGAGGAAGATGACTCTTCTCGGTCACCGTCTCCTCAGGAGATTcTGCTGGAA
GTGGAGCTGGATGAAAACGAGGTCAAAGAGTTTGAGAAGCAGGTGAAGATCATC
ACCATACCCGAGTACACAGCTGACAACAAGAGCATGATCATATCTCTGGATGTGC
TACCGAGCATTTATGAGGAAGGTG"/>

<sequence id="seq_Aeun_Nuc_Apisto_302558"
taxon="Aeun_Nuc_Apisto_302558" totalcount="4"
value="TGTTGGAGAAGCCAGAAGTGTTGCTTTAGTGGTAGTTGTATCACGGGAAG
TGACATTCATGCCCGCCCCACCTGCCGTCTCCCACCAGCATGTGATGGAGTTCGA
TGTGGAGGAAGATGACTCTTCTCGGTCACCGTCTCCTCAGGAGATTCTGCTGGAA
GTGGAGCTGGATGAaAACGAGGTCAAAGAGTTTGAGAAGCAGGTGAAGATCATC
ACCATACCCGAGTACACAGCTGACAACAAGAGCATGATCATATCTCTGGATGTGC
TACCGAGCATTTATGAGGAAGGTG"/>
<sequence id="seq_Aeun_Nuc_Apisto_302559"
taxon="Aeun_Nuc_Apisto_302559" totalcount="4"
value="TGTTGGAGAAGCCAGAAGTGTTGCTTTAGTGGTAGTTGTATCACGGGAAG
TGACATTCATGCCCGCCCCACCTGCCGTCTCCCACCAGCATGTGATGGAGTTCGA
TGTGGAGGAAGATGACTCTTCTCGGTCACCGTCTCCTCAGGAGATTCTGCTGGAA
GTGGAGCTGGATGAAAACGAGGTCAAAGAGTTTGAGAAGCAGGTGAAGATCATC
ACCATACCCGAGTACACAGCTGACAACAAGAGCATGATCATATCTCTGGATGTGC
TACCGAGCATTTATGAGGAAGGTG"/>
<sequence id="seq_Aeun_Nuc_Apisto_302560"
taxon="Aeun_Nuc_Apisto_302560" totalcount="4"
value="TGTTGGAGAAGCCAGAAGTGTTGCTTTAGTGGTAGTTGTATCACGGGAAG
TGACATTCATGCCCGCCCCACCTGCCGTCTCCCACCAGCATGTGATGGAGTTCGA
TGTGGAGGAAGATGACTCTTCTCGGTCACCGTCTCCTCAGGAGATTCTGCTGGAA
GTGGAGCTGGATGAAAACGAGGTCAAAGAGTTTGAGAAGCAGGTGAAGATCATC
ACCATACCCGAGTACACAGCTGACAACAAGAGCATGATCATATCTCTGGATGTGC
TACCGAGCATTTATGAGGAAGGTG"/>
<sequence id="seq_Aeun_Nuc_Apisto_302938"
taxon="Aeun_Nuc_Apisto_302938" totalcount="4"
value="TGTTGGAGAAGCCAGAAGTGTTGCTTTAGTGGTAGTTGTATCACGGGAAG
TGACATTCATGCCCGCCCCACCTGCCGTCTCCCACCAGCATGTGATGGAGTTcGAT
GTGGAGGAAGATGACTCTTCTCGGTCACCGTCTCCTCAGGAGATTCTGCTGGAAG
TGGAGCTGGATGAAAACGAGGTCAAAGAGTTTGAGAAGCAGGTGAAGATCATCA
CCATACCCGAGTACACAGCTGACAACAAGAGCATGATCATATCTCTGGATGTGCT
ACCGAGCATTTATGAGGAAGGTG"/>
<sequence id="seq_Aeun_Nuc_Apisto_302940"
taxon="Aeun_Nuc_Apisto_302940" totalcount="4"
value="TGTTGGAGAAGCCAGAAGTGTTGCTTTAGTGGTAGTTGTATCACGGGAAG
TGACATTCATGCCCGCCCCACCTGCCGTCTCCCAcCAGCATGTGATGGAGTTcGAT
GTGGAGGAAGATGACTCTTCTCGGTCACCGTCTCCTCAGGAGATTCTGCTGGAAG
TGGAGCTGGATGAAAACGAgGTCAAAGAGTTTGAGAAGCAGGTGAAGATCATCA
CCATACCCGAGTACACaGCTGACAACAAGAGCATGATCATATCTCTGGATGTGCTa
CCGAGCATTTATGAGGAAGGTG"/>
<sequence id="seq_Aeun_Nuc_Apisto_301804"
taxon="Aeun_Nuc_Apisto_301804" totalcount="4"
value="TGTTGGAGAAGCCAGAAGTGTTGCTTTAGTGGTAGTTGTATCACGGGAAG
TGACATTCATGCCCGCCCCACCTGCCGTCTCCCACCAGCATGTGATGGAGTTCGA
TGTGGAGGAAGATGACTCTTCTCGGTCACCGTCTCCTCAGGAGATTCTGCTGGAA
GTGGAGCTgGATGAAAACGAGGTCAAAGAGTTTGAGAAGCAGGTGAAGATCATC
ACCATACCCGAGTACACAgCTGACAACAAGAGCATGATCATATCTCTGGATGTGC
TACCGAGCATTTATGAGGAAGGTG"/>
<sequence id="seq_Ahua_Nuc_Apisto_302705"
taxon="Ahua_Nuc_Apisto_302705" totalcount="4"

value="TGTTGGAGAAGCCAGAAGTGTTGCTTTAGTGGTAGTTGTATCACGGGAAG
TGACATTCATGCCCGCCCCACCTGCCGTCTCCCACCAGCATGTGATGGAGTTCGA
TGTGGAGGAAGATGACTCTTCTCGGTCACCGTCTCCTCAGGAGATTCTGCTGGAA
GTGGAGCTGGATGAAAACGAGGTCAAAGAGTTTGAGAAGCAGGTGAAGATCATC
ACCATACCCGAGTACACAGCTGACAACAAGAGCATGATCATATCTCTGGATGTGC
TACCGAGCATTTATGAGGAAGGTG"/>
<sequence id="seq_Ahua_Nuc_Apisto_302706"
taxon="Ahua_Nuc_Apisto_302706" totalcount="4"
value="TGTTGGAGAAGCCAGAAGTGTTGCTTTAGTGGTAGTTGTATCACGGGAAG
TGACATTCATGCCCGCCCCACCTGCCGTCTCCCACCAGCATGTGATGGAGTTCGA
TGTGGAGGAAGATGACTCTTCTCGGTCACCGTCTCCTCAGGAGATTCTGCTGGAA
GTGGAGCTGGATGAAAACGAGGTCAAAGAGTTTGAGAAGCAGGTGAAGATCATC
ACCATACCCGAGTACACAGCTGACAACAAGAGCATGATCATATCTCTGGATGTGC
TACCGAGCATTTATGAGGAAGGTG"/>
<sequence id="seq_Ahua_Nuc_Apisto_302707"
taxon="Ahua_Nuc_Apisto_302707" totalcount="4"
value="TGTTGGAGAAGCCAGAAGTGTTGCTTTAGTGGTAGTTGTATCACGGGAAG
TGACATTCATGCCCGCCCCACCTGCCGTCTCCCACCAGCATGTGATGGAGTTCGA
TGTGGAGGAAGATGACTcTTcTCGGTCACCGTCTCCTCAGGAGATTcTGCTGGAAG
TGGAGCTGGATGAAAACGAGGTCAAAGAGTTTGAGAAGCAGGTGAAGATCATCA
CCATACCCGAGTACACAGCTGACAACAAGAGCATGATCATATCTCTGGATGTGCT
ACCGAGCATTTATGAGGAAGGTG"/>
<sequence id="seq_Ahua_Nuc_Apisto_302708"
taxon="Ahua_Nuc_Apisto_302708" totalcount="4"
value="TGTTGGAGAAGCCAGAAGTGTTGCTTTAGTGGTAGTTGTATCACGGGAAG
TGACATTCATGCCCGCCCCACCTGCCGTCTCCCACCAGCATGTGATGGAGTTCGA
TGTGGAGGAAGATGACTcTTcTCGGTCACCGTCTCCTCAGGAGATTcTGCTGGAAG
TGGAGCTGGATGAAAACGAGGTCAAAGAGTTTGAGAAGCAGGTGAAGATCATCA
CCATACCCGAGTACACAGCTGACAACAAGAGCATGATCATATCTCTGGATGTGCT
ACCGAGCATTTATGAGGAAGGTG"/>
<sequence id="seq_Ahua_Nuc_Apisto_302709"
taxon="Ahua_Nuc_Apisto_302709" totalcount="4"
value="TGTTGGAGAAGCCAGAAGTGTTGCTTTAGTGGTAGTTGTATCACGGGAAG
TGACATTCATGCCCGCCCCACCTGCCGTCTCCCACCAGCATGTGATGGAGTTCGA
TGTGGAGGAAGATGACTCTTCTCGGTCACCGTCTCCTCAGGAGATTcTGCTGGAA
GTGGAGCTGGATGAAAACGAGGTCAAAGAGTTTGAGAAGCAGGTGAAGATCATC
ACCATACCCGAGTACACAGCTGACAACAAGAGCATGATCATATCTCTGGATGTGC
TACCGAGCATTTATGAGGAAGGTG"/>
<sequence id="seq_Ajur_Nuc_Apisto_302878"
taxon="Ajur_Nuc_Apisto_302878" totalcount="4"
value="TGTTGGAGAAGCCAGGAGTGTTGCTTTAGTGGTGGTTGTATCACGGGAAG
TGACGTTCTTGCCCGCCCCACCTGCCGTCTCCCATCAGCATGTGATGGAGTTTGAT
GTGGAGGAAGATGACTCCTCTCGGTCACCATCTCCTCAGGAGATTCTGCTCGAGG
TGGAGCTGGATGAAAACGAAGTCAAAGAGTTTGAGAAGCAGGTGAAGATCATCA
CCATACCCGAGTACACGGCTGACAACAAGAGCATGATCATATCTCTGGACGTGCT
GCCGAGCATTTATGAGGAAGGTG"/>
<sequence id="seq_Ajur_Nuc_Apisto_302879"
taxon="Ajur_Nuc_Apisto_302879" totalcount="4"
value="TGTTGGAGAAGCCAGGAGTGTTGCTTTAGTGGTGGTTGTATCACGGGAAG
TGACGTTCtTGCCCGCCCCACCTGCCGTCTCCCATCAGCATGTGATGGAGTTTGAT

GTGGAGGAAGATGACTCCTCTCGGTCACCATCTCCTCAGGAGATTCTGCTCGAGG
TGGAGCTGGATGAAAACGAAGTCAAAGAGTTTGAGAAGCAGGTGAAGATCATCA
CCATACCCGAGTACACGGCTGACAACAAGAGCATGATCATATCTCTGGACGTGCT
GCCGAGCATTTATGAGGAAGGTG"/>
<sequence id="seq_Ajur_Nuc_Apisto_302880”
taxon="Ajur_Nuc_Apisto_302880” totalcount="4"
value="TGTTGGAGAAGCCAGGAGTGTTGCTTTAGTGGTGGTTGTATCACGGGAAG
TGACGTTCtTGCCcGCCCCACCTGCCGTCTCCCATCAGCATGTGATGGAGTTTGAT
GTGGAGGAAGATGACTCCTCTCGGTCACCATCTCCTCAGGAGATTCTGCTCGAGG
TGGAGCTGGATGAAAACGAAGTCAAAGAGTTTGAGAAGCAGGTGAAGATCATCA
CCATACCCGAGTACACGGCTGACAACAAGAGCATGATCATATCTCTGGACGTGCT
GCCGAGCATTTATGAGGAAGGTG"/>
<sequence id="seq_Ameg_Nuc_Apisto_302869"
taxon="Ameg_Nuc_Apisto_302869" totalcount="4"
value="TGTTGGAGAAGCCAGAAGTGTTGCTTTAGTGGTGGTTGTATCACGGGAAG
TGACGTTCATGCCCGCCCCACCTGCCGTCTCCCATCAGCATGTGATGGAGTTTGAT
GTGGAGGAGGATGACTCCTCTCGGTCACCATCTCCTCAGGAGATTCTGCTCGAGG
TGGAGCTGGATGAAAACGAAGTCAAAGAGTTTGAGAAGCAGGTGAAGATCATCA
CCATACCCGAGTACACAGCTGACAACAAGAGCATGATCATATCTCTGGACGTGCT
GCCGAGCATTTATGAGGAAGGTG"/>
<sequence id="seq_Ameg_Nuc_Apisto_302870"
taxon="Ameg_Nuc_Apisto_302870" totalcount="4"
value="TGTTGGAGAAGCCAGAAGTGTTGCTTTAGTGGTGGTTGTATCACGGGAAG
TGACGTTCATGCCCGCCCCACCTGCCGTCTCCCATCAGCATGTGATGGAGTTTGAT
GTGGAGGAGGATGACTCCTCTCGGTCACCATCTCCTCAGGAGATTCTGCTCGAGG
TGGAGCTGGATGAAAACGAAGTCAAAGAGTTTGAGAAGCAGGTGAAGATCATCA
CCATACCCGAGTACACAGCTGACAACAAGAGCATGATCATATCTCTGGACGTGCT
GCCGAGCATTTATGAGGAAGGTG"/>
<sequence id="seq_Ameg_Nuc_Apisto_302871"
taxon="Ameg_Nuc_Apisto_302871" totalcount="4"
value="TGTTGGAGAAGCCAGAAGTGTTGCTTTAGTGGTGGTTGTATCACGGGAAG
TGACGTTCATGCCCGCCCCACCTGCCGTCTCCCATCAGCATGTGATGGAGTTTGAT
GTGGAGGAGGATGACTCCTCTCGGTCACCATCTCCTCAGGAGATTCTGCTCGAGG
TGGAGCTGGATGAAAACGAAGTCAAAGAGTTTGAGAAGCAGGTGAAGATCATCA
CCATACCCGAGTACACAGCTGACAACAAGAGCATGATCATATCTCTGGACGTGCT
GCCGAGCATTTATGAGGAAGGTG"/>
<sequence id="seq_Ameg_Nuc_Apisto_302872"
taxon="Ameg_Nuc_Apisto_302872" totalcount="4"
value="TGTTGGAGAAGCCAGAAGTGTTGCTTTAGTGGTGGTTGTATCACGGGAAG
TGACGTTCATGCCCGCCCCACCTGCCGTCTCCCATCAGCATGTGATGGAGTTTGAT
GTGGAGGAGGATGACTCCTCTCGGTCACCATCTCCTCAGGAGATTCTGCTCGAGG
TGGAGCTGGATGAAAACGAAGTCAAAGAGTTTGAGAAGCAGGTGAAGATCATCA
CCATACCCGAGTACACAGCTGACAACAAGAGCATGATCATATCTCTGGACGTGCT
GCCGAGCATTTATGAGGAAGGTG"/>
<sequence id="seq_Ameg_Nuc_Apisto_302873"
taxon="Ameg_Nuc_Apisto_302873" totalcount="4"
value="TGTTGGAGAAGCCAGAAGTGTTGCTTTAGTGGTGGTTGTATCACGGGAAG
TGACGTTCATGCCCGCCCCACCTGCCGTCTCCCATCAGCATGTGATGGAGTTTGAT
GTGGAGGAGGATGACTCCTCTCGGTCACCATCTCCTCAGGAGATTCTGCTCGAGG
TGGAGCTGGATGAAAACGAAGTCAAAGAGTTTGAGAAGCAGGTGAAGATCATCA

CCATACCCGAGTACACAGCTGACAACAAGAGCATGATCATATCTCTGGACGTGCT
GCCGAGCATTTATGAGGAAGGTG"/>
<sequence id="seq_Amel_Nuc_Apisto_301639”
taxon="Amel_Nuc_Apisto_301639” totalcount="4"
value="TGTTGGAGAAGCCAGAAGTGTTGCTTTAGTGGTaGTTGTATCACGGGAAGT
GACaTTCATGCCCGCCCCACCTGCCGTCTCCCAcCAGCATGTGATGGAGTTcGATG
TGGAGGAaGATGACTCtTCTCGGTCACCgTCTCCTCAGGAGATTcTGCTGGAAGTGG
AGCTGGATGAAAACGAgGTCAAAGAGTTTGAGAAGCAGGTGAAGATCATCACCA
TACCCGAGTACACaGCTGACAACAAGAGCATGATCATATCTCTGGAtGTGCTACCG
AGCaTTTATGAGGAAGGTG"/>
<sequence id="seq_Amel_Nuc_Apisto_301640"
taxon="Amel_Nuc_Apisto_301640" totalcount="4"
value="TGTTGGAGAAGCCAGAAGTGTTGCTTTAGTGGTAGTTGTATCACGGGAAG
TGACATTCATGCCCGCCCCACCTGCCGTCTCCCACCAGCATGTGATGGAGTTCGA
TGTGGAGGAAGATGACTCTTCTCGGTCACCGTCTCCTCAGGAGATTcTGCTGGAA
GTGGAGCTGGATGAAAACGAGGTCAAAGAGTTTGAGAAGCAGGTGAAGATCATC
ACCATACCCGAGTACACAGCTGACAACAAGAGCATGATCATATCTCTGGATGTGC
TACCGAGCATTTATGAGGAAGGTG"/>
<sequence id="seq_Amel_Nuc_Apisto_301641"
taxon="Amel_Nuc_Apisto_301641" totalcount="4"
value="TGTTGGAGAAGCCAGAAGTGTTGCTTTAGTGGTAGTTGTATCACGGGAAG
TGACATTCATGCCCGCCCCACCTGCCGTCTCCCACCAGCATGTGATGGAGTTCGA
TGTGGAGGAAGATGACTCTTCTCGGTCACCGTCTCCTCAGGAGATTCTGCTGGAA
GTGGAGCTGGATGAAAACGAGGTCAAAGAGTTTGAGAAGCAGGTGAAGATCATC
ACCATACCCGAGTACACAGCTGACAACAAGAGCATGATCATATCTCTGGATGTGC
TACCGAGCATTTATGAGGAAGGTG"/>
<sequence id="seq_Amel_Nuc_Apisto_301649”
taxon="Amel_Nuc_Apisto_301649” totalcount="4"
value="TGTTGGAGAAGCCAGAAGTGTTGCTTTAGTGGTAGTTGTATCACGGGAAG
TGACATTCATGCCCGCCCCACCTGCCGTCTCCCACCAGCATGTGATGGAGTTCGA
TGTGGAGGAAGATGACTcTTCTCGGTCACCGTCTCCTCAGGAGATTcTGCTGGAAG
TGGAGCTGGATGAAAACGAGGTCAAAGAGTTTGAGAAGCAGGTGAAGATCATCA
CCATACCCGAGTACACAGCTGACAACAAGAGCATGATCATATCTCTGGATGTGCT
ACCGAGCATTTATGAGGAAGGTG"/>
<sequence id="seq_Amel_Nuc_Apisto_301650"
taxon="Amel_Nuc_Apisto_301650" totalcount="4"
value="TGTTGGAGAAGCCAGAAGTGTTGCTTTAGTGGTAGTTGTATCACGGGAAG
TGACATTCATGCCCGCCCCACCTGCCGTCTCCCACCAGCATGTGATGGAGTTCGA
TGTGGAGGAAGATGACTCTTCTCGGTCACCGTCTCCTCAGGAGATTCTGCTGGAA
GTGGAGCTGGATGAAAACGAGGTCAAAGAGTTTGAGAAGCAGGTGAAGATCATC
ACCATACCCGAGTACACAGCTGACAACAAGAGCATGATCATATCTCTGGATGTGC
TACCGAGCATTTATGAGGAAGGTG"/>
<sequence id="seq_Amoa_Nuc_Apisto_302864"
taxon="Amoa_Nuc_Apisto_302864" totalcount="4"
value="TGTTGGAGAAGCCAGAAGTGTTGCTTTAGTGGTAGTTGTATCACGGGAAG
TGACATTCATGCCCGCCCCACCTGCCGTCTCCCACCAGCATGTGATGGAGTTCGA
TGTGGAGGAAGATGACTCTTCTCGGTCACCGTCTCCTCAGGAGATTCTGCTGGAA
GTGGAGCTGGATGAAAACGAGGTCAAAGAGTTTGAGAAGCAGGTGAAGATCATC
ACCATACCCGAGTACACAGCTGACAACAAGAGCATGATCATATCTCTGGATGTGC
TGCCGAGCATTTATGAGGAAGGTG"/>

<sequence id="seq_Amoa_Nuc_Apisto_302865"
taxon="Amoa_Nuc_Apisto_302865" totalcount="4"
value="TGTTGGAGAAGCCAGAAGTGTTGCTTTAGTGGTAGTTGTATCACGGGAAG
TGACATTCATGCCCGCCCCACCTGCCGTCTCCCACCAGCATGTGATGGAGTTCGA
TGTGGAGGAAGATGACTCTTCTCGGTCACCGTCTCCTCAGGAGATTCTGCTGGAA
GTGGAGCTGGATGAAAACGAGGTCAAAGAGTTTGAGAAGCAGGTGAAGATCATC
ACCATACCCGAGTACACAGCTGACAACAAGAGCATGATCATATCTCTGGATGTGC
TGCCGAGCATTTATGAGGAAGGTG"/>
<sequence id="seq_Amoa_Nuc_Apisto_302866"
taxon="Amoa_Nuc_Apisto_302866" totalcount="4"
value="TGTTGGAGAAGCCAGAAGTGTTGCTTTAGTGGTAGTTGTATCACGGGAAG
TGACATTCATGCCCGCCCCACCTGCCGTCTCCCACCAGCATGTGATGGAGTTCGA
TGTGGAGGAAGATGACTCTTCTCGGTCACCGTCTCCTCAGGAGATTCTGCTGGAA
GTGGAGCTGGATGAAAACGAGGTCAAAGAGTTTGAGAAGCAGGTGAAGATCATC
ACCATACCCGAGTACACAGCTGACAACAAGAGCATGATCATATCTCTGGATGTGC
TGCCGAGCATTTATGAGGAAGGTG"/>
<sequence id="seq_Amoa_Nuc_Apisto_302867"
taxon="Amoa_Nuc_Apisto_302867" totalcount="4"
value="TGTTGGAGAAGCCAGAAGTGTTGCTTTAGTGGTAGTTGTATCACGGGAAG
TGACATTCATGCCCGCCCCACCTGCCGTCTCCCACCAGCATGTGATGGAGTTCGA
TGTGGAGGAAGATGACTCTTCTCGGTCACCGTCTCCTCAGGAGATTCTGCTGGAA
GTGGAGCTGGATGAAAACGAGGTCAAAGAGTTTGAGAAGCAGGTGAAGATCATC
ACCATACCCGAGTACACAGCTGACAACAAGAGCATGATCATATCTCTGGATGTGC
TGCCGAGCATTTATGAGGAAGGTG"/>
<sequence id="seq_Amoa_Nuc_Apisto_302868"
taxon="Amoa_Nuc_Apisto_302868" totalcount="4"
value="TGTTGGAGAAGCCAGAAGTGTTGCTTTAGTGGTAGTTGTATCACGGGAAG
TGACATTCATGCCCGCCCCACCTGCCGTCTCCCACCAGCATGTGATGGAGTTCGA
TGTGGAGGAAGATGACTCTTCTCGGTCACCGTCTCCTCAGGAGATTCTGCTGGAA
GTGGAGCTGGATGAAAACGAGGTCAAAGAGTTTGAGAAGCAGGTGAAGATCATC
ACCATACCTGAGTACACAGCTGACAACAAGAGCATGATCATATCTCTGGATGTGC
TGCCGAGCATTTATGAGGAAGGTG"/>
<sequence id="seq_Amor_Nuc_Apisto_301601”
taxon="Amor_Nuc_Apisto_301601” totalcount="4"
value="TGtTGGAGAAGCCAGAAGTGTTGCTTTAGTGGTAGTTGTATCACGGGAAGT
GACATTCATGCCCGCCCCACCTGCCGTCTCCCACCAGCATGTGATGGAGTTCGAT
GTGGAGGAAGATGACTCTTCTCGGTCACCGTCTCCTCAGGAGATTcTGCTGGAAG
TGGAGCTGGATGAAAACGAGGTCAAAGAGTTTGAGAAGCAGGTGAAGATCATCA
CCATACCCGAGTACACAGCTGACAACAAGAGCATGATCATATCTCTGGATGTGCT
ACCGAGCATTTATGAGGAAGGTG"/>
<sequence id="seq_Amor_Nuc_Apisto_302772"
taxon="Amor_Nuc_Apisto_302772" totalcount="4"
value="TGTTGGAGAAGCCAGAAGTGTTGCTTTAGTGGTAGTTGTATCACGGGAAG
TGACATTCATGCCCGCCCCACCTGCCGTCTCCCACCAGCATGTGATGGAGTTCGA
TGTGGAGGAAGATGACTCTTCTCGGTCACCGTCTCCTCAGGAGATTCTGCTGGAA
GTGGAGCTGGATGAAAACGAGGTCAAAGAGTTTGAGAAGCAGGTGAAGATCATC
ACCATACCCGAGTACACAGCTGACAACAAGAGCATGATCATATCTCTGGATGTGC
TACCGAGCATTTATGAGGAAGGTG"/>
<sequence id="seq_Amor_Nuc_Apisto_302773"
taxon="Amor_Nuc_Apisto_302773" totalcount="4"

value="TGTTGGAGAAGCCAGAAGTGTTGCTTTAGTGGTAGTTGTATCACGGGAAG
TGACATTCATGCCCGCCCCACCTGCCGTCTCCCACCAGCATGTGATGGAGTTCGA
TGTGGAGGAAGATGACTCTTCTCGGTCACCGTCTCCTCAGGAGATTcTGCTGGAA
GTGGAGCTGGATGAAAACGAGGTCAAAGAGTTTGAGAAGCAGGTGAAGATCATC
ACCATACCCGAGTACACAGCTGACAACAAGAGCATGATCATATCTCTGGATGTGC
TACCGAGCATTTATGAGGAAGGTG"/>
<sequence id="seq_Amor_Nuc_Apisto_302775"
taxon="Amor_Nuc_Apisto_302775" totalcount="4"
value="TGTTGGAGAAGCCAGAAGTGTTGCTTTAGTGGTAGTTGTATCACGGGAAG
TGACATTCATGCCCGCCCCACCTGCCGTCTCCCACCAGCATGTGATGGAGTTCGA
TGTGGAGGAAGATGACTCTTCTCGGTCACCGTCTCCTCAGGAGATTcTGCTGGAA
GTGGAGCTGGATGAAAACGAGGTCAAAGAGTTTGAGAAGCAGGTGAAGATCATC
ACCATACCCGAGTACACAGCTGACAACAAGAGCATGATCATATCTCTggATGTGC
TACCGAGCATTTATGAGGAAGGTG"/>
<sequence id="seq_Amor_Nuc_Apisto_302776"
taxon="Amor_Nuc_Apisto_302776" totalcount="4"
value="TGTTGGAGAAGCCAGAAGTGTTGCTTTAGTGGTAGTTGTATCACGGGAAG
TGACATTCATGCCCGCCCCACCTGCCGTCTCCCACCAGCATGTGATGGAGTTCGA
TGTGGAGGAAGATGACTCTTCTCGGTCACCGTCTCCTCAGGAGATTcTGCTGGAA
GTGGAGCTgGATGAaAACGAGGTCAAAGAGTTTGAGAAGCAGGTGAAGATCATC
ACCATACCCGAGTACACAGCTGACAACAAGAGCATGATCATATCTCTGGATGTGC
TACCGAGCATTTATGAGGAAGGTG"/>
<sequence id="seq_Amor_Nuc_Apisto_302816"
taxon="Amor_Nuc_Apisto_302816" totalcount="4"
value="TGTTGGAGAAGCCAGAAGTGTTGCTTTAGTGGTAGTTGTATCACGGGAAG
TGACATTCATGCCCGCCCCACCTGCCGTCTCCCACCAGCATGTGATGGAgtTcGAT
GTGGAGGAAGATGACTCTTCTCGGTCACCGTCTCCTCAGGAGATTCTGCTGGAAG
TGGAGCTGGATGAAAAcGaGGTCAAAGAGTTTGAGAAGCAGGTGAAGATCATCAC
CaTaCCCGaGTACACAGCTGACAACAAGAGCATGATCATATCTCTGGATGTGCTAC
CGAGCATTTATGAGGAAGGTG"/>
<sequence id="seq_Amor_Nuc_Apisto_302817"
taxon="Amor_Nuc_Apisto_302817" totalcount="4"
value="TGTTGGAGAAGCCAGAAGTGTTGCTTTAGTGGTAGTTGTATCACGGGAAG
TGACATTCATGCCCGCCCCACCTGCCGTCTCCCACCAGCATGTGATGGAGTTCGA
TGTGGAGGAAGATGACTCTTCTCGGTCACCGTCTCCTCAGGAGATTcTGCTGGAA
GTGGAGCTGGATGAAAACGAGGTCAAAGAGTTTGAGAAGCAGGTGAAGATCATC
ACCATACCCGAGTACACAGCTGACAACAAGAGCATGATCATATCTCTggATGTGC
TACCGAGCATTTATGAGGAAGGTG"/>
<sequence id="seq_Amor_Nuc_Apisto_302818"
taxon="Amor_Nuc_Apisto_302818" totalcount="4"
value="TGTTGGAGAAGCCAGAAGTGTTGCTTTAGTGGTAGTTGTATCACGGGAAG
TGACATTCATGCCCGCCCCACCTGCCGTCTCCCACCAGCATGTGATGGAGTTCGA
TGTGGAGGAAGATGACTCTTCTCGGTCACCGTCTCCTCAGGAGATTcTGCTGGAA
GTGGAGCTGGATGAAAACGAGGTCAAAGAGTTTGAGAAGCAGGTGAAGATCATC
ACCATACCCGAGTACACAGCTGACAACAAGAGCATGATCATATCTCTGGATGTGC
TACCGAGCATTTATGAGGAAGGTG"/>
<sequence id="seq_Amor_Nuc_Apisto_302819"
taxon="Amor_Nuc_Apisto_302819" totalcount="4"
value="TGTTGGAGAAGCCAGAAGTGTTGCTTTAGTGGTAGTTGTATCACGGGAAG
TGACATTCATGCCCGCCCCACCTGCCGTCTCCCACCAGCATGTGATGGAGTTCGA

TGTGGAGGAAGATGACTCTTCTCGGTCACCGTCTCCTCAGGAGATTcTGCTGGAA
GTGGAGCTGGATGAAAACGAGGTCAAAGAGTTTGAGAAGCAGGTGAAGATCATC
ACCATACCCGAGTACACAGCTGACAACAAGAGCATGATCATATCTCTGGATGTGC
TACCGAGCATTTATGAGGAAGGTG"/>
<sequence id="seq_Amor_Nuc_Apisto_300564"
taxon="Amor_Nuc_Apisto_300564" totalcount="4"
value="TGTTGGAGAAGCCAGAAGTGTTGCTTTAGTGGTAGTTGTATCACGGGAAG
TGACATTCATGCCCGCCCCACCTGCCGTCTCCCACCAGCaTGTGATGGAGTTCGAT
GTGGAGGAAGATGACTCTTCTCGGTCACCGTCTCCTCAGGAGATTCTGCTGGAAG
TGGAGCTGGATGAAAACGAGGTcaAAGAGtTTGAGAAGCAGGTgAAGATcaTCACca
TACCCGAgTACaCAGCTgACAACaAGAgCATgATCATATCTCTGGATGTGCTACCGA
GCATTTATGAGGAAGGTG"/>
<sequence id="seq_Amor_Nuc_Apisto_300577"
taxon="Amor_Nuc_Apisto_300577" totalcount="4"
value="TGTTGGAGAAGCCAGAAGTGTTGCTTTAGTGGTAGTTGTATCACGGGAAG
TGACATTCATGCCCGCCCCACCTGCCGTCTCCCACCAGCATGTGATGGAGTTCGA
TGTGGAGGAAGATGACTCTTCTCGGTCACCGTCTCCTCAGGAGATTCTGCTGGAA
GTGGAGCTGGATGAAAACGAGGTCAAAGAGTTTGAGAAGCAGGTGAAGATCATC
ACCATACCCGAGTACACAGCTGAcAACAAGAGCAtGATCATATCTCTGGATGTGCT
ACCGAGCATTTATGAGGAAGGTG"/>
<sequence id="seq_Amor_Nuc_Apisto_300578"
taxon="Amor_Nuc_Apisto_300578" totalcount="4"
value="TGTTGGAGAAGCCAGAAGTGTTGCTTTAGTGGTAGTTGTATCACGGGAAG
TGACATTCATGCCCGCCCCACCTGCCGTCTCCCACCAGCATGTGATGGAGTTCGA
TGTGGAGGAAGATGACTCTTCTCGGTCACCGTCTCCTCAGGAGATTCTGCTGGAA
GTGGAGCTGGATGAAAACGAGGTCAAAGAGTTTGAGAAGCAGGTGAAGATCATC
ACCATACCCGAGTACACAGCTGACAACAAGAGCATGATCATATCTCTGGATGTGC
TACCGAGCATTTATGAGGAAGGTG"/>
<sequence id="seq_Amor_Nuc_Apisto_300581"
taxon="Amor_Nuc_Apisto_300581" totalcount="4"
value="TGTTGGAGAAGCCAGAAGTGTTGCTTTAGTGGTAGTTGTATCACGGGAAG
TGACATTCATGCCCGCCCCACCTGCCGTCTCCCACCAGCATGTGATGGAGTTCGA
TGTGGAGGAAGATGACTCTTCTCGGTCACCGTCTCCTCAGGAGATTCTGCTGGAA
GTGGAGCTGGATGAAAACGAGGTCAAAGAGTTTGAGAAGCAGGTGAAGATCATC
ACCATACCCGAGTACACAGCTGACAACAAGAGCATGATCATATCTCTGGATGTGC
TACCGAGCATTTATGAGGAAGGTG"/>
<sequence id="seq_Amor_Nuc_Apisto_301724"
taxon="Amor_Nuc_Apisto_301724" totalcount="4"
value="TGTTGGAGAAGCCAGAAGTGTTGCTTTAGTGGTAGTTGTATCACGGGAAG
TGACATTCATGCCCGCCCCACCTGCCGTCTCCCACCAGCATGTGATGGAGTTCGA
TGTGGAGGAAGATGACTCTTCTCGGTCACCGTCTCCTCAGGAGATTCTGCTGGAA
GTGGAGCTGGATGAAAACGAGGTCAAAGAGTTTGAGAAGCAGGTGAAGATCATC
ACCATACCCGAGTACACAGCTGACAACAAGAGCATGATCATATCTCTGGATGTGC
TACCGAGCATTTATGAGGAAGGTG"/>
<sequence id="seq_Amor_Nuc_Apisto_301726"
taxon="Amor_Nuc_Apisto_301726" totalcount="4"
value="TGTTGGAGAAGCCAGAAGTGTTGCTTTAGTGGTAGTTGTATCACGGGAAG
TGACATTCATGCCCGCCCCACCTGCCGTCTCCCACCAGCATGTGATGGAGTTCGA
TGTGGAGGAAGATGACTCTTCTCGGTCACCGTCTCCTCAGGAGATTCTGCTGGAA
GTGGAGCTGGATGAAAACGAGGTCAAAGAGTTTGAGAAGCAGGTGAAGATCATC

ACCATACCCGAGTACACAGCTGACAACAAGAGCATGATCATATCTCTGGATGTGC
TACCGAGCATTTATGAGGAAGGTG"/>
<sequence id="seq_Amor_Nuc_Apisto_301727"
taxon="Amor_Nuc_Apisto_301727" totalcount="4"
value="TGTTGGAGAAGCCAGAAGTGTTGCTTTAGTGGTAGTTGTATCACGGGAAG
TGACATTCATGCCCGCCCCACCTGCCGTCTCCCACCAGCATGTGATGGAGTTCGA
TGTGGAGGAAGATGACTCTTCTCGGTCACCGTCTCCTCAGGAGATTcTGCTGGAA
GTGGAGCTGGATGAAAACGAGGTCAAAGAGTTTGAGAAGCAGGTGAAGATCATC
ACCATACCCGAGTACACAGCTGACAACAAGAGCATGATCATATCTCTGGATGTGC
TACCGAGCATTTATGAGGAAGGTG"/>
<sequence id="seq_Amor_Nuc_Apisto_301730"
taxon="Amor_Nuc_Apisto_301730" totalcount="4"
value="TGTTGGAGAAGCCAGAAGTGTTGCTTTAGTGGTAGTTGTATCACGGGAAG
TGACATTCATGCCCGCCCCACCTGCCGTCTCCCACCAGCATGTGATGGAGTTCGA
TGTGGAGGAAGATGACTcTTCTCGGTCACCGTCTCCTCAGGAGATTcTGCTGGAAG
TGGAGCTGGATGAAAACGAGGTCAAAGAGTTTGAGAAGCAGGTGAAGATCATCA
CCATACCCGAGTACACAGCTGACAACAAGAGCATGATCATATCTCTGGATGTGCT
ACCGAGCATTTATGAGGAAGGTG"/>
<sequence id="seq_Amor_Nuc_Apisto_301792"
taxon="Amor_Nuc_Apisto_301792" totalcount="4"
value="TGTTGGAGAAGCCAGAAGTGTTGCTTTAGTGGTAGTTGTATCACGGGAAG
TGACATTCATGCCCGCCCCACCTGCCGTCTCCCACCAGCATGTGATGGAGTTCGA
TGTGGAGGAAGATGACTcTTCTCGGTCACCGTCTCCTCAGGAGATTcTGCTGGAAG
TGGAGCTgGATGAaAACGAGGTCAAAGAGTTTGAGAAGCAGGTGAAGATCATCAC
CATAcCCGAGTACACAGCTGACAACAAGAGCATGATCATATCTCTGGATGTGCTA
CCGAGCATTTATGAGGAAGGTG"/>
<sequence id="seq_Amor_Nuc_Apisto_301599”
taxon="Amor_Nuc_Apisto_301599” totalcount="4"
value="TGTTGGAGAAGCCAGAAGTGTTGCTTTAGTGGTAGTTGTATCACGGGAAG
TGACATTCATGCCCGCCCCACCTGCCGTCTCCCACCAGCATGTGATGGAGTTCGA
TGTGGAGGAAGATGACTCTTCTCGGTCACCGTCTCCTCAGGAGATTCTGCTGGAA
GTGGAGCTGGATGAAAACGAGGTCAAAGAGTTTGAGAAGCAGGTGAAGATCATC
ACCATACCCGAGTACACAGCTGACAACAAGAGCATGATCATATCTCTGGATGTGC
TACCGAGCATTTATGAGGAAGGTG"/>
<sequence id="seq_Amor_Nuc_Apisto_301600”
taxon="Amor_Nuc_Apisto_301600” totalcount="4"
value="TGTTGGAGAAGCCAGAAGTGTTGCTTTAGTGGTAGTTGTATCACGGGAAG
TGACATTCATGCCCGCCCCACCTGCCGTCTCCCACCAGCATGTGATGGAGTTCGA
TGTGGAGGAAGATGACTCTTCTCGGTCACCGTCTCCTCAGGAGATTCTGCTGGAA
GTGGAGCTGGATGAAAACGAGGTCAAAGAGTTTGAGAAGCAGGTGAAGATCATC
ACCATACCCGAGTACACAGCTGACAACAAGAGCATGATCATATCTCTGGATGTGC
TACCGAGCATTTATGAGGAAGGTG"/>
<sequence id="seq_Anij_Nuc_Apisto_300304"
taxon="Anij_Nuc_Apisto_300304" totalcount="4"
value="TGTTGGAGAAGCCAGAAGTGTTGCTTTAGTGGTGGTTGTATCACGGGAAG
TGACGTTCATGCCCGCCCCACCTGCCGTCTCCCATCAGCATGTGATGGAGTTTGAT
GTGGAGGAGGATGACTCCTCTCGGTCACCATCTCCTCAGGAGATTCTGCTCGAGG
TGGAGCTGGATGAAAACGAAGTCAAAGAGTTTgAGAAGCAGGtgAAGATCAtCAC
CATACCtGAgTAcACGgCTgACAAcAAGAgCATGATCATATCTCTGGACGTGCTGCC
GAGCATTTATGAGGAAGGTG"/>

<sequence id="seq_Anij_Nuc_Apisto_300306"
taxon="Anij_Nuc_Apisto_300306" totalcount="4"
value="TGTTGGAGAAGCCAGAAGTGTTGCTTTAGTGGTGGTTGTATCACGGGAAG
TGACGTTCATGCCCGCCCCACCTGCCGTCTCCCaTCAGCATGTGATGGAGTTTGAT
GTGGAGGAGGATGACTCCTCTCGGTCACCaTCTCCTCaGGAGATTCTGCTCGAGGT
GGAGCTGGATGAAAACGAAGTCAAAGAGTTTGAGAAGCAGGTGAAGATCATCAC
CATACCTGAGTACACGGCTGACAACAAGAGCATGATCATATCTCTGGACGTGCTG
CCGAGCATTTATGAGGAAGGTG"/>
<sequence id="seq_Anij_Nuc_Apisto_300328"
taxon="Anij_Nuc_Apisto_300328" totalcount="4"
value="TGTTGGAGAAGCCAGAAGTGTTGCTTTAGTGGTGGTTGTATCACGGGAAG
TGACGTTCATGCCCGCCCCACCTGCCGTCTCCCATCAGCATGTGATGGAGTTTGAT
GTGGAGGAGGATGACTCCTCTCGGTCACCATCTCCTCAGGAGATTCTGCTCGAGG
TGGAGCTGGATGAAAACGAAGTCAAAGAGTTTGAGAAGCAGGTGAAGATCATCA
CCATACCTGAGTACACGGCTGACAACAAGAGCATGATCATATCTCTGGACGTGCT
GCCGAGCATTTATGAGGAAGGTG"/>
<sequence id="seq_Aore_Nuc_Apisto_302891"
taxon="Aore_Nuc_Apisto_302891" totalcount="4"
value="TGTTGGAGAAGCCAGAAGTGTTGCTTTAGTGGTGGTTGTATCACGGGAAG
TGACGTTCATGCCCGCCCCACCTGCCGTCTCCCATCAGCATGTGATGGAGTTTGAT
GTGGAGGAGGATGACTCCTCTCGGTCACCATCTCCTCAGGAGATTCTGCTCGAGG
TGGAGCTGGATGAAAACGAAGTCAAAGAGTTTGAGAAGCAGGTGAAGATCATCA
CCATACCCGAGTACACGGCTGACAACAAGAGCATGATCATATCTCTGGACGTGCT
GCCGAGCATTTATGAGGAAGGTG"/>
<sequence id="seq_Aore_Nuc_Apisto_302892"
taxon="Aore_Nuc_Apisto_302892" totalcount="4"
value="TGTTGGAGAAGCCAGAAGTGTTGCTTTAGTGGTGGTTGTATCACGGGAAG
TGACGTTCATGCCCGCCCCACCTGCCGTCTCCCATCAGCATGTGATGGAGTTTGAT
GTGGAGGAGGATGACTCCTCTCGGTCACCATCTCCTCAGGAGATTCTGCTCGAGG
TGGAGCTGGATGAAAACGAAGTCAAAGAGTTTGAGAAGCAGGTGAAGATCATCA
CCATACCCGAGTACACGGCTGACAACAAGAGCATGATCATATCTCTGGACGTGCT
GCCGAGCATTTATGAGGAAGGTG"/>
<sequence id="seq_Aore_Nuc_Apisto_302893"
taxon="Aore_Nuc_Apisto_302893" totalcount="4"
value="TGTTGGAGAAGCCAGAAGTGTTGCTTTAGTGGTGGTTGTATCACGGGAAG
TGACGTTCATGCCCGCCCCACCTGCCGTCTCCCATCAGCATGTGATGGAGTTTGAT
GTGGAGGAGGATGACTCCTCTCGGTCACCATCTCCTCAGGAGATTCTGCTCGAGG
TGGAGCTGGATGAAAACGAAGTCAAAGAGTTTGAGAAGCAGGTGAAGATCATCA
CCATACCCGAGTACACGGCTGACAACAAGAGCATGATCATATCTCTGGACGTGCT
GCCGAGCATTTATGAGGAAGGTG"/>
<sequence id="seq_Aore_Nuc_Apisto_302894"
taxon="Aore_Nuc_Apisto_302894" totalcount="4"
value="TGTTGGAGAAGCCAGAAGTGTTGCTTTAGTGGTGGTTGTATCACGGGAAG
TGACGTTCATGCCCGCCCCACCTGCCGTCTCCCATCAGCATGTGATGGAGTTTGAT
GTGGAGGAGGATGACTCCTCTCGGTCACCATCTCCTCAGGAGATTCTGCTCGAGG
TGGAGCTGGATGAAAACGAAGTCAAAGAGTTTGAGAAGCAGGTGAAGATCATCA
CCATACCCGAGTACACGGCTGACAACAAGAGCATGATCATATCTCTGGACGTGCT
GCCGAGCATTTATGAGGAAGGTG"/>
<sequence id="seq_Aore_Nuc_Apisto_302895"
taxon="Aore_Nuc_Apisto_302895" totalcount="4"

value="TGTTGGAGAAGCCAGAAGTGTTGCTTTAGTGGTGGTTGTATCACGGGAAG
TGACGTTCATGCCCGCCCCACCTGCCGTCTCCCATCAGCATGTGATGGAGTTTGAT
GTGGAGGAGGATGACTCCTCTCGGTCACCATCTCCTCAGGAGATTCTGCTCGAGG
TGGAGCTGGATGAAAACGAAGTCAAAGAGTTTGAGAAGCAGGTGAAGATCATCA
CCATACCCGAGTACACGGCTGACAACAAGAGCATGATCATATCTCTGGACGTGCT
GCCGAGCATTTATGAGGAAGGTG"/>
<sequence id="seq_Apap_Nuc_Apisto_301773"
taxon="Apap_Nuc_Apisto_301773" totalcount="4"
value="TGTTGGAGAAGCCAGAAGTGTTGCTTTAGTGGTAGTTGTATCACGGGAAG
TGACATTCATGCCCGCCCCACCTGCCGTCTCCCACCAGCATGTGATGGAGTTCGA
TGTGGAGGAAGATGACTCTTCTCGGTCACCGTCTCCTCAGGAGATTCTGCTGGAA
GTGGAGCTGGATGAAAACGAGGTCAAAGAGTTTGAGAAGCAGGTGAAGATCATC
ACCATACCCGAGTACACAGCTGACAACAAGAGCATGATCATATCTCTGGATGTGC
TACCGAGCATTTATGAGGAAGGTG"/>
<sequence id="seq_Apap_Nuc_Apisto_301774"
taxon="Apap_Nuc_Apisto_301774" totalcount="4"
value="TGTTGGAGAAGCCAGAAGTGTTGCTTTAGTGGTAGTTGTATCACGGGAAG
TGACATTCATGCCCGCCCCACCTGCCGTCTCCCACCAGCATGTGATGGAGTTCGA
TGTGGAGGAAGATGACTCTTCTCGGTCACCGTCTCCTCAGGAGATTCTGCTGGAA
GTGGAGCTGGATGAAAACGAGGTCAAAGAGTTTGAGAAGCAGGTGAAGATCATC
ACCATACCCGAGTACACAGCTGACAACAAGAGCATGATCATATCTCTGGATGTGC
TACCGAGCATTTATGAGGAAGGTG"/>
<sequence id="seq_Apap_Nuc_Apisto_301766"
taxon="Apap_Nuc_Apisto_301766" totalcount="4"
value="TGTTGGAGAAGCCAGAAGTGTTGCTTTAGTGGTAGTTGTATCACGGGAAG
TGACATTCATGCCCGCCCCACCTGCCGTCTCCCACCAGCATGTGATGGAGTTCGA
TGTGGAGGAAGATGACTCTTCTCGGTCACCGTCTCCTCAGGAGATTCTGCTGGAA
GTGGAGCTGGATGAAAACGAGGTCAAAGAGTTTGAGAAGCAGGTGAAGATCATC
ACCATACCCGAGTACACAGCTGACAACAAGAGCATGATCATATCTCTGGATGTGC
TACCGAGCATTTATGAGGAAGGTG"/>
<sequence id="seq_Apap_Nuc_Apisto_301768"
taxon="Apap_Nuc_Apisto_301768" totalcount="4"
value="TGTTGGAGAAGCCAGAAGTGTTGCTTTAGTGGTAGTTGTATCACGGGAAG
TGACATTCATGCCCGCCCCACCTGCCGTCTCCCACCAGCATGTGATGGAGTTCGA
TGTGGAGGAAGATGACTCTTCTCGGTCACCGTCTCCTCAGGAGATTcTGCTGGAA
GTGGAGCTGGATGAAAACGAGGTCAAAGAGTTTGAGAAGCAGGTGAAGATCATC
ACCATACCCGAGTACACAGCTGACAACAAGAGCATGATCATATCTCTgGATGTGC
TACCGAGCATTTATGAGGAAGGTG"/>
<sequence id="seq_Apap_Nuc_Apisto_301769"
taxon="Apap_Nuc_Apisto_301769" totalcount="4"
value="TGTTGGAGAAGCCAGAAGTGTTGCTTTAGTGGTAGTTGTATCACGGGAAG
TGACATTCATGCCCGCCCCACCTGCCGTCTCCCACCAGCATGTGATGGAGTTCGA
TGTGGAGGAAGATGACTCTTCTCGGTCACCGTCTCCTCAGGAGATTCTGCTGGAA
GTGGAGCTGGATGAAAACGAGGTCAAAGAGTTTGAGAAGCAGGTGAAGATCATC
ACCATACCCGAGTACACAGCTGACAACAAGAGCATGATCATATCTCTgGATGTGC
TACCGAGCATTTATGAGGAAGGTG"/>
<sequence id="seq_Apap_Nuc_Apisto_301770"
taxon="Apap_Nuc_Apisto_301770" totalcount="4"
value="TGTTGGAGAAGCCAGAAGTGTTGCTTTAGTGGTAGTTGTATCACGGGAAG
TGACATTCATGCCCGCCCCACCTGCCGTCTCCCACCAGCATGTGATGGAGTTCGA

TGTGGAGGAAGATGACTCTTCTCGGTCACCGTCTCCTCAGGAGATTcTGCTGGAA
GTGGAGCTGGATGAAAACGAGGTCAAAGAGTTTGAGAAGCAGGTGAAGATCATC
ACCATACCCGAGTACACAGCTGACAACAAGAGCATGATCATATCTCTGGATGTGC
TACCGAGCATTTATGAGGAAGGTG"/>
<sequence id="seq_Apaul_Nuc_Apisto_300387"
taxon="Apaul_Nuc_Apisto_300387" totalcount="4"
value="TGTTGGAGAAGCCAGAAGTGTTGCTTTAGTGGTAGTTGTATCACGGGAAG
TGACATTCATGCCCGCCCCACCTGCCGTCTCCCATCAGCATGTGATGGAGTTCGA
TGTGGAGGAAGATGACTCTTCTCGGTCACCGTCTCCTCAGGAGATTCTGCTGGAA
GTGGAGCTGGATGAAAACGAGGTCAAAGAGTTTGAGAAGCAGGTGAAGATCATC
ACCATACCCGAGTACACAGCTGACAACAAGAGCATGATCATATCTCTGGATGTGC
TACCGAGCATTTATGAGGAAGGTG"/>
<sequence id="seq_Apaul_Nuc_Apisto_300391"
taxon="Apaul_Nuc_Apisto_300391" totalcount="4"
value="TGTTGGAGAAGCCAGAAGTGTTGCTTTAGTGGTAGTTGTATCACGGGAAG
TGACATTCATGCCCGCCCCACCTGCCGTCTCCCATCAGCATGTGATGGAGTTCGA
TGTGGAGGAAGATGACTCTTCTCGGTCACCGTCTCCTCAGGAGATTCTGCTGGAA
GTGGAGCTGGATGAAAACGAGGTCAAAGAGTTTGAGAAGCAGGTGAAGATCATC
ACCATACCCGAGTACACAGCTGACAACAAGAGCATGATCATATCTCTGGATGTGC
TACCGAGCATTTATGAGGAAGGTG"/>
<sequence id="seq_Apaul_Nuc_Apisto_300393"
taxon="Apaul_Nuc_Apisto_300393" totalcount="4"
value="TGTTGGAGAAGCCAGAAGTGTTGCTTTAGTGGTAGTTGTATCACGGGAAG
TGACATTCATGCCCGCCCCACCTGCCGTCTCCCATCAGCATGTGATGGAGTTCGA
TGTGGAGGAAGATGACTCTTCTCGGTCACCGTCTCCTCAGGAGATTCTGCTGGAA
GTGGAGCTGGATGAAAACGAGGTCAAAGAGTTTGAGAAGCAGGTGAAGATCATC
ACCATACCCGAGTACACAGCTGACAACAAGAGCATGATCATATCTCTgGATGTGC
TACCGAGCATTTATGAGGAAGGTG"/>
<sequence id="seq_Apaul_Nuc_Apisto_302881"
taxon="Apaul_Nuc_Apisto_302881" totalcount="4"
value="TGTTGGAGAAGCCAGAAGTGTTGCTTTAGTGGTAGTTGTATCACGGGAAG
TGACATTCATGCCCGCCCCACCTGCCGTCTCCCATCAGCATGTGATGGAGTTCGA
TGTGGAGGAAGATGACTCTTCTCGGTCACCGTCTCCTCAGGAGATTCTGCTGGAA
GTGGAGCTGGATGAAAACGAGGTCAAAGAGTTTGAGAAGCAGGTGAAGATCATC
ACCATACCCGAGTACACAGCTGACAACAAGAGCATGATCATATCTCTGGATGTGC
TACCGAGCATTTATGAGGAAGGTG"/>
<sequence id="seq_Apaul_Nuc_Apisto_302882"
taxon="Apaul_Nuc_Apisto_302882" totalcount="4"
value="TGTTGGAGAAGCCAGAAGTGTTGCTTTAGTGGTAGTTGTATCACGGGAAG
TGACATTCATGCCCGCCCCACCTGCCGTCTCCCATCAGCATGTGATGGAGTTCGA
TGTGGAGGAAGATGACTCTTCTCGGTCACCGTCTCCTCAGGAGATTCTGCTGGAA
GTGGAGCTGGATGAAAACGAGGTCAAAGAGTTTGAGAAGCAGGTGAAGATCATC
ACCATACCCGAGTACACAGCTGACAACAAGAGCATGATCATATCTCTGGATGTGC
TACCGAGCATTTATGAGGAAGGTG"/>
<sequence id="seq_Apaul_Nuc_Apisto_302883"
taxon="Apaul_Nuc_Apisto_302883" totalcount="4"
value="TGTTGGAGAAGCCAGAAGTGTTGCTTTAGTGGTAGTTGTATCACGGGAAG
TGACATTCATGCCCGCCCCACCTGCCGTCTCCCATCAGCATGTGATGGAGTTCGA
TGTGGAGGAAGATGACTCTTCTCGGTCACCGTCTCCTCAGGAGATTCTGCTGGAA
GTGGAGCTGGATGAAAACGAGGTCAAAGAGTTTGAGAAGCAGGTGAAGATCATC

ACCATACCCGAGTACACAGCTGACAACAAGAGCATGATCATATCTCTGGATGTGC
TACCGAGCATTTATGAGGAAGGTG"/>
<sequence id="seq_Apaul_Nuc_Apisto_302885"
taxon="Apaul_Nuc_Apisto_302885" totalcount="4"
value="TGTTGGAGAAGCCAGAAGTGTTGCTTTAGTGGTAGTTGTATCACGGGAAG
TGACATTCATGCCCGCCCCACCTGCCGTCTCCCATCAGCATGTGATGGAGTTCGA
TGTGGAGGAAGATGACTCTTCTCGGTCACCGTCTCCTCAGGAGATTCTGCTGGAA
GTGGAGCTGGATGAAAACGAGGTCAAAGAGTTTGAGAAGCAGGTGAAGATCATC
ACCATACCCGAGTACACAGCTGACAACAAGAGCATGATCATATCTCTGGATGTGC
TACCGAGCATTTATGAGGAAGGTG"/>
<sequence id="seq_Apeb_Nuc_Apisto_302561"
taxon="Apeb_Nuc_Apisto_302561" totalcount="4"
value="TGTTGGAGAAGCCAGAAGTGTTGCTTTAGTGGTAGTTGTATCACGGGAAG
TGACATTCATGCCCGCCCCACCTGCCGTCTCCCACCAGCATGTGATGGAGTTCGA
TGTGGAGGAAGATGACTCTTCTCGGTCACCGTCTCCTCAGGAGATTCTGCTGGAA
GTGGAGCTGGATGAAAACGAGGTCAAAGAGTTTGAGAAGCAGGTGAAGATCATC
ACCATACCCGAGTACACAGCTGACAACAAGAGCATGATCATATCTCTGGATGTGC
TACCGAGCATTTATGAGGAAGGTG"/>
<sequence id="seq_Apeb_Nuc_Apisto_302562"
taxon="Apeb_Nuc_Apisto_302562" totalcount="4"
value="TGTTGGAGAAGCCAGAAGTGTTGCTTTAGTGGTAGTTGTATCACGGGAAG
TGACATTCATGCCCGCCCCACCTGCCGTCTCCCACCAGCATGTGATGGAGTTCGA
TGTGGAGGAAGATGACTCTTCTCGGTCACCGTCTCCTCAGGAGATTcTGCTGGAA
GTGGAGCTGGATGAAAACGAGGTCAAAGAGTTTGAGAAGCAGGTGAAGATCATC
ACCATACCCGAGTACACAGCTGACAACAAGAGCATGATCATATCTCTGGATGTGC
TACCGAGCATTTATGAGGAAGGTG"/>
<sequence id="seq_Apeb_Nuc_Apisto_302563"
taxon="Apeb_Nuc_Apisto_302563" totalcount="4"
value="TGTTGGAGAAGCCAGAAGTGTTGCTTTAGTGGTAGTTGTATCACGGGAAG
TGACATTCATGCCCGCCCCACCTGCCGTCTCCCACCAGCATGTGATGGAGTTCGA
TGTGGAGGAAGATGACTCTTCTCGGTCACCGTCTCCTCAGGAGATTcTGCTGGAA
GTGGAGCTGGATGAAAACGAGGTCAAAGAGTTTGAGAAGCAGGTGAAGATCATC
ACCATACCCGAGTACACAGCTGACAACAAGAGCATGATCATATCTCTGGATGTGC
TACCGAGCATTTATGAGGAAGGTG"/>
<sequence id="seq_Apeb_Nuc_Apisto_302564"
taxon="Apeb_Nuc_Apisto_302564" totalcount="4"
value="TGTTGGAGAAGCCAGAAGTGTTGCTTTAGTGGTAGTTGTATCACGGGAAG
TGACATTCATGCCCGCCCCACCTGCCGTCTCCCACCAGCATGTGATGGAGTTCGA
TGTGGAGGAAGATGACTCTTCTCGGTCACCGTCTCCTCAGGAGATTcTGCTGGAA
GTGGAGCTGGATGAAAACGAGGTCAAAGAGTTTGAGAAGCAGGTGAAGATCATC
ACCATACCCGAGTACACAGCTGACAACAAGAGCATGATCATATCTCTGGATGTGC
TAcCGAGCATTTATGAGGAAGGTG"/>
<sequence id="seq_Apeb_Nuc_Apisto_302565"
taxon="Apeb_Nuc_Apisto_302565" totalcount="4"
value="TGTTGGAGAAGCCAGAAGTGTTGCTTTAGTGGTAGTTGTATCACGGGAAG
TGACATTCATGCCCGCCCCACCTGCCGTCTCCCACCAGCATGTGATGGAGTTCGA
TGTGGAGGAAGATGACTCTTCTCGGTCACCGTCTCCTCAGGAGATTcTGCTGGAA
GTGGAGCTGGATGAAAACGAGGTCAAAGAGTTTGAGAAGCAGGTGAAGATCATC
ACCATACCCGAGTACACAGCTGACAACAAGAGCATGATCATATCTCTGGATGTGC
TACCGAGCATTTATGAGGAAGGTG"/>

<sequence id="seq_Aper_Nuc_Apisto_301543"
taxon="Aper_Nuc_Apisto_301543" totalcount="4"
value="TGTTGGAGAAGCCAGAAGTGTTGCTTTAGTGGTaGTTGTATCACGGGAAGT
GACaTTCATGCCCGCCCCACCTGCCGTCTCCCATCAGCATGTGATGGAGTTcGATG
TGGAGGAaGATGACTCtTCTCGGTCACCgTCTCCTCAGGAGATTCTGCTGGAAGTG
GAGCTGGATGAAAACGAgGTCAAAGAGTTTGAGAAGCAGGTGAAGATCATCACC
ATACCCGAGTACACaGCTGACAACAAGAGCATGATCATATCTCTGGAtGTGCTaCC
GAGCATTTATGAGGAAGGTG"/>
<sequence id="seq_Aros_Nuc_Apisto_301032”
taxon="Aros_Nuc_Apisto_301032” totalcount="4"
value="TGTTGGAGAAGCCAGAAGTGTTGCTTTAGTGGTGGTTGTATCACGGGAAG
TGACGTTCATGCCCGCCCCACCTGCCGTCTCCCATCAGCATGTGATGGAGTTTGAT
GTGGAGGAGGATGACTCCTCTCGGTCACCATCTCCTCAGGAGATTCTGCTCGAGG
TGGAGCTGGATGAAAACGAAGTCAAAGAGTTTGAGAAGCAGGTGAAGATCATCA
CCATACCCGAGTACACGGCTGACAACAAGAGCATGATCATATCTCTGGACGTGCT
GCCGAGCATTTATGAGGAAGGTG"/>
<sequence id="seq_Aros_Nuc_Apisto_301034”
taxon="Aros_Nuc_Apisto_301034” totalcount="4"
value="TGTTGGAGAAGCCAGAAGTGTTGCTTTAGTGGTGGTTGTATCACGGGAAG
TGACGTTCATGCCCGCCCCACCTGCCGTCTCCCATCAGCATGTGATGGAGTTTGAT
GTGGAGGAGGATGACTCCTCTCGGTCACCATCTCCTCAGGAGATTCTGCTCGAGG
TGGAGCTGGATGAAAACGAAGTCAAAGAGTTTGAGAAGCAGGTGAAGATCATCA
CCATACCCGAGTACACGGCTGACAACAAGAGCATGATCATATCTCTGGACGTGCT
GCCGAGCATTTATGAGGAAGGTG"/>
<sequence id="seq_Aros_Nuc_Apisto_301035”
taxon="Aros_Nuc_Apisto_301035” totalcount="4"
value="TGTTGGAGAAGCCAGAAGTGTTGCTTTAGTGGTGGTTGTATCACGGGAAG
TGACGTTCATGCCCGCCCCACCTGCCGTCTCCCATCAGCATGTGATGGAGTTTGAT
GTGGAGGAGGATGACTCCTCTCGGTCACCATCTCCTCAGGAGATTCTGCTCGAGG
TGGAGCTGGATGAAAACGAAGTCAAAGAGTTTGAGAAGCAGGTGAAGATCATCA
CCATACCCGAGTACACGGCTGACAACAAGAGCATGATCATATCTCTGGACGTGCT
GCCGAGCATTTATGAGGAAGGTG"/>
<sequence id="seq_Aros_Nuc_Apisto_301037”
taxon="Aros_Nuc_Apisto_301037” totalcount="4"
value="TGTTGGAGAAGCCAGAAGTGTTGCTTTAGTGGTGGTTGTATCACGGGAAG
TGACGTTCATGCCCGCCCCACCTGCCGTCTCCCATCAGCATGTGATGGAGTTTGAT
GTGGAGGAGGATGACTCCTCTCGGTCACCATCTCCTCAGGAGATTCTGCTCGAGG
TGGAGCTGGATGAAAACGAAGTCAAAGAGTTTGAGAAGCAGGTGAAGATCATCA
CCATACCCGAGTACACGGCTGACAACAAGAGCATGATCATATCTCTGGACGTGCT
GCCGAGCATTTATGAGGAAGGTG"/>
<sequence id="seq_Asp2_Nuc_Apisto_301290"
taxon="Asp2_Nuc_Apisto_301290" totalcount="4"
value="TGTTGGAGAAGCCAGAAGTGTTGCTTTAGTGGTAGTCGTATCACGGGAAG
TGACATTCATGCCCGCCCCACCTGCCGTCTCCCATCAGCATGTGATGGAGTTCGA
TGTGGAGGAAGATGACTCTTCTCGGTCACCGTCTCCTCAGGAGATTcTGCTGGAA
GTGGAGCTGGATGAAAACGAGGTCAAAGAGTTTGAGAAGCAGGTGAAGATCATC
ACCATACCCGAGTACACAGCTGACAACAAGAGCATGATCATATCTCTGGATGTGC
TACCGAGCATTTATGAGGAAGGTG"/>
<sequence id="seq_Asp2_Nuc_Apisto_301291"
taxon="Asp2_Nuc_Apisto_301291" totalcount="4"

value="TGTTGGAGAAGCCAGAAGTGTTGCTTTAGTGGTAGTCGTATCACGGGAAG
TGACATTCATGCCCGCCCCACCTGCCGTCTCCCATCAGCATGTGATGGAGTTCGA
TGTGGAGGAAGATGACTCTTCTCGGTCACCGTCTCCTCAGGAGATTcTGCTGGAA
GTGGAGCTGGATGAAAACGAGGTCAAAGAGTTTGAGAAGCAGGTGAAGATCATC
ACCATACCCGAGTACACAGCTGACAACAAGAGCATGATCATATCTCTGGATGTGC
TACCGAGCATTTATGAGGAAGGTG"/>
<sequence id="seq_Asp2_Nuc_Apisto_301292"
taxon="Asp2_Nuc_Apisto_301292" totalcount="4"
value="TGTTGGAGAAGCCAGAAGTGTTGCTTTAGTGGTAGTCGTATCACGGGAAG
TGACATTCATGCCCGCCCCACCTGCCGTCTCCCATCAGCATGTGATGGAGTTCGA
TGTGGAGGAAGATGACTCTTCTCGGTCACCGTCTCCTCAGGAGATTcTGCTGGAA
GTGGAGCTGGATGAAAACGAGGTCAAAGAGTTTGAGAAGCAGGTGAAGATCATC
ACCATACCCGAGTACACAGCTGACAACAAGAGCATGATCATATCTCTGGATGTGC
TACCGAGCATTTATGAGGAAGGTG"/>
<sequence id="seq_Awol_Nuc_Apisto_302976"
taxon="Awol_Nuc_Apisto_302976" totalcount="4"
value="TGTTGGAGAAGCCAGAAGTGTTGCTTTAGTGGTGGTTGTATCACGGGAAG
TGACGTTCATGCCCGCCCCACCTGCCGTCTCCCATCAGCATGTGATGGAGTTTGAT
GTGGAGGAGGATGACTCCTCTCGGTCACCATCTCCTCAGGAGATTCTGCTCGAGG
TGGAGCTGGATGAAAACGAAGTCAAAGAGTTTGAGAAGCAGGTGAAGATCATCA
CCATACCCGAGTACACGgCTGACAACAAGAGCATGATCATATCTCTGGACGTGCT
GCCGAGCATTTATGAGGAAGGTG"/>
<sequence id="seq_Bio_Nuc_Biotodoma" taxon="Bio_Nuc_Biotodoma"
totalcount="4"
value="TGTTGGAGAAGCCAGAAGTGTTGCTTTAGTGGTAGTTGTATCACGGGAAG
TGACATTCATGCCTGCCCCACCTGCCGTCTCCCATCAGCATGTGATGGAGTTTGAT
GTGGAGGAAGATGACTCCTCTCGTTCACCATCTCCTCAAGAGATTCTGCTCGAAG
TAGAGCTGGATGAAAATGAAGTCAAAGAGTTTGAGAAACAGGTGAAGATCATCA
CCATACCTGAGTACACAGCTGACAACAAGAGTATGATCATATCTCTGGATGTGTT
ACCGAGTATTTATGAGGAAGGCG"/>
<sequence id="seq_Cre_Nuc_Crenicara" taxon="Cre_Nuc_Crenicara"
totalcount="4"
value="TGTTGGAGAAGCCAGAAGTGTTGCTTTAGTGGTGGTTTTATCACGGGAAG
TGACATTTATGCCTGCCCCACCTGCTGTCTCCCATCAGCACGTGATGGAGTTTGAT
GTGGAGGAAGATGACTCTTCTCGTTCACCATCTCCTCAAGAGATTCTGCTCGAAG
TAGAGCTGGATGAAAATGAAGTCAAAGAGTTTGAGAAACAGGTGAAGATCATCA
CCATACCTGAGTACACAGCTGACAACAAGAGTATGATCATATCTCTGGATGTGTT
ACCGAGTATTTATGAGGAAGGCG"/>
<sequence id="seq_Geo_Nuc_Geophagus" taxon="Geo_Nuc_Geophagus"
totalcount="4"
value="TGTTGGGGAAGCCAGAAGTGTTGCTTTAGTGGTGGTTGTATCACGGGAAG
TGACATTCATGCCTGCCCCACCTGCTGTCTCCCATCAGCATGTGATGGAGTTTGAT
GTGGAGGAAGATGACTCTTCTCGTTCACCATCTCCTCAAGAGATTCTGCTCGAAG
TAGAGCTGGATGAAAATGAAGTCAAAGAGTTTGAGAAACAGGTGAAGATCATCA
CCATACCTGAGTACACAGCTGACAACAAGAGTATGATCATATCTCTGGATGTGTT
ACCGAGTATTTATGAGGAAGGC-"/>
<sequence id="seq_Ggym_Nuc_Gymnogymno"
taxon="Ggym_Nuc_Gymnogymno" totalcount="4"
value="TGTTGGAGAAGCCAGAAGTGTTGCTTTGGTGGTAGTAGTGTCACGGGAAG
TGACATTCATGCCCGCCCCACCTGCTGTCTCCCATCAGCATGTGATGGAGTTTGAT

GTGGAGGAAGACGACTCCTCTCGTTCGCCATCTCCTCAAGAGATTCTGCTCGAAG
TCGAGCTGGATGAAAATGAGGTCAAAGAGTTTGAGAAACAGGTGAAGATCATCA
CCATACCTGAGTACACAGCTGACAACAAGAGTATGATCATATCTCTGGATGTGTT
ACCGAGTATTTATGAGGAAAGCG"/>
<sequence id="seq_Gmer_Nuc_Gymnomer" taxon="Gmer_Nuc_Gymnomer"
totalcount="4"
value="TGTTGGAGAAGCCAGAAGTGTTGCTTTGGTGGTAGTAGTGTCACGGGAAG
TGACATTCATGCCCGCCCCACCTGCTGTCTCCCATCAGCATGTGATGGAGTTTGAT
GTGGAGGAAGACGACTCCTCTCGTTCGCCATCTCCTCAAGAGATTCTGCTCGAAG
TCGAGCTGGATGAAAATGAGGTCAAAGAGTTTGAGAAACAGGTGAAGATCATCA
CCATACCTGAGTACACAGCTGACAACAAGAGTATGATCATATCTCTGGATGTGTT
ACCGAGTATTTATGAGGAAAGCG"/>
<sequence id="seq_Sat_Nuc_Satanoperca" taxon="Sat_Nuc_Satanoperca"
totalcount="4"
value="TGTTGGAGAAGCCAGAAGTGTTGCTTTAGTGGTGGTTGTATCACGGGAAG
TGACATTTATGCCTGCCCCACCTGCTGTCTCCCATCAGCATGTGATGGAGTTTGAT
GTGGAGGAAGATGACTCTTCTCGTTCACCATCTCCTCAAGAGATTCTGCTCGAAG
TAGAGCTGGATGAAAATGAAGTCAAAGAGTTTGAGAAGCAGGTGAAGATCATCA
CCATACCTGAGTACACGGCTGACAACAAGAGTATGATCATATCTCTGGATGTGTT
ACCGAGCATTTATGAGGAAGGCG"/>
</data>
<data
id="Apisto_Combi_Mito"
name="alignment">
<sequence id="seq_Aaga_Mito_Apisto_302902"
taxon="Aaga_Mito_Apisto_302902" totalcount="4"
value="CGCTAATCGACCTTCCCACCCCCTCCAACATCTCCATCTGATGAAATTTTG
GCTCCCTTCTAGGCCTCTGCCTAGTCTCCCAAATCCTAACAGGCTTATTTCTCTCC
ATACATTACACTGCTGACACCAGTACAGCTTTTTCCTCCATCGCCCACATCTGCCG
AGACGTAAACTACGGATGACTAATCCGGAATATACATGCTAACGGAGCATCTTTT
TTCTTCATTTGCATCTATCTACACATCGGACGAGGTCTTTACTTCGGCTCTTATCTT
TACAAAGAGACATGAAATATCGGGGTAATTCTACTACTATTAGTGATAATAACCG
CTTTCGTGGGCTACGTCCTCCCATGAGGGCAAATATCATTCTGGGGGGCCACCGT
CATCACTAACCTGCTATCAGCAGTCCCCTACATTGGCGACTCTTTAGTTCAATGAA
TCTGAGGCGGCTTCTCAGTTGACAACGCCACACTAACCCGTTTTTTGCCTTTCAC
TTTTTACTCCCATTCGCTATCGCAGCCATAACCCTTATTCACCTAATCTTTCTCCAC
GAGACAGGCTCTACTAATCCAATCGGACTAAACCCAAACACAGATAAGATCTCCT
TCCACCCATTCTACGCCCTCAAAGACCTCCTCGGATTCCTAATCCTGCTAGTAGCC
CTAATATTTGGTGCTTGGGCTGGGGTAGTAGGTACCGCGTTAAGCATACTGATTC
GGACAGAGCTTACTCTGCCCGGCTCCTTTTTTGAGGACGACCAGGTCTATAACGT
ACTCGTAACTGCACACGCCTTCGTAATAATTTTCTTTATGGTTATACCAATCATAA
TTGGCGGATTTGGCAATTGACTGATCCCACTAATAATTGGCGCCCCGGACATGGC
CTTCCCCCGCATAAACAACATAAGCTTCTGACTTCTGCCCCCATCCTTCCTCCTCC
TCCTCGCTTCCTCAACAGTCGAAGCCGGCGTGGGAACAGGCTGAACTGTATACCC
TCCCCTCGCCGGAAATTTAGCCCATGACGGCCCAGCCGTAGATCTGGCCATTTTC
TCTCTTCATCTAGCAGGGGTATCTTCAATTTTAGGTGCAATCAACTTTATCACCAC
TATTATTAATATGAAGCCCCCTGCCATCCCTATGTCCCGTACACCCTTATTTATTT
GATCCCTCCTCATCACTGCTGTACTCTTACTTCTTTCGCTTCCAGTGCTTGCTGCCG
GGATCACCATACTCTTGACAGACCGAAACCT"/>

<sequence id="seq_Aaga_Mito_Apisto_302911"
taxon="Aaga_Mito_Apisto_302911" totalcount="4"
value="CGCTAATCGACCTTCCCACCCCCTCCAACATCTCCATCTGATGAAATTTTG
GCTCCCTTCTAGGCCTCTGCCTAGTCTCCCAAATCCTAACAGGCTTATTTCTCTCC
ATACATTACACTGCTGACACCAGTACAGCTTTTTCCTCCATCGCCCACATCTGCCG
AGACGTAAACTACGGATGACTAATCCGGAATATACATGCTAACGGAGCATCTTTT
TTCTTCATTTGCATCTATCTACACATCGGACGAGGTCTTTACTTCGGCTCTTATCTT
TACAAAGAGACATGAAATATCGGGGTAATTCTACTACTATTAGTGATAATAACCG
CTTTCGTGGGCTACGTCCTCCCATGAGGGCAAATATCATTCTGGGGGGCCACCGT
CATCACTAACCTGCTATCAGCAGTCCCCTACATTGGCGACTCTTTAGTTCAATGAA
TCTGAGGCGGCTTCTCAGTTGACAACGCCACACTAACCCGTTTTTTTGCCTTTCAC
TTTTTACTCCCATTCGCTATCGCAGCCATAACCCTTATTCACCTAATCTTTCTCCAC
GAGACAGGCTCTACTAATCCAATCGGACTAAACCCAAACACAGATAAGATCTCCT
TCCACCCATTCTACGCCCTCAAAGACCTCCTCGGATTCCTAATCCTGCTAGTAGCC
CTAATATTTGGTGCTTGGGCTGGGGTAGTAGGTACCGCGTTAAGCATACTGATTC
GGACAGAGCTTACTCTGCCCGGCTCCTTTTTTGAGGACGACCAGGTCTATAACGT
ACTCGTAACTGCACACGCCTTCGTAATAATTTTCTTTATGGTTATACCAATCATAA
TTGGCGGATTTGGCAATTGACTGATCCCACTAATAATTGGCGCCCCGGACATGGC
CTTCCCCCGCATAAACAACATAAGCTTCTGACTTCTGCCCCCATCCTTCCTCCTCC
TCCTCGCTTCCTCAACAGTCGAAGCCGGCGTGGGAACAGGCTGAACTGTATACCC
TCCCCTCGCCGGAAATTTAGCCCATGACGGCCCAGCCGTAGATCTGGCCATTTTC
TCTCTTCATCTAGCAGGGGTATCTTCAATTTTAGGTGCAATCAACTTTATCACCAC
TATTATTAATATGAAGCCCCCTGCCATCCCTATGTCCCGTACACCCTTATTTATTT
GATCCCTCCTCATCACTGCTGTACTCTTACTTCTTTCGCTTCCAGTGCTTGCTGCCG
GGATCACCATACTCTTGACAGACCGAAACCT"/>
<sequence id="seq_Aaga_Mito_Apisto_302918"
taxon="Aaga_Mito_Apisto_302918" totalcount="4"
value="CGCTAATCGACCTTCCCACCCCCTCCAACATCTCCATCTGATGAAATTTTG
GCTCCCTTCTAGGCCTCTGCCTAGTCTCCCAGATCCTAACAGGCTTATTTCTCTCC
ATACACTACACTGCTGACACCAGTACAGCTTTTTCCTCCATCGCCCACATCTGCCG
AGACGTAAACTACGGATGACTAATCCGGAATATACATGCTAACGGAGCATCTTTT
TTCTTCATTTGCACCTATCTACACATCGGACGAGGTCTTTACTTCGGCTCTTATCTT
TACAAAGAGACATGAAATATCGGGGTAATTCTACTACTATTAGTGATAATAACCG
CTTTCGTGGGCTACGTCCTCCCATGAGGGCAAATATCATTCTGGGGGGCCACCGT
CATCACTAACCTGCTATCAGCAGTCCCCTACATTGGCGACTCTTTAGTTCAATGAA
TCTGAGGCGGCTTCTCAGTTGACAACGCCACACTAACCCGTTTTTTTGCCTTTCAC
TTTTTACTCCCATTCGCTATCGCAGCCATAACCCTTATTCACCTAATCTTTCTCCAC
GAGACAGGCTCTACTAATCCAATCGGACTAAACCCAAACACAGATAAGATCTCCT
TCCACCCATTCTACGCCCTCAAAGACCTCCTCGGATTCCTAATCCTGCTAGTAGCC
CTAATATTTGGTGCTTGGGCTGGGGTAGTAGGTACCGCGTTAAGCATACTGATTC
GGACAGAGCTTACTCTGCCCGGCTCCTTTTTTGAGGACGACCAGGTCTATAACGT
ACTCGTAACTGCACACGCCTTCGTAATAATTTTCTTTATGGTTATACCAATCATAA
TTGGCGGATTTGGCAATTGACTGATCCCACTAATAATTGGCGCCCCGGACATGGC
CTTCCCCCGCATAAACAACATAAGCTTCTGACTTCTGCCCCCATCCTTCCTCCTCC
TCCTCGCTTCCTCAACAGTCGAAGCCGGCGTGGGAACAGGCTGAACTGTATACCC
TCCCCTCGCCGGAAATTTAGCCCATAACGGCCCAGCCGTAGATCTGGCCATTTTC
TCTCTTCATCTAGCGGGGGTATCTTCAATTTTAGGTGCAATCAACTTTATCACCAC
TATTATTAATATGAAGCCCCCTGCCATCCCTATGTACCGTACACCCTTATTTATTT
GATCCCTCCTCATCACTGCTGTACTCTTACTTCTTTCGCTTCCAGTGCTTGCTGCCG
GGATCACCATACTCTTGACAGACCGAAACCT"/>

<sequence id="seq_Aaga_Mito_Apisto_302920"
taxon="Aaga_Mito_Apisto_302920" totalcount="4"
value="CGCTAATCGATCTTCCCACCCCCTCCAACATCTCCATCTGATGAAATTTTG
GCTCCCTTCTAGGCCTCTGTCTAGTCTCCCAGATCCTAACAGGCTTATTTCTCTCT
ATACACTACACTGCTGACACCAGTACAGCTTTTACTTCCATCGCCCACATCTGCCG
AGACGTAAACTACGGGTGACTAATCCGGAATATACATGCTAACGGAGCATCTTTT
TTCTTCATTTGCATCTATCTACATATCGGACGGGGTCTTTACTTCGGCTCTTATCTC
TACAAAGAGACATGGAGCATCGGGGTAATCCTACTACTATTAGTGATAATAACCG
CTTTCGTAGGCTACGTCCTCCCATGGGGGCAAATATCATTCTGGGGGGCCACCGT
CATCACTAACCTACTATCAGCAGTCCCCTACATCGGCGACTCTCTAGTTCAATGA
ATCTGAGGCGGCTTCTCAGTTGACAACCCCACACTAACCCGTTTTTTTGCCTTTCA
CTTTTTACTCCCATTCGCTATCGCAGCCATAACCCTTATTCACCTAATCTTTCTCCA
CGAAACAGGCTCTACTAATCCAATCGGACTAAACCCAAACGCAGATAAAATCTC
CTTCCACCCATTCTATGCCCTCAAAGACCTCCTCGGATTTCTAATCCTGCTAATAG
CCCTGATATTTGGTGCTTGGGCAGGGGTAGTAGGTACCGCGTTAAGCATACTAAT
TCGGACAGAGCTTACTCTGCCCGGCTCCTTTTTTGAGGACGACCAGGTCTATAAC
GTACTCGTAACTGCACACGCCTTCGTAATAATTTTCTTTATGGTCATACCAATCAT
AATTGGTGGATTTGGCAATTGACTGATCCCACTAATAATTGGCGCCCCGGACATG
GCCTTCCCCCGCATAAACAACATAAGCTTCTGACTTCTGCCCCCATCCTTTCTCCT
CCTCCTCGCTTCCTCAACAGTCGAAGCCGGCGTGGGAACAGGCTGAACTGTATAC
CCCCCGCTCGCCGGAAATCTAGCCCATGACGGCCCAGCCGTAGATCTGGCCATTT
TCTCTCTTCATTTAGCAGGGGTATCTTCAATTTTAGGTGCAATCAACTTTATCACC
ACTATTATTAACATGAAACCCCCTGCCATCCCTTTATACCGTACACCCTTATTTAT
TTGATCCCTCCTCATCACTGCTGTACTCTTACTTCTTTCGCTTCCAGTGCTTGCTGC
CGGGATCACCATACTCTTGACAGACCGAAACCT"/>
<sequence id="seq_Aaga_Mito_Apisto_302958"
taxon="Aaga_Mito_Apisto_302958" totalcount="4"
value="CGCTAATCGACCTTCCCACCCCCTCCAACATCTCCATCTGATGAAATTTTG
GCTCCCTTCTAGGCCTCTGCCTAGTCTCCCAAATCCTAACAGGCTTATTTCTCTCC
ATACATTACACTGCTGACACCAGTACAGCTTTTTCCTCCATCGCCCACATCTGCCG
AGACGTAAACTACGGATGACTAATCCGGAATATACATGCTAACGGAGCATCTTTT
TTCTTCATTTGCATCTATCTACACATCGGACGAGGTCTTTACTTCGGCTCTTATCTT
TACAAAGAGACATGAAATATCGGGGTTATTCTACTACTATTAGTGATAATAACCG
CTTTCGTGGGCTACGTCCTCCCATGAGGGCAAATATCATTCTGGGGGGCCACCGT
CATCACTAACCTGCTATCAGCAGTCCCCTACATTGGCGACTCTTTAGTTCAATGAA
TCTGAGGCGGCTTCTCAGTTGACAACGCCACACTAACCCGTTTTTTTGCCTTTCAC
TTTTTACTCCCATTCGCTATCGCAGCCATAACCCTTATTCACCTAATCTTTCTCCAC
GAGACAGGCTCTACTAATCCAATCGGACTAAACCCAAACACAGATAAGATCTCCT
TCCACCCATTCTACGCCCTCAAAGACCTCCTCGGATTCCTAATCCTGCTAGTAGCC
CTAATATTTGGTGCTTGGGCTGGGGTAGTAGGTACCGCGTTAAGCATACTGATTC
GGACAGAGCTTACTCTGCCCGGCTCCTTTTTTGAGGACGACCAGGTCTATAACGT
ACTCGTAACTGCACACGCCTTCGTAATAATTTTCTTTATGGTTATACCAATCATAA
TTGGCGGATTTGGCAATTGACTGATCCCACTAATAATTGGCGCCCCGGACATGGC
CTTCCCCCGCATAAACAACATAAGCTTCTGACTTCTGCCCCCATCCTTCCTCCTCC
TCCTCGCTTCCTCAACAGTCGAAGCCGGCGTGGGAACAGGCTGAACTGTATACCC
TCCCCTCGCCGGAAATTTAGCCCATGACGGCCCAGCCGTAGATCTGGCCATTTTC
TCTCTTCATCTAGCAGGGGTATCTTCAATTTTAGGTGCAATCAACTTTATCACCAC
TATTATTAATATGAAGCCCCCTGCCATCCCTATGTCCCGTACACCCTTATTTATTT
GATCCCTCCTCATCACTGCTGTACTCTTACTTCTTTCGCTTCCAGTGCTTGCTGCCG
GGATCACCATACTCTTGACAGACCGAAACCT"/>

<sequence id="seq_Aaga_Mito_Apisto_302959"
taxon="Aaga_Mito_Apisto_302959" totalcount="4"
value="CGCTAATCGACCTTCCCACCCCCTCCAACATCTCCATCTGATGAAATTTTG
GCTCCCTTCTAGGCCTCTGCCTAGTCTCCCAAATCCTAACAGGCTTATTTCTCTCC
ATACATTACACTGCTGACACCAGTACAGCTTTTTCCTCCATCGCCCACATCTGCCG
AGACGTAAACTACGGATGACTAATCCGGAATATACATGCTAACGGAGCATCTTTT
TTCTTCATTTGCATCTATCTACACATCGGACGAGGTCTTTACTTCGGCTCTTATCTT
TACAAAGAGACATGAAATATCGGGGTAATTCTACTACTATTAGTGATAATAACCG
CTTTCGTGGGCTACGTCCTCCCATGAGGGCAAATATCATTCTGGGGGGCCACCGT
CATCACTAACCTGCTATCAGCAGTCCCCTACATTGGCGACTCTTTAGTTCAATGAA
TCTGAGGCGGCTTCTCAGTTGACAACGCCACACTAACCCGTTTTTTTGCCTTTCAC
TTTTTACTCCCATTCGCTATCGCAGCCATAACCCTTATTCACCTAATCTTTCTCCAC
GAGACAGGCTCTACTAATCCAATCGGACTAAACCCAAACACAGATAAGATCTCCT
TCCACCCATTCTACGCCCTCAAAGACCTCCTCGGATTCCTAATCCTGCTAGTAGCC
CTAATATTTGGTGCTTGGGCTGGGGTAGTAGGTACCGCGTTAAGCATACTGATTC
GGACAGAGCTTACTCTGCCCGGCTCCTTTTTTGAGGACGACCAGGTCTATAACGT
ACTCGTAACTGCACACGCCTTCGTAATAATTTTCTTTATGGTTATACCAATCATAA
TTGGCGGATTTGGCAATTGACTGATCCCACTAATAATTGGCGCCCCGGACATGGC
CTTCCCCCGCATAAACAACATAAGCTTCTGACTTCTGCCCCCATCCTTCCTCCTCC
TCCTCGCTTCCTCAACAGTCGAAGCCGGCGTGGGAACAGGCTGAACTGTATACCC
TCCCCTCGCCGGAAATTTAGCCCATGACGGCCCAGCCGTAGATCTGGCCATTTTC
TCTCTTCATCTAGCAGGGGTATCTTCAATTTTAGGTGCAATCAACTTTATCACCAC
TATTATTAATATGAAGCCCCCTGCCATCCCTATGTCCCGTACACCCTTATTTATTT
GATCCCTCCTCATCACTGCTGTACTCTTACTTCTTTCGCTTCCAGTGCTTGCTGCCG
GGATCACCATACTCTTGACAGACCGAAACCT"/>
<sequence id="seq_Aaga_Mito_Apisto_303077"
taxon="Aaga_Mito_Apisto_303077" totalcount="4"
value="CGCTAATCGATCTTCCCACCCCCTCCAACATCTCCATCTGATGAAATTTTG
GCTCCCTTCTAGGCCTCTGTCTAGTCTCCCAGATCCTAACAGGCTTATTTCTCTCT
ATACACTACACTGCTGACACCAGTACAGCTTTTACTTCCATCGCCCACATCTGCCG
AGACGTAAACTACGGGTGACTAATCCGGAATATACATGCTAACGGAGCATCTTTT
TTCTTCATTTGCATCTATCTACATATCGGACGGGGTCTTTACTTCGGCTCTTATCTC
TACAAAGAGACATGGAGCATCGGGGTAATCCTACTACTATTAGTGATAATAACCG
CTTTCGTAGGCTACGTCCTCCCATGGGGGCAAATATCATTCTGGGGGGCCACCGT
CATCACTAACCTACTATCAGCAGTCCCCTACATCGGCGACTCTCTAGTTCAATGA
ATCTGAGGCGGCTTCTCAGTTGACAACCCCACACTAACCCGTTTTTTTGCCTTTCA
CTTTTTACTCCCATTCGCTATCGCAGCCATAACCCTTATTCACCTAATCTTTCTCCA
CGAAACAGGCTCTACTAATCCAATCGGACTAAACCCAAACGCAGATAAAATCTC
CTTCCACCCATTCTATGCCCTCAAAGACCTCCTCGGATTTCTAATCCTGCTAATAG
CCCTGATATTTGGTGCTTGGGCTGGGGTAGTAGGTACCGCGTTAAGCATACTGAT
TCGGACAGAGCTTACTCTGCCCGGCTCCTTTTTTGAGGACGACCAGGTCTATAAC
GTACTCGTAACTGCACACGCCTTCGTAATAATCTTCTTTATGGTTATACCAATCAT
AATTGGCGGATTTGGCAATTGACTGATCCCACTAATAATTGGCGCCCCGGACATG
GCCTTCCCCCGCATAAACAACATAAGCTTCTGACTTCTGCCCCCATCCTTCCTCCT
CCTCCTCGCTTCCTCAACAGTCGAAGCCGGCGTGGGAACAGGCTGAACTGTATAC
CCTCCCCTCGCCGGAAATTTAGCCCATGACGGCCCAGCCGTAGATCTGGCCATTT
TCTCTCTTCATCTAGCGGGGGTATCTTCAATTTTAGGTGCAATCAACTTTATCACC
ACTATTATTAATATGAAGCCCCCTGCCATCCCTATGTCCCGTACACCCTTATTTAT
TTGATCCCTCCTCATCACTGCTGTACTCTTACTTCTTTCGCTTCCAGTGCTTGCTGC
CGGGATCACCATACTCTTGACAGACCGaAACCT"/>

<sequence id="seq_Aaga_Mito_Apisto_303078"
taxon="Aaga_Mito_Apisto_303078" totalcount="4"
value="CGCTAATCGACCTTCCCACCCCCTCCAACATCTCCATCTGATGAAATTTTG
GCTCCCTTCTAGGCCTCTGCCTAGTCTCCCAGATCCTAACAGGCTTATTTCTCTCC
ATACACTACACTGCTGACACCAGTACAGCTTTTCCTCCATCGCCCACATCTGCCG
AGACGTAAACTACGGATGACTAATCCGGAATATACATGCTAACGGAGCATCTTTT
TTCTTCATTTGCATCTATCTACACATCGGACGAGGTCTTTACTTCGGCTCTTATCTT
TACAAAGAGACATGAAATATCGGGGTAATTCTACTACTATTAGTGATAATAACCG
CTTTCGTGGGCTACGTCCTCCCATGAGGGCAAATATCATTCTGGGGGGCCACCGT
CATCACTAACCTGCTATCAGCAGTCCCCTACATTGGCGACTCTTTAGTTCAATGAA
TCTGAGGCGGCTTCTCAGTTGACAACGCCACACTAACCCGTTTTTTGCCTTTCAC
TTTTTACTCCCATTCGCTATCGCAGCCATAACCCTTATTCACCTAATCTTTCTCCAC
GAGACAGGCTCTACTAATCCAATCGGACTAAACCCAAACACAGATAAGATCTCCT
TCCACCCATTCTACGCCCTCAAAGACCTCCTCGGATTCCTAATCCTGCTAGTAGCC
CTAATATTTGGTGCTTGGGCAGGGGTAGTAGGTACCGCGTTAAGCATACTAATTC
GGACAGAGCTTACTCTGCCCGGCTCCTTTTTTGAGGACGACCAGGTCTATAACGT
ACTCGTAACTGCACACGCCTTCGTAATAATTTTCTTTATGGTCATACCAATCATAA
TTGGTGGATTTGGCAATTGACTGATCCCACTAATAATTGGCGCCCCGGACATGGC
CTTCCCCCGCATAAACAACATAAGCTTCTGACTTCTGCCCCCATCCTTTCTCCTCC
TCCTCGCTTCCTCAACAGTCGAAGCCGGCGTGGGAACAGGCTGAACTGTATACCC
CCCGCTCGCCGGAAATCTAGCCCATGACGGCCCAGCCGTAGATCTGGCCATTTTC
TCTCTTCATTTAGCAGGGGTATCTTCAATTTTAGGTGCAATCAACTTTATCACCAC
TATTATTAACATGAAACCCCCTGCCATCCCTTTATACCGTACACCCTTATTTATTT
GATCCCTCCTCATCACTGCTGTACTCTTACTTCTTTCGCTTCCAGTGCTTGCTGCCG
GGATCACCATACTCTTGACAGACCGAAACCT"/>
<sequence id="seq_Aaga_Mito_Apisto_300492"
taxon="Aaga_Mito_Apisto_300492" totalcount="4"
value="CGCTAATCGACCTTCCCACCCCCTCCAACATCTCCATCTGATGAAATTTTG
GCTCCCTTCTAGGCCTCTGCCTAGTCTCCCAAATCCTAACAGGCTTATTTCTCTCC
ATACACTACACTGCTGACACCACTACAGCTTTTCCTCCATCGCCCACATCTGCCG
AGACGTAAACTACGGGTGGCTAATCCGGAATATACATGCTAACGGAGCATCTTTT
TTCTTCATTTGCATCTATCTACACATCGGACGAGGTCTTTACTTCGGCTCTTATCTT
TACAAAGAGACATGAAATATTGGGGTAATTCTACTGCTATTAGTGATAATAACCG
CTTTCGTGGGCTACGTCCTCCCATGAGGACAAATATCATTCTGGGGGGCCACCGT
CATCACTAACCTGCTATCAGCAGTCCCCTACATTGGCGACTCTTTAGTTCAATGAA
TCTGAGGCGGCTTCTCAGTTGACAACGCCACACTAACCCGTTTTTTGCCTTTCAC
TTTTTACTCCCATTCGCTATCGCAGCCATAACCCTTATTCACCTAATCTTTCTCCAC
GAGACAGGCTCTACTAATCCAATCGGACTAAACCCAAACACAGATAAAATCTCCT
TCCACCCATTCTACGCCCTCAAAGACCTCCTCGGATTCCTAATCCTGCTAGTAGCC
CTAATATTTGGTGCTTGGGCTGGGGTAGTAGGTACCGCGTTAAGCATACTGATTC
GGACAGAGCTTACTCTGCCCGGCTCCTTTTTTGAGGACGACCAGGTCTATAACGT
ACTCGTAACTGCACACGCCTTCGTAATAATTTTCTTTATGGTTATACCAATCATAA
TTGGCGGATTTGGCAATTGACTGATCCCACTAATAATTGGCGCCCCGGACATGGC
CTTCCCCCGCATAAACAACATAAGCTTCTGACTTCTGCCCCCATCCTTCCTCCTCC
TCCTCGCTTCCTCAACAGTCGAAGCCGGCGTGGGAACAGGCTGAACTGTATACCC
TCCCCTCGCCGGAAATTTAGCCCATGACGGCCCAGCCGTAGATCTGGCCATTTTC
TCTCTTCATCTAGCGGGGGTATCTTCAATTTTAGGTGCAATCAACTTTATCACCAC
TATTATTAATATGAAGCCCCCTGCCATCCCTATGTACCGTACACCCTTATTTATTT
GATCCCTCCTCATCACTGCTGTACTCTTACTTCTTTCGCTTCCAGTGCTTGCTGCCG
GGATCACCATACTCTTGACAGACCGAAACCT"/>

<sequence id="seq_Aaga_Mito_Apisto_300494"
taxon="Aaga_Mito_Apisto_300494" totalcount="4"
value="CGCTAATCGACCTTCCCACCCCCTCCAACATCTCCATCTGATGAAATTTTG
GCTCCCTTCTAGGCCTCTGCCTAGTCTCCCAAATCCTAACAGGCTTATTTCTCTCC
ATACACTACACTGCTGACACCACTACAGCTTTTTCCTCCATCGCCCACATCTGCCG
AGACGTAAACTACGGGTGGCTAATCCGGAATATACATGCTAACGGAGCATCTTTT
TTCTTCATTTGCATCTATCTACACATCGGACGAGGTCTTTACTTCGGCTCTTATCTT
TACAAAGAGACATGAAATATTGGGGTAATTCTACTGCTATTAGTGATAATAACCG
CTTTCGTGGGCTACGTCCTCCCATGAGGACAAATATCATTCTGGGGGGCCACCGT
CATCACTAACCTGCTATCAGCAGTCCCCTACATTGGCGACTCTTTAGTTCAATGAA
TCTGAGGCGGCTTCTCAGTTGACAACGCCACACTAACCCGTTTTTTTGCCTTTCAC
TTTTTACTCCCATTCGCTATCGCAGCCATAACCCTTATTCACCTAATCTTTCTCCAC
GAGACAGGCTCTACTAATCCAATCGGACTAAACCCAAACACAGATAAAATCTCCT
TCCACCCATTCTACGCCCTCAAAGACCTCCTCGGATTCCTAATCCTGCTAGTAGCC
CTAATATTTGGTGCTTGGGCTGGGGTAGTGGGTACCGCGTTAAGCATACTGATTC
GGACAGAGCTTACTCTGCCCGGCTCCTTTTTTGAGGACGACCAGGTCTATAACGT
ACTCGTAACTGCACACGCCTTCGTAATAATTTTCTTTATGGTTATACCAATCATAA
TTGGCGGATTTGGCAATTGACTGATCCCACTAATAATTGGCGCCCCGGACATGGC
CTTCCCCCGCATAAACAACATAAGCTTCTGACTTCTGCCCCCATCCTTCCTCCTCC
TCCTCGCTTCCTCAACAGTCGAAGCCGGCGTGGGAACAGGCTGAACTGTATACCC
TCCCCTCGCCGGAAATTTAGCCCATGACGGCCCAGCCGTAGATCTGGCCATTTTC
TCTCTTCATCTAGCGGGGGTATCTTCAATTTTAGGTGCAATCAACTTTATCACCAC
TATTATTAATATGAAGCCCCCTGCCATCCCTATGTACCGTACACCCTTATTTATTT
GATCCCTCCTCATCACTGCTGTACTCTTACTTCTTTCGCTTCCAGTGCTTGCTGCCG
GGATCACCATACTCTTGACAGACCGAAACCT"/>
<sequence id="seq_Aaga_Mito_Apisto_302185"
taxon="Aaga_Mito_Apisto_302185" totalcount="4"
value="CGCTAATCGACCTTCCCACCCCCTCCAACATCTCCATCTGATGAAATTTTG
GCTCCCTTCTAGGCCTCTGCCTAGTCTCCCAAATCCTAACAGGCTTATTTCTCTCC
ATACATTACACTGCTGACACCAGTACAGCTTTTTCCTCCATCGCCCACATCTGCCG
AGACGTAAACTACGGATGACTAATCCGGAATATACATGCTAACGGAGCATCTTTT
TTCTTCATTTGCATCTATCTACACATCGGACGAGGTCTTTACTTCGGCTCTTATCTT
TACAAAGAGACATGAAATATCGGGGTAATTCTACTACTATTAGTGATAATAACCG
CTTTCGTGGGCTACGTCCTCCCATGAGGGCAAATATCATTCTGGGGGGCCACCGT
CATCACTAACCTGCTATCAGCAGTCCCCTACATTGGCGACTCTTTAGTTCAATGAA
TCTGAGGCGGCTTCTCAGTTGACAACGCCACACTAACCCGTTTTTTTGCCTTTCAC
TTTTTACTCCCATTCGCTATCGCAGCCATAACCCTTATTCACCTAATCTTTCTCCAC
GAGACAGGCTCTACTAATCCAATCGGACTAAACCCAAACACAGATAAGATCTCCT
TCCACCCATTCTACGCCCTCAAAGACCTCCTCGGATTCCTAATCCTGCTAGTAGCC
CTAATATTTGGTGCTTGGGCTGGGGTAGTAGGTACCGCGTTAAGCATACTGATTC
GGACAGAGCTTACTCTGCCCGGCTCCTTTTTTGAGGACGACCAGGTCTATAACGT
ACTCGTAACTGCACACGCCTTCGTAATAATTTTCTTTATGGTTATACCAATCATAA
TTGGCGGATTTGGCAATTGACTGATCCCACTAATAATTGGCGCCCCGGACATGGC
CTTCCCCCGCATAAACAACATAAGCTTCTGACTTCTGCCCCCATCCTTCCTCCTCC
TCCTCGCTTCCTCAACAGTCGAAGCCGGCGTGGGAACAGGCTGAACTGTATACCC
TCCCCTCGCCGGAAATTTAGCCCATGACGGCCCAGCCGTAGATCTGGCCATTTTC
TCTCTTCATCTAGCGGGGGTATCTTCAATTTTAGGTGCAATCAACTTTATCACCAC
TATTATTAATATGAAGCCCCCTGCCATCCCTATGTCCCGTACACCCTTATTTATTT
GATCCCTCCTCATCACTGCTGTACTCTTACTTCTTTCGCTTCCAGTGCTTGCTGCCG
GGATCACCATACTCTTGACAGACCGAAACTT"/>

<sequence id="seq_Aaga_Mito_Apisto_302186"
taxon="Aaga_Mito_Apisto_302186" totalcount="4"
value="CGCTAATCGACCTTCCCACCCCCTCCAACATCTCCATCTGATGAAATTTTG
GCTCCCTTCTAGGCCTCTGCCTAGTCTCCCAAATCCTAACAGGCTTATTTCTCTCC
ATACATTACACTGCTGACACCAGTACAGCTTTTTCCTCCATCGCCCACATCTGCCG
AGACGTAAACTACGGATGACTAATCCGGAATATACATGCTAACGGAGCATCTTTT
TTCTTCATTTGCATCTATCTACACATCGGACGAGGTCTTTACTTCGGCTCTTATCTT
TACAAAGAGACATGAAATATCGGGGTAATTCTACTACTATTAGTGATAATAACCG
CTTTCGTGGGCTACGTCCTCCCATGAGGGCAAATATCATTCTGGGGGGCCACCGT
CATCACTAACCTGCTATCAGCAGTCCCCTACATTGGCGACTCTTTAGTTCAATGAA
TCTGAGGCGGCTTCTCAGTTGACAACGCCACACTAACCCGTTTTTTGCCTTTCAC
TTTTTACTCCCATTCGCTATCGCAGCCATAACCCTTATTCACCTAATCTTTCTCCAC
GAGACAGGCTCTACTAATCCAATCGGACTAAACCCAAACACAGATAAGATCTCCT
TCCACCCATTCTACGCCCTCAAAGACCTCCTCGGATTCCTAATCCTGCTAGTAGCC
CTAATATTTGGTGCTTGGGCTGGGGTAGTAGGTACCGCGTTAAGCATACTGATTC
GGACAGAGCTTACTCTGCCCGGCTCCTTTTTTGAGGACGACCAGGTCTATAACGT
ACTCGTAACTGCACACGCCTTCGTAATAATTTTCTTTATGGTTATACCAATCATAA
TTGGCGGATTTGGCAATTGACTGATCCCACTAATAATTGGCGCCCCGGACATGGC
CTTCCCCCGCATAAACAACATAAGCTTCTGACTTCTGCCCCCATCCTTCCTCCTCC
TCCTCGCTTCCTCAACAGTCGAAGCCGGCGTGGGAACAGGCTGAACTGTATACCC
TCCCCTCGCCGGAAATTTAGCCCATGACGGCCCAGCCGTAGATCTGGCCATTTTC
TCTCTTCATCTAGCGGGGGTATCTTCAATTTTAGGTGCAATCAACTTTATCACCAC
TATTATTAATATGAAGCCCCCTGCCATCCCTATGTCCCGTACACCCTTATTTATTT
GATCCCTCCTCATCACTGCTGTACTCTTACTTCTTTCGCTTCCAGTGCTTGCTGCCG
GGATCACCATACTCTTGACAGACCGAAACTT"/>
<sequence id="seq_Aaga_Mito_Apisto_302306"
taxon="Aaga_Mito_Apisto_302306" totalcount="4"
value="CGCTAATCGACCTTCCCACCCCCTCCAACATCTCCATCTGATGAAATTTTG
GCTCCCTTCTAGGCCTCTGCCTAGTCTCCCAGATCCTAACAGGCTTATTTCTCTCC
ATACACTACACTGCTGACACCAGTACAGCTTTTTCTTCCATCGCCCACATCTGCCG
AGACGTAAACTACGGGTGGCTGATCCGGAGTATACATGCTAACGGAGCATCTTTT
TTCTTCATTTGCATCTATCTACACATCGGACGGGGTCTTTACTTCGGCTCTTATCTT
TACAAAGAGACATGAAATATCGGAGTAATTCTGCTACTATTAGTGATAATAACCG
CTTTCATGGGCTACGTCCTCCCATGAGGACAAATATCATTCTGGGGGGCCACCGT
CATCACTAACCTGCTATCAGCAGTCCCCCACATTGGCGACTCTTTAGTTCAATGA
ATCTGAGGCGGCTTCTCAGTTGACAACGCCACACTAACCCGTTTTTTGCCTTTCA
CTTTTTACTCCCATTCGCTATCGCAGCCATAACCCTTATTCACCTAATCTTTCTCCA
CGAGACAGGCTCTACTAATCCAATCGGACTAAACCCAAACACAGATAAAATCTC
CTTCCACCCATTCTACGCCCTCAAAGACCTCCTCGGATTTCTAATCCTACTAGTAG
CCCTAATATTTGGTGCTTGGGCTGGGGTAGTAGGTACCGCGTTAAGCATACTGAT
TCGGACAGAGCTTACTCTGCCCGGCTCCTTTTTTGAGGACGACCAGGTCTACAAC
GTACTCGTAACTGCACACGCCTTCGTAATAATTTTCTTTATGGTTATACCAATCAT
AATTGGTGGATTTGGCAATTGACTGATCCCACTAATAATTGGCGCCCCGGACATG
GCCTTCCCCCGCATAAACAACATAAGCTTCTGACTTCTACCCCCATCCTTCCTCCT
CCTCCTCGCTTCCTCAACAGTCGAAGCCGGCGTGGGAACAGGCTGAACTGTATAC
CCTCCCCTCGCCGGAAATTTAGCCCACAACGGCCCAGCCGTAGATCTGGCCATTT
TCTCTCTTCATCTAGCGGGGGTATCTTCAATTTTAGGTGCAATCAACTTTATCACC
ACTATTATTAATATGAAACCCCCTGCCATCCCTATGTACCGTACACCCTTATTTAT
TTGATCCCTCCTTATCACTGCTGTACTCTTACTTCTTTCGCTTCCAGTGCTTGCTGC
CGGGATCACCATACTCTTGACAGACCGAAACCT"/>

<sequence id="seq_Aaga_Mito_Apisto_302307"
taxon="Aaga_Mito_Apisto_302307" totalcount="4"
value="CGCTAATCGACCTTCCCACCCCCTCCAACATCTCCATCTGATGAAATTTTG
GCTCCCTTCTAGGCCTCTGCCTAGTCTCCCAAATCCTAACAGGCTTATTTCTCTCC
ATACATTACACTGCTGACACCAGTACAGCTTTTTCCTCCATCGCCCACATCTGCCG
AGACGTAAACTACGGATGACTAATCCGGAATATACATGCTAACGGAGCATCTTTT
TTCTTCATTTGCATCTATCTACACATCGGACGAGGTCTTTACTTCGGCTCTTATCTT
TACAAAGAGACATGAAATATCGGGGTAATTCTACTACTATTAGTGATAATAACCG
CTTTCGTGGGCTACGTCCTCCCATGAGGCAAATATCATTCTGGGGGGCCACCGT
CATCACTAACCTGCTATCAGCAGTCCCCTACATTGGCGACTCTTTAGTTCAATGAA
TCTGAGGCGGCTTCTCAGTTGACAACGCCACACTAACCCGTTTTTTTGCCTTTCAC
TTTTTACTCCCATTCGCTATCGCAGCCATAACCCTTATTCACCTAATCTTTCTCCAC
GAGACAGGCTCTACTAATCCAATCGGACTAAACCCAAACACAGATAAGATCTCCT
TCCACCCATTCTACGCCCTCAAAGACCTCCTCGGATTCCTAATCCTGCTAGTAGCC
CTAATATTTGGTGCTTGGGCTGGGGTAGTAGGTACCGCGTTAAGCATACTGATTC
GGACAGAGCTTACTCTGCCCGGCTCCTTTTTTGAGGACGACCAGGTCTATAACGT
ACTCGTAACTGCACACGCCTTCGTAATAATTTTCTTTATGGTTATACCAATCATAA
TTGGCGGATTTGGCAATTGACTGATCCCACTAATAATTGGCGCCCCGGACATGGC
CTTCCCCCGCATAAACAACATAAGCTTCTGACTTCTGCCCCCATCCTTCCTCCTCC
TCCTCGCTTCCTCAACAGTCGAAGCCGGCGTGGGAACAGGCTGAACTGTATACCC
TCCCCTCGCCGGAAATTTAGCCCATGACGGCCCAGCCGTAGATCTGGCCATTTTC
TCTCTTCATCTAGCGGGGGTATCTTCAATTTTAGGTGCAATCAACTTTATCACCAC
TATTATTAATATGAAGCCCCCTGCCATCCCTATGTCCCGTACACCCTTATTTATTT
GATCCCTCCTCATCACTGCTGTACTCTTACTTCTTTCGCTTCCAGTGCTTGCTGCCG
GGATCACCATACTCTTGACAGACCGAAACCT"/>
<sequence id="seq_Aalg_Mito_Apisto_300787"
taxon="Aalg_Mito_Apisto_300787" totalcount="4"
value="CACTAATTGACCTCCCCACCCCCTCCAACATCTCTGCTTGATGAAATTTCG
GGTCTCTACTAGGACTATGCTTAATTTCCCAAATTCTAACAGGCTTATTTCTTTCC
ATGCATTATACTGCCGACATCAATACAGCTTTTTCATCCATCACCCACATCTGCCG
AGACGTAAACTACGGTTGGCTAATCCGAAATTTACATGCCAACGGGGCATCCTTT
TTTTTCATTTGTATCTACTTACACATTGCACGAGGCCTCTATTACGGCTCATTTCTC
TACAAAGAAACATGAAACATTGGAGTAATCCTTTTACTATTAATAATAATAACCG
CTTTCGTAGGCTATGTACTCCCATGAGGACAAATATCGTTTTGAGGGGCTACAGT
CATTACCAACTTACTTTCCGCTGTTCCTTACATTGGAAACTCACTAGTCCAATGAA
TCTGAGGTGGCTTCTCAGTCGACAGTGCCACCCTTACACGATTCTTCGCTATCCAT
TTTCTGCTTCCATTTGTCATCGCAGCCCTAACCCTAATCCACCTAATTTTTCTTCAT
GAGACAGGGTCCACCAACCCAATTGGACTAAGCCCAAACACAGACAAAATCTCC
TTCCACCCATACTTCTCTTACAAAGACCTTCTAGGCTTCTTAATCCTACTTTTAACT
TTAGTGTTCGGTGCCTGAGCTGGTATGGCAGGAACCGCACTAAGCATACTAATTC
GAGCGGAGCTAACTCAGCCCGGCTCCTTTTTTGGGGACGACCAAATCTATAATGT
AATTGTTACTGCACATGCCTTCGTAATAATCTTTTTTATAGTGATGCCAATTATGA
TTGGCGGATTCGGTAATTGATTAATTCCACTAATAATTGGTGCCCCAGATATGGC
TTTCCCTCGCATAAATAATATAAGCTTTTGACTGCTACCCCCTTCCTTCCTCCTCCT
CCTTGCCTCCTCCACTGTTGAAGCTGGGGTGGGAACAGGTTGAACTGTGTACCCC
CCCCTTTCAGGAAATTTAGCCCACGACGGCCCATCTGTAGACCTAGCCATCTTCTC
CCTTCACCTAGCAGGAGTATCTTCAATTTTAGGTGCAATTAATTTTATCACCACTA
TTATTAACATAAAACCTCCAACCACCTCCCTGTATAATGCACCATTATTTATCTGG
TCTCTCCTTGTCACGGCTGTTCTTCTACTTCTCTCTCTACCAGTCCTTGCTGCAGGA
ATCACTATACTTCTAACAGATCGAAACCT"/>

<sequence id="seq_Aalg_Mito_Apisto_300788"
taxon="Aalg_Mito_Apisto_300788" totalcount="4"
value="CACTAATTGACCTCCCCACCCCCTCCAACATCTCTGCTTGATGAAATTTCG
GGTCTCTACTAGGACTATGCTTAATTTCCCAAATTCTAACAGGCTTATTTCTTTCC
ATGCATTATACTGCCGACATCAATACAGCTTTTTCATCCATCACCCACATCTGCCG
AGACGTAAACTACGGTTGGCTAATCCGAAATTTACATGCCAACGGGGCATCCTTT
TTTTTCATTTGTATCTACTTACACATTGCACGAGGCCTCTATTACGGCTCATTTCTC
TACAAAGAAACATGAAACATTGGAGTAATCCTTTTACTATTAATAATAATAACCG
CTTTCGTAGGCTATGTACTCCCATGAGGACAAATATCGTTTTGAGGGGCTACAGT
CATTACCAACTTACTTTCCGCTGTTCCTTACATTGGAAACTCACTAGTCCAATGAA
TCTGAGGTGGCTTCTCAGTCGACAGTGCCACCCTTACACGATTCTTCGCTATCCAT
TTTCTGCTTCCATTTATCATCGCAGCCCTAACCCTAATCCACCTAATTTTTCTTCAT
GAGACAGGGTCCACCAACCCAATTGGGCTAAGCCCAAACACAGACAAAATCTCC
TTCCACCCATACTTCTCTTACAAAGACCTTCTAGGCTTCTTAATCCTACTTTTAACT
TTAGTGTTCGGTGCCTGAGCTGGTATGGCAGGAACCGCACTAAGCATACTAATTC
GAGCGGAGCTAACTCAGCCCGGCTCCTTTTTTGGGGACGACCAAATCTATAATGT
AATTGTTACTGCACATGCCTTCGTAATAATCTTTTTTATAGTGATGCCAATTATGA
TTGGCGGATTCGGTAATTGATTAATTCCACTAATAATTGGTGCCCCAGATATGGC
TTTCCCTCGCATAAATAATATAAGCTTTTGACTGCTACCCCCTTCCTTCCTCCTCCT
CCTTGCCTCCTCCACTGTTGAAGCTGGGGTGGGAACAGGTTGAACTGTGTACCCC
CCCCTTTCAGGAAATTTAGCCCACGACGGCCCATCTGTAGACCTAGCCATCTTCTC
CCTTCACCTAGCAGGAGTATCTTCAATTTTAGGTGCAATTAATTTTATCACCACTA
TTATTAACATAAAACCTCCAACCACCTCCCTGTATAATGCTCCATTATTTATCTGG
TCTCTCCTTGTCACGGCTGTTCTTCTACTTCTCTCTCTACCAGTCCTTGCTGCAGGA
ATCACTATACTTCTAACAGATCGAAACCT"/>
<sequence id="seq_Aata_Mito_Apisto_300411"
taxon="Aata_Mito_Apisto_300411" totalcount="4"
value="CGCTAATTGACCTCCCCACCCCCTCCAACATTTCATCATGATGAAATTTTG
GGTCCCTGCTAGGCCTCTGCCTAATCTCCCAGATCCTAACAGGCCTATTTCTTTCC
GTACACTACGCTGCCGACATCAACACAGCTTTTTCATCCGTTGCCCACATCTGTCG
AGATGTAAACTACGGATGACTAATCCGAAACCTACACGCCAACGGAGCGTCCTTT
TTCTTCATTTGCATTTACCTGCACATCGGACGAGGCCTTTACTTCGGCTCCTATCT
CTGTAAAGAGACATGAAATGTCGGGGTACTGCTTCTACTGTTAGTTATAATAACC
GCTTTCGTGGGCTATGTCCTCCCATGAGGACAAATATCGTTTTGAGGAGCCACTG
TCATCACCAACCTTCTATCAGCAGTTCCCTACATCGGAGACTCCCTGGTCCAATG
AATTTGAGGGGGCTTCTCAGTTGACAACGCCACCTTAACCCGATTCTTTGCCTTCC
ACTTCCTTCTCCCGTTCGTCATCGCAGCAATAGCTTTGATACACCTAATTTTCCTG
CATGAGACAGGCTCCACAAACCCAATCGGACTAAACCCAAATACAGATAAAATT
TCTTTTCACCCATTTTACTCCCTCAAAGACCTCCTTGGCTTCCTAATCTTACTCATA
GCCCTCATATTTGGTGCTTGAGCTGGAATAGtAGGcACCGCATTAAGCATACTAATt
CGAGCAGAGCTAACcCAGCCCGGcTCCTTTTTcGGAGACGACCAAcTTTATAATGT
AgTcGTAACTGCACACGCCTTTGTAATAATcTTTTTTATAGTCATACCAATTATAAT
TGGCGGaTTTGGTAATTGAcTAATcCCGCTAATAATCGGCGCCCCAGACATGGCCT
TCCCTCGTATGAATAACATAAGCTTTTGACTACTCCCCCCGTCCTTCCTCCTTCTC
CTCGCCTCCTCGACTGTTGAGGCTGGcGTAGGAACCGGCTGAACTGTTTATCCCCC
CCTCTCCGGGAGCCTGGCTCACGACGGCCCATCCGTgGACCTGGCCATTTTCTCCC
TcCATcTAGCAGGGGTcTCcTCAATTTTAGGTGCAATCAAcTTTATtACaACTgTTAtC
AAcATGAAACCcCCAgCCACCTCCATATATAgTACgCCcTTATTTATCTGgTCCCTCC
TCatTACTGCTGTGCTTCTACTTCTTTCACTACCAGTACTTGCTGCTGGTATTACTAT
ACTTCTgACAGATCGaAACCT"/>

<sequence id="seq_Aata_Mito_Apisto_301983"
taxon="Aata_Mito_Apisto_301983" totalcount="4"
value="CGCTAATTGACCTCCCCACCCCCTCCAACATTTCATCATGATGAAATTTTG
GGTCCCTGCTAGGCCTCTGCCTAATCTCCCAGATCCTAACAGGCCTATTTCTTTCC
GTACACTACACTGCCGACATCAACACAGCTTTTCATCCGTTGCCCACATCTGTCG
AGATGTAAACTACGGATGACTAATCCGAAACCTACACGCCAACGGAGCATCCTTT
TTCTTCATTTGCATTTACCTGCACATCGGACGAGGCCTTTACTTCGGCTCCTATCT
CTGTAAAGAGACATGAAATGTCGGGGTACTGCTTCTACTGTTAGTTATAATAACC
GCTTTCGTGGGCTATGTCCTCCCATGAGGACAAATATCGTTTTGAGGAGCCACTG
TCATCACCAACCTTCTATCAGCAGTTCCCTACATCGGAGACTCCCTGGTCCAATG
AATTTGAGGGGGCTTCTCAGTTGACAACGCCACCTTAACCCGATTCTTTGCCTTCC
ACTTCCTTCTCCCGTTCGTCATCGCAGCAATAGCTTTGATACACCTAATTTTCCTG
CATGAGACAGGCTCCACAAACCCAATCGGACTAAACCCAAATACAGATAAAATT
TCTTTTCACCCATTTTACTCCCTCAAAGACCTCCTTGGCTTCCTAATCTTACTCATA
GCCCTCATATTTGGTGCTTGAGCTGGAATAGTAGGCACCGCATTAAGCATACTAA
TCCGAGCAGAGCTAACCCAGCCCGGCTCCTTTTTCGGAGACGACCAACTTTATAA
TGTAGTCGTAACTGCACACGCCTTTGTAATAATCTTTTTTATAGTCATACCAATTA
TAATTGGCGGCTTTGGTAATTGACTAATCCCGCTAATAATCGGCGCCCCAGACAT
GGCCTTCCCTCGTATGAATAACATAAGCTTTTGACTACTCCCCCCGTCCTTCCTCC
TTCTCCTCGCCTCCTCGACTGTTGAGGCTGGCGTAGGAACCGGCTGAACTGTTTAT
CCTCCCCTCTCCGGGAGCCTGGCTCACGACGGCCCATCCGTGGACCTGGCCATTT
TCTCCCTCCATCTAGCAGGGGTCTCCTCAATTTTAGGTGCAATCAACTTTATTACA
ACTGTTATCAATATGaaACCCCCAGCCACCTCCATATATAGTATGCCCTTATTTATc
TGGTCCCTCCTCATTACTGCTGTGCTTATACTTCTTTCACTACCAGTACTTGCTGCT
GGTATTACTATACTTCTGACAGATCgAAACCT"/>
<sequence id="seq_Aata_Mito_Apisto_301990"
taxon="Aata_Mito_Apisto_301990" totalcount="4"
value="CGCTAATTGACCTCCCCGCCCCCTCCAACATTTCATCATGATGAAATTTTG
GGTCCCTGCTAGGCCTCTGCCTAATCTCCCAGATCCTAACAGGCCTATTTCTTTCC
ATACACTACACTGCCGACATCAACACAGCTTTTCATCCGTTGCCCACATCTGTCG
AGATGTAAACTACGGATGACTAATCCGAAACCTACACGCCAACGGAGCATCCTTT
TTCTTCATTTGCATTTACCTGCACATCGGACGAGGCCTTTACTTCGGCTCCTATCT
CTGTAAAGAGACATGAAATGTCGGGGTACTGCTTCTACTGTTAGTCATAATAACC
GCTTTCGTGGGCTATGTCCTCCCATGAGGACAAATATCGTTTTGAGGGGCCACTG
TCATCACCAACCTTCTATCAGCAGTTCCCTACATTGGAGACTCCCTGGTCCAATGA
ATTTGAGGGGGCTTCTCAGTTGACAACGCCACCTTAACCCGATTCTTTGCCTTCCA
CTTCCTTCTCCCGTTCGTCATCGCAGCAATAGCTTTAATACACCTAATTTTCCTGC
ATGAAACAGGCTCCACAAACCCAATCGGACTAAACCCAAATACAGATAAAATTT
CTTTTCACCCATTTTACTCCCTCAAAGACCTCCTTGGCTTCCTAATCTTACTCATAG
CCCTCATATTTGGTGCTTGAGCTGGAATAGTAGGTACCGCATTAAGCATACTAAT
CCGAGCAGAGCTTACCCAGCCCGGCTCCTTTTTCGGAGACGACCAACTTTATAAT
GTAGTCGTAACTGCACACGCCTTTGTAATAATCTTTTTTATAGTCATACCAATTAT
AATTGGCGGCTTTGGTAATTGACTAATCCCGCTAATAATCGGCGCCCCAGACATG
GCCTTCCCTCGTATGAATAACATAAGCTTTTGACTACTCCCCCCGTCCTTCCTCCT
TCTCCTCGCCTCCTCGACTGTTGAAGCTGGCGTAGGAACCGGCTGAACTGTTTATC
CTCCCCTCTCCGGGAACCTGGCTCACGACGGCCCATCCGTGGACCTGGCCATTTT
CTCCCTCCATCTAGCAGGGGTCTCCTCAATTTTAGGTGCAATCAACTTTATTACAA
CTGTCATCAATATGAAACCCCCAGCCACCTCCATATATAGTGCGCCCTTATTTATC
TGATCCCTCCTCATTACTGCTGTGCTTCTACTTCTTTCACTACCAGTACTTGCTGCT
GGTATTACCATACTTCTGACAGATCgaAACCT"/>

<sequence id="seq_Aata_Mito_Apisto_301991"
taxon="Aata_Mito_Apisto_301991" totalcount="4"
value="CGCTAATTGACCTCCCCGCCCCCTCCAACATTTCATCATGATGAAATTTTG
GGTCCCTGCTAGGCCTCTGCCTAATCTCCCAGATCCTAACAGGCCTATTTCTTTCC
ATACACTACACTGCCGACATCAACACAGCTTTTCATCCGTTGCCCACATCTGTCG
AGATGTAAACTACGGATGACTAATCCGAAACCTACACGCCAACGGAGCATCCTTT
TTCTTCATTTGCATTTACCTGCACATCGGACGAGGCCTTTACTTCGGCTCCTATCT
CTGTAAAGAGACATGAAATGTCGGGGTACTGCTTCTACTGTTAGTCATAATAACC
GCTTTCGTGGGCTATGTCCTCCCGTGAGGACAAATATCGTTTTGAGGGGCCACTG
TCATCACCAACCTTCTATCAGCAGTTCCCTACATCGGAGACTCCCTGGTCCAATG
AATTTGAGGGGGCTTCTCAGTTGACAACGCCACCTTAACCCGATTCTTTGCCTTCC
ACTTCCTTCTCCCGTTCGTCATCGCAGCAATAGCTTTAATACACCTAATTTTCCTG
CATGAAACAGGCTCCACAAACCCAATCGGACTAAACCCAAATACAGATAAAATT
TCTTTTCACCCATTTTACTCCCTCAAAGACCTCCTTGGCTTCCTAATCTTACTCATA
GCCCTCATATTTGGTGCTTGAGCTGGAATAGTAGGTACCGCATTAAGCATACTAA
TCCGAGCAGAGCTTACCCAGCCCGGCTCCTTTTTCGGAGACGACCAACTTTATAA
TGTAGTCGTAACTGCACACGCCTTTGTAATAATCTTTTTTATAGTCATACCAATTA
TAATTGGCGGCTTTGGTAATTGACTAATCCCGCTAATAATCGGCGCCCCAGACAT
GGCCTTCCCTCGTATGAATAACATAAGCTTTTGACTACTCCCCCCGTCCTTCCTCC
TTCTCCTCGCCTCCTCGACTGTTGAAGCTGGCGTAGGAACCGGCTGAACTGTTTAT
CCTCCCCTCTCCGGGAACCTGGCTCACGACGGCCCATCCGTGGACCTGGCCATTT
TCTCCCTCCATCTAGCAGGGGTCTCCTCAATTTTAGGTGCAATCAACTTTATTACA
ACTGTCATCAATATGAAACCCCCAGCCACCTCCATATATAGTGCGCCCTTATTTAT
CTGATCCCTCCTCATTACTGCTGTGCTTCTACTTCTTTCACTACCAGTACTTGCTGC
TGGTATTACCATACTTCTGACAGATCGAAACCT"/>
<sequence id="seq_Aata_Mito_Apisto_302106"
taxon="Aata_Mito_Apisto_302106" totalcount="4"
value="CGCTAATTGACCTCCCCGCCCCCTCCAACATTTCATCATGATGAAATTTTG
GGTCCCTGCTAGGCCTCTGCCTAATCTCCCAGATCCTAACAGGCCTATTTCTTTCC
ATACACTACACTGCCGACATCAACACAGCTTTTCATCCGTTGCCCACATCTGTCG
AGATGTAAACTACGGATGACTAATCCGAAACCTACACGCCAACGGAGCATCCTTT
TTCTTCATTTGCATTTACCTGCACATCGGACGAGGCCTTTACTTCGGCTCCTATCT
CTGTAAAGAGACATGAAATGTCGGGGTACTGCTTCTACTGTTAGTCATAATAACC
GCTTTCGTGGGCTATGTCCTCCCGTGAGGACAAATATCGTTTTGAGGGGCCACTG
TCATCACCAACCTTCTATCAGCAGTTCCCTACATCGGAGACTCCCTGGTCCAATG
AATTTGAGGGGGCTTCTCAGTTGACAACGCCACCTTAACCCGATTCTTTGCCTTCC
ACTTCCTTCTCCCGTTCGTCATCGCAGCAATAGCTTTAATACACCTAATTTTCCTG
CATGAAACAGGCTCCACAAACCCAATCGGACTAAACCCAAATACAGATAAAATT
TCTTTTCACCCATTTTACTCCCTCAAAGACCTCCTTGGCTTCCTAATCTTACTCATA
GCCCTCATATTTGGTGCTTGAGCTGGAATAGTAGGTACCGCATTAAGCATACTAA
TCCGAGCAGAGCTTACCCAGCCCGGCTCCTTTTTCGGAGACGACCAACTTTATAA
TGTAGTCGTAACTGCACACGCCTTTGTAATAATCTTTTTTATAGTCATACCAATTA
TAATTGGCGGCTTTGGTAATTGACTAATCCCGCTAATAATCGGCGCCCCAGACAT
GGCCTTCCCTCGTATGAATAACATAAGCTTTTGACTACTCCCCCCGTCCTTCCTCC
TTCTCCTCGCCTCCTCGACTGTTGAAGCTGGCGTAGGAACCGGCTGAACTGTTTAT
CCTCCCCTCTCCGGGAACCTGGCTCACGACGGCCCATCCGTGGACCTGGCCATTT
TCTCCCTCCATCTAGCAGGGGTCTCCTCAATTTTAGGTGCAATCAACTTTATTACA
ACTGTCATCAATATGAAACCCCCAGCCACCTCCATATATAGTGCGCCCTTATTTAT
CTGATCCCTCCTCATTACTGCTGTGCTTCTACTTCTTTCACTACCAGTACTTGCTGC
TGGTATTACCATACTTCTGACAGATCGAAACCT"/>

<sequence id="seq_Aata_Mito_Apisto_302107"
taxon="Aata_Mito_Apisto_302107" totalcount="4"
value="CGCTAATTGACCTCCCCGCCCCCTCCAACATTTCATCATGATGAAATTTTG
GGTCCCTGCTAGGCCTCTGCCTAATCTCCCAGATCCTAACAGGCCTATTTCTTTCC
ATACACTACACTGCCGACATCAACACAGCTTTTCATCCGTTGCCCACATCTGTCG
AGATGTAAACTACGGATGACTAATCCGAAACCTACACGCCAACGGAGCATCCTTT
TTCTTCATTTGCATTTACCTGCACATCGGACGAGGCCTTTACTTCGGCTCCTATCT
CTGTAAAGAGACATGAAATGTCGGGGTACTGCTTCTACTGTTAGTCATAATAACC
GCTTTCGTGGGCTATGTCCTCCCGTGAGGACAAATATCGTTTTGAGGGGCCACTG
TCATCACCAACCTTCTATCAGCAGTTCCCTACATCGGAGACTCCCTGGTCCAATG
AATTTGAGGGGGCTTCTCAGTTGACAACGCCACCTTAACCCGATTCTTTGCCTTCC
ACTTCCTTCTCCCGTTCGTCATCGCAGCAATAGCTTTAATACACCTAATTTTCCTG
CATGAAACAGGCTCCACAAACCCAATCGGACTAAACCCAAATACAGATAAAATT
TCTTTTCACCCATTTTACTCCCTCAAAGACCTCCTTGGCTTCCTAATCTTACTCATA
GCCCTCATATTTGGTGCTTGAGCTGGAATAGTAGGTACCGCATTAAGCATACTAA
TCCGAGCAGAGCTTACCCAGCCCGGCTCCTTTTTCGGAGACGACCAACTTTATAA
TGTAGTCGTAACTGCACACGCCTTTGTAATAATCTTTTTTATAGTCATACCAATTA
TAATTGGCGGCTTTGGTAATTGACTAATCCCGCTAATAATCGGCGCCCCAGACAT
GGCCTTCCCTCGTATGAATAACATAAGCTTTTGACTACTCCCCCCGTCCTTCCTCC
TTCTCCTCGCCTCCTCGACTGTTGAAGCTGGCGTAGGAACCGGCTGAACTGTTTAT
CCTCCCCTCTCCGGGAACCTGGCTCACGACGGCCCATCCGTGGACCTGGCCATTT
TCTCCCTCCATCTAGCAGGGGTCTCCTCAATTTTAGGTGCAATCAACTTTATTACA
ACTGTCATCAATATGAAACCCCCAGCCACCTCCATATATAGTGCGCCCTTATTTAT
CTGATCCCTCCTCATTACTGCTGTGCTTCTACTTCTTTCACTACCAGTACTTGCTGC
TGGTATTACCATACTTCTGACAGATCGAAACCT"/>
<sequence id="seq_Abae_Mito_Apisto_301654"
taxon="Abae_Mito_Apisto_301654" totalcount="4"
value="CACTAATTGACCTTCCCGCCCCCTCCAACATCTCCTCTTGATGAAATTTTG
GATCCCTACTGGGCCTCTGCTTGATTTCCCAAGTCCTAACAGGTTTATTCCTCTCC
ATACACTATACTGCTGACATCAACACAGCTTTCTCATCCGTCACCCACATCTGCCG
AGACGTAAACTACGGATGACTGATTCGGAACCTACATGCTAACGGAGCATCTTTT
TTCTTTATTTGCATCTACTTGCACATCGGACGGGGCCTTTACTTCGGCTCCTACCT
CTACAAAGAAACATGAAACATTGGAGTAGTTCTCCTGCTGCTAGTTATAATAACC
GCTTTTGTAGGCTATGTCCTCCCATGAGGACAAATATCGTTTTGAGGGGCCACCG
TCATCACCAACCTGCTTTCAGCCATCCCTTACATGGGGGACTCCCTAGTCCAATG
AATTTGAGGCGGCTTCTCAGTTGACAACGCTACCCTGACCCGGTTCTTTGCCATCC
ATTTCCTTCTCCCATTCATCATCACAGCCATAACCTTGATTCACCTAATCTTTCTCC
ACGAAACAGGCTCTACAAACCCAATCGGATTAAGCCCAGACACAGATAAGATTT
CTTTCCACCCATTCTACTCCCTCAAAGACCTCCTCGGCTTTCTAATTTTACTCACA
GCTCTTATATTTGGTGCCTGAGCTGGAATAGCAGGCACCGCACTAAGCATGCTTA
TTCGAGCAGAACTTACTCAACCCGGCTCCTTTTTCGGAGACGACCAAACCTACAA
TGTAATCGTAACTGCACACGCCTTTGTAATAATCTTTTTTATAGTAATACCAGTTA
TGATCGGTGGCTTTGGAAATTGACTAATCCCGCTGATAATCGGCGCCCCGGACAT
GGCTTTCCCTCGAATAAATAACATAAGCTTTTGACTACTCCCCCCATCTTTCCTCC
TCCTCCTAGCCTCTTCGACTGTTGAAGCTGGCGTAGGAACAGGCTGGACCGTGTA
CCCGCCCCTTTCCGGAAATTTAGCCCACGACGGCCCGTCCGTGGACCTGGCCATT
TTCTCCCTTCATCTAGCCGGAATCTCCTCTATTTTAGGCGCAATCAACTTTATCAC
CACCATCATTAACATGAAACCTCCAGCCATCTCCATGTACACTACACCCCTATTTA
TCTGATCTCTTCTTATTACTGCGGTACTTCTACTTCTATCACTACCAGTTCTTGCTG
CTGGCATCACTATACTCCTCACAGACCGTAACTT"/>

<sequence id="seq_Abae_Mito_Apisto_301676"
taxon="Abae_Mito_Apisto_301676" totalcount="4"
value="CACTAATTGACCTTCCCGCCCCCTCCAACATCTCCTCTTGATGAAATTTTG
GGTCCCTACTGGGCCTCTGCTTGATTTCCCAAGTCCTAACAGGTTTATTCCTCTCC
ATACACTATACTGCTGACATCAACACAGCTTTCTCATCCGTCACCCACATCTGCCG
AGATGTAAACTACGGATGACTGATTCGGAACCTACATGCTAACGGAGCATCTTTT
TTCTTTATTTGCATCTACTTGCACATCGGACGGGGCCTTTACTTCGGCTCCTACCT
TTACAAAGAAACATGAAACATTGGAGTAGTTCTCCTGCTGCTAGTTATAATAACC
GCTTTTGTAGGCTATGTCCTCCCATGAGGACAAATATCGTTTTGAGGGGCCACCG
TCATCACCAACCTGCTTTCAGCCATCCCTTACATCGGGGATTACCTAGTCCAATGA
ATTTGAGGCGGCTTCTCAGTTGACAACGCTACCCTGACCCGGTTCTTTGCCATCCA
CTTCCTTCTCCCATTCATCATCACAGCCATAACCTTGATTCACCTAATCTTTCTCCA
CGAAACAGGCTCTACAAACCCAATTGGATTAAACCCAAACACAGATAAAATTTCT
TTCCACCCATTCTACTCCCTCAAAGACCTCCTCGGCTTTATAATTTTACTCACAGC
TCTTATATTTGGTGCCTGAGCTGGAATAGCAGGCACCGCACTAAGCATGCTTATT
CGAGCAGAACTTACTCAACCCGGCTCCTTTTTCGGAGACGACCAAACCTACAATG
TAATCGTAACTGCACACGCCTTTGTAATAATCTTTTTTATAGTAATACCAGTTATG
ATCGGTGGCTTTGGAAATTGACTAATCCCGCTGATAATCGGCGCCCCGGACATGG
CTTTCCCTCGAATAAATAACATAAGCTTTTGACTACTCCCCCCATCTTTCCTCCTC
CTCCTAGCCTCTTCGACTGTTGAAGCTGGCGTAGGAACAGGCTGGACCGTGTACC
CGCCCCTTTCCGGAAATTTAGCCCACGACGGCCCGTCCGTGGACCTGGCCATTTT
CTCCCTTCATCTAGCCGGAATCTCCTCTATTTTAGGCGCAATCAACTTTATCACCA
CCATCATTAACATGAAACCTCCAGCCATCTCCATGTACACTACACCCCTATTTATC
TGATCTCTTCTTATTACTGCGGTACTTCTACTTCTATCACTACCAGTTCTTGCTGCT
GGCATCACTATACTCCTCACAGACCGTAACTT"/>
<sequence id="seq_Abae_Mito_Apisto_301677"
taxon="Abae_Mito_Apisto_301677" totalcount="4"
value="CACTAATTGACCTTCCCGCCCCCTCCAACATCTCCTCTTGATGAAATTTTG
GATCCCTACTGGGCCTCTGCTTGATTTCCCAAGTCCTAACAGGTTTATTCCTCTCC
ATACACTATACTGCTGACATCAACACAGCTTTCTCATCCGTCACCCACATCTGCCG
AGATGTAAACTACGGATGACTGATTCGGAACCTACATGCTAACGGAGCATCTTTT
TTCTTTATTTGCATCTACTTGCACATCGGACGGGGCCTTTACTTCGGCTCCTACCT
CTACAAAGAAACATGAAACATTGGAGTAGTTCTCCTGCTGCTAGTTATAATAACC
GCTTTTGTAGGCTATGTCCTCCCATGAGGACAAATATCGTTTTGAGGGGCCACCG
TCATCACCAACCTGCTTTCAGCCATCCCTTACATGGGGGACTCCCTAGTCCAATG
AATTTGAGGCGGCTTCTCAGTTGACAACGCTACCCTGACCCGGTTCTTTGCCATCC
ATTTCCTTCTCCCATTCATCATCACAGCCATAACCTTGATTCACCTAATCTTTCTCC
ACGAAACAGGCTCTACAAACCCAATCGGATTAAACCCAAACACAGATAAGATTT
CTTTCCACCCATTCTACTCCCTCAAAGACCTCCTCGGCTTTCTAATTTTACTCACA
GCTCTTATATTTGGTGCCTGAGCTGGAATAGCAGGCACCGCACTAAGCATGCTTA
TTCGAGCAGAACTTACTCAACCCGGCTCCTTTTTCGGAGACGACCAAACCTACAA
TGTAATCGTAACTGCACACGCCTTTGTAATAATCTTTTTTATAGTAATACCAGTTA
TGATCGGTGGCTTTGGAAATTGACTAATCCCGCTGATAATCGGCGCCCCGGACAT
GGCTTTCCCTCGAATAAATAACATAAGCTTTTGACTACTCCCCCCATCTTTCCTCC
TCCTCCTAGCCTCTTCGACTGTTGAAGCTGGCGTAGGAACAGGCTGGACCGTGTA
CCCGCCCCTTTCCGGAAATTTAGCCCACGACGGCCCGTCCGTGGACCTGGCCATT
TTCTCCCTTCATCTAGCCGGAATCTCCTCTATTTTAGGCGCAATCAACTTTATCAC
CACCATCATTAACATGAAACCTCCAGCCATCTCCATGTACACTACACCCCTATTTA
TCTGATCTCTTCTTATTACTGCGGTACTTCTACTTCTATCACTACCAGTTCTTGCTG
CTGGCATCACTATACTCCTCACAGACCGTAACTT"/>

<sequence id="seq_Abae_Mito_Apisto_301678"
taxon="Abae_Mito_Apisto_301678" totalcount="4"
value="CACTAATTGACCTTCCCGCCCCCTCCAACATCTCCTCTTGATGAAATTTTG
GATCCCTACTGGGCCTCTGCTTGATTTCCCAAGTCCTAACAGGTTTATTCCTCTCC
ATACACTATACTGCTGACATCAACACAGCTTTCTCATCCGTCACCCACATCTGCCG
AGACGTAAACTACGGATGACTGATTCGGAACCTACATGCTAACGGAGCATCTTTT
TTCTTTATTTGCATCTACTTGCACATCGGACGGGGCCTTTACTTCGGCTCCTACCT
CTACAAAGAAACATGAAACATTGGAGTAGTTCTCCTGCTGCTAGTTATAATAACC
GCTTTTGTAGGCTATGTCCTCCCATGAGGACAAATATCGTTTTGAGGGGCCACCG
TCATCACCAACCTGCTTTCAGCCATCCCTTACATGGGGGACTCCCTAGTCCAATG
AATTTGAGGCGGCTTCTCAGTTGACAACGCTACCCTGACCCGGTTCTTTGCCATCC
ATTTCCTTCTCCCATTCATCATCACAGCCATAACCTTGATTCACCTAATCTTTCTCC
ACGAAACAGGCTCTACAAACCCAATCGGATTAAGCCCAGACACAGATAAAATTT
CTTTCCACCCATTCTACTCCCTCAAAGACCTCCTCGGTTTTCTAATTTTACTCACA
GCTCTTATATTTGGTGCCTGGGCTGGAATAGCAGGCACCGCACTAAGCATGCTTA
TTCGAGCAGAACTTACTCAACCCGGCTCCTTTTTCGGAGACGACCAAACCTACAA
TGTAATCGTAACTGCACACGCCTTTGTAATAATCTTTTTTATAGTAATACCAGTTA
TGATCGGTGGGTTTGGAAATTGACTAATCCCGCTGATAATCGGCGCCCCGGACAT
GGCTTTCCCTCGAATAAATAACATAAGCTTTTGACTACTCCCCCCATCTTTCCTCC
TCCTCCTAGCCTCTTCGACTGTTGAAGCTGGCGTAGGAACAGGCTGGACCGTGTA
CCCGCCCCTTTCCGGAAATTTAGCCCACGACGGCCCGTCCGTAGACCTGGCCATT
TTCTCCCTTCATCTAGCCGGAATCTCCTCTATTTTAGGCGCAATCAACTTTATCAC
CACCATCATTAACATGAAACCTCCAGCCATCTCCATGTACACTACACCCCTATTTA
TCTGATCTCTCCTTATTACTGCAGTACTTCTACTTCTATCACTACCAGTTCTTGCTG
CTGGAATCACTATACTCCTCACAGACCGTAACTT"/>
<sequence id="seq_Abae_Mito_Apisto_301679"
taxon="Abae_Mito_Apisto_301679" totalcount="4"
value="CACTAATTGACCTTCCCGCCCCCTCCAACATCTCCTCTTGATGAAATTTTG
GATCCCTACTGGGCCTCTGCTTGATTTCCCAAGTCCTAACAGGTTTATTCCTCTCC
ATACACTATACTGCTGACATCAACACAGCTTTCTCATCCGTCACCCACATCTGCCG
AGACGTAAACTACGGATGACTGATTCGGAACCTACATGCTAACGGAGCATCTTTT
TTCTTTATTTGCATCTACTTGCACATCGGACGGGGCCTTTACTTCGGCTCCTACCT
CTACAAAGAAACATGAAACATTGGAGTAGTTCTCCTGCTGCTAGTTATAATAACC
GCTTTTGTAGGCTATGTCCTCCCATGAGGACAAATATCGTTTTGAGGGGCCACCG
TCATCACCAACCTGCTTTCAGCCATCCCTTACATGGGGGACTCCCTAGTCCAATG
AATTTGAGGCGGCTTCTCAGTTGACAACGCTACCCTGACCCGGTTCTTTGCCATCC
ATTTCCTTCTCCCATTCATCATCACAGCCATAACCTTGATTCACCTAATCTTTCTCC
ACGAAACAGGCTCTACAAACCCCATCGGATTAAGCCCAGACACAGATAAGATTT
CTTTCCACCCATTCTACTCCCTCAAAGACCTCCTCGGCTTTCTAATTTTACTCACA
GCTCTTATATTTGGTGCCTGAGCTGGAATAGCAGGCACCGCACTAAGCATGCTTA
TTCGAGCAGAACTTACTCAACCCGGCTCCTTTTTCGGAGACGACCAAACCTACAA
TGTAATCGTAACTGCACACGCCTTTGTAATAATCTTTTTTATAGTAATACCAATTA
TGATCGGTGGGTTTGGAAATTGACTAATCCCGCTGATAATCGGCGCCCCGGACAT
GGCTTTCCCTCGAATAAATAACATAAGCTTTTGACTACTCCCCCCATCTTTCCTCC
TCCTCCTAGCCTCTTCGACTGTTGAAGCTGGCGTAGGAACAGGCTGAACCGTGTA
CCCGCCCCTTTCCGGAAATTTAGCCCACGACGGCCCGTCCGTAGACCTGGCCATT
TTCTCCCTTCATCTAGCCGGAATCTCCTCTATTTTAGGCGCAATCAACTTTATCAC
CACCATCATTAACATGAAACCTCCAGCCATCTCCATGTACACTACACCCCTATTTA
TCTGATCTCTCCTTATTACTGCGGTACTTCTACTTCTATCACTACCAGTTCTTGCTG
CTGGCATCACTATACTCCTCACAGACCGTAACTT"/>

<sequence id="seq_Abar_Mito_Apisto_302256"
taxon="Abar_Mito_Apisto_302256" totalcount="4"
value="CCCTAATTGATCTTCCCACCCCCTCCAACATTTCCTCTTGATGAAATTTTGG
GTCTCTACTAGGCCTCTGCTTAATTTCCCAAATTCTTACAGGCCTATTTCTTTCCAT
ACACTATTCTGCTGACATCAACACAGCTTTTTCATCCGTTGCCCACATCTCTCGAG
ACGTAAACTACGGATGGCTAATCCGCAATTTACACGCTAACGGAGCATCCTTTTT
CTTCATTTGCATCTATCTTCACATCGGACGGGGCCTTTACTTCGGATCTTACCTCT
ACAAAGAGACATGAAACATTGGAGTAGTACTTCTACTCCTAGTTATAATAACCGC
TTTTGTGGGCTACGTCCTCCCCTGAGGCCAAATATCGTTTTGAGGAGCTACCGTCA
TCACCAACCTACTGTCAGCAATCCCCTACATTGGAAACTCCCTAGTCCAATGAAT
TTGAGGTGGCTTCTCAGTTGACAACGCTACCCTAACCCGATTCTTTGCCATCCACT
TCCTACTTCCATTCGTCATCGCAGCCGTAACCCTAATACACCTAATTTTTCTCCAC
GAAACAGGCTCCACCAACCCAATCGGACTGACCTCCAACACGGATAAAATTTCTT
TCCACCCATTTTTCTCCCTCAAAGACCTCCTCGGATTCCTAATTTTACTTATAACTC
TGATATTTGGTGCTTGAGCTGGAATAGCAGGTACCGCATTAAGCATGCTAATTCG
AGCAGAACTTACCCAACCCGGCTCCTTTTTTGGGGACGATCAAGTATATAATGTA
ATCGTAACTGCACACGCTTTCGTAATAATCTTCTTTATAGTAATACCAATTATAAT
CGGTGGATTTGGCAACTGACTAATTCCACTAATAATCGGCGCCCCAGACATGGCT
TTCCCTCGTATGAACAACATAAGCTTTTGACTGCTCCCCCCATCTTTCCTCCTCCT
CCTCGCCTCCTCAACTGTTGAAGCCGGGGTAGGGACAGGCTGGACTGTGTACCCG
CCTCTTTCCGGGAATTTGGCCCACGACGGCCCATCCGTGGACCTGGCCATCTTTTC
CCTCCACTTAGCGGGGGTGTCCTCAATTTTAGGTGCAATCAACTTTATCACTACTA
TCATTAACATGAAACCCCCAGCCACCTCTTTATACAATACCCCCCTGTTTATCTGA
TCCCTCCTCGTCACTGCTGTGCTTCTACTTCTTTCACTACCAGTTCTTGCTGCTGGT
ATCACTATACTTCTAACGGACCGAAACCT"/>
<sequence id="seq_Abar_Mito_Apisto_302257"
taxon="Abar_Mito_Apisto_302257" totalcount="4"
value="CCCTAATTGATCTTCCCACCCCCTCCAACATTTCCTCTTGATGAAATTTTGG
GTCTCTACTAGGCCTCTGCTTAATTTCCCAAATTCTTACAGGCCTATTTCTTTCCAT
ACACTATTCTGCTGACATCAACACAGCTTTTTCATCCGTTGCCCACATCTCTCGAG
ACGTAAACTACGGATGGCTAATCCGCAATTTACACGCTAACGGAGCATCCTTTTT
CTTCATTTGCATCTATCTTCACATCGGACGGGGCCTTTACTTCGGATCTTACCTCT
ACAAAGAGACATGAAACATTGGAGTAGTACTTCTACTCCTAGTTATAATAACCGC
TTTTGTGGGCTACGTCCTCCCCTGAGGCCAAATATCGTTTTGAGGAGCTACCGTCA
TCACCAACCTACTGTCAGCAATCCCCTACATTGGAAACTCCCTAGTCCAATGAAT
TTGAGGTGGCTTCTCAGTTGACAACGCTACCCTAACCCGATTCTTTGCCATCCACT
TCCTACTTCCATTCGTCATCGCAGCCGTAACCCTAATACACCTAATTTTTCTCCAC
GAAACAGGCTCCACCAACCCAATCGGACTGACCTCCAACACGGATAAAATTTCTT
TCCACCCATTTTTCTCCCTCAAAGACCTCCTCGGATTCCTAATTTTACTTATAACTC
TGATATTTGGTGCTTGAGCTGGAATAGCAGGTACCGCATTAAGCATGCTAATTCG
AGCAGAACTTACCCAACCCGGCTCCTTTTTTGGGGACGATCAAGTATATAATGTA
ATCGTAACTGCACACGCTTTCGTAATAATCTTCTTTATAGTAATACCAATTATAAT
CGGTGGATTTGGCAACTGACTAATTCCACTAATAATCGGCGCCCCAGACATGGCT
TTCCCTCGTATGAACAACATAAGCTTTTGACTGCTCCCCCCATCTTTCCTCCTCCT
CCTCGCCTCCTCAACTGTTGAAGCCGGGGTAGGGACAGGCTGGACTGTGTACCCG
CCTCTTTCCGGGAATTTGGCCCACGACGGCCCATCCGTGGACCTGGCCATCTTTTC
CCTCCACTTAGCGGGGGTGTCCTCAATTTTAGGTGCAATCAACTTTATCACTACTA
TCATTAACATGAAACCCCCAGCCACCTCTTTATACAATACCCCCCTGTTTATCTGA
TCCCTCCTCGTCACTGCTGTGCTTCTACTTCTTTCACTACCAGTTCTTGCTGCTGGT
ATCACTATACTTCTAACGGACCGAAACCT"/>

<sequence id="seq_Abar_Mito_Apisto_302258"
taxon="Abar_Mito_Apisto_302258" totalcount="4"
value="CCCTAATTGATCTTCCCACCCCCTCCAACATTTCCTCTTGATGAAATTTTGG
GTCTCTACTAGGCCTCTGCTTAATTTCCCAAATTCTTACAGGCCTATTTCTTTCCAT
ACACTATTCTGCTGACATCAACACAGCTTTTTCATCCGTTGCCCACATCTCTCGAG
ACGTAAACTACGGATGGCTAATCCGCAATTTACACGCTAACGGAGCATCCTTTTT
CTTCATTTGCATCTATCTTCACATCGGACGGGGCCTTTACTTCGGATCTTACCTCT
ACAAAGAGACATGAAACATTGGAGTAGTACTTCTACTCCTAGTTATAATAACCGC
TTTTGTGGGCTACGTCCTCCCCTGAGGCCAAATATCGTTTTGAGGAGCTACCGTCA
TCACCAACCTACTGTCAGCAATCCCCTACATTGGAAACTCCCTAGTCCAATGAAT
TTGAGGTGGCTTCTCAGTTGACAACGCTACCCTAACCCGATTCTTTGCCATCCACT
TCCTACTTCCATTCGTCATCGCAGCCGTAACCCTAATACACCTAATTTTTCTCCAC
GAAACAGGCTCCACCAACCCAATCGGACTGACCTCCAACACGGATAAAATTTCTT
TCCACCCATTTTTCTCCCTCAAAGACCTCCTCGGATTCCTAATTTTACTTATAACTC
TGATATTTGGTGCTTGAGCTGGAATAGCAGGTACCGCATTAAGCATGCTAATTCG
AGCAGAACTTACCCAACCCGGCTCCTTTTTTGGGGACGATCAAGTATATAATGTA
ATCGTAACTGCACACGCTTTCGTAATAATCTTCTTTATAGTAATACCAATTATAAT
CGGTGGATTTGGCAACTGACTAATTCCACTAATAATCGGCGCCCCAGACATGGCT
TTCCCTCGTATGAACAACATAAGCTTTTGACTGCTCCCCCCATCTTTCCTCCTCCT
CCTCGCCTCCTCAACTGTTGAAGCCGGGGTAGGGACAGGCTGGACTGTGTACCCG
CCTCTTTCCGGGAATTTGGCCCACGACGGCCCATCCGTGGACCTGGCCATCTTTTC
CCTCCACTTAGCGGGGGTGTCCTCAATTTTAGGTGCAATCAACTTTATCACTACTA
TCATTAACATGAAACCCCCAGCCACCTCTTTATACAATACCCCCCTGTTTATCTGA
TCCCTCCTCGTCACTGCTGTGCTTCTACTTCTTTCACTACCAGTTCTTGCTGCTGGT
ATCACTATACTTCTAACGGACCGAAACCT"/>
<sequence id="seq_Abar_Mito_Apisto_302259"
taxon="Abar_Mito_Apisto_302259" totalcount="4"
value="CCCTAATTGATCTTCCCACCCCCTCCAACATTTCCTCTTGATGAAATTTTGG
GTCTCTACTAGGCCTCTGCTTAATTTCCCAAATTCTTACAGGCCTATTTCTTTCCAT
ACACTATTCTGCTGACATCAACACAGCTTTTTCATCCGTTGCCCACATCTCTCGAG
ACGTAAACTACGGATGGCTAATCCGCAATTTACACGCTAACGGAGCATCCTTTTT
CTTCATTTGCATCTATCTTCACATCGGACGGGGCCTTTACTTCGGATCTTACCTCT
ACAAAGAGACATGAAACATTGGAGTAGTACTTCTACTCCTAGTTATAATAACCGC
TTTTGTGGGCTACGTCCTCCCCTGAGGCCAAATATCGTTTTGAGGAGCTACCGTCA
TCACCAACCTACTGTCAGCAATCCCCTACATTGGAAACTCCCTAGTCCAATGAAT
TTGAGGTGGCTTCTCAGTTGACAACGCTACCCTAACCCGATTCTTTGCCATCCACT
TCCTACTTCCATTCGTCATCGCAGCCGTAACCCTAATACACCTAATTTTTCTCCAC
GAAACAGGCTCCACCAACCCAATCGGACTGACCTCCAACACGGATAAAATTTCTT
TCCACCCATTTTTCTCCCTCAAAGACCTCCTCGGATTCCTAATTTTACTTATAACTC
TGATATTTGGTGCTTGAGCTGGAATAGCAGGTACCGCATTAAGCATGCTAATTCG
AGCAGAACTTACCCAACCCGGCTCCTTTTTTGGGGACGATCAAGTATATAATGTA
ATCGTAACTGCACACGCTTTCGTAATAATCTTCTTTATAGTAATACCAATTATAAT
CGGTGGATTTGGCAACTGACTAATTCCACTAATAATCGGCGCCCCAGACATGGCT
TTCCCTCGTATGAACAACATAAGCTTTTGACTGCTCCCCCCATCTTTCCTCCTCCT
CCTCGCCTCCTCAACTGTTGAAGCCGGGGTAGGGACAGGCTGGACTGTGTACCCG
CCTCTTTCCGGGAATTTGGCCCACGACGGCCCATCCGTGGACCTGGCCATCTTTTC
CCTCCACTTAGCGGGGGTGTCCTCAATTTTAGGTGCAATCAACTTTATCACTACTA
TCATTAACATGAAACCCCCAGCCACCTCTTTATACAATACCCCCCTGTTTATCTGA
TCCCTCCTCGTCACTGCTGTGCTTCTACTTCTTTCACTACCAGTTCTTGCTGCTGGT
ATCACTATACTTCTAACGGACCGAAACCT"/>

<sequence id="seq_Abar_Mito_Apisto_302896"
taxon="Abar_Mito_Apisto_302896" totalcount="4"
value="CCCTAATTGATCTTCCCACCCCCTCCAACATTTCCTCTTGATGAAATTTTGG
GTCTCTACTAGGCCTCTGCTTAATTTCCCAAATTCTTACAGGCCTATTTCTTTCCAT
ACACTATTCTGCTGACATCAACACAGCTTTTTCATCCGTTGCCCACATCTCTCGAG
ACGTAAACTACGGATGGCTAATCCGCAATTTACACGCTAACGGAGCATCCTTTTT
CTTCATTTGCATCTATCTTCACATCGGACGGGGCCTTTACTTCGGATCTTACCTCT
ACAAAGAGACATGAAACATTGGAGTAGTACTTCTACTCCTAGTTATAATAACCGC
TTTTGTGGGCTACGTCCTCCCCTGAGGCCAAATATCGTTTTGAGGAGCTACCGTCA
TCACCAACCTACTGTCAGCAATCCCCTACATTGGAAACTCCCTAGTCCAATGAAT
TTGAGGTGGCTTCTCAGTTGACAACGCTACCCTAACCCGATTCTTTGCCATCCACT
TCCTACTTCCATTCGTCATCGCAGCCGTAACCCTAATACACCTAATTTTTCTCCAC
GAAACAGGCTCCACCAACCCAATCGGACTGACCTCCAACACGGATAAAATTTCTT
TCCACCCATTTTTCTCCCTCAAAGACCTCCTCGGATTCCTAATTTTACTTATAACTC
TGATATTTGGTGCTTGAGCTGGAATAGCAGGTACCGCATTAAGCATGCTAATTCG
AGCAGAACTTACCCAACCCGGCTCCTTTTTTGGGGACGATCAAGTATATAATGTA
ATCGTAACTGCACACGCTTTCGTAATAATCTTCTTTATAGTAATACCAATTATAAT
CGGTGGATTTGGCAACTGACTAATTCCACTAATAATCGGCGCCCCAGACATGGCT
TTCCCTCGTATGAACAACATAAGCTTTTGACTGCTCCCCCCATCTTTCCTCCTCCT
CCTCGCCTCCTCAACTGTTGAAGCCGGGGTAGGGACAGGCTGGACTGTGTACCCG
CCTCTTTCCGGGAATTTGGCCCACGACGGCCCATCCGTGGACCTGGCCATCTTTTC
CCTCCACTTAGCGGGGGTGTCCTCAATTTTAGGTGCAATCAACTTTATCACTACTA
TCATTAACATGAAACCCCCAGCCACCTCTTTATACAATACCCCCCTGTTTATCTGA
TCCCTCCTCGTCACTGCTGTGCTTCTACTTCTTTCACTACCAGTTCTTGCTGCTGGT
ATCACTATACTTCTAACGGACCGAAACCT"/>
<sequence id="seq_Abar_Mito_Apisto_302897"
taxon="Abar_Mito_Apisto_302897" totalcount="4"
value="CCCTAATTGATCTTCCCACCCCCTCCAACATTTCCTCTTGATGAAATTTTGG
GTCTCTACTAGGCCTCTGCTTAATTTCCCAAATTCTTACAGGCCTATTTCTTTCCAT
ACACTATTCTGCTGACATCAACACAGCTTTTTCATCCGTTGCCCACATCTCTCGAG
ACGTAAACTACGGATGGCTAATCCGCAATTTACACGCTAACGGAGCATCCTTTTT
CTTCATTTGCATCTATCTTCACATCGGACGGGGCCTTTACTTCGGATCTTACCTCT
ACAAAGAGACATGAAACATTGGAGTAGTACTTCTACTCCTAGTTATAATAACCGC
TTTTGTGGGCTACGTCCTCCCCTGAGGCCAAATATCGTTTTGAGGAGCTACCGTCA
TCACCAACCTACTGTCAGCAATCCCCTACATTGGAAACTCCCTAGTCCAATGAAT
TTGAGGTGGCTTCTCAGTTGACAACGCTACCCTAACCCGATTCTTTGCCATCCACT
TCCTACTTCCATTCGTCATCGCAGCCGTAACCCTAATACACCTAATTTTTCTCCAC
GAAACAGGCTCCACCAACCCAATCGGACTGACCTCCAACACGGATAAAATTTCTT
TCCACCCATTTTTCTCCCTCAAAGACCTCCTCGGATTCCTAATTTTACTTATAACTC
TGATATTTGGTGCTTGAGCTGGAATAGCAGGTACCGCATTAAGCATGCTAATTCG
AGCAGAACTTACCCAACCCGGCTCCTTTTTTGGGGACGATCAAGTATATAATGTA
ATCGTAACTGCACACGCTTTCGTAATAATCTTCTTTATAGTAATACCAATTATAAT
CGGTGGGTTTGGTAACTGACTAATTCCACTAATAATCGGCGCCCCAGACATGGCT
TTCCCTCGTATGAACAACATAAGCTTTTGACTGCTCCCCCCATCTTTCCTCCTCCT
CCTCGCCTCCTCAACTGTTGAAGCCGGGGTAGGGACAGGCTGGACTGTGTACCCG
CCTCTTTCCGGGAATTTGGCCCACGACGGCCCATCCGTGGACCTGGCCATCTTTTC
CCTCCACTTAGCGGGGGTGTCCTCAATTTTAGGTGCAATCAACTTTATTACTACTA
TCATTAACATGAAACCCCCAGCCACCTCTTTATACAATACCCCCCTGTTTATCTGA
TCCCTCCTCGTCACTGCTGTGCTTCTACTTCTTTCACTACCAGTTCTTGCTGCTGGT
ATCACTATACTTCTAACGGACCgAAACCT"/>

<sequence id="seq_Abar_Mito_Apisto_302899"
taxon="Abar_Mito_Apisto_302899" totalcount="4"
value="CCCTAATTGATCTTCCCACCCCCTCCAACATTTCCTCTTGATGAAATTTTGG
GTCTCTACTAGGCCTCTGCTTAATTTCCCAAATTCTTACAGGCCTATTTCTTTCCAT
ACACTATTCTGCTGACATCAACACAGCTTTTTCATCCGTTGCCCACATCTCTCGAG
ACGTAAACTACGGATGGCTAATCCGCAATTTACACGCTAACGGAGCATCCTTTTT
CTTCATTTGCATCTATCTTCACATCGGACGGGGCCTTTACTTCGGATCTTACCTCT
ACAAAGAGACATGAAACATTGGAGTAGTACTTCTACTCCTAGTTATAATAACCGC
TTTTGTGGGCTACGTCCTCCCCTGAGGCCAAATATCGTTTTGAGGAGCTACCGTCA
TCACCAACCTACTGTCAGCAATCCCCTACATTGGAAACTCCCTAGTCCAATGAAT
TTGAGGTGGCTTCTCAGTTGACAACGCTACCCTAACCCGATTCTTTGCCATCCACT
TCCTACTTCCATTCGTCATCGCAGCCGTAACCCTAATACACCTAATTTTTCTCCAC
GAAACAGGCTCCACCAACCCAATCGGACTGACCTCCAACACGGATAAAATTTCTT
TCCACCCATTTTTCTCCCTCAAAGACCTCCTCGGATTCCTAATTTTACTTATAACTC
TGATATTTGGTGCTTGAGCTGGAATAGCAGGTACCGCATTAAGCATGCTAATTCG
AGCAGAACTTACCCAACCCGGCTCCTTTTTTGGGGACGATCAAGTATATAATGTA
ATCGTAACTGCACACGCTTTCGTAATAATCTTCTTTATAGTAATACCAATTATAAT
CGGTGGGTTTGGTAACTGACTAATTCCACTAATAATCGGCGCCCCAGACATGGCT
TTCCCTCGTATGAACAACATAAGCTTTTGACTGCTCCCCCCATCTTTCCTCCTCCT
CCTCGCCTCCTCAACTGTTGAAGCCGGGGTAGGGACAGGCTGGACTGTGTACCCG
CCTCTTTCCGGGAATTTGGCCCACGACGGCCCATCCGTGGACCTGGCCATCTTTTC
CCTCCACTTAGCGGGGGTGTCCTCAATTTTAGGTGCAATCAACTTTATTACTACTA
TCATTAACATGAAACCCCCAGCCACCTCTTTATACAATACCCCCCTGTTTATCTGA
TCCCTCCTCGTCACTGCTGTGCTTCTACTTCTTTCACTACCAGTTCTTGCTGCTGGT
ATCACTATACTTCTAACGGACCGAAACCT"/>
<sequence id="seq_Abar_Mito_Apisto_302900"
taxon="Abar_Mito_Apisto_302900" totalcount="4"
value="CCCTAATTGATCTTCCCACCCCCTCCAACATTTCCTCTTGATGAAATTTTGG
GTCTCTACTAGGCCTCTGCTTAATTTCCCAAATTCTTACAGGCCTATTTCTTTCCAT
ACACTATTCTGCTGACATCAACACAGCTTTTTCATCCGTTGCCCACATCTCTCGAG
ACGTAAACTACGGATGGCTAATCCGCAATTTACACGCTAACGGAGCATCCTTTTT
CTTCATTTGCATCTATCTTCACATCGGACGGGGCCTTTACTTCGGATCTTACCTCT
ACAAAGAGACATGAAACATTGGAGTAGTACTTCTACTCCTAGTTATAATAACCGC
TTTTGTGGGCTACGTCCTCCCCTGAGGCCAAATATCGTTTTGAGGAGCTACCGTCA
TCACCAACCTACTGTCAGCAATCCCCTACATTGGAAACTCCCTAGTCCAATGAAT
TTGAGGTGGCTTCTCAGTTGACAACGCTACCCTAACCCGATTCTTTGCCATCCACT
TCCTACTTCCATTCGTCATCGCAGCCGTAACCCTAATACACCTAATTTTTCTCCAC
GAAACAGGCTCCACCAACCCAATCGGACTGACCTCCAACACGGATAAAATTTCTT
TCCACCCATTTTTCTCCCTCAAAGACCTCCTCGGATTCCTAATTTTACTTATAACTC
TGATATTTGGTGCTTGAGCTGGAATAGCAGGTACCGCATTAAGCATGCTAATTCG
AGCAGAACTTACCCAACCCGGCTCCTTTTTTGGGGACGATCAAGTATATAATGTA
ATCGTAACTGCACACGCTTTCGTAATAATCTTCTTTATAGTAATACCAATTATAAT
CGGTGGGTTTGGTAACTGACTAATTCCACTAATAATCGGCGCCCCAGACATGGCT
TTCCCTCGTATGAACAACATAAGCTTTTGACTGCTCCCCCCATCTTTCCTCCTCCT
CCTCGCCTCCTCAACTGTTGAAGCCGGGGTAGGGACAGGCTGGACTGTGTACCCG
CCTCTTTCCGGGAATTTGGCCCACGACGGCCCATCCGTGGACCTGGCCATCTTTTC
CCTCCACTTAGCGGGGGTGTCCTCAATTTTAGGTGCAATCAACTTTATTACTACTA
TCATTAACATGAAACCCCCAGCCACCTCTTTATACAATACCCCCCTGTTTATCTGA
TCCCTCCTCGTCACTGCTGTGCTTCTACTTCTTTCACTACCAGTTCTTGCTGCTGGT
ATCACTATACTTCTAACGGACCGAAACCT"/>

<sequence id="seq_Abar_Mito_Apisto_300542"
taxon="Abar_Mito_Apisto_300542" totalcount="4"
value="CCCTAATTGATCTTCCCACCCCCTCCAACATTTCCTCTTGATGAAATTTTGG
GTCTCTACTAGGCCTCTGCTTAATTTCCCAAATTCTTACAGGCCTATTTCTTTCCAT
ACACTATTCTGCTGACATCAACACAGCTTTTTCATCCGTTGCCCACATCTCTCGAG
ACGTAAACTACGGATGGCTAATCCGCAATTTACACGCTAACGGAGCATCCTTTTT
CTTCATTTGCATCTATCTTCACATCGGACGGGGCCTTTACTTCGGATCTTACCTCT
ACAAAGAGACATGAAACATTGGAGTAGTACTTCTACTCCTAGTTATAATAACCGC
TTTTGTGGGCTACGTCCTCCCCTGAGGCCAAATATCGTTTTGAGGAGCTACCGTCA
TCACCAACCTACTGTCAGCAATCCCCTACATTGGAAACTCCCTAGTCCAATGAAT
TTGAGGTGGCTTCTCAGTTGACAACGCTACCCTAACCCGATTCTTTGCCATCCACT
TCCTACTTCCATTCGTCATCGCAGCCGTAACCCTAATACACCTAATTTTTCTCCAC
GAAACAGGCTCCACCAACCCAATCGGACTGACCTCCAACACGGATAAAATTTCTT
TCCACCCATTTTTCTCCCTCAAAGACCTCCTCGGATTCCTAATTTTACTTATAACTC
TGATATTTGGTGCTTGAGCTGGAATAGCAGGTACCGCATTAAGCATGCTAATTCG
AGCAGAACTTACCCAACCCGGCTCCTTTTTTGGGGACGATCAAGTATATAATGTA
ATCGTAACTGCACACGCTTTCGTAATAATCTTCTTTATAGTAATACCAATTATAAT
CGGTGGATTTGGCAACTGACTAATTCCACTAATAATCGGCGCCCCAGACATGGCT
TTCCCTCGTATGAACAACATAAGCTTTTGACTGCTCCCCCCATCTTTCCTCCTCCT
CCTCGCCTCCTCAACTGTTGAAGCCGGGGTAGGGACAGGCTGGACTGTGTACCCG
CCTCTTTCCGGGAATTTGGCCCACGACGGCCCATCCGTGGACCTGGCCATCTTTTC
CCTCCACTTAGCGGGGGTGTCCTCAATTTTAGGTGCAATCAACTTTATCACTACTA
TCATTAACATGAAACCCCCAGCCACCTCTTTATACAATACCCCCCTGTTTATCTGA
TCCCTCCTCGTCACTGCTGTGCTTCTACTTCTTTCACTACCAGTTCTTGCTGCTGGT
ATCACTATACTTCTAACGGACCGGAATTT"/>
<sequence id="seq_Abar_Mito_Apisto_300549"
taxon="Abar_Mito_Apisto_300549" totalcount="4"
value="CCCTAATTGATCTTCCCACCCCCTCCAACATTTCCTCTTGATGAAATTTTGG
GTCTCTACTAGGCCTCTGCTTAATTTCCCAAATTCTTACAGGCCTATTTCTTTCCAT
ACACTATTCTGCTGACATCAACACAGCTTTTTCATCCGTTGCCCACATCTCTCGAG
ACGTAAACTACGGATGGCTAATCCGCAATTTACACGCTAACGGAGCATCCTTTTT
CTTCATTTGCATCTATCTTCACATCGGACGGGGCCTTTACTTCGGATCTTACCTCT
ACAAAGAGACATGAAACATTGGAGTAGTACTTCTACTCCTAGTTATAATAACCGC
TTTTGTGGGCTACGTCCTCCCCTGAGGCCAAATATCGTTTTGAGGAGCTACCGTCA
TCACCAACCTACTGTCAGCAATCCCCTACATTGGAAACTCCCTAGTCCAATGAAT
TTGAGGTGGCTTCTCAGTTGACAACGCTACCCTAACCCGATTCTTTGCCATCCACT
TCCTACTTCCATTCGTCATCGCAGCCGTAACCCTAATACACCTAATTTTTCTCCAC
GAAACAGGCTCCACCAACCCAATCGGACTGACCTCCAACACGGATAAAATTTCTT
TCCACCCATTTTTCTCCCTCAAAGACCTCCTCGGATTCCTAATTTTACTTATAACTC
TGATATTTGGTGCTTGAGCTGGAATAGCAGGTACCGCATTAAGCATGCTAATTCG
AGCAGAACTTACCCAACCCGGCTCCTTTTTTGGGGACGATCAAGTATATAATGTA
ATCGTAACTGCACACGCTTTCGTAATAATCTTCTTTATAGTAATACCAATTATAAT
CGGTGGATTTGGCAACTGACTAATTCCACTAATAATCGGCGCCCCAGACATGGCT
TTCCCTCGTATGAACAACATAAGCTTTTGACTGCTCCCCCCATCTTTCCTCCTCCT
CCTCGCCTCCTCAACTGTTGAAGCCGGGGTAGGGACAGGCTGGACTGTGTACCCG
CCTCTTTCCGGGAATTTGGCCCACGACGGCCCATCCGTGGACCTGGCCATCTTTTC
CCTCCACTTAGCGGGGGTGTCCTCAATTTTAGGTGCAATCAACTTTATCACTACTA
TCATTAACATGAAACCCCCAGCCACCTCTTTATACAATACCCCCCTGTTTATCTGA
TCCCTCCTCGTCACTGCTGTGCTTCTACTTCTTTCACTACCAGTTCTTGCTGCTGGT
ATCACTATACTTCTAACGGACCGGAATTT"/>

<sequence id="seq_Abar_Mito_Apisto_300550"
taxon="Abar_Mito_Apisto_300550" totalcount="4"
value="CCCTAATTGATCTTCCCACCCCCTCCAACATTTCCTCTTGATGAAATTTTGG
GTCTCTACTAGGCCTCTGCTTAATTTCCCAAATTCTTACAGGCCTATTTCTTTCCAT
ACACTATTCTGCTGACATCAACACAGCTTTTTCATCCGTTGCCCACATCTCTCGAG
ACGTAAACTACGGATGGCTAATCCGCAATTTACACGCTAACGGAGCATCCTTTTT
CTTCATTTGCATCTATCTTCACATCGGACGGGGCCTTTACTTCGGATCTTACCTCT
ACAAAGAGACATGAAACATTGGAGTAGTACTTCTACTCCTAGTTATAATAACCGC
TTTTGTGGGCTACGTCCTCCCCTGAGGCCAAATATCGTTTTGAGGAGCTACCGTCA
TCACCAACCTACTGTCAGCAATCCCCTACATTGGAAACTCCCTAGTCCAATGAAT
TTGAGGTGGCTTCTCAGTTGACAACGCTACCCTAACCCGATTCTTTGCCATCCACT
TCCTACTTCCATTCGTCATCGCAGCCGTAACCCTAATACACCTAATTTTTCTCCAC
GAAACAGGCTCCACCAACCCAATCGGACTGACCTCCAACACGGATAAAATTTCTT
TCCACCCATTTTTCTCCCTCAAAGACCTCCTCGGATTCCTAATTTTACTTATAACTC
TGATATTTGGTGCTTGAGCTGGAATAGCAGGTACCGCATTAAGCATGCTAATTCG
AGCAGAACTTACCCAACCCGGCTCCTTTTTTGGGGACGATCAAGTATATAATGTA
ATCGTAACTGCACACGCTTTCGTAATAATCTTCTTTATAGTAATACCAATTATAAT
CGGTGGATTTGGCAACTGACTAATTCCACTAATAATCGGCGCCCCAGACATGGCT
TTCCCTCGTATGAACAACATAAGCTTTTGACTGCTCCCCCCATCTTTCCTCCTCCT
CCTCGCCTCCTCAACTGTTGAAGCCGGGGTAGGGACAGGCTGGACTGTGTACCCG
CCTCTTTCCGGGAATTTGGCCCACGACGGCCCATCCGTGGACCTGGCCATCTTTTC
CCTCCACTTAGCGGGGGTGTCCTCAATTTTAGGTGCAATCAACTTTATCACTACTA
TCATTAACATGAAACCCCCAGCCACCTCTTTATACAATACCCCCCTGTTTATCTGA
TCCCTCCTCGTCACTGCTGTGCTTCTACTTCTTTCACTACCAGTTCTTGCTGCTGGT
ATCACTATACTTCTAACGGACCGGAATTT"/>
<sequence id="seq_Abar_Mito_Apisto_300554"
taxon="Abar_Mito_Apisto_300554" totalcount="4"
value="CCCTAATTGATCTTCCCACCCCCTCCAACATTTCCTCTTGATGAAATTTTGG
GTCTCTACTAGGCCTCTGCTTAATTTCCCAAATTCTTACAGGCCTATTTCTTTCCAT
ACACTATTCTGCTGACATCAACACAGCTTTTTCATCCGTTGCCCACATCTCTCGAG
ACGTAAACTACGGATGGCTAATCCGCAATTTACACGCTAACGGAGCATCCTTTTT
CTTCATTTGCATCTATCTTCACATCGGACGGGGCCTTTACTTCGGATCTTACCTCT
ACAAAGAGACATGAAACATTGGAGTAGTACTTCTACTCCTAGTTATAATAACCGC
TTTTGTGGGCTACGTCCTCCCCTGAGGCCAAATATCGTTTTGAGGAGCTACCGTCA
TCACCAACCTACTGTCAGCAATCCCCTACATTGGAAACTCCCTAGTCCAATGAAT
TTGAGGTGGCTTCTCAGTTGACAACGCTACCCTAACCCGATTCTTTGCCATCCACT
TCCTACTTCCATTCGTCATCGCAGCCGTAACCCTAATACACCTAATTTTTCTCCAC
GAAACAGGCTCCACCAACCCAATCGGACTGACCTCCAACACGGATAAAATTTCTT
TCCACCCATTTTTCTCCCTCAAAGACCTCCTCGGATTCCTAATTTTACTTATAACTC
TGATATTTGGTGCTTGAGCTGGAATAGCAGGTACCGCATTAAGCATGCTAATTCG
AGCAGAACTTACCCAACCCGGCTCCTTTTTTGGGGACGATCAAGTATATAATGTA
ATCGTAACTGCACACGCTTTCGTAATAATCTTCTTTATAGTAATACCAATTATAAT
CGGTGGATTTGGCAACTGACTAATTCCACTAATAATCGGCGCCCCAGACATGGCT
TTCCCTCGTATGAACAACATAAGCTTTTGACTGCTCCCCCCATCTTTCCTCCTCCT
CCTCGCCTCCTCAACTGTTGAAGCCGGGGTAGGGACAGGCTGGACTGTGTACCCG
CCTCTTTCCGGGAATTTGGCCCACGACGGCCCATCCGTGGACCTGGCCATCTTTTC
CCTCCACTTAGCGGGGGTGTCCTCAATTTTAGGTGCAATCAACTTTATCACTACTA
TCATTAACATGAAACCCCCAGCCACCTCTTTATACAATACCCCCCTGTTTATCTGA
TCCCTCCTCGTCACTGCTGTGCTTCTACTTCTTTCACTACCAGTTCTTGCTGCTGGT
ATCACTATACTTCTAACGGACCGGAATTT"/>

<sequence id="seq_Abar_Mito_Apisto_300557"
taxon="Abar_Mito_Apisto_300557" totalcount="4"
value="CCCTAATTGATCTTCCCACCCCCTCCAACATTTCCTCTTGATGAAATTTTGG
GTCTCTACTAGGCCTCTGCTTAATTTCCCAAATTCTTACAGGCCTATTTCTTTCCAT
ACACTATTCTGCTGACATCAACACAGCTTTTTCATCCGTTGCCCACATCTCTCGAG
ACGTAAACTACGGATGGCTAATCCGCAATTTACACGCTAACGGAGCATCCTTTTT
CTTCATTTGCATCTATCTTCACATCGGACGGGGCCTTTACTTCGGATCTTACCTCT
ACAAAGAGACATGAAACATTGGAGTAGTACTTCTACTCCTAGTTATAATAACCGC
TTTTGTGGGCTACGTCCTCCCCTGAGGCCAAATATCGTTTTGAGGAGCTACCGTCA
TCACCAACCTACTGTCAGCAATCCCCTACATTGGAAACTCCCTAGTCCAATGAAT
TTGAGGTGGCTTCTCAGTTGACAACGCTACCCTAACCCGATTCTTTGCCATCCACT
TCCTACTTCCATTCGTCATCGCAGCCGTAACCCTAATACACCTAATTTTTCTCCAC
GAAACAGGCTCCACCAACCCAATCGGACTGACCTCCAACACGGATAAAATTTCTT
TCCACCCATTTTTCTCCCTCAAAGACCTCCTCGGATTCCTAATTTTACTTATAACTC
TGATATTTGGTGCTTGAGCTGGAATAGCAGGTACCGCATTAAGCATGCTAATTCG
AGCAGAACTTACCCAACCCGGCTCCTTTTTTGGGGACGATCAAGTATATAATGTA
ATCGTAACTGCACACGCTTTCGTAATAATCTTCTTTATAGTAATACCAATTATAAT
CGGTGGATTTGGCAACTGACTAATTCCACTAATAATCGGCGCCCCAGACATGGCT
TTCCCTCGTATGAACAACATAAGCTTTTGACTGCTCCCCCCATCTTTCCTCCTCCT
CCTCGCCTCCTCAACTGTTGAAGCCGGGGTAGGGACAGGCTGGACTGTGTACCCG
CCTCTTTCCGGGAATTTGGCCCACGACGGCCCATCCGTGGACCTGGCCATCTTTTC
CCTCCACTTAGCGGGGGTGTCCTCAATTTTAGGTGCAATCAACTTTATCACTACTA
TCATTAACATGAAACCCCCAGCCACCTCTTTATACAATACCCCCCTGTTTATCTGA
TCCCTCCTCGTCACTGCTGTGCTTCTACTTCTTTCACTACCAGTTCTTGCTGCTGGT
ATCACTATACTTCTAACGGACCGGAATTT"/>
<sequence id="seq_Abit1_Mito_Apisto_300438"
taxon="Abit1_Mito_Apisto_300438" totalcount="4"
value="CCCTAATTGACCTTCCCGCCCCCTCCAACATCTCCGCCTGATGAAATTTCG
GCTCCCTTCTAGGCCTCTGCTTGGTTTCACAAATCCTGACAGGCCTATTCCTCTCC
ATACATTACACTGCTGACATCAACACAGCCTTTTCATCCGTCGCCCACATCTCTCG
AGATGTAAACTACGGGTGATTAATTCGAAGTATTCATGCTAATGGGGCATCTTTT
TTCTTCATTTGCATTTATCTACACATCGGACGAGGCCTCTACTTCGGCTCCTATCT
CTACAAAGAGACATGAAATATTGGAGTAGTACTACTACTACTACTAATAATAACC
GCTTTTACAGGCTATGTCCTCCCATGAGGACAAATATCATTTTGAGGGGCCACCG
TCATCACCAACCTACTTTCAGCAGCCCCCTACATCGGGGACTCCCTAGTTCAATG
AATTTGAGGCGGCTTCTCAGTTGACAGCGCTACCTTAACCCGCTTCTTCACCTTCC
ACTTCATTCTTCCCTTCGCTATCACAGCCATAGCCCTGGTTCACCTAATCTTTCTTC
ACGAAACAGGCTCTACAAATCCAATTGGATTAAACCCAAACGCAGATAAAGTTT
CCTTCCACCCATTCTTCTCCCTCAAAGACCTTCTCGGGTTTCTAATTCTGCTCACA
GCCCTGATATTTGGTGCTTTTGCCGGACTAGTGGGCGCCGCGTTAAGCACACTGA
TTCGAGCAGAACTTACTCAGTCCGGCTCCCTTTTTGAAGACGATCAGCTTTACAA
CGTAATTGTAACTGCACACGCCTTCGTAATAATTTTCTTTATGGTTATACCAATCA
TGATTGGTGGGTTCGGCAATTGACTAATCCCATTAATAATCGGCGCCCCAGACAT
GGCCTTCCCTCGTTTGAACAATCTAAGCTTTTGACTTCTCCCCCCATCCTTTTTCCT
CCTCCTCGCCTCCTCCCTCGTTGAGGGAGGCGTGGGGACAGGCTGAACTGTGTAC
CCCCCCCTCTCCGGAAATTTGGCCCATGCCGGACCATCCGTAGATCTGGCCATCTT
TTCCCTTCACTTAGCAGGGGTGTCCTCGATTTTAGGAGCAATCAACTTTATCACCA
CTGTTATTAATATAAAACCCCCTGCTATCCCTATGCGCCACACACCTCTATTTATC
TGATCCCTTCTCATCACTGCTGTACTCTTACTCCTATCGCTTCCAGTACTTGCTGCC
GGGATCACCATACTCATAACGGACCgAAACCT"/>

<sequence id="seq_Abit1_Mito_Apisto_300440"
taxon="Abit1_Mito_Apisto_300440" totalcount="4"
value="CCCTAATTGACCTTCCCGCCCCCTCCAACATCTCCGCCTGATGAAATTTCG
GCTCCCTTCTAGGCCTCTGCTTGGTTTCACAAATCCTGACAGGCCTATTCCTCTCC
ATACATTACACTGCTGACATCAACACAGCCTTTTCATCCGTCGCCCACATCTCTCG
AGATGTAAACTACGGGTGATTAATTCGAAGTATTCATGCTAATGGGGCATCTTTT
TTCTTCATTTGCATTTATCTACACATCGGACGAGGCCTCTACTTCGGCTCCTATCT
CTACAAAGAGACATGAAATATTGGAGTAGTACTACTACTACTACTAATAATAACC
GCTTTTACAGGCTATGTCCTCCCATGAGGACAAATATCATTTTGAGGGGCCACCG
TCATCACCAACCTACTTTCAGCAGCCCCCTACATCGGGGACTCCCTAGTTCAATG
AATTTGAGGCGGCTTCTCAGTTGACAGCGCTACCTTAACCCGCTTCTTCACCTTCC
ACTTCATTCTTCCCTTCGCTATCACAGCCATAGCCCTGGTTCACCTAATCTTTCTTC
ACGAAACAGGCTCTACAAATCCAATTGGATTAAACCCAAACGCAGATAAAGTTT
CCTTCCACCCATTCTTCTCCCTCAAAGACCTTCTCGGGTTTCTAATTCTGCTCACA
GCCCTGATATTTGGTGCTTTTGCCGGACTAGTGGGCGCCGCGTTAAGCACACTGA
TTCGAGCAGAACTTACTCAGTCCGGCTCCCTTTTTGAAGACGATCAGCTTTACAA
CGTAATTGTAACTGCACACGCCTTCGTAATAATTTTCTTTATGGTTATACCAATCA
TGATTGGTGGGTTCGGCAATTGACTAATCCCATTAATAATCGGCGCCCCAGACAT
GGCCTTCCCTCGTTTGAACAATCTAAGCTTTTGACTTCTCCCCCCATCCTTTTTCCT
CCTCCTCGCCTCCTCCCTCGTTGAGGGAGGCGTGGGGACAGGCTGAACTGTGTAC
CCCCCCCTCTCCGGAAATTTGGCCCATGCCGGACCATCCGTAGATCTGGCCATCTT
TTCCCTTCACTTAGCAGGGGTGTCCTCGATTTTAGGAGCAATCAACTTTATCACCA
CTGTTATTAATATAAAACCCCCTGCTATCCCTATGCGCCACACACCTCTATTTATC
TGATCCCTTCTCATCACTGCTGTACTCTTACTCCTATCGCTTCCAGTACTTGCTGCC
GGGATCACCATACTCTTAACGGACCGAAACCT"/>
<sequence id="seq_Abit3_Mito_Apisto_303098"
taxon="Abit3_Mito_Apisto_303098" totalcount="4"
value="CCCTAATTGACCTTCCCGCCCCCTCCAACATCTCCGCCTGATGAAATTTCG
GCTCCCTTCTAGGCCTCTGCCTGGTCCTACAAATCCTAACAGGCCTATTCCTCTCT
ATGCATTACACTGCTGACATCAACACAGCCTTTTCATCCGTCGCCCATATCTGTCG
AGACGTAAACTACGGATGATTAATCCGAAGTATGCATGCTAATGGAGCATCTTTC
TTCTTTATTTGCATTTATCTGCACATCGGACGAGGCCTTTACTTCGGCTCCTATCTC
TACAAAGAGACATGAAATATTGGAGTAGTACTACTACTACTACTAATAATAACCG
CTTTTACAGGCTATGTCCTTCCATGAGGACAAATATCATTTTGAGGGGCCACCGT
CATCACTAACCTACTATCAGCAGCCCCCTACATCGGGGACTCCCTAGTTCAATGA
ATTTGAGGCGGCTTCTCAGTTGACAGTGCTACCCTAACCCGCTTCTTCACCTTTCA
CTTTCTTCTTCCCTTCGCTATCACAGCCGCAACCCTGATTCACCTAATCTTTCTCCA
CGAAACAGGCTCCACAAACCCAATTGGACTAAACCCAAACGCAGATAAAGTTTC
CTTCCACCCGTTCTTCTCCCTCAAAGACCTTCTCGGGTTCCTAATTCTGCTCACAG
CCCTGATATTTGGTGCTTTTGCCGGACTAGTGGGCGCCGCGTTAAGCACACTGAT
TCGAGCAGAACTTACTCAGTCCGGCTCCCTTTTTGAAGACGATCAGCTTTACAAC
GTAATCGTAACTGCACACGCCTTCGTAATAATTTTCTTTATGGTTATACCAATCAT
GATTGGTGGGTTCGGCAATTGACTAATCCCGTTAATAATCGGCGCCCCAGACATG
GCCTTCCCTCGTTTGAACAATCTAAGCTTTTGACTTCTCCCCCCATCCTTTCTCCTC
CTCCTCGCCTCCTCCTTCGTTGAGGGCGGCGTGGGGACAGGCTGAACTGTGTACC
CCCCCCTCTCCGGAAATTTGGCCCATGCCGGACCATCCGTAGATCTGGCCATCTTT
TCCCTTCACTTAGCAGGGGTGTCCTCGATTTTAGGAGCAATCAACTTTATCACCAC
TGTCATTAACATAAAACCCCCTGCTATCCCTATGCGTCACACACCTCTATTTATCT
GATCCCTTCTCATCACTGCTGTACTCCTACTCCTATCGCTTCCAGTACTTGCTGCC
GGGATCACCATACTTTTAACGGACCGAAACCT"/>

<sequence id="seq_Abit4_Mito_Apisto_302612"
taxon="Abit4_Mito_Apisto_302612" totalcount="4"
value="CCCTAATTGACCTTCCCGCCCCCTCCAACATCTCCGCCTGATGAAATTTTG
GCTCCCTTCTAGGCCTCTGCCTGGTCTTACAAATCCTAACAGGCCTATTCCTCTCT
ATGCATTACACTGCTGACATCAACACAGCCTTTTCATCCGTCGCCCATATCTGTCG
AGACGTAAACTACGGGTGATTAATTCGAAGTATGCATGCTAATGGGGCATCTTTT
TTCTTTATTTGTATTTATCTGCACATCGGACGAGGCCTTTACTTCGGCTCCTATCTC
TACAAAGAGACATGAAATATTGGAGTAGTACTACTATTACTACTAATAATAACCG
CTTTTACAGGCTATGTCCTTCCATGAGGACAAATATCATTTTGAGGGGCCACCGT
CATCACTAACCTACTATCAGCAGCCCCCTACATTGGGGACTCCCTAGTTCAATGA
ATTTGGGGCGGCTTCTCAGTTGACAGTGCTACCCTAACCCGCTTCTTCACCTTTCA
CTTTCTTCTTCCCTTCGCTATCACAGCCACAACCCTGATTCACCTAATCTTTCTCCA
CGAAACGGGCTCCACAAACCCAATTGGACTAAACCCAAATGCAGATAAAGTTTC
CTTCCACCCATTCTTCTCCCTCAAAGACCTTCTCGGGTTCCTAATTCTGCTCACAG
CCCTGATATTTGGTGCTTTTGCCGGATTAGTGGGCGCCGCGTTAAGCACACTGATT
CGAGCAGAACTTACTCAGTCCGGCTCCTTTTTTGAAGACGACCAGTTTTACAACG
TAATTGTGACTGCACACGCCTTCGTAATAATTTTCTTTATGGTTATACCAATTATG
ATCGGTGGGTTCGGCAATTGACTAATCCCACTAATGATCGGCGCCCCGGACATGG
CCTTCCCTCGTTTGAATAATCTAAGCTTTTGACTTCTCCCCCCATCCTTTCTCCTCC
TCCTCGCCTCCTCCTTCGTTGAGGGCGGCGTAGGGACAGGCTGAACTGTGTACCC
CCCCCTCTCCGGAAATTTGGCCCATGCCGGGCCATCCGTAGATCTGGCCATTTTTT
CCCTTCACTTAGCAGGGGTGTCCTCGATTTTAGGAGCAATCAACTTTATCACCACT
ATTATTAACATAAAACCCCCCGCTATCTCTATGCGCCGCACACCCCTATTTATCTG
ATCCCTCCTCATCACTGCTGTACTCTTGCTTCTGTCGCTTCCAGTACTTGCTGCTGG
AATCACCATGCTCTTAACGGACCGAAACCT"/>
<sequence id="seq_Abit4_Mito_Apisto_302614"
taxon="Abit4_Mito_Apisto_302614" totalcount="4"
value="CCCTAATTGACCTTCCCGCCCCCTCCAACATCTCCGCCTGATGAAATTTTG
GCTCCCTTCTAGGCCTCTGCCTGGTCTTACAAATCCTAACAGGCCTATTCCTCTCT
ATGCATTACACTGCTGACATCAACACAGCCTTTTCATCCGTCGCCCATATCTGTCG
AGACGTAAACTACGGGTGATTAATTCGAAGTATGCATGCTAATGGGGCATCTTTT
TTCTTTATTTGTATTTATCTGCACATCGGACGAGGCCTTTACTTCGGCTCCTATCTC
TACAAAGAGACATGAAATATTGGAGTAGTACTACTATTACTACTAATAATAACCG
CTTTTACAGGCTATGTCCTTCCATGAGGACAAATATCATTTTGAGGGGCCACCGT
CATCACTAACCTACTATCAGCAGCCCCCTACATTGGGGACTCCCTAGTTCAATGA
ATTTGGGGCGGCTTCTCAGTTGACAGTGCTACCCTAACCCGCTTCTTCACCTTTCA
CTTTCTTCTTCCCTTCGCTATCACAGCCACAACCCTGATTCACCTAATCTTTCTTCA
CGAAACGGGCTCCACAAACCCAATTGGACTAAACCCAAATGCAGATAAAGTTTC
CTTCCACCCATTCTTCTCCCTCAAAGACCTTCTCGGGTTCCTAATTCTGCTCACAG
CCCTGATATTTGGTGCTTTTGCCGGATTAGTGGGCGCCGCGTTAAGCACACTGATT
CGAGCAGAACTTACTCAGTCCGGCTCCTTTTTTGAAGACGACCAGTTTTACAACG
TAATTGTGACTGCACACGCCTTCGTAATAATTTTCTTTATGGTTATACCAATTATG
ATCGGTGGGTTCGGCAATTGACTAATCCCACTAATGATCGGCGCCCCGGACATGG
CCTTCCCTCGTTTGAATAATCTAAGCTTTTGACTTCTCCCCCCATCCTTTCTCCTCC
TCCTCGCCTCCTCCTTCGTTGAGGGCGGCGTAGGGACAGGCTGAACTGTGTACCC
CCCCCTCTCCGGAAATTTGGCCCATGCCGGGCCATCCGTAGATCTGGCCATTTTTT
CCCTTCACTTAGCAGGGGTGTCCTCGATTTTAGGAGCAATCAACTTTATCACCACT
ATTATTAACATAAAACCCCCCGCTATCTCTATGCGCCGCACACCCCTATTTATCTG
ATCCCTCCTCATCACTGCTGTACTCTTGCTTCTGTCGCTTCCAGTACTTGCTGCTGG
AATCACCATGCTCTTAACGGACCGAAACCT"/>

<sequence id="seq_Abit5_Mito_Apisto_302926"
taxon="Abit5_Mito_Apisto_302926" totalcount="4"
value="CCCTAATTGACCTTCCCGCCCCCTCCAACATCTCCGCCTGATGAAATTTCG
GCTCCCTTCTAGGCCTCTGCCTGGTCCTACAAATCCTAACAGGCCTATTCCTCTCT
ATGCATTACACTGCTGACATCAACACAGCCTTTTCATCCGTCGCCCATATCTGTCG
AGACGTAAACTACGGATGATTAATCCGAAGTATGCATGCTAATGGAGCATCTTTC
TTCTTTATTTGCATTTATCTGCACATCGGACGAGGCCTTTACTTCGGCTCCTATCTC
TACAAAGAGACATGAAATATTGGAGTAGTACTACTACTACTACTAATAATAACCG
CTTTTACAGGCTATGTCCTTCCATGAGGACAAATATCATTTTGAGGGGCCACCGT
CATCACTAACCTACTATCAGCAGCCCCCTACATCGGGGACTCCCTAGTTCAATGA
ATTTGAGGCGGCTTCTCAGTTGACAGTGCTACCCTAACCCGCTTCTTCACCTTTCA
CTTTCTTCTTCCCTTCGCTATCACAGCCGCAACCCTGATTCACCTAATCTTTCTCCA
CGAAACAGGCTCCACAAACCCAATTGGACTAAACCCAAACGCAGATAAAGTTTC
CTTCCACCCGTTCTTCTCCCTCAAAGACCTTCTCGGGTTCCTAATTCTGCTCACAG
CCCTGATATTTGGTGCTATTGCCGGATTAGTAGGCGCCGCGTTAAGCACACTGAT
TCGAGCAGAACTTACTCAGTCCGGCTCCCTTTTTGAAGACGACCAGCTTTACAAC
GTAATTGTAACTGCACACGCCTTCGTAATAATTTTCTTTATGGTTATACCAATCAT
GATCGGTGGGTTCGGCAATTGACTAATCCCACTAATGATCGGCGCCCCGGACATG
GCCTTCCCTCGTTTGAATAATCTAAGCTTTTGACTTCTCCCCCCATCCTTTCTCCTC
CTCCTCGCCTCCTCCTCCGTTGAGGGCGGCGTGGGGACAGGCTGAACTGTGTACC
CCCCCCTCTCCGGAAATTTGGCCCATGCCGGGCCATCCGTAGATCTGGCCATTTTC
TCCCTTCACTTAGCAGGGGTGTCCTCGATTTTAGGAGCAATCAACTTTATTACCAC
TATTATTAACATAAAACCCCCCGCTATCTCTATGCGCCGCACACCCCTATTTATCT
GATCCCTCCTCATCACTGCTGTACTCTTACTTCTGTCGCTTCCAGTACTTGCTGCTG
GGATCACCATGCTCTTAACGGACCGAAACCT"/>
<sequence id="seq_Abit5_Mito_Apisto_302928"
taxon="Abit5_Mito_Apisto_302928" totalcount="4"
value="CCCTAATTGACCTTCCCGCCCCCTCCAACATCTCCGCCTGATGAAATTTCG
GCTCCCTTCTAGGCCTCTGCCTGGTCCTACAAATCCTAACAGGCCTATTCCTCTCT
ATGCATTACACTGCTGACATCAACACAGCCTTTTCATCCGTCGCCCATATCTGTCG
AGACGTAAACTACGGATGATTAATCCGAAGTATGCATGCTAATGGAGCATCTTTC
TTCTTTATTTGCATTTATCTGCACATCGGACGAGGCCTTTACTTCGGCTCCTATCTC
TACAAAGAGACATGAAATATTGGAGTAGTACTACTACTACTACTAATAATAACCG
CTTTTACAGGCTATGTCCTTCCATGAGGACAAATATCATTTTGAGGGGCCACCGT
CATCACTAACCTACTATCAGCAGCCCCCTACATCGGGGACTCCCTAGTTCAATGA
ATTTGAGGCGGCTTCTCAGTTGACAGTGCTACCCTAACCCGCTTCTTCACCTTTCA
CTTTCTTCTTCCCTTCGCTATCACAGCCGCAACCCTGATTCACCTAATCTTTCTCCA
CGAAACAGGCTCCACAAACCCAATTGGACTAAACCCAAACGCAGATAAAGTTTC
CTTCCACCCGTTCTTCTCCCTCAAAGACCTTCTCGGGTTCCTAATTCTGCTCACAG
CCCTGATATTTGGTGCTATTGCCGGATTAGTAGGCGCCGCGTTAAGCACACTGAT
TCGAGCAGAACTTACTCAGTCCGGCTCCCTTTTTGAAGACGACCAGCTTTACAAC
GTAATTGTAACTGCACACGCCTTCGTAATAATTTTCTTTATGGTTATACCAATCAT
GATCGGTGGGTTCGGCAATTGACTAATCCCACTAATGATCGGCGCCCCGGACATG
GCCTTCCCTCGTTTGAATAATcTAAGCTTTTGACTTCTCCCCCCATCCTTTCTCCTC
CTCCTCGCCTCCTCCTCCGTTGAGGGCGGCGTGGGGACAGGCTGAACTGTGTACC
CCCCCCTCTCCGGAAATTTGGCCCATGCCGGGCCATCCGTAGATCTGGCCATTTTC
TCCCTTCACTTAGCAGGGGTGTCCTCGATTTTAGGAGCAATCAACTTTATTACCAC
TATTATTAACATAAAACCCCCCGCTATCTCTATGCGCCGCACACCCCTATTTATCT
GATCCCTCCTCATCACTGCTGTACTCTTACTTCTGTCGCTTCCAGTACTTGCTGCTG
GGATCACCATGCTCTTAACGGACCGAAACCT"/>

<sequence id="seq_Abit5_Mito_Apisto_302951"
taxon="Abit5_Mito_Apisto_302951" totalcount="4"
value="CCCTAATTGACCTTCCCGCCCCCTCCAACATCTCCGCCTGATGAAATTTCG
GCTCCCTTCTAGGCCTCTGCCTGGTCCTACAAATCCTAACAGGCCTATTCCTCTCT
ATGCATTACACTGCTGATATCAACACAGCCTTTTCATCCGTCGCCCATATCTGTCG
AGACGTAAACTACGGATGATTAATCCGAAGTATGCATGCTAATGGAGCATCTTTC
TTCTTTATTTGCATTTATCTGCACATCGGACGAGGCCTTTACTTCGGCTCCTATCTC
TACAAAGAGACATGAAATATTGGAGTAGTACTACTACTACTACTAATAATAACCG
CTTTTACAGGCTATGTCCTTCCATGAGGACAAATATCATTTTGAGGGGCCACCGT
CATCACTAACCTACTATCAGCAGCCCCCTACATCGGGGACTCCCTAGTTCAATGA
ATTTGAGGCGGCTTCTCAGTTGACAGTGCTACCCTAACCCGCTTCTTCACCTTTCA
CTTTCTTCTTCCCTTCGCTATCACAGCCGCAACCCTGATTCACCTAATCTTTCTCCA
CGAAACAGGCTCCACAAACCCAATTGGACTAAACCCAAACGCAGATAAAGTTTC
CTTCCACCCGTTCTTCTCCCTCAAAGACCTTCTCGGGTTCCTAATTCTGCTCACAG
CCCTGATATTTGGTGCTATtGCCGGAtTAGCAGGCGCCGCGTTAAGCACACTGATT
CGAGCAGAACTTACTCAGTCCGGCTCCCTTTTTGAAGACGACCAgCTTTACAACGT
AATTGTAACTGCACACGCCTTCGTAATAATTTTcTTTATGGTTATACCAATCATGA
TCGGTGGGTTCGGCAATTGAcTAATcCCACTAATgATCGGCGCCCCGGACATGGCC
TTCCCTCGttTGAATAATcTAAGCTTTTGACTTCTCCCCCCATCCTTTCTCCTCCTCC
TCGCCTCCTCCTCCGTTGAgGGCGGcGTGGGGACAGGCTGAACTGTGTACCCCCCC
CTCTCCGGAAATTTGGCCCATGCCGGGCCATCCGTAGATCTGGCCATTTTCTCCCT
TCACTTAGCAGGGGTGTCCTCGATTTAGGAGCAATCAACTTTATTACCACTATTA
TTAACATAAAACCCCCCGCTATCTCTATGCGCCGCaCACCCCTATTTATCTGATCC
CTCCTCaTCACTGCTGTACTCTTACTTCTGTCGCTTCCAGTACTTGCTGCTGGGATC
ACCATGCTCTTAACGGACCgAAACCT"/>
<sequence id="seq_Abit5_Mito_Apisto_302952"
taxon="Abit5_Mito_Apisto_302952" totalcount="4"
value="CCCTAATTGACCTTCCCGCCCCCTCCAACATCTCCGCCTGATGAAATTTCG
GCTCCCTTCTAGGCCTCTGCCTGGTCCTACAAATCCTAACAGGCCTATTCCTCTCT
ATGCATTACACTGCTGACATCAACACAGCCTTTTCATCCGTCGCCCATATCTGTCG
AGACGTAAACTACGGATGATTAATCCGAAGTATGCATGCTAATGGAGCATCTTTC
TTCTTTATTTGCATTTATCTGCACATCGGACGAGGCCTTTACTTCGGCTCCTATCTC
TACAAAGAGACATGAAATATTGGAGTAGTACTACTACTACTACTAATAATAACCG
CTTTTACAGGCTATGTCCTTCCATGAGGACAAATATCATTTTGAGGGGCCACCGT
CATCACTAACCTACTATCAGCAGCCCCCTACATCGGGGACTCCCTAGTTCAATGA
ATTTGAGGCGGCTTCTCAGTTGACAGTGCTACCCTAACCCGCTTCTTCACCTTTCA
CTTTCTTCTTCCCTTCGCTATCACAGCCGCAACCCTGATTCACCTAATCTTTCTCCA
CGAAACAGGCTCCACAAACCCAATTGGACTAAACCCAAACGCAGATAAAGTTTC
CTTCCACCCGTTCTTCTCCCTCAAAGACCTTCTCGGGTTCCTAATTCTGCTCACAG
CTTTGATATTTGGTGCTATTGCCGGATTAGTAGGCGCCGCGTTAAGCACACTGATT
CGAGCAGAACTTACTCAGTCCGGCTCCCTTTTTGAAGACGACCAGCTTTACAACG
TAATTGTAACTGCACACGCCTTCGTAATAATTTTCTTTATGGTTATACCAATCATG
ATCGGTGGGTTCGGCAATTGACTAATCCCACTAATGATCGGCGCCCCGGACATGG
CCTTCCCTCGTTTGAATAATCTAAGCTTTTGACTTCTCCCCCCATCCTTTCTCCTCC
TCCTCGCCTCCTCCTCCGTTGAGGGCGGCGTGGGGACAGGCTGAACTGTGTACCC
CCCCCTCTCCGGAAATTTGGCCCATGCCGGGCCATCCGTAGATCTGGCCATTTTCT
CCCTTCACTTAGCAGGGGTGTCCTCGATTTAGGAGCAATCAACTTTATTACCACT
ATTATTAACATAAAACCCCCCGCTATCTCTATGCGCCGCACACCCCTATTTATCTG
ATCCCTCCTCATCACTGCTGTACTCTTACTTCTGTCGCTTCCAGTACTTGCTGCTGG
GATCACCATGCTCTTAACGGACCgAAACCT"/>

<sequence id="seq_Abit_Mito_Apisto_302613"
taxon="Abit_Mito_Apisto_302613" totalcount="4"
value="CCCTAATTGACCTTCCCGCCCCCTCCAACATCTCCGCCTGATGAAATTTCG
GCTCCCTCTTAGGCCTCTGCTTGATTTCACAAATCCTGACAGGCCTATTCCTCTCC
ATGCATTACACTGCTGACATCAACACAGCCTTTTCATCCGTCGCCCACATCTCTCG
AGATGTAAACTACGGGTGATTAATTCGAAATATTCATGCTAATGGGGCATCTTTT
TTCTTCATTTGCATTTATCTGCACATCGGACGAGGCCTTTACTTCGGCTCCTATCT
CTACAAAGAGACATGAAATATCGGAGTAGTACTACTGTTACTACTAATAATAACC
GCTTTTACAGGCTATGTCCTCCCATGGGGACAAATATCATTTTGAGGGGCCACCG
TCATCACCAACCTACTATCAGCAGCCCCCTACATCGGGGACTCCCTAGTTCAATG
AATTTGGGGCGGCTTCTCAGTTGACAGCGCTACCTTAACCCGCTTCTTCACCTTCC
ACTTCATTCTTCCCTTCGCTATTACAGCCATAACCCTGGTTCACCTAATCTTTCTTC
ACGAAACAGGCTCTACAAATCCAATTGGGTTAAACCCAAACGCAGATAAAGTTT
CCTTCCACCCATTCTTCTCCCTTAAAGACCTTCTCGGGTTTCTAATTCTGCTCACA
GCCCTGATATTTGGTGCTTTTGCCGGACTAGTGGGCGCCGCGTTAAGCACACTGA
TTCGAGCAGAACTTACTCAGTCCGGCTCCCTTTTTGAAGACGATCAGCTTTACAA
CGTAATCGTAACTGCACACGCCTTCGTAATAATTTTCTTTATGGTTATACCAATCA
TGATTGGTGGGTTCGGCAATTGACTAATCCCGTTAATAATCGGCGCCCCAGACAT
GGCCTTCCCTCGTTTGAACAATCTAAGCTTTTGACTTCTCCCCCCATCCTTTCTCCT
CCTCCTCGCCTCCTCCTTCGTTGAGGGCGGCGTGGGGACAGGCTGAACTGTGTAC
CCCCCCCTCTCCGGAAATTTGGCCCATGCCGGACCATCCGTAGATCTGGCCATCTT
TTCCCTTCACTTAGCAGGGGTGTCCTCGATTTTAGGAGCAATCAACTTTATCACCA
CTGTTATTAACATAAAACCCCCTGCTATCCCTATGCGTCACACACCTCTATTTATC
TGATCCCTTCTCATCACTGCTGTACTCCTACTCCTATCGCTTCCAGTACTTGCTGCC
GGGATCACCATACTTTTAACGGACCGAAACCT"/>
<sequence id="seq_Abit_Mito_Apisto_302615"
taxon="Abit_Mito_Apisto_302615" totalcount="4"
value="CCCTAATTGACCTTCCCGCCCCCTCCAACATCTCCGCCTGATGAAATTTCG
GCTCCCTCTTAGGCCTCTGCTTGATTTCACAAATCCTGACAGGCCTATTCCTCTCC
ATGCATTACACTGCTGACATCAACACAGCCTTTTCATCCGTCGCCCACATCTCTCG
AGATGTAAACTACGGGTGATTAATTCGAAATATTCATGCTAATGGGGCATCTTTT
TTCTTCATTTGCATTTATCTGCACATCGGACGAGGCCTTTACTTCGGCTCCTATCT
CTACAAAGAGACATGAAATATCGGAGTAGTACTACTGTTACTACTAATAATAACC
GCTTTTACAGGCTATGTCCTCCCATGGGGACAAATATCATTTTGAGGGGCCACCG
TCATCACCAACCTACTATCAGCAGCCCCCTACATCGGGGACTCCCTAGTTCAATG
AATTTGGGGCGGCTTCTCAGTTGACAGCGCTACCTTAACCCGCTTCTTCACCTTCC
ACTTCATTCTTCCCTTCGCTATTACAGCCATAACCCTGGTTCACCTAATCTTTCTTC
ACGAAACAGGCTCTACAAATCCAATTGGGTTAAACCCAAACGCAGATAAAGTTT
CCTTCCACCCATTCTTCTCCCTTAAAGACCTTCTCGGGTTTCTAATTCTGCTCACA
GCCCTGATATTTGGTGCTTTTGCCGGACTAGTGGGCGCCGCGTTAAGCACACTGA
TTCGAGCAGAACTTACTCAGTCCGGCTCCCTTTTTGAAGACGATCAGCTTTACAA
CGTAATCGTAACTGCACACGCCTTCGTAATAATTTTCTTTATGGTTATACCAATCA
TGATTGGTGGGTTCGGCAATTGACTAATCCCGTTAATAATCGGCGCCCCAGACAT
GGCCTTCCCTCGTTTGAACAATCTAAGCTTTTGACTTCTCCCCCCATCCTTTCTCCT
CCTCCTCGCCTCCTCCTTCGTTGAGGGCGGCGTGGGGACAGGCTGAACTGTGTAC
CCCCCCCTCTCCGGAAATTTGGCCCATGCCGGACCATCCGTAGATCTGGCCATCTT
TTCCCTTCACTTAGCAGGGGTGTCCTCGATTTTAGGAGCAATCAACTTTATCACCA
CTGTTATTAACATAAAACCCCCTGCTATCCCTATGCGTCACACACCTCTATTTATC
TGATCCCTTCTCATCACTGCTGTACTCCTACTCCTATCGCTTCCAGTACTTGCTGCC
GGGATCACCATACTTTTAACGGACCGAAACCT"/>

<sequence id="seq_Abit_Mito_Apisto_302616"
taxon="Abit_Mito_Apisto_302616" totalcount="4"
value="CCCTAATTGACCTTCCCGCCCCCTCCAACATCTCCGCCTGATGAAATTTCG
GCTCCCTTTTAGGCCTCTGCTTGATTTCACAAATCCTGACAGGCCTATTCCTCTCC
ATGCATTACACTGCTGACATCAACACAGCCTTTTCATCCGTCGCCCACATCTCTCG
AGATGTAAACTACGGGTGATTAATTCGAAGTATTCATGCTAATGGGGCATCTTTT
TTCTTCATTTGCATTTATCTGCACATCGGACGAGGCCTTTACTTCGGCTCCTATCT
CTACAAAGAGACATGAAATATCGGAGTAGTACTACTATTACTACTAATAATAACC
GCTTTTACAGGCTATGTCCTCCCATGAGGACAAATATCATTTTGAGGGGCCACCG
TCATCACCAACCTACTATCAGCAGCCCCCTACATCGGGGACTCCCTAGTTCAATG
AATTTGGGGCGGCTTCTCAGTTGACAGCGCTACCTTAACCCGCTTCTTCACCTTCC
ACTTCATTCTTCCCTTCGCTATTACAGCCATAACCCTGGTTCACCTAATCTTTCTTC
ACGAAACAGGCTCTACAAATCCAATTGGATTAAACCCAAACGCAGATAAAGTTT
CCTTCCACCCATTCTTCTCCCTTAAAGACCTTCTCGGGTTTCTAATTCTGCTCACA
GCCCTGATATTTGGTGCTTTTGCCGGACTAGTGGGCGCCGCGTTAAGCACACTGA
TTCGAGCAGAACTTACTCAGTCCGGCTCCCTTTTTGAAGACGATCAGCTTTACAA
CGTAATTGTAACTGCACACGCCTTCGTAATAATTTTCTTTATGGTTATACCAATCA
TGATTGGTGGGTTCGGCAATTGACTAATCCCGTTAATAATCGGCGCCCCAGACAT
GGCCTTCCCTCGTTTGAACAATCTAAGCTTTTGACTTCTCCCCCCATCCTTTCTCCT
CCTCCTCGCCTCCTCCTTCGTTGAGGGCGGCGTGGGGACAGGCTGAACTGTGTAC
CCCCCCCTCTCCGGAAATTTGGCCCATGCCGGACCATCCGTAGATCTGGCCATCTT
TTCCCTTCACTTAGCAGGGGTGTCCTCGATTTTAGGAGCAATCAACTTTATCACCA
CTGTTATTAACATAAAACCCCCTGCTATCCCTATGCGTCACACACCTCTATTTATC
TGATCCCTTCTCATCACTGCTGTACTCCTACTCCTATCGCTCCCAGTACTTGCTGC
CGGGATCACCATACTTTTAACGGACCGAAACCT"/>
<sequence id="seq_Acac_Mito_Apisto_300690"
taxon="Acac_Mito_Apisto_300690" totalcount="4"
value="CCCTGATTGATCTCCCCACCCCCTCCAACATTTCTGCTTGATGAAATTTTG
GGTCCCTACTAGGCCTCTGCCTAATTCTTCAAATCTTAACAGGCCTATTCCTTTCA
ATACATTACACTGCTGAAGTCAACACAGCTTTTTTATCCGTCACCCACATCTGCCG
AGACGTAAACTATGGATGACTAATCCGTAATTTACATGCTAATGGGGCATCTTTT
TTCTTCATTTGCATTTATCTGCATATCGGCCGAGGCCTTTACTTCGGCTCTTACCTT
TATAAAGAGACATGAAACATTGGGGTAGTACTCCTACTGCTAGTTATAATAACCG
CTTTCGTAGGCTATGTCCTCCCGTGAGGACAAATATCGTTTTGAGGAGCTACCGTT
ATTACCAACCTTCTGTCAGCAATCCCCTATATTGGAAACTCCCTGGTTCAATGAAT
CTGAGGGGGCTTCTCAATTGACAACGCTACTTTAACCCGATTCTTTGCCATCCACT
TCCTTTCCCCCTTCATCACCGCAGCCATAACCCTAATTCACCTAATCTTTCTTCAC
GAAACAGGCTCCGCTAATCCAATTGGATTAAACCCAAACCTAGATAAAATTTCGT
TTCACCCATTTTTTTCCCTCAAAGATCTCACAGGCTTCCTAATTTTTCTTACACCCC
TAGTATTTGGCGCTTGAGCCGGACTAGTAGGTACCGCATTAAGCATACTAATTCG
AGTAGAACTCACTCAGCCCGGCCCCTTTCTTGAGGACGATCAGCTTTATAATGTA
ATCGTAACTGCACACGCCTTTGTAATAATTTTCTTTATAGTGATGCCAATCATAAT
TGGAGGGTTTGGTAATTGGCTTATCCCAATAATAATTAGTGCTCCAGACATAGCC
TTTCCCCGTATAAATAACATAAGCTTCTGACTCCTCCCCCCATCTTTCCTACTACT
TCTCGCCTCCTCAGCTGTTGAAGCCGGAGTAGGAACAGGCTGAACTGTTTACCCT
CCCCTTTCTGGGAATTTGGCCCATAACGGACCATCCGTGGATCTAGCCATTTTTC
TCTCCATTTAGCAGGAGTATCCTCAATTCTAGGTTCAATCAACTTTATTGCTACCA
TCATTAATATGAAGCCCCCAACTATCTCTATGTACAATTCACCCTTATTTATCTGG
TCCCTCCTCATCACCGCCGTGCTTCTACTTCTTTCATTGCCGGTACTTGCTGCGGG
CATCACTATACTTCTAACAGACCGTAATTT"/>

<sequence id="seq_Acac_Mito_Apisto_300709" taxon="Acac_Mito_Apisto_300709" totalcount="4" value="CCCTGATTGATCTCCCCACCCCCTCCAACATTTCTGCTTGATGAAATTTTG
GGTCCCTACTAGGCCTCTGCCTAATTCTTCAAATCTTAACAGGCCTATTCCTTTCA
ATACATTACACTGCTGAAGTCAACACAGCTTTTTATCCGTCACCCACATCTGCCG
AGACGTAAACTATGGATGACTAATCCGTAATTTACATGCTAATGGGGCATCTTTT
TTCTTCATTTGCATTTATCTGCATATCGGCCGAGGCCTTTACTTCGGCTCTTACCTT
TATAAAGAGACATGAAACATTGGGGTAGTACTCCTACTGCTAGTTATAATAACCG
CTTTCGTAGGCTATGTCCTCCCGTGAGGACAAATATCGTTTTGAGGAGCTACCGTT
ATTACCAACCTTCTGTCAGCAATCCCCTATATTGGAAACTCCCTGGTTCAATGAAT
CTGAGGGGGCTTCTCAATTGACAACGCTACTTTAACCCGATTCTTTGCCATCCACT
TCCTTTCCCCCTTCATCACCGCAGCCATAACCCTAATTCACCTAATCTTTCTTCAC
GAAACAGGCTCCGCTAATCCAATTGGATTAAACCCAAACCTAGATAAAATTTCGT
TTCACCCATTTTTTTCCCTCAAAGATCTCACAGGCTTCCTAATTTTTCTTACACCCC
TAGTATTTGGCGCTTGAGCCGGACTAGTAGGTACCGCATTAAGCATACTAATTCG
AGTAGAACTCACTCAGCCCGGCCCCTTTCTTGAGGACGATCAGCTTTATAATGTA
ATCGTAACTGCACACGCCTTTGTAATAATTTTCTTTATAGTGATGCCAATCATAAT
TGGAGGGTTTGGTAATTGGCTTATCCCAATAATAATTAGTGCTCCAGACATAGCC
TTTCCCCGTATAAATAACATAAGCTTCTGACTCCTCCCCCCATCTTTCCTACTACT
TCTCGCCTCCTCAGCTGTTGAAGCCGGAGTAGGAACAGGCTGAACTGTTTACCCT
CCCCTTTCTGGGAATTTGGCCCATAACGGACCATCCGTGGATCTAGCCATTTTTTC
TCTCCATTTAGCAGGAGTATCCTCAATTCTAGGTTCAATCAACTTTATTGCTACCA
TCATTAATATGAAGCCCCCAACTATCTCTATGTACAATTCACCCTTATTTATCTGG
TCCCTCCTCATCACCGCCGTGCTTCTACTTCTTTCATTGCCGGTACTTGCTGCGGG
CATCACTATACTTCTAACAGACCGTAATTT"/>
<sequence id="seq_Acac_Mito_Apisto_301162" taxon="Acac_Mito_Apisto_301162" totalcount="4" value="CCCTGATTGATCTCCCCACCCCCTCCAACATTTCTGCCTGATGAAATTTTG
GGTCCCTACTAGGCCTCTGCCTAATTCTTCAAATCTTAACAGGCCTATTCCTTTCA
ATACATTACACTGCTGAAGTCAACACAGCTTTTTATCCGTCACCCACATCTGCCG
AGACGTAAACTATGGATGACTAATCCGTAATTTACATGCTAATGGGGCATCTTTT
TTCTTCATTTGCATTTATCTGCATATCGGCCGAGGCCTTTACTTCGGCTCTTACCTT
TATAAAGAGACATGAAACATTGGGGTAGTACTCCTACTGCTAGTTATAATAACCG
CTTTCGTAGGCTATGTCCTCCCGTGAGGACAAATATCGTTTTGAGGAGCTACCGTT
ATTACCAACCTTCTGTCAGCAATCCCCTATATTGGAAACTCCCTGGTTCAATGAAT
CTGAGGGGGCTTCTCAATTGACAACGCTACTTTAACCCGATTCTTTGCCATCCACT
TCCTTTCCCCCTTCATCACCGCAGCCATAACCCTAATTCACCTAATCTTTCTTCAC
GAAACAGGCTCCGCTAATCCAATTGGATTAAACCCAAACCTAGATAAAATTTCGT
TTCACCCATTTTTTTCCCTCAAAGATCTCACAGGCTTCCTAATTTTTCTTACACCCC
TAGTATTTGGCGCTTGAGCCGGACTAGTAGGTACCGCATTAAGCATACTAATTCG
AGTAGAACTCACTCAGCCCGGCCCCTTTCTTGAGGACGATCAGCTTTATAATGTA
ATCGTAACCGCACACGCCTTTGTAATAATTTTCTTTATAGTGATGCCAATCATAAT
TGGAGGGTTTGGTAATTGGCTTATCCCAATAATAATTAGTGCTCCAGACATAGCC
TTTCCCCGTATAAATAACATAAGCTTCTGACTCCTCCCCCCATCTTTCCTACTACT
TCTCGCCTCCTCAGCTGTTGAAGCCGGAGTAGGAACAGGCTGAACTGTTTACCCC
CCCCTTTCTGGGAATTTGGCCCATAACGGACCATCCGTGGATCTAGCCATTTTTTC
TCTCCATTTAGCAGGAGTATCCTCAATTCTAGGTTCAATCAACTTTATTGCTACCA
TCATTAATATGAAGCCCCCAACTATCTCTATGTACAATTCACCCTTATTTATCTGG
TCCCTCCTCATCACCGCCGTGCTTCTACTTCTTTCATTGCCGGTACTTGCTGCGGG
CATCACTATACTTCTAACAGACCGTAATTT"/>

<sequence id="seq_Acac_Mito_Apisto_301422" taxon="Acac_Mito_Apisto_301422" totalcount="4" value="CCCTGATTGATCTCCCCACCCCCTCCAACATTTCTGCCTGATGAAATTTTG
GGTCCCTACTAGGCCTCTGCCTAATTCTTCAAATCTTAACAGGCCTATTCCTTTCA
ATACATTACACTGCTGAAGTCAACACAGCTTTTTATCCGTCACCCACATCTGCCG
AGACGTAAACTATGGATGACTAATCCGTAATTTACATGCTAATGGGGCATCTTTT
TTCTTCATTTGCATTTATCTGCATATCGGCCGAGGCCTTTACTTCGGCTCTTACCTT
TATAAAGAGACATGAAACATTGGGGTAGTACTCCTACTGCTAGTTATAATAACCG
CTTTCGTAGGCTATGTCCTCCCGTGAGGACAAATATCGTTTTGAGGAGCTACCGTT
ATTACCAACCTTCTGTCAGCAATCCCCTATATTGGAAACTCCCTGGTTCAATGAAT
CTGAGGGGGCTTCTCAATTGACAACGCTACTTTAACCCGATTCTTTGCCATCCACT
TCCTTTCCCCCTTCATCACCGCAGCCATAACCCTAATTCACCTAATCTTTCTTCAC
GAAACAGGCTCCGCTAATCCAATTGGATTAAACCCAAACCTAGATAAAATTTCGT
TTCACCCATTTTTTTCCCTCAAAGATCTCACAGGCTTCCTAATTTTTCTTACACCCC
TAGTATTTGGCGCTTGAGCCGGACTAGTAGGTACCGCATTAAGCATACTAATTCG
AGTAGAACTCACTCAGCCCGGCCCCTTTCTTGAGGACGATCAGCTTTATAATGTA
ATCGTAACCGCACACGCCTTTGTAATAATTTTCTTTATAGTGATGCCAATCATAAT
TGGAGGGTTTGGTAATTGGCTTATCCCAATAATAATTAGTGCTCCAGACATAGCC
TTTCCCCGTATAAATAACATAAGCTTCTGACTCCTCCCCCCATCTTTCCTACTACT
TCTCGCCTCCTCAGCTGTTGAAGCCGGAGTAGGAACAGGCTGAACTGTTTACCCT
CCCCTTTCTGGGAATTTGGCCCATAACGGACCATCCGTGGATCTAGCCATTTTTTC
TCTCCATTTAGCAGGAGTATCCTCAATTCTAGGTTCAATCAACTTTATTGCTACCA
TCATTAATATGAAGCCCCCAACTATCTCTATGTACAATTCACCCTTATTTATCTGG
TCCCTCCTCATCACCGCCGTGCTTCTACTTCTTTCATTGCCGGTACTTGCTGCGGG
CATCACTATACTTCTAACAGACCGTAATTT"/>
<sequence id="seq_Acac_Mito_Apisto_301505" taxon="Acac_Mito_Apisto_301505" totalcount="4" value="CCCTGATTGATCTCCCCACCCCCTCCAACATTTCTGCCTGATGAAATTTTG
GGTCCCTACTAGGCCTCTGCCTAATTCTTCAAATCTTAACAGGCCTATTCCTTTCA
ATACATTACACTGCTGAAGTCAACACAGCTTTTTATCCGTCACCCACATCTGCCG
AGACGTAAACTATGGATGACTAATCCGTAATTTACATGCTAATGGGGCATCTTTT
TTCTTCATTTGCATTTATCTGCATATCGGCCGAGGCCTTTACTTCGGCTCTTACCTT
TATAAAGAGACATGAAACATTGGGGTAGTACTCCTACTGCTAGTTATAATAACCG
CTTTCGTAGGCTATGTCCTCCCGTGAGGACAAATATCGTTTTGAGGAGCTACCGTT
ATTACCAACCTTCTGTCAGCAATCCCCTATATTGGAAACTCCCTGGTTCAATGAAT
CTGAGGGGGCTTCTCAATTGACAACGCTACTTTAACCCGATTCTTTGCCATCCACT
TCCTTTCCCCCTTCATCACCGCAGCCATAACCCTAATTCACCTAATCTTTCTTCAC
GAAACAGGCTCCGCTAATCCAATTGGATTAAACCCAAACCTAGATAAAATTTCGT
TTCACCCATTTTTTTCCCTCAAAGATCTCACAGGCTTCCTAATTTTTCTTACACCCC
TAGTATTTGGCGCTTGAGCCGGACTAGTAGGTACCGCATTAAGCATACTAATTCG
AGTAGAACTCACTCAGCCCGGCCCCTTTCTTGAGGACGATCAGCTTTATAATGTA
ATCGTAACTGCACACGCCTTTGTAATAATTTTCTTTATAGTGATGCCAATCATAAT
TGGAGGGTTTGGTAATTGGCTTATCCCAATAATAATTAGTGCTCCAGACATAGCC
TTTCCCCGTATAAATAACATAAGCTTCTGACTCCTCCCCCCATCTTTCCTACTACT
TCTCGCCTCCTCAGCTGTTGAAGCCGGAGTAGGAACAGGCTGAACTGTTTACCCT
CCCCTTTCTGGGAATTTGGCCCATAACGGACCATCCGTGGATCTAGCCATTTTTTC
TCTCCATTTAGCAGGAGTATCCTCAATTCTAGGTTCAATCAACTTTATTGCTACCA
TCATTAATATGAAGCCCCCAACTATCTCTATGTACAATTCACCCTTATTTATCTGG
TCCCTCCTCATCACCGCCGTGCTTCTACTTCTTTCATTACCGGTACTTGCTGCGGG
CATCACTATACTTCTAACAGACCGTAATTT"/>

<sequence id="seq_Acac_Mito_Apisto_302195"
taxon="Acac_Mito_Apisto_302195" totalcount="4"
value="CCCTGATTGATCTCCCCACCCCCTCCAACATTTCTGCCTGATGAAATTTTG
GGTCCCTACTAGGCCTCTGCCTAATTCTTCAAATCTTAACAGGCCTATTCCTTTCA
ATACATTACACTGCTGAAGTCAACACAGCTTTTTATCCGTCACCCACATCTGCCG
AGACGTAAACTATGGATGACTAATCCGTAATTTACATGCTAATGGGGCATCTTTT
TTCTTCATTTGCATTTATCTGCATATCGGCCGAGGCCTTTACTTCGGCTCTTACCTT
TATAAAGAGACATGAAACATTGGGGTAGTACTCCTACTGCTAGTTATAATAACCG
CTTTCGTAGGCTATGTCCTCCCGTGAGGACAAATATCGTTTTGAGGAGCTACCGTT
ATTACCAACCTTCTGTCAGCAATCCCCTATATTGGAAACTCCCTGGTTCAATGAAT
CTGAGGGGGCTTCTCAATTGACAACGCTACTTTAACCCGATTCTTTGCCATCCACT
TCCTTTCCCCCTTCATCACCGCAGCCATAACCCTAATTCACCTAATCTTTCTTCAC
GAAACAGGCTCCGCTAATCCAATTGGATTAAACCCAAACCTAGATAAAATTTCGT
TTCACCCATTTTTTTCCCTCAAAGATCTCACAGGCTTCCTAATTTTTCTTACACCCC
TAGTATTTGGCGCTTGAGCCGGACTAGTAGGTACCGCATTAAGCATACTAATTCG
AGTAGAACTCACTCAGCCCGGCCCCTTTCTTGAGGACGATCAGCTTTATAATGTA
ATCGTAACCGCACACGCCTTTGTAATAATTTTCTTTATAGTGATGCCAATCATAAT
TGGAGGGTTTGGTAATTGGCTTATCCCAATAATAATTAGTGCTCCAGACATAGCC
TTTCCCCGTATAAATAACATAAGCTTCTGACTCCTCCCCCCATCTTTCCTACTACT
TCTCGCCTCCTCAGCTGTTGAAGCCGGAGTAGGAACAGGCTGAACTGTTTACCCT
CCCCTTTCTGGGAATTTGGCCCATAACGGACCATCCGTGGATCTAGCCATTTTTTC
TCTCCATTTAGCAGGAGTATCCTCAATTCTAGGTTCAATCAACTTTATTGCTACCA
TCATTAATATGAAGCCCCCAACTATCTCTATGTACAATTCACCCTTATTTATCTGG
TCCCTCCTCATCACCGCCGTGCTTCTACTTCTTTCATTGCCGGTACTTGCTGCGGG
CATCACTATACTTCTAACAGACCGTAATTT"/>
<sequence id="seq_Acac_Mito_Apisto_302196"
taxon="Acac_Mito_Apisto_302196" totalcount="4"
value="CCCTGATTGATCTCCCCACCCCCTCCAACATTTCTGCCTGATGAAATTTTG
GGTCCCTACTAGGCCTCTGCCTAATTCTTCAAATCTTAACAGGCCTATTCCTTTCA
ATACATTACACTGCTGAAGTCAACACAGCTTTTTATCCGTCACCCACATCTGCCG
AGACGTAAACTATGGATGACTAATCCGTAATTTACATGCTAATGGGGCATCTTTT
TTCTTCATTTGCATTTATCTGCATATCGGCCGAGGCCTTTACTTCGGCTCTTACCTT
TATAAAGAGACATGAAACATTGGGGTAGTACTCCTACTGCTAGTTATAATAACCG
CTTTCGTAGGCTATGTCCTCCCGTGAGGACAAATATCGTTTTGAGGAGCTACCGTT
ATTACCAACCTTCTGTCAGCAATCCCCTATATTGGAAACTCCCTGGTTCAATGAAT
CTGAGGGGGCTTCTCAATTGACAACGCTACTTTAACCCGATTCTTTGCCATCCACT
TCCTTTCCCCCTTCATCACCGCAGCCATAACCCTAATTCACCTAATCTTTCTTCAC
GAAACAGGCTCCGCTAATCCAATTGGATTAAACCCAAACCTAGATAAAATTTCGT
TTCACCCATTTTTTTCCCTCAAAGATCTCACAGGCTTCCTAATTTTTCTTACACCCC
TAGTATTTGGCGCTTGAGCCGGACTAGTAGGTACCGCATTAAGCATACTAATTCG
AGTAGAACTCACTCAGCCCGGCCCCTTTCTTGAGGACGATCAGCTTTATAATGTA
ATCGTAACTGCACACGCCTTTGTAATAATTTTCTTTATAGTGATGCCAATCATAAT
TGGAGGGTTTGGTAATTGGCTTATCCCAATAATAATTAGTGCTCCAGACATAGCC
TTTCCCCGTATAAATAACATAAGCTTCTGACTCCTCCCCCCATCTTTCCTACTACT
TCTCGCCTCCTCAGCTGTTGAAGCCGGAGTAGGAACAGGCTGAACTGTTTACCCC
CCCCTTTCTGGGAATTTGGCCCATAACGGACCATCCGTGGATCTAGCCATTTTTTC
TCTCCATTTAGCAGGAGTATCCTCAATTCTAGGTTCAATCAACTTTATTGCTACCA
TCATTAATATGAAGCCCCCAACTATCTCTATGTACAATTCACCCTTATTTATCTGG
TCCCTCCTCATCACCGCCGTGCTTCTACTTCTTTCATTGCCGGTACTTGCTGCGGG
CATCACTATAATTCTAACAGACCGTAATTT"/>

<sequence id="seq_Acac_Mito_Apisto_302197"
taxon="Acac_Mito_Apisto_302197" totalcount="4"
value="CCCTGATTGATCTCCCCACCCCCTCCAACATTTCTGCCTGATGAAATTTTG
GGTCCCTACTAGGCCTCTGCCTAATTCTTCAAATCTTAACAGGCCTATTCCTTTCA
ATACATTACACTGCTGAAGTCAACACAGCTTTTTATCCGTCACCCACATCTGCCG
AGACGTAAACTATGGATGACTAATCCGTAATTTACATGCTAATGGGGCATCTTTT
TTCTTCATTTGCATTTATCTGCATATCGGCCGAGGCCTTTACTTCGGCTCTTACCTT
TATAAAGAGACATGAAACATTGGGGTAGTACTCCTACTGCTAGTTATAATAACCG
CTTTCGTAGGCTATGTCCTCCCGTGAGGACAAATATCGTTTTGAGGAGCTACCGTT
ATTACCAACCTTCTGTCAGCAATCCCCTATATTGGAAACTCCCTGGTTCAATGAAT
CTGAGGGGGCTTCTCAATTGACAACGCTACTTTAACCCGATTCTTTGCCATCCACT
TCCTTTCCCCCTTCATCACCGCAGCCATAACCCTAATTCACCTAATCTTTCTTCAC
GAAACAGGCTCCGCTAATCCAATTGGATTAAACCCAAACCTAGATAAAATTTCGT
TTCACCCATTTTTTTCCCTCAAAGATCTCACAGGCTTCCTAATTTTTCTTACACCCC
TAGTATTTGGCGCTTGAGCCGGACTAGTAGGTACCGCATTAAGCATACTAATTCG
AGTAGAACTCACTCAGCCCGGCCCCTTTCTTGAGGACGATCAGCTTTATAATGTA
ATCGTAACTGCACACGCCTTTGTAATAATTTTCTTTATAGTGATGCCAATCATAAT
TGGAGGGTTTGGTAATTGGCTTATCCCAATAATAATTAGTGCTCCAGACATAGCC
TTTCCCCGTATAAATAACATAAGCTTCTGACTCCTCCCCCCATCTTTCCTACTACT
TCTCGCCTCCTCAGCTGTTGAAGCCGGAGTAGGAACAGGCTGAACTGTTTACCCC
CCCCTTTCTGGGAATTTGGCCCATAACGGACCATCCGTGGATCTAGCCATTTTTTC
TCTCCATTTAGCAGGAGTATCCTCAATTCTAGGTTCAATCAACTTTATTGCTACCA
TCATTAATATGAAGCCCCCAACTATCTCTATGTACAATTCACCCTTATTTATCTGG
TCCCTCCTCATCACCGCCGTGCTTCTACTTCTTTCATTGCCGGTACTTGCTGCGGG
CATCACTATACTTCTAACAGACCGTAATTT"/>
<sequence id="seq_Acac_Mito_Apisto_302302"
taxon="Acac_Mito_Apisto_302302" totalcount="4"
value="CCCTGATTGATCTCCCCACCCCCTCCAACATTTCTGCCTGATGAAATTTTG
GGTCCCTACTAGGCCTCTGCCTAATTCTTCAAATCTTAACAGGCCTATTCCTTTCA
ATACATTACACTGCTGAAGTCAACACAGCTTTTTATCCGTCACCCACATCTGCCG
AGACGTAAACTATGGATGACTAATCCGTAATTTACATGCTAATGGGGCATCTTTT
TTCTTCATTTGCATTTATCTGCATATCGGCCGAGGCCTTTACTTCGGCTCTTACCTT
TATAAAGAGACATGAAACATTGGGGTAGTACTCCTACTGCTAGTTATAATAACCG
CTTTCGTAGGCTATGTCCTCCCGTGAGGACAAATATCGTTTTGAGGAGCTACCGTT
ATTACCAACCTTCTGTCAGCAATCCCCTATATTGGAAACTCCCTGGTTCAATGAAT
CTGAGGGGGCTTCTCAATTGACAACGCTACTTTAACCCGATTCTTTGCCATCCACT
TCCTTTCCCCCTTCATCACCGCAGCCATAACCCTAATTCACCTAATCTTTCTTCAC
GAAACAGGCTCCGCTAATCCAATTGGATTAAACCCAAACCTAGATAAAATTTCGT
TTCACCCATTTTTTTCCCTCAAAGATCTCACAGGCTTCCTAATTTTTCTTACACCCC
TAGTATTTGGCGCTTGAGCCGGACTAGTAGGTACCGCATTAAGCATACTAATTCG
AGTAGAACTCACTCAGCCCGGCCCCTTTCTTGAGGACGATCAGCTTTATAATGTA
ATCGTAACTGCACACGCCTTTGTAATAATTTTCTTTATAGTGATGCCAATCATAAT
TGGAGGGTTTGGTAATTGGCTTATCCCAATAATAATTAGTGCTCCAGACATAGCC
TTTCCCCGTATAAATAACATAAGCTTCTGACTCCTCCCCCCATCTTTCCTACTACT
TCTCGCCTCCTCAGCTGTTGAAGCCGGAGTAGGAACAGGCTGAACTGTTTACCCC
CCCCTTTCTGGGAATTTGGCCCATAACGGACCATCCGTGGATCTAGCCATTTTTTC
TCTCCATTTAGCAGGAGTATCCTCAATTCTAGGTTCAATCAACTTTATTGCTACCA
TAATTAATATGAAGCCCCCAACTATCTCTATGTACAATTCACCCTTATTTATCTGG
TCCCTCCTCATCACCGCCGTGCTTCTACTTCTTTCATTGCCGGTACTTGCTGCGGG
CATCACTATACTTCTAACAGACCGTAATTT"/>

<sequence id="seq_Acac_Mito_Apisto_302303"
taxon="Acac_Mito_Apisto_302303" totalcount="4"
value="CCCTGATTGATCTCCCCACCCCCTCCAACATTTCTGCCTGATGAAATTTTG
GGTCCCTACTAGGCCTCTGCCTAATTCTTCAAATCTTAACAGGCCTATTCCTTTCA
ATACATTACACTGCTGAAGTCAACACAGCTTTTTATCCGTCACCCACATCTGCCG
AGACGTAAACTATGGATGACTAATCCGTAATTTACATGCTAATGGGGCATCTTTT
TTCTTCATTTGCATTTATCTGCATATCGGCCGAGGCCTTTACTTCGGCTCTTACCTT
TATAAAGAGACATGAAACATTGGGGTAGTACTCCTACTGCTAGTTATAATAACCG
CTTTCGTAGGCTATGTCCTCCCGTGAGGACAAATATCGTTTTGAGGAGCTACCGTT
ATTACCAACCTTCTGTCAGCAATCCCCTATATTGGAAACTCCCTGGTTCAATGAAT
CTGAGGGGGCTTCTCAATTGACAACGCTACTTTAACCCGATTCTTTGCCATCCACT
TCCTTTCCCCCTTCATCACCGCAGCCATAACCCTAATTCACCTAATCTTTCTTCAC
GAAACAGGCTCCGCTAATCCAATTGGATTAAACCCAAACCTAGATAAAATTTCGT
TTCACCCATTTTTTTCCCTCAAAGATCTCACAGGCTTCCTAATTTTTCTTACACCCC
TAGTATTTGGCGCTTGAGCCGGACTAGTAGGTACCGCATTAAGCATACTAATTCG
AGTAGAACTCACTCAGCCCGGCCCCTTTCTTGAGGACGATCAGCTTTATAATGTA
ATCGTAACTGCACACGCCTTTGTAATAATTTTCTTTATAGTGATGCCAATCATAAT
TGGAGGGTTTGGTAATTGGCTTATCCCAATAATAATTAGTGCTCCAGACATAGCC
TTTCCCCGTATAAATAACATAAGCTTCTGACTCCTCCCCCCATCTTTCCTACTACT
TCTCGCCTCCTCAGCTGTTGAAGCCGGAGTAGGAACAGGCTGAACTGTTTACCCC
CCCCTTTCTGGGAATTTGGCCCATAACGGACCATCCGTGGATCTAGCCATTTTTTC
TCTCCATTTAGCAGGAGTATCCTCAATTCTAGGTTCAATCAACTTTATTGCTACCA
TCATTAATATGAAGCCCCCAACTATCTCTATGTACAATTCACCCTTATTTATCTGG
TCCCTCCTCATCACCGCCGTGCTTCTACTTCTTTCATTGCCGGTACTTGCTGCGGG
CATCACTATACTTCTAACAGACCGTAATTT"/>
<sequence id="seq_Acar_Mito_Apisto_302364"
taxon="Acar_Mito_Apisto_302364" totalcount="4"
value="CACTAATTGACCTCCCCACCCCCTCCAACATCTCTGCTTGATGGAATTTTG
GGTCTCTATTAGGATTATGCTTAATTTCCCAGATCCTAACAGGCTTATTTCTTTCC
ATACACTACACTGCCGACATCAATACAGCTTTTCATCCATCACTCACATCTCCCG
AGATGTAAACTACGGATGACTAATCCGAAATTTACATGCCAATGGAGCATCCTTT
TTCTTCATTTGCATCTATCTACACATTGCACGAGGCCTCTATTACAGCTCATTTCT
CTACAAAGGAACATGAAACATTGGGGTAGCCCTTCTACTATTAGTTATAATAACC
GCTTTTGTAGGCTATGTCCTCCCGTGAGGGCAAATATCATTTTGAGGGGCCACAG
TCATCACCAACCTACTTTCCGCTGTCCCTTACATTGGAAACTCGCTAGTCCAATGA
ATCTGGGGTGGCTTCTCAGTCGACAATGCCACTCTTACCCGATTCTTCGCTATCCA
TTTCCTGCTTCCATTTGTTATCGCAGCCCTAACTCTAATTCACCTAATTTTTCTTCA
TGAAACAGGGTCCACTAACCCAATTGGGCTAAACCCAAACACAGACAAAATTTC
CTTTCACCCATACTTCTCTTACAAAGACCTTCTCGGTTTCTTAATCCTACTCTTGGC
TTTAATATTCGGTGCCTGAGCTGGAATAGCAGGGACCGCGCTAAGCATACTAATT
CGAGCTGAATTAACTCAGCCCGGCTCCTTTTTTGGGGACGACCAAATCTATAATG
TAATCGTTACTGCACATGCCTTCGTAATAATCTTCTTTATAGTAATGCCAATTATA
ATTGGCGGGTTTGGTAATTGATTAATCCCACTCATAATTGGTGCCCCAGACATGG
CTTTCCCTCGTATAAATAACATAAGCTTTTGACTACTACCCCCCTCCTTCCTCCTC
CTCCTCGCCTCTTCAACTGTTGAAGCCGGAGTGGGGACAGGCTGGACCGTGTACC
CCCCCCTCTCAGGGAACTTAGCCCACGATGGCCCATCGGTAGACCTAGCCATCTT
CTCCCTCCACCTAGCGGGAGTATCTTCAATCTTAGGTGCAATCAATTTCATCACCA
CTATTATTAACATAAAACCTCCAACCACCTCCCTGTATAATACACCATTATTTATT
TGATCTCTTCTTGTCACGGCTGTTCTCCTACTTCTTTCCCTACCAGTCCTTGCTGCA
GGTATTACCATACTTCTAACAGATCGAAACCT"/>

<sequence id="seq_Acar_Mito_Apisto_302365"
taxon="Acar_Mito_Apisto_302365" totalcount="4"
value="CACTAATTGACCTCCCCACCCCCTCCAACATCTCTGCTTGATGGAATTTTG
GGTCTCTATTAGGATTATGCTTAATTTCCCAGATCCTACCAGGCTTATTTCTTTCC
ATACACTACACTGCCGACATCAATACAGCTTTTTCATCCATCACTCACATCTCCCG
AGATGTAAACTACGGATGACTAATCCGAAATTTACATGCCAATGGAGCATCCTTT
TTCTTCATTTGCATCTATCTACACATTGCACGAGGCCTCTATTACAGCTCATTTCT
CTACAAAGGAACATGAAACATTGGGGTAGCCCTTCTACTATTAGTTATAATAACC
GCTTTTGTAGGCTATGTCCTCCCGTGAGGGCAAATATCATTTTGAGGGGCCACAG
TCATCACCAACCTACTTTCCGCTGTCCCTTACATTGGAAACTCGCTAGTCCAATGA
ATCTGGGGTGGCTTCTCAGTCGACAATGCCACTCTTACCCGATTCTTCGCTATCCA
TTTCCTGCTTCCATTTGTTATCGCAGCCCTAACTCTAATTCACCTAATTTTTCTTCA
TGAAACAGGGTCCACTAACCCAATTGGGCTAAACCCAAACACAGACAAAATTTC
CTTTCACCCATACTTCTCTTACAAAGACCTTCTCGGTTTCTTAATCCTACTCTTGGC
TTTAATATTCGGTGCCTGAGCTGGAATAGCAGGGACCGCGCTAAGCATACTAATT
CGAGCTGAATTAACTCAGCCCGGCTCCTTTTTTGGGGACGACCAAATCTATAATG
TAATCGTTACTGCACATGCCTTCGTAATAATCTTCTTTATAGTAATGCCAATTATA
ATTGGCGGGTTTGGTAATTGATTAATCCCACTCATAATTGGTGCCCCAGACATGG
CTTTCCCTCGTATAAATAACATAAGCTTTTGACTACTACCCCCCTCCTTCCTCCTC
CTCCTCGCCTCTTCAACTGTTGAAGCCGGAGTGGGGACAGGCTGGACCGTGTACC
CCCCCCTCTCAGGGAACTTAGCCCACGATGGCCCATCGGTAGACCTAGCCATCTT
CTCCCTCCACCTAGCGGGAGTATCTTCAATCTTAGGTGCAATCAATTTCATCACCA
CTATTATTAACATAAAACCTCCAACCACCTCCCTGTATAATACACCATTATTTATT
TGATCTCTTCTTGTCACGGCTGTTCTCCTACTTCTTTCCCTACCAGTCCTTGCTGCA
GGTATTACCATACTTCTAACAGATCGAAACCT"/>
<sequence id="seq_Acar_Mito_Apisto_302366"
taxon="Acar_Mito_Apisto_302366" totalcount="4"
value="CACTAATTGACCTCCCCACCCCCTCCAACATCTCTGCTTGATGGAATTTTG
GGTCTCTATTAGGATTATGCTTAATTTCCCAGATCCTAACAGGCTTATTTCTTTCC
ATACACTACACTGCCGACATCAATACAGCTTTTTCATCCATCACTCACATCTCCCG
AGATGTAAACTACGGATGACTAATCCGAAATTTACATGCCAATGGAGCATCCTTT
TTCTTCATTTGCATCTATCTACACATTGCACGAGGCCTCTATTACAGCTCATTTCT
CTACAAAGGAACATGAAACATTGGGGTAGCCCTTCTACTATTAGTTATAATAACC
GCTTTTGTAGGCTATGTCCTCCCGTGAGGGCAAATATCATTTTGAGGGGCCACAG
TCATCACCAACCTACTTTCCGCTGTCCCTTACATTGGAAACTCGCTAGTCCAATGA
ATCTGGGGTGGCTTCTCAGTCGACAATGCCACTCTTACCCGATTCTTCGCTATCCA
TTTCCTGCTTCCATTTGTTATCGCAGCCCTAACTCTAATTCACCTAATTTTTCTTCA
TGAAACAGGGTCCACTAACCCAATTGGGCTAAACCCAAACACAGACAAAATTTC
CTTTCACCCATACTTCTCTTACAAAGACCTTCTCGGTTTCTTAATCCTACTCTTGGC
TTTAATATTCGGTGCCTGAGCTGGAATAGCAGGGACCGCGCTAAGCATACTAATT
CGAGCTGAATTAACTCAGCCCGGCTCCTTTTTTGGGGACGACCAAATCTATAATG
TAATCGTTACTGCACATGCCTTCGTAATAATCTTCTTTATAGTAATGCCAATTATA
ATTGGCGGGTTTGGTAATTGATTAATCCCACTCATAATTGGTGCCCCAGACATGG
CTTTCCCTCGTATAAATAACATAAGCTTTTGACTACTACCCCCCTCCTTCCTCCTC
CTCCTCGCCTCTTCAACTGTTGAAGCCGGAGTGGGGACAGGCTGGACCGTGTACC
CCCCCCTCTCAGGGAACTTAGCCCACGATGGCCCATCGGTAGACCTAGCCATCTT
CTCCCTCCACCTAGCGGGAGTATCTTCAATCTTAGGTGCAATCAATTTCATCACCA
CTATTATTAACATAAAACCTCCAACCACCTCCCTGTATAATACACCATTATTTATT
TGATCTCTTCTTGTCACGGCTGTTCTCCTACTTCTTTCCCTACCAGTCCTTGCTGCA
GGTATTACCATACTTCTAACAGATCGAAACCT"/>

<sequence id="seq_Acar_Mito_Apisto_302367"
taxon="Acar_Mito_Apisto_302367" totalcount="4"
value="CACTAATTGACCTCCCCACCCCCTCCAACATCTCTGCTTGATGGAATTTTG
GGTCTCTATTAGGATTATGCTTAATTTCCCAGATCCTAACAGGCTTATTTCTTTCC
ATACACTACACTGCCGACATCAATACAGCTTTTTCATCCATCACTCACATCTCCCG
AGATGTAAACTACGGATGACTAATCCGAAATTTACATGCCAATGGAGCATCCTTT
TTCTTCATTTGCATCTATCTACACATTGCACGAGGCCTCTATTACAGCTCATTTCT
CTACAAAGGAACATGAAACATTGGGGTAGCCCTTCTACTATTAGTTATAATAACC
GCTTTTGTAGGCTATGTCCTCCCGTGAGGGCAAATATCATTTTGAGGGGCCACAG
TCATCACCAACCTACTTTCCGCTGTCCCTTACATTGGAAACTCGCTAGTCCAATGA
ATCTGGGGTGGCTTCTCAGTCGACAATGCCACTCTTACCCGATTCTTCGCTATCCA
TTTCCTGCTTCCATTTGTTATCGCAGCCCTAACTCTAATTCACCTAATTTTTCTTCA
TGAAACAGGGTCCACTAACCCAATTGGGCTAAACCCAAACACAGACAAAATTTC
CTTTCACCCATACTTCTCTTACAAAGACCTTCTCGGTTTCTTAATCCTACTCTTGGC
TTTAATATTCGGTGCCTGAGCTGGAATAGCAGGGACCGCGCTAAGCATACTAATT
CGAGCTGAATTAACTCAGCCCGGCTCCTTTTTTGGGGACGACCAAATCTATAATG
TAATCGTTACTGCACATGCCTTCGTAATAATCTTCTTTATAGTAATGCCAATTATA
ATTGGCGGGTTTGGTAATTGATTAATCCCACTCATAATTGGTGCCCCAGACATGG
CTTTCCCTCGTATAAATAACATAAGCTTTTGACTACTACCCCCCTCCTTCCTCCTC
CTCCTCGCCTCTTCAACTGTTGAAGCCGGAGTGGGGACAGGCTGGACCGTGTACC
CCCCCCTCTCAGGGAACTTAGCCCACGATGGCCCATCGGTAGACCTAGCCATCTT
CTCCCTCCACCTAGCGGGAGTATCTTCAATCTTAGGTGCAATCAATTTCATCACCA
CTATTATTAACATAAAACCTCCAACCACCTCCCTGTATAATACACCATTATTTATT
TGATCTCTTCTTGTCACGGCTGTTCTCCTACTTCTTTCCCTACCAGTCCTTGCTGCA
GGTATTACCATACTTCTAACAGATCGAAACCT"/>
<sequence id="seq_Acin_Mito_Apisto_301616"
taxon="Acin_Mito_Apisto_301616" totalcount="4"
value="CACTAATTGACCTCCCCACCCCCCCCAACATCTCTGCTTGATGAAATTTCG
GGTCTCTACTAGGACTATGCTTAATTTCCCAAATCCTAACAGGCTTATTTCTTTCC
ATACACTACACTGCCGACATCAACACAGCTTTTTCATCCATCACTCACATTTGCCG
AGACGTAAACTACGGATGGCTAATCCGAAATTTACATGCCAACGGAGCATCCTTT
TTTTTCATTTGTATCTATTTACACATTGCACGAGGCCTCTATTACGGCTCATTTCTC
TACAAAGAAACATGAAACATCGGGGTAATCCTTCTACTATTAGTAATAATAACCG
CTTTTGTAGGCTATGTACTCCCATGAGGACAAATATCGTTTTGAGGGGCTACAGT
CATTACCAACCTACTTTCCGCTATTCCTTACATTGGAAATTCACTAGTTCAATGAA
TCTGAGGTGGCTTCTCAGTCGACAATGCCACCCTTACACGATTCTTCGCTATCCAT
TTTCTGCTTCCATTTATCATCGCAGCCCTAACCCTAATTCACCTAATTTTTCTTCAT
GAGACAGGATCCACCAACCCAATTGGACTAAACCCAAACACAGACAAAATCGCC
TTCCACCCATACTTCTCTTACAAAGACCTTCTCGGTTTCTTAATCCTACTTTTAACT
TTAGTGTTCGGTGCCTGAGCTGGTATAGCAGGAACCGCACTAAGCATGCTAATTC
GAGCTGAACTAACTCAGCCCGGTTCCTTTTTTGGGGACGACCAAATCTATAATGT
AATTGTTACTGCACATGCCTTCGTAATAATTTTTTTTATGGTGATGCCAATTATGA
TTGGCGGATTTGGTAATTGATTAATTCCCCTAATAATTGGTGCCCCAGATATGGCT
TTCCCTCGCATAAATAATATAAGCTTTTGACTACTACCCCCCTCCTTCCTCCTCCT
CCTTGCCTCCTCCACTGTTGAAGCTGGGGTGGGGACAGGCTGAACTGTGTACCCC
CCCCTCTCAGGAAATTTAGCCCACAACGGCCCATCTGTGGATCTAGCCATCTTCTC
CCTTCATCTAGCAGGGGTATCTTCAATTTTGGGGGCAATTAATTTTATCACTACTA
TTATTAACATAAAACCTCCAACCACCTCCCTGTATAATGCACCATTATTTATTTGA
TCTCTCCTTGTCACGGCTGTTCTTCTACTTCTCTCTCTACCAGTCCTTGCTGCAGGT
ATCACTATACTTCTAACAGATCGAAACCT"/>

<sequence id="seq_Acin_Mito_Apisto_301618”
taxon="Acin_Mito_Apisto_301618” totalcount="4"
value="CACTAATTGACCTCCCCACCCCCTCCAACATCTCTGCTTGATGAAATTTCG
GGTCTCTACTAGGACTATGCTTAATTTCCCAAATCCTAACAGGCTTATTTCTTTCC
ATACACTACACTGCCGACATCAACACAGCTTTTCATCCATCACTCACATTTGCCG
AGACGTAAACTACGGATGGCTAATCCGAAATTTACATGCCAACGGAGCATCCTTT
TTTTTCATTTGTATCTATTTACACATTGCACGAGGCCTCTATTACGGCTCATTTCTC
TACAAAGAAACATGAAACATCGGGGTAATCCTTCTACTATTAGTAATAATAACCG
CTTTTGTAGGCTATGTACTCCCATGAGGACAAATATCGTTTTGAGGGGCTACAGT
CATTACCAACCTACTTTCCGCTATTCCTTACATTGGAAATTCACTAGTTCAATGAA
TCTGAGGTGGCTTCTCAGTCGACAATGCCACCCTTACACGATTCTTCGCTATCCAT
TTTCTGCTTCCATTTATCATCGCAGCCCTAACCCTAATTCACCTAATTTTTCTTCAT
GAGACAGGATCCACCAACCCAATTGGACTAAACCCAAACACAGACAAAATCGCC
TTCCACCCATACTTCTCTTACAAAGACCTTCTCGGTTTCTTAATCCTACTTTTAACT
TTAGTGTTCGGTGCCTGAGCTGGTATAGCAGGAACCGCACTAAGCATGCTAATTC
GAGCTGAACTAACTCAGCCCGGTTCCTTTTTTGGGGACGACCAAATCTATAATGT
AATTGTTACTGCACATGCCTTCGTAATAATTTTTTTTATGGTGATGCCAATTATGA
TTGGCGGATTTGGTAATTGATTAATTCCCCTAATAATTGGTGCCCCAGATATGGCT
TTCCCTCGCATAAATAATATAAGCTTTTGACTACTACCCCCCTCCTTCCTCCTCCT
CCTTGCCTCCTCCACTGTTGAAGCTGGGGTGGGGACAGGCTGAACTGTGTACCCC
CCCCTCTCAGGAAATTTAGCCCACAACGGCCCATCTGTGGATCTAGCCATCTTCTC
CCTTCATCTAGCAGGGGTATCTTCAATTTTGGGGGCAATTAATTTTATCACTACTA
TTATTAACATAAAACCTCCAACCACCTCCCTGTATAATGCACCATTATTTATTTGA
TCTCTCCTTGTCACGGCTGTTCTTCTACTTCTCTCTCTACCAGTCCTTGCTGCAGGT
ATCACTATACTTCTAACAGATCGAAACCT"/>
<sequence id="seq_Acin_Mito_Apisto_300371"
taxon="Acin_Mito_Apisto_300371" totalcount="4"
value="CACTAATTGACCTCCCCACCCCCTCCAACATCTCTGCTTGATGAAATTTCG
GGTCTCTACTAGGACTATGCTTAATTTCCCAAATCCTAACAGGCTTATTTCTTTCC
ATACACTACACTGCCGACATCAACACAGCTTTTCATCCATCACTCACATTTGCCG
AGACGTAAACTACGGATGGCTAATCCGAAATTTACATGCCAACGGAGCATCCTTT
TTTTTCATTTGTATCTATTTACACATTGCACGAGGCCTCTATTACGGCTCATTTCTC
TACAAAGAAACATGAAACATCGGGGTAATCCTTCTACTATTAGTAATAATAACCG
CTTTTGTAGGCTATGTACTCCCATGAGGACAAATATCGTTTTGAGGGGCTACAGT
CATTACCAACCTACTTTCCGCTATTCCTTACATTGGAAATTCACTAGTTCAATGAA
TCTGAGGTGGCTTCTCAGTCGACAATGCCACCCTTACACGATTCTTCGCTATCCAT
TTTCTGCTTCCATTTATCATCGCAGCCCTAACCCTAATTCACCTAATTTTTCTTCAT
GAGACAGGATCCACCAACCCAATTGGACTAAACCCAAACACAGACAAAATCGCC
TTCCACCCATACTTCTCTTACAAAGACCTTCTCGGTTTCTTAATCCTACTTTTAACT
TTAGTGTTCGGTGCCTGAGCTGGTATAGCAGGAACCGCACTAAGCATGCTAATTC
GAGCTGAACTAACTCAGCCCGGTTCCTTTTTTGGGGACGACCAAATCTATAATGT
AATTGTTACTGCACATGCCTTCGTAATAATTTTTTTTATGGTGATGCCAATTATGA
TTGGCGGATTTGGTAATTGATTAATTCCCCTAATAATTGGTGCCCCAGATATGGCT
TTCCCTCGCATAAATAATATAAGCTTTTGACTACTACCCCCCTCCTTCCTCCTCCT
CCTTGCCTCCTCCACTGTTGAAGCTGGGGTGGGGACAGGCTGAACTGTGTACCCC
CCCCTCTCAGGAAATTTAGCCCACAACGGCCCATCTGTGGATCTAGCCATCTTCTC
CCTTCATCTAGCAGGGGTATCTTCAATTTTGGGGGCAATTAATTTTATCACTACTA
TTATTAACATAAAACCTCCAACCACCTCCCTGTATAATGCACCATTATTTATTTGA
TCTCTCCTTGTCACGGCTGTTCTTCTACTTCTCTCTCTACCAGTCCTTGCTGCAGGT
ATCACTATACTTCTAACAGATCGaAACCT"/>

<sequence id="seq_Acin_Mito_Apisto_301619”
taxon="Acin_Mito_Apisto_301619” totalcount="4"
value="CACTAATTGACCTCCCCACCCCCTCCAACATCTCTGCTTGATGAAATTTCG
GGTCTCTACTAGGACTATGCTTAATTTCCCAAATCCTAACAGGCTTATTTCTTTCC
ATACACTACACTGCCGACATCAACACAGCTTTTTCATCCATCACTCACATTTGCCG
AGACGTAAACTACGGATGGCTAATCCGAAATTTACATGCCAACGGAGCATCCTTT
TTTTTCATTTGTATCTATTTACACATTGCACGAGGCCTCTATTACGGCTCATTTCTC
TACAAAGAAACATGAAACATCGGGGTAATCCTTCTACTATTAGTAATAATAACCG
CTTTTGTAGGCTATGTACTCCCATGAGGACAAATATCGTTTTGAGGGGCTACAGT
CATTACCAACCTACTTTCCGCTATTCCTTACATTGGAAATTCACTAGTTCAATGAA
TCTGAGGTGGCTTCTCAGTCGACAATGCCACCCTTACACGATTCTTCGCTATCCAT
TTTCTGCTTCCATTTATCATCGCAGCCCTAACCCTAATTCACCTAATTTTTCTTCAT
GAGACAGGATCCACCAACCCAATTGGACTAAACCCAAACACAGACAAAATCGCC
TTCCACCCATACTTCTCTTACAAAGACCTTCTCGGTTTCTTAATCCTACTTTTAACT
TTAGTGTTCGGTGCCTGAGCTGGTATAGCAGGAACCGCACTAAGCATGCTAATTC
GAGCTGAACTAACTCAGCCCGGTTCCTTTTTTGGGGACGACCAAATCTATAATGT
AATTGTTACTGCACATGCCTTCGTAATAATTTTTTTTATGGTGATGCCAATTATGA
TTGGCGGATTTGGTAATTGATTAATTCCCCTAATAATTGGTGCCCCAGATATGGCT
TTCCCTCGCATAAATAATATAAGCTTTTGACTACTACCCCCCTCCTTCCTCCTCCT
CCTTGCCTCCTCCACTGTTGAAGCTGGGGTGGGGACAGGCTGAACTGTGTACCCC
CCCCTCTCAGGAAATTTAGCCCACAACGGCCCATCTGTGGATCTAGCCATCTTCTC
CCTTCATCTAGCAGGGGTATCTTCAATTTTGGGGGCAATTAATTTTATCACTACTA
TTATTAACATAAAACCTCCAACCACCTCCCTGTATAATGCACCATTATTTATTTGA
TCTCTCCTTGTCACGGCTGTTCTTCTACTTCTCTCTCTACCAGTCCTTGCTGCAGGT
ATCACTATACTTCTAACAGATCGAAACCT"/>
<sequence id="seq_Acin_Mito_Apisto_300374"
taxon="Acin_Mito_Apisto_300374" totalcount="4"
value="CACTAATTGACCTCCCCACCCCCTCCAACATCTCTGCTTGATGAAATTTCG
GGTCTCTACTAGGACTATGCTTAATTTCCCAAATCCTAACAGGCTTATTTCTTTCC
ATACACTACACTGCCGACATCAACACAGCTTTTTCATCCATCACTCACATTTGCCG
AGACGTAAACTACGGATGGCTAATCCGAAATTTACATGCCAACGGAGCATCCTTT
TTTTTCATTTGTATCTATTTACACATTGCACGAGGCCTCTATTACGGCTCATTTCTC
TACAAAGAAACATGAAACATCGGGGTAATCCTTCTACTATTAGTAATAATAACCG
CTTTTGTAGGCTATGTACTCCCATGAGGACAAATATCGTTTTGAGGGGCTACAGT
CATTACCAACCTACTTTCCGCTATTCCTTACATTGGAAATTCACTAGTTCAATGAA
TCTGAGGTGGCTTCTCAGTCGACAATGCCACCCTTACACGATTCTTCGCTATCCAT
TTTCTGCTTCCATTTATCATCGCAGCCCTAACCCTAATTCACCTAATTTTTCTTCAT
GAGACAGGATCCACCAACCCAATTGGACTAAACCCAAACACAGACAAAATCGCC
TTCCACCCATACTTCTCTTACAAAGACCTTCTCGGTTTCTTAATCCTACTTTTAACT
TTAGTGTTCGGTGCCTGAGCTGGTATAGCAGGAACCGCACTAAGCATGCTAATTC
GAGCTGAACTAACTCAGCCCGGTTCCTTTTTTGGGGACGACCAAATCTATAATGT
AATTGTTACTGCACATGCCTTCGTAATAATTTTTTTTATGGTGATGCCAATTATGA
TTGGCGGATTTGGTAATTGATTAATTCCCCTAATAATTGGTGCCCCAGATATGGCT
TTCCCTCGCATAAATAATATAAGCTTTTGACTACTACCCCCCTCCTTCCTCCTCCT
CCTTGCCTCCTCCACTGTTGAAGCTGGGGTGGGGACAGGCTGAACTGTGTACCCC
CCCCTCTCAGGAAATTTAGCCCACAACGGCCCATCTGTGGATCTAGCCATCTTCTC
CCTTCATCTAGCAGGGGTATCTTCAATTTTGGGGGCAATTAATTTTATCACTACTA
TTATTAACATAAAACCTCCAACCACCTCCCTGTATAATGCACCATTATTTATTTGA
TCTCTCCTTGTCACGGCTGTTCTTCTACTTCTCTCTCTACCAGTCCTTGCTGCAGGT
ATCACTATATTAATAACAGATCGAAACCT"/>

<sequence id="seq_Acin_Mito_Apisto_300378"
taxon="Acin_Mito_Apisto_300378" totalcount="4"
value="CACTAATTGACCTCCCCACCCCCTCCAACATCTCTGCTTGATGAAATTTCG
GGTCTCTACTAGGACTATGCTTAATTTCCCAAATCCTAACAGGCTTATTTCTTTCC
ATACACTACACTGCCGACATCAACACAGCTTTTTCATCCATCACTCACATTTGCCG
AGACGTAAACTACGGATGGCTAATCCGAAATTTACATGCCAACGGAGCATCCTTT
TTTTTCATTTGTATCTATTTACACATTGCACGAGGCCTCTATTACGGCTCATTTCTC
TACAAAGAAACATGAAACATCGGGGTAATCCTTCTACTATTAGTAATAATAACCG
CTTTTGTAGGCTATGTACTCCCATGAGGACAAATATCGTTTTGAGGGGCTACAGT
CATTACCAACCTACTTTCCGCTATTCCTTACATTGGAAATTCACTAGTTCAATGAA
TCTGAGGTGGCTTCTCAGTCGACAATGCCACCCTTACACGATTCTTCGCTATCCAT
TTTCTGCTTCCATTTATCATCGCAGCCCTAACCCTAATTCACCTAATTTTTCTTCAT
GAGACAGGATCCACCAACCCAATTGGACTAAACCCAAACACAGACAAAATCGCC
TTCCACCCATACTTCTCTTACAAAGACCTTCTCGGTTTCTTAATCCTACTTTTAACT
TTAGTGTTCGGTGCCTGAGCTGGTATAGCAGGAACCGCACTAAGCATGCTAATTC
GAGCTGAACTAACTCAGCCCGGTTCCTTTTTTGGGGACGACCAAATCTATAATGT
AATTGTTACTGCACATGCCTTCGTAATAATTTTTTTTATGGTGATGCCAATTATGA
TTGGCGGATTTGGTAATTGATTAATTCCCCTAATAATTGGTGCCCCAGATATGGCT
TTCCCTCGCATAAATAATATAAGCTTTTGACTACTACCCCCCTCCTTCCTCCTCCT
CCTTGCCTCCTCCACTGTTGAAGCTGGGGTGGGGACAGGCTGAACTGTGTACCCC
CCCCTCTCAGGAAATTTAGCCCACAACGGCCCATCTGTGGATCTAGCCATCTTCTC
CCTTCATCTAGCAGGGGTATCTTCAATTTTGGGGGCAATTAATTTTATCACTACTA
TTATTAACATAAAACCTCCAACCACCTCCCTGTATAATGCACCATTATTTATTTGA
TCTCTCCTTGTCACGGCTGTTCTTCTACTTCTCTCTCTACCAGTCCTTGCTGCAGGT
ATCACTATACTTCTAACAGATCgAAACCT"/>
<sequence id="seq_Acin_Mito_Apisto_300379"
taxon="Acin_Mito_Apisto_300379" totalcount="4"
value="CACTAATTGACCTCCCCACCCCCTCCAACATCTCTGCTTGATGAAATTTCG
GGTCTCTACTAGGACTATGCTTAATTTCCCAAATCCTAACAGGCTTATTTCTTTCC
ATACACTACACTGCCGACATCAACACAGCTTTTTCATCCATCACTCACATTTGCCG
AGACGTAAACTACGGATGGCTAATCCGAAATTTACATGCCAACGGAGCATCCTTT
TTTTTCATTTGTATCTATTTACACATTGCACGAGGCCTCTATTACGGCTCATTTCTC
TACAAAGAAACATGAAACATCGGGGTAATCCTTCTACTATTAGTAATAATAACCG
CTTTTGTAGGCTATGTACTCCCATGAGGACAAATATCGTTTTGAGGGGCTACAGT
CATTACCAACCTACTTTCCGCTATTCCTTACATTGGAAATTCACTAGTTCAATGAA
TCTGAGGTGGCTTCTCAGTCGACAATGCCACCCTTACACGATTCTTCGCTATCCAT
TTTCTGCTTCCATTTATCATCGCAGCCCTAACCCTAATTCACCTAATTTTTCTTCAT
GAGACAGGATCCACCAACCCAATTGGACTAAACCCAAACACAGACAAAATCGCC
TTCCACCCATACTTCTCTTACAAAGACCTTCTCGGTTTCTTAATCCTACTTTTAACT
TTAGTGTTCGGTGCCTGAGCTGGTATAGCAGGAACCGCACTAAGCATGCTAATTC
GAGCTGAACTAACTCAGCCCGGTTCCTTTTTTGGGGACGACCAAATCTATAATGT
AATTGTTACTGCACATGCCTTCGTAATAATTTTTTTTATGGTGATGCCAATTATGA
TTGGCGGATTTGGTAATTGATTAATTCCCCTAATAATTGGTGCCCCAGATATGGCT
TTCCCTCGCATAAATAATATAAGCTTTTGACTACTACCCCCCTCCTTCCTCCTCCT
CCTTGCCTCCTCCACTGTTGAAGCTGGGGTGGGGACAGGCTGAACTGTGTACCCC
CCCCTCTCAGGAaATTTAGCCCACAACGGCCCATCTGTGGATCTAGCCATCTTCTC
CCTTCATCTAGCAGGGGTATCTTCAATTTTGGGGGCaATTAATTTTATCACTACTA
TTATTAACATAAAACCTCCAACCACCTCCCTGTATAATGCACCATTATTTATTTGA
TCTCTCCTTGTCACGGCTGTTCTTCTACTTCTCTCTCTACCAGTCCTTGCTGCAGGT
ATCACTATACTTCTAACAGATCGAAACCT"/>

<sequence id="seq_Acin_Mito_Apisto_300380"
taxon="Acin_Mito_Apisto_300380" totalcount="4"
value="CACTAATTGACCTCCCCACCCCCTCCAACATCTCTGCTTGATGAAATTTCG
GGTCTCTACTAGGACTATGCTTAATTTCCCAAATCCTAACAGGCTTATTTCTTTCC
ATACACTACACTGCCGACATCAACACAGCTTTTCATCCATCACTCACATTTGCCG
AGACGTAAACTACGGATGGCTAATCCGAAATTTACATGCCAACGGAGCATCCTTT
TTTTTCATTTGTATCTATTTACACATTGCACGAGGCCTCTATTACGGCTCATTTCTC
TACAAAGAAACATGAAACATCGGGGTAATCCTTCTACTATTAGTAATAATAACCG
CTTTTGTAGGCTATGTACTCCCATGAGGACAAATATCGTTTTGAGGGGCTACAGT
CATTACCAACCTACTTTCCGCTATTCCTTACATTGGAAATTCACTAGTTCAATGAA
TCTGAGGTGGCTTCTCAGTCGACAATGCCACCCTTACACGATTCTTCGCTATCCAT
TTTCTGCTTCCATTTATCATCGCAGCCCTAACCCTAATTCACCTAATTTTTCTTCAT
GAGACAGGATCCACCAACCCAATTGGACTAAACCCAAACACAGACAAAATCGCC
TTCCACCCATACTTCTCTTACAAAGACCTTCTCGGTTTCTTAATCCTACTTTTAACT
TTAGTGTTCGGTGCCTGAGCTGGTATAGCAGGAACCGCACTAAGCATGCTAATTC
GAGCTGAACTAACTCAGCCCGGTTCCTTTTTTGGGGACGACCAAATCTATAATGT
AATTGTTACTGCACATGCCTTCGTAATAATTTTTTTTATGGTGATGCCAATTATGA
TTGGCGGATTTGGTAATTGATTAATTCCCCTAATAATTGGTGCCCCAGATATGGCT
TTCCCTCGCATAAATAATATAAGCTTTTGACTACTACCCCCCTCCTTCCTCCTCCT
CCTTGCCTCCTCCACTGTTGAAGCTGGGGTGGGGACAGGCTGAACTGTGTACCCC
CCCCTCTCAGGAAATTTAGCCCACAACGGCCCATCTGTGGATCTAGCCATCTTCTC
CCTTCATCTAGCAGGGGTATCTTCAATTTTGGGGGCAATTAATTTTATCACTACTA
TTATTAACATAAAACCTCCAACCACCTCCCTGTATAATGCACCATTATTTATTTGA
TCTCTCCTTGTCACGGCTGTTCTTCTACTTCTCTCTCTACCAGTCCTTGCTGCAGGT
ATCACTATACTTCTAACAgATCgAAACCT"/>
<sequence id="seq_Acin_Mito_Apisto_301628”
taxon="Acin_Mito_Apisto_301628” totalcount="4"
value="CACTAATTGACCTCCCCACCCCCTCCAACATCTCTGCTTGATGAAATTTCG
GGTCTCTACTAGGACTATGCTTAATTTCCCAAATCCTAACAGGCTTATTTCTTTCC
ATACACTACACTGCCGACATCAACACAGCTTTTCATCCATCACTCACATTTGCCG
AGACGTAAACTACGGATGGCTAATCCGAAATTTACATGCCAACGGAGCATCCTTT
TTTTTCATTTGTATCTATTTACACATTGCACGAGGCCTCTATTACGGCTCATTTCTC
TACAAAGAAACATGAAACATCGGGGTAATCCTTCTACTATTAGTAATAATAACCG
CTTTTGTAGGCTATGTACTCCCATGAGGACAAATATCGTTTTGAGGGGCTACAGT
CATTACCAACCTACTTTCCGCTATTCCTTACATTGGAAATTCACTAGTTCAATGAA
TCTGAGGTGGCTTCTCAGTCGACAATGCCACCCTTACACGATTCTTCGCTATCCAT
TTTCTGCTTCCATTTATCATCGCAGCCCTAACCCTAATTCACCTAATTTTTCTTCAT
GAGACAGGATCCACCAACCCAATTGGACTAAACCCAAACACAGACAAAATCGCC
TTCCACCCATACTTCTCTTACAAAGACCTTCTCGGTTTCTTAATCCTACTTTTAACT
TTAGTGTTCGGTGCCTGAGCTGGTATAGCAGGAACCGCACTAAGCATGCTAATTC
GAGCTGAACTAACTCAGCCCGGTTCCTTTTTTGGGGACGACCAAATCTATAATGT
AATTGTTACTGCACATGCCTTCGTAATAATTTTTTTTATGGTGATGCCAATTATGA
TTGGCGGATTTGGTAATTGATTAATTCCCCTAATAATTGGTGCCCCAGATATGGCT
TTCCCTCGCATAAATAATATAAGCTTTTGACTACTACCCCCCTCCTTCCTCCTCCT
CCTTGCCTCCTCCACTGTTGAAGCTGGGGTGGGGACAGGCTGAACTGTGTACCCC
CCCCTCTCAGGAAATTTAGCCCACAACGGCCCATCTGTGGATCTAGCCATCTTCTC
CCTTCATCTAGCAGGGGTATCTTCAATTTTGGGGGCAATTAATTTTATCACTACTA
TTATTAACATAAAACCTCCAACCACCTCCCTGTATAATGCACCATTATTTATTTGA
TCTCTCCTTGTCACGGCTGTTCTTCTACTTCTCTCTCTACCAGTCCTTGCTGCAGGT
ATCACTATACTTCTAACAGATCGAAACCT"/>

<sequence id="seq_Acin_Mito_Apisto_301629”
taxon="Acin_Mito_Apisto_301629” totalcount="4"
value="CACTAATTGACCTCCCCACCCCCTCCAACATCTCTGCTTGATGAAATTTCG
GGTCTCTACTAGGACTATGCTTAATTTCCCAAATCCTAACAGGCTTATTTCTTTCC
ATACACTACACTGCCGACATCAACACAGCTTTTCATCCATCACTCACATTTGCCG
AGACGTAAACTACGGATGGCTAATCCGAAATTTACATGCCAACGGAGCATCCTTT
TTTTTCATTTGTATCTATTTACACATTGCACGAGGCCTCTATTACGGCTCATTTCTC
TACAAAGAAACATGAAACATCGGGGTAATCCTTCTACTATTAGTAATAATAACCG
CTTTTGTAGGCTATGTACTCCCATGAGGACAAATATCGTTTTGAGGGGCTACAGT
CATTACCAACCTACTTTCCGCTATTCCTTACATTGGAAATTCACTAGTTCAATGAA
TCTGAGGTGGCTTCTCAGTCGACAATGCCACCCTTACACGATTCTTCGCTATCCAT
TTTCTGCTTCCATTTATCATCGCAGCCCTAACCCTAATTCACCTAATTTTTCTTCAT
GAGACAGGATCCACCAACCCAATTGGACTAAACCCAAACACAGACAAAATCGCC
TTCCACCCATACTTCTCTTACAAAGACCTTCTCGGTTTCTTAATCCTACTTTTAACT
TTAGTGTTCGGTGCCTGAGCTGGTATAGCAGGAACCGCACTAAGCATGCTAATTC
GAGCTGAACTAACTCAGCCCGGTTCCTTTTTTGGGGACGACCAAATCTATAATGT
AATTGTTACTGCACATGCCTTCGTAATAATTTTTTTATGGTGATGCCAATTATGA
TTGGCGGATTTGGTAATTGATTAATTCCCCTAATAATTGGTGCCCCAGATATGGCT
TTCCCTCGCATAAATAATATAAGCTTTTGACTACTACCCCCCTCCTTCCTCCTCCT
CCTTGCCTCCTCCACTGTTGAAGCTGGGGTGGGGACAGGCTGAACTGTGTACCCC
CCCCTCTCAGGAAATTTAGCCCACAACGGCCCATCTGTGGATCTAGCCATCTTCTC
CCTTCATCTAGCAGGGGTATCTTCAATTTTGGGGGCAATTAATTTTATCACTACTA
TTATTAACATAAAACCTCCAACCACCTCCCTGTATAATGCACCATTATTTATTTGA
TCTCTCCTTGTCACGGCTGTTCTTCTACTTCTCTCTCTACCAGTCCTTGCTGCAGGT
ATCACTATACTTCTAACAGATCGAAACCT"/>
<sequence id="seq_Aere_Mito_Apisto_300401"
taxon="Aere_Mito_Apisto_300401" totalcount="4"
value="CCCTAATTGACCTTCCCGCCCCCTCCAACATCTCCGCCTGATGAAATTTCG
GCTCCCTTCTAGGCCTCTGCCTGGTCTTACAAATCCTAACAGGCCTATTCCTCTCC
ATACATTACACTGCTGACATCAACACAGCCTTTTCATCCGTCGCCCATATCTGTCG
AGACGTAAACTACGGATGATTAATCCGAAGTATACATGCTAATGGGGCATCTTTT
TTCTTTATTTGCATTTATCTGCACATCGGACGAGGCCTTTACTTCGGCTCCTATCTC
TACAAAGAGACATGAAATATTGGAGTAGTACTACTATTACTACTAATAATAACCG
CTTTTACAGGCTATGTCCTTCCATGAGGACAAATATCATTTTGAGGGGCCACCGT
CATCACTAACCTACTATCAGCAGCCCCCTACATCGGGGACTCCCTAGTTCAATGA
ATTTGGGGCGGCTTCTCAGTTGACAGTGCTACCCTAACCCGCTTCTTCACCTTTCA
CTTTCTTCTTCCCTTCGCCATCACAGCCACAACCCTGATTCACCTAATCTTTCTCCA
CGAAACAGGCTCCACAAACCCAATTGGACTAAACCCAAACGCAGATAAAGTTTC
CTTCCACCCATTCTTCTCCCTCAAAGACCTTCTCGGGTTCCTAATTCTGCTCACAG
CCCTGATATTTGGTGCTTTTGCCGGATTAGTGGGCGCCGCGTTAAGCACACTGATT
CGAGCAGAACTTACTCAGTCCGGCTCCCTTTTTGAAGACGACCAGCTTTACAACG
TAATTGTAACTGCACACGCCTTCGTAATAATTTTCTTTATGGTTATACCAATCATG
ATCGGCGGGTTCGGCAATTGACTGATCCCACTAATGATCGGCGCCCCGGACATGG
CCTTCCCTCGTTTAAATAATCTAAGCTTTTGACTTCTCCCCCCATCCTTTCTCCTCC
TCCTCGCCTCCTCCTTCGTTGAGGGCGGCGTGGGGACAGGCTGAACTGTGTACCC
CCCCCTCTCCGGAAATTTGGCCCATGCCGGGCCATCCGTAGATCTGGCCATTTTTT
CCCTTCACTTAGCAGGGGTGTCCTCGATTTTAGGAGCAATCAACTTTATTACCACT
ATTATTAACATAAAACCCCCCGCTATCTCTATGCGCCGCACACCCCTATTTATCTG
ATCCCTCCTCATCACTGCTGTACTCTTACTTCTATCGCTTCCAGTACTTGCTGCTGG
AATCACCATGCTCTTAACGGACCGAAACCT"/>

<sequence id="seq_Aere_Mito_Apisto_300402"
taxon="Aere_Mito_Apisto_300402" totalcount="4"
value="CCCTAATTGACCTTCCCGCCCCCTCCAACATCTCCGCCTGATGAAATTTCG
GCTCCCTTCTAGGCCTCTGCCTGGTCTTACAAATCCTAACAGGCCTATTCCTCTCC
ATACATTACACTGCTGACATCAACACAGCCTTTTCATCCGTCGCCCATATCTGTCG
AGACGTAAACTACGGATGATTAATCCGAAGTATACATGCTAATGGGGCATCTTTT
TTCTTTATTTGCATTTATCTGCACATCGGACGAGGCCTTTACTTCGGCTCCTATCTC
TACAAAGAGACATGAAATATTGGAGTAGTACTACTATTACTACTAATAATAACCG
CTTTTACAGGCTATGTCCTTCCATGAGGACAAATATCATTTTGAGGGGCCACCGT
CATCACTAACCTACTATCAGCAGCCCCCTACATCGGGGACTCCCTAGTTCAATGA
ATTTGGGGCGGCTTCTCAGTTGACAGTGCTACCCTAACCCGCTTCTTCACCTTTCA
CTTTCTTCTTCCCTTCGCCATCACAGCCACAACCCTGATTCACCTAATCTTTCTCCA
CGAAACAGGCTCCACAAACCCAATTGGACTAAACCCAAACGCAGATAAAGTTTC
CTTCCACCCATTCTTCTCCCTCAAAGACCTTCTCGGGTTCCTAATTCTGCTCACAG
CCCTGATATTTGGTGCTTTTGCCGGATTAGTGGGCGCCGCGTTAAGCACACTGATT
CGAGCAGAACTTACTCAGTCCGGCTCCCTTTTTGAAGACGACCAGCTTTACAACG
TAATTGTAACTGCACACGCCTTCGTAATAATTTTCTTTATGGTTATACCAATCATG
ATCGGCGGGTTCGGCAATTGACTGATCCCACTAATGATCGGCGCCCCGGACATGG
CCTTCCCTCGTTTAAATAATCTAAGCTTTTGACTTCTCCCCCCATCCTTTCTCCTCC
TCCTCGCCTCCTCCTTCGTTGAGGGCGGCGTGGGGACAGGCTGAACTGTGTACCC
CCCCCTCTCCGGAAATTTGGCCCATGCCGGGCCATCCGTAGATCTGGCCATTTTTT
CCCTTCACTTAGCAGGGGTGTCCTCGATTTTAGGAGCAATCAACTTTATTACCACT
ATTATTAACATAAAACCCCCCGCTATCTCTATGCGCCGCACACCCCTATTTATCTG
ATCCCTCCTCATCACTGCTGTACTCTTACTTCTATCGCTTCCAGTACTTGCTGCTGG
AATCACCATGCTCTTAACGGACCGAAACCT"/>
<sequence id="seq_Aere_Mito_Apisto_300403"
taxon="Aere_Mito_Apisto_300403" totalcount="4"
value="CCCTAATTGACCTTCCCGCCCCCTCCAACATCTCCGCCTGATGAAATTTCG
GCTCCCTTCTAGGCCTCTGCCTGGTCTTACAAATCCTAACAGGCCTATTCCTCTCC
ATACATTACACTGCTGACATCAACACAGCCTTTTCATCCGTCGCCCATATCTGTCG
AGACGTAAACTACGGATGATTAATCCGAAGTATACATGCTAATGGGGCATCTTTT
TTCTTTATTTGCATTTATCTGCACATCGGACGAGGCCTTTACTTCGGCTCCTATCTC
TACAAAGAGACATGAAATATTGGAGTAGTACTACTATTACTACTAATAATAACCG
CTTTTACAGGCTATGTCCTTCCATGAGGACAAATATCATTTTGAGGGGCCACCGT
CATCACTAACCTACTATCAGCAGCCCCCTACATCGGGGACTCCCTAGTTCAATGA
ATTTGGGGCGGCTTCTCAGTTGACAGTGCTACCCTAACCCGCTTCTTCACCTTTCA
CTTTCTTCTTCCCTTCGCCATCACAGCCACAACCCTGATTCACCTAATCTTTCTCCA
CGAAACAGGCTCCACAAACCCAATTGGACTAAACCCAAACGCAGATAAAGTTTC
CTTCCACCCATTCTTCTCCCTCAAAGACCTTCTCGGGTTCCTAATTCTGCTCACAG
CCCTGATATTTGGTGCTTTTGCCGGATTAGTGGGCGCCGCGTTAAGCACACTGATT
CGAGCAGAACTTACTCAGTCCGGCTCCCTTTTTGAAGACGACCAGCTTTACAACG
TAATTGTAACTGCACACGCCTTCGTAATAATTTTCTTTATGGTTATACCAATCATG
ATCGGCGGGTTCGGCAATTGACTGATCCCACTAATGATCGGCGCCCCGGACATGG
CCTTCCCTCGTTTAAATAATCTAAGCTTTTGACTTCTCCCCCCATCCTTTCTCCTCC
TCCTCGCCTCCTCCTTCGTTGAGGGCGGCGTGGGGACAGGCTGAACTGTGTACCC
CCCCCTCTCCGGAAATTTGGCCCATGCCGGGCCATCCGTAGATCTGGCCATTTTTT
CCCTTCACTTAGCAGGGGTGTCCTCGATTTTAGGAGCAATCAACTTTATTACCACT
ATTATTAACATAAAACCCCCCGCTATCTCTATGCGCCGCACACCCCTATTTATCTG
ATCCCTCCTCATCACTGCTGTACTCTTACTTCTATCGCTTCCAGTACTTGCTGCTGG
AATCACCATGCTCTTAACGGACCGAAACCT"/>

<sequence id="seq_Aere_Mito_Apisto_300405"
taxon="Aere_Mito_Apisto_300405" totalcount="4"
value="CCCTAATTGACCTTCCCGCCCCCTCCAACATCTCCGCCTGATGAAATTTCG
GCTCCCTTCTAGGCCTCTGCCTGGTCTTACAAATCCTAACAGGCCTATTCCTCTCC
ATACATTACACTGCTGACATCAACACAGCCTTTTCATCCGTCGCCCATATCTGTCG
AGACGTAAACTACGGATGATTAATCCGAAGTATACATGCTAATGGGGCATCTTTT
TTCTTTATTTGCATTTATCTGCACATCGGACGAGGCCTTTACTTCGGCTCCTATCTC
TACAAAGAGACATGAAATATTGGAGTAGTACTACTATTACTACTAATAATAACCG
CTTTTACAGGCTATGTCCTTCCATGAGGACAAATATCATTTTGAGGGGCCACCGT
CATCACTAACCTACTATCAGCAGCCCCCTACATCGGGGACTCCCTAGTTCAATGA
ATTTGGGGCGGCTTCTCAGTTGACAGTGCTACCCTAACCCGCTTCTTCACCTTTCA
CTTTCTTCTTCCCTTCGCCATCACAGCCACAACCCTGATTCACCTAATCTTTCTCCA
CGAAACAGGCTCCACAAACCCAATTGGACTAAACCCAAACGCAGATAAAGTTTC
CTTCCACCCATTCTTCTCCCTCAAAGACCTTCTCGGGTTCCTAATTCTGCTCACAG
CCCTGATATTTGGTGCTTTTGCCGGATTAGTGGGCGCCGCGTTAAGCACACTGATT
CGAGCAGAACTTACTCAGTCCGGCTCCCTTTTTGAAGACGACCAGCTTTACAACG
TAATTGTAACTGCACACGCCTTCGTAATAATTTTCTTTATGGTTATACCAATCATG
ATCGGCGGGTTCGGCAATTGACTGATCCCACTAATGATCGGCGCCCCGGACATGG
CCTTCCCTCGTTTAAATAATCTAAGCTTTTGACTTCTCCCCCCATCCTTTCTCCTCC
TCCTCGCCTCCTCCTTCGTTGAGGGCGGCGTGGGGACAGGCTGAACTGTGTACCC
CCCCCTCTCCGGAAATTTGGCCCATGCCGGGCCATCCGTAGATCTGGCCATTTTTT
CCCTTCACTTAGCAGGGGTGTCCTCGATTTTAGGAGCAATCAACTTTATTACCACT
ATTATTAACATAAAACCCCCCGCTATCTCTATGCGCCGCACACCCCTATTTATCTG
ATCCCTCCTCATCACTGCTGTACTCTTACTTCTATCGCTTCCAGTACTTGCTGCTGG
AATCACCATGCTCTTAACGGACCGAAACCT"/>
<sequence id="seq_Aere_Mito_Apisto_300412"
taxon="Aere_Mito_Apisto_300412" totalcount="4"
value="CCCTAATTGACCTTCCCGCCCCCTCCAACATCTCCGCCTGATGAAATTTCG
GCTCCCTTCTAGGCCTCTGCCTGGTCTTACAAATCCTAACAGGCCTATTCCTCTCC
ATACATTACACTGCTGACATCAACACAGCCTTTTCATCCGTCGCCCATATCTGTCG
AGACGTAAACTACGGATGATTAATCCGAAGTATACATGCTAATGGGGCATCTTTT
TTCTTTATTTGCATTTATCTGCACATCGGACGAGGCCTTTACTTCGGCTCCTATCTC
TACAAAGAGACATGAAATATTGGAGTAGTACTACTATTACTACTAATAATAACCG
CTTTTACAGGCTATGTCCTTCCATGAGGACAAATATCATTTTGAGGGGCCACCGT
CATCACTAACCTACTATCAGCAGCCCCCTACATCGGGGACTCCCTAGTTCAATGA
ATTTGGGGCGGCTTCTCAGTTGACAGTGCTACCCTAACCCGCTTCTTCACCTTTCA
CTTTCTTCTTCCCTTCGCCATCACAGCCACAACCCTGATTCACCTAATCTTTCTCCA
CGAAACAGGCTCCACAAACCCAATTGGACTAAACCCAAACGCAGATAAAGTTTC
CTTCCACCCATTCTTCTCCCTCAAAGACCTTCTCGGGTTCCTAATTCTGCTCACAG
CCCTGATATTTGGTGCTTTTGCCGGATTAGTGGGCGCCGCGTTAAGCACACTGATT
CGAGCAGAACTTACTCAGTCCGGCTCCCTTTTTGAAGACGACCAGCTTTACAACG
TAATTGTAACTGCACACGCCTTCGTAATAATTTTCTTTATGGTTATACCAATCATG
ATCGGCGGGTTCGGCAATTGACTGATCCCACTAATGATCGGCGCCCCGGACATGG
CCTTCCCTCGTTTAAATAATCTAAGCTTTTGACTTCTCCCCCCATCCTTTCTCCTCC
TCCTCGCCTCCTCCTTCGTTGAGGGCGGCGTGGGGACAGGCTGAACTGTGTACCC
CCCCCTCTCCGGAAATTTGGCCCATGCCGGGCCATCCGTAGATCTGGCCATTTTTT
CCCTTCACTTAGCAGGGGTGTCCTCGATTTTAGGAGCAATCAACTTTATTACCACT
ATTATTAACATAAAACCCCCCGCTATCTCTATGCGCCGCACACCCCTATTTATCTG
ATCCCTCCTCATCACTGCTGTACTCTTACTTCTATCGCTTCCAGTACTTGCTGCTGG
AATCACCATGCTCTTAACGGACCGAAACCT"/>

<sequence id="seq_Aeun_Mito_Apisto_302554"
taxon="Aeun_Mito_Apisto_302554" totalcount="4"
value="CACTAATTGACCTCCCCACCCCCTCCAACATCTCTGCTTGATGAAATTTCG
GGTCTCTATTAGGACTATGTTTAATCTCCCAGATCCTAACAGGCTTATTTCTTTCC
ATGCACTACACTGCCGACATCAACACAGCTTTTCATCCATCACCCACATCTGCC
GAGACGTAAACTACGGATGGCTAATCCGAAATTTACATGCCAACGGAGCATCCTT
TTTTTTCATTTGTATCTATTTACACATTGCACGAGGCCTCTATTACGGCTCATTTCT
CTACAAAGAAACATGAAACATCGGGGTAATCCTTCTACTATTAGTAATAATAACC
GCTTTTGTAGGCTATGTACTCCCATGAGGACAAATATCGTTTTGGGGGGCTACAG
TCATTACCAACCTACTTTCCGCTGTTCCTTACATTGGAAATTCACTAGTTCAATGA
ATCTGAGGTGGCTTCTCAGTCGACAATGCCACCCTTACACGATTCTTCGCTATCCA
TTTTCTGCTTCCATTTGTTATCGCAGCCCTAACCCTAATTCACCTAATTTTTCTTCA
CGAGACAGGATCCACCAACCCAATTGGACTAAGCCCAAACACAGACAAAATCTC
CTTCCACCCATACTTCTCGTACAAAGACCTTCTCGGCTTCTTAATCCTACTTTTAA
CTCTAATGTTTGGTGCCTGAGCTGGTATAGCAGGAACCGCACTAAGCATGCTAAT
TCGAGCTGAACTAACTCAGCCCGGTTCCTTTTTTGGGGACGACCAAATCTATAAT
GTAATTGTTACTGCACATGCCTTCGTAATAATTTTTTTTATAGTGATGCCAATTAT
GATTGGCGGATTTGGTAATTGATTAATTCCACTAATAATCGGTGCCCCAGATATG
GCTTTCCCTCGCATGAATAATATAAGCTTTTGACTACTACCCCCCTCCTTCCTCCT
CCTCCTTGCCTCCTCCACTGTTGAAGCTGGGGTGGGAACAGGCTGAACTGTGTAC
CCCCCCCTCTCAGGAAATTTAGCTCACGACGGCCCATCTGTAGACCTAGCCATCT
TCTCCCTTCACCTAGCAGGGGTATCTTCAATTTTAGGAGCAATTAATTTTATCACC
ACTATTATCAACATAAAACCTCCAACCACCTCCCTGTATAATGCACCATTATTTAT
TTGATCTCTCCTTGTCACGGCTGTTCTTTTACTTCTCTCTCTACCAGTCCTTGCTGC
GGGTATCACTATACTTCTAACAGATCGAAACCT"/>
<sequence id="seq_Aeun_Mito_Apisto_302555"
taxon="Aeun_Mito_Apisto_302555" totalcount="4"
value="CACTAATTGACCTCCCCACCCCCTCCAACATCTCTGCTTGATGAAATTTCG
GGTCTCTATTAGGACTATGTTTAATCTCCCAGATCCTAACAGGCTTATTTCTTTCC
ATGCACTACACTGCCGACATCAACACAGCTTTTCATCCATCACCCACATCTGCC
GAGACGTAAACTACGGATGGCTAATCCGAAATTTACATGCCAACGGAGCATCCTT
TTTTTTCATTTGTATCTATTTACACATTGCACGAGGCCTCTATTACGGCTCGTTTCT
CTACAAAGAAACATGAAACATCGGGGTAATCCTTCTACTATTAGTAATAATAACC
GCTTTTGTAGGCTATGTACTCCCATGAGGACAAATATCGTTTTGGGGGGCTACAG
TCATTACCAACCTACTTTCCGCTGTTCCTTACATTGGAAATTCACTAGTTCAATGA
ATCTGAGGTGGCTTCTCAGTCGACAATGCCACCCTTACACGATTCTTCGCTATCCA
TTTTCTGCTTCCATTTGTTATCGCAGCCCTAACCCTAATTCACCTAATTTTTCTTCA
CGAGACAGGATCCACCAACCCAATTGGACTAAACCCAAACACAGACAAAATCTC
CTTCCACCCATACTTCTCGTACAAAGACCTTCTCGGCTTCTTAATCCTACTTTTAA
CTCTAATGTTTGGTGCCTGAGCTGGTATAGCAGGAACCGCACTAAGCATGCTAAT
TCGAGCTGAACTAACTCAGCCCGGTTCCTTTTTTGGGGACGACCAAATCTATAAT
GTAATTGTTACTGCACATGCCTTCGTAATAATTTTTTTTATAGTGATGCCAATTAT
GATTGGCGGATTTGGTAATTGATTAATTCCACTAATAATCGGTGCCCCAGATATG
GCTTTCCCTCGCATGAATAATATAAGCTTTTGACTACTACCCCCCTCCTTCCTCCT
CCTCCTTGCCTCCTCCACTGTTGAAGCTGGGGTGGGAACAGGCTGAACTGTGTAC
CCCCCCCTCTCAGGAAATTTAGCTCACGACGGCCCATCTGTAGACCTAGCCATCT
TCTCCCTTCACCTAGCAGGGGTATCTTCAATTTTAGGAGCAATTAATTTTATCACC
ACTATTATCAACATAAAACCTCCAACCACCTCCCTGTATAATGCACCATTATTTAT
TTGATCTCTCCTTGTCACGGCTGTTCTTTTACTTCTCTCTCTACCAGTCCTTGCTGC
GGGTATCACTATACTTCTAACAGATCGAAACCT"/>

<sequence id="seq_Aeun_Mito_Apisto_302556"
taxon="Aeun_Mito_Apisto_302556" totalcount="4"
value="CACTAATTGACCTCCCCACCCCCTCCAACATCTCTGCTTGATGAAATTTCG
GGTCTCTATTAGGACTATGTTTAATCTCCCAGATCCTAACAGGCTTATTTCTTTCC
ATGCACTACACTGCCGACATCAACACAGCTTTTCATCCATCACCCACATCTGCC
GAGACGTAAACTACGGATGGCTAATCCGAAATTTACATGCCAACGGAGCATCCTT
TTTTTTCATTTGTATCTATTTACACATTGCACGAGGCCTCTATTACGGCTCATTTCT
CTACAAAGAAACATGAAACATCGGGGTAATCCTTCTACTATTAGTAATAATAACC
GCTTTTGTAGGCTATGTACTCCCATGAGGACAAATATCGTTTTGGGGGGCTACAG
TCATTACCAACCTACTTTCCGCTGTTCCTTACATTGGAAATTCACTAGTTCAATGA
ATCTGAGGTGGCTTCTCAGTCGACAATGCCACCCTTACACGATTCTTCGCTATCCA
TTTTCTGCTTCCATTTGTTATCGCAGCCCTAACCCTAATTCACCTAATTTTTCTTCA
CGAGACAGGATCCACCAACCCAATTGGACTAAGCCCAAACACAGACAAAATCTC
CTTCCACCCATACTTCTCGTACAAAGACCTTCTCGGCTTCTTAATCCTACTTTTAA
CTCTAATGTTTGGTGCCTGAGCTGGTATAGCAGGAACCGCACTAAGCATGCTAAT
TCGAGCTGAACTAACTCAGCCCGGTTCCTTTTTTGGGGACGACCAAATCTATAAT
GTAATTGTTACTGCACATGCCTTCGTAATAATTTTTTTTATAGTGATGCCAATTAT
GATTGGCGGATTTGGTAATTGATTAATTCCACTAATAATCGGTGCCCCAGATATG
GCTTTCCCTCGCATGAATAATATAAGCTTTTGACTACTACCCCCCTCCTTCCTCCT
CCTCCTTGCCTCCTCCACTGTTGAAGCTGGGGTGGGAACAGGCTGAACTGTGTAC
CCCCCCCTCTCAGGAAATTTAGCTCACGACGGCCCATCTGTAGACCTAGCCATCT
TCTCCCTTCACCTAGCAGGGGTATCTTCAATTTTAGGAGCAATTAATTTTATCACC
ACTATTATCAACATAAAACCTCCAACCACCTCCCTGTATAATGCACCATTATTTAT
TTGATCTCTCCTTGTCACGGCTGTTCTTTTACTTCTCTCTCTACCAGTCCTTGCTGC
GGGTATCACTATACTTCTAACAGATCGAAACCT"/>
<sequence id="seq_Aeun_Mito_Apisto_302557"
taxon="Aeun_Mito_Apisto_302557" totalcount="4"
value="CACTAATTGACCTCCCCACCCCCTCCAACATCTCTGCTTGATGAAATTTCG
GGTCTCTATTAGGACTATGTTTAATCTCCCAGATCCTAACAGGCTTATTTCTTTCC
ATGCACTACACTGCCGACATCAACACAGCTTTTCATCCATCACCCACATCTGCC
GAGACGTAAACTACGGATGGCTAATCCGAAATTTACATGCCAACGGAGCATCCTT
TTTTTTCATTTGTATCTATTTACACATTGCACGAGGCCTCTATTACGGCTCATTTCT
CTACAAAGAAACATGAAACATCGGGGTAATCCTTCTACTATTAGTAATAATAACC
GCTTTTGTAGGCTATGTACTCCCATGAGGACAAATATCGTTTTGGGGGGCTACAG
TCATTACCAACCTACTTTCCGCTGTTCCTTACATTGGAAATTCACTAGTTCAATGA
ATCTGAGGTGGCTTCTCAGTCGACAATGCCACCCTTACACGATTCTTCGCTATCCA
TTTTCTGCTTCCATTTGTTATCGCAGCCCTAACCCTAATTCACCTAATTTTTCTTCA
CGAGACAGGATCCACCAACCCAATTGGACTAAGCCCAAACACAGACAAAATCTC
CTTCCACCCATACTTCTCGTACAAAGACCTTCTCGGCTTCTTAATCCTACTTTTAA
CTCTAATGTTTGGTGCCTGAGCTGGTATAGCAGGAACCGCACTAAGCATGCTAAT
TCGAGCTGAACTAACTCAGCCCGGTTCCTTTTTTGGGGACGACCAAATCTATAAT
GTAATTGTTACTGCACATGCCTTCGTAATAATTTTTTTTATAGTGATGCCAATTAT
GATTGGCGGATTTGGTAATTGATTAATTCCACTAATAATCGGTGCCCCAGATATG
GCTTTCCCTCGCATGAATAATATAAGCTTTTGACTACTACCCCCCTCCTTCCTCCT
CCTCCTTGCCTCCTCCACTGTTGAAGCTGGGGTGGGAACAGGCTGAACTGTGTAC
CCCCCCCTCTCAGGAAATTTAGCTCACGACGGCCCATCTGTAGACCTAGCCATCT
TCTCCCTTCACCTAGCAGGGGTATCTTCAATTTTAGGAGCAATTAATTTTATCACC
ACTATTATCAACATAAAACCTCCAACCACCTCCCTGTATAATGCACCATTATTTAT
TTGATCTCTCCTTGTCACGGCTGTTCTTTTACTTCTCTCTCTACCAGTCCTTGCTGC
GGGTATCACTATACTTCTAACAGATCGAAACCT"/>

<sequence id="seq_Aeun_Mito_Apisto_302558"
taxon="Aeun_Mito_Apisto_302558" totalcount="4"
value="CACTAATTGACCTCCCCACCCCCTCCAACATCTCTGCTTGATGAAATTTCG
GGTCTCTATTAGGACTATGTTTAATCTCCCAGATCCTAACAGGCTTATTTCTTTCC
ATGCACTACACTGCCGACATCAACACAGCTTTTTCATCCATCACCCACATCTGCC
GAGACGTAAACTACGGATGGCTAATCCGAAATTTACATGCCAACGGAGCATCCTT
TTTTTTCATTTGTATCTATTTACACATTGCACGAGGCCTCTATTACGGCTCATTTCT
CTACAAAGAAACATGAAACATCGGGGTAATCCTTCTACTATTAGTAATAATAACC
GCTTTTGTAGGCTATGTACTCCCATGAGGACAAATATCGTTTTGGGGGGCTACAG
TCATTACCAACCTACTTTCCGCTGTTCCTTACATTGGAAATTCACTAGTTCAATGA
ATCTGAGGTGGCTTCTCAGTCGACAATGCCACCCTTACACGATTCTTCGCTATCCA
TTTTCTGCTTCCATTTGTTATCGCAGCCCTAACCCTAATTCACCTAATTTTTCTTCA
CGAGACAGGATCCACCAACCCAATTGGACTAAGCCCAAACACAGACAAAATCTC
CTTCCACCCATACTTCTCGTACAAAGACCTTCTCGGCTTCTTAATCCTACTTTTAA
CTCTAATGTTTGGTGCCTGAGCTGGTATAGCAGGAACCGCACTAAGCATGCTAAT
TCGAGCTGAACTAACTCAGCCCGGTTCCTTTTTTGGGGACGACCAAATCTATAAT
GTAATTGTTACTGCACATGCCTTCGTAATAATTTTTTTTATAGTGATGCCAATTAT
GATTGGCGGATTTGGTAATTGATTAATTCCACTAATAATCGGTGCCCCAGATATG
GCTTTCCCTCGCATGAATAATATAAGCTTTTGACTACTACCCCCCTCCTTCCTCCT
CCTCCTTGCCTCCTCCACTGTTGAAGCTGGGGTGGGAACAGGCTGAACTGTGTAC
CCCCCCCTCTCAGGAAATTTAGCTCACGACGGCCCATCTGTAGACCTAGCCATCT
TCTCCCTTCACCTAGCAGGGGTATCTTCAATTTTAGGAGCAATTAATTTTATCACC
ACTATTATCAACATAAAACCTCCAACCACCTCCCTGTATAATGCACCATTATTTAT
TTGATCTCTCCTTGTCACGGCTGTTCTTTTACTTCTCTCTCTACCAGTCCTTGCTGC
GGGTATCACTATACTTCTAACAGATCGAAACCT"/>
<sequence id="seq_Aeun_Mito_Apisto_302559"
taxon="Aeun_Mito_Apisto_302559" totalcount="4"
value="CACTAATTGACCTCCCCACCCCCTCCAACATCTCTGCTTGATGAAATTTCG
GGTCTCTATTAGGACTATGTTTAATCTCCCAGATCCTAACAGGCTTATTTCTTTCC
ATGCACTACACTGCCGACATCAACACAGCTTTTTCATCCATCACCCACATCTGCC
GAGACGTAAACTACGGATGGCTAATCCGAAATTTACATGCCAACGGAGCATCCTT
TTTTTTCATTTGTATCTATTTACACATTGCACGAGGCCTCTATTACGGCTCATTTCT
CTACAAAGAAACATGAAACATCGGGGTAATCCTTCTACTATTAGTAATAATAACC
GCTTTTGTAGGCTATGTACTCCCATGAGGACAAATATCGTTTTGGGGGGCTACAG
TCATTACCAACCTACTTTCCGCTGTTCCTTACATTGGAAATTCACTAGTTCAATGA
ATCTGAGGTGGCTTCTCAGTCGACAATGCCACCCTTACACGATTCTTCGCTATCCA
TTTTCTGCTTCCATTTGTTATCGCAGCCCTAACCCTAATTCACCTAATTTTTCTTCA
CGAGACAGGATCCACCAACCCAATTGGACTAAGCCCAAACACAGACAAAATCTC
CTTCCACCCATACTTCTCGTACAAAGACCTTCTCGGCTTCTTAATCCTACTTTTAA
CTCTAATGTTTGGTGCCTGAGCTGGTATAGCAGGAACCGCACTAAGCATGCTAAT
TCGAGCTGAACTAACTCAGCCCGGTTCCTTTTTTGGGGACGACCAAATCTATAAT
GTAATTGTTACTGCACATGCCTTCGTAATAATTTTTTTTATAGTGATGCCAATTAT
GATTGGCGGATTTGGTAATTGATTAATTCCACTAATAATCGGTGCCCCAGATATG
GCTTTCCCTCGCATGAATAATATAAGCTTTTGACTACTACCCCCCTCCTTCCTCCT
CCTCCTTGCCTCCTCCACTGTTGAAGCTGGGGTGGGAACAGGCTGAACTGTGTAC
CCCCCCCTCTCAGGAAATTTAGCTCACGACGGCCCATCTGTAGACCTAGCCATCT
TCTCCCTTCACCTAGCAGGGGTATCTTCAATTTTAGGAGCAATTAATTTTATCACC
ACTATTATCAACATAAAACCTCCAACCACCTCCCTGTATAATGCACCATTATTTAT
TTGATCTCTCCTTGTCACGGCTGTTCTTTTACTTCTCTCTCTACCAGTCCTTGCTGC
GGGTATCACTATACTTCTAACAGATCgAAACCT"/>

<sequence id="seq_Aeun_Mito_Apisto_302560" taxon="Aeun_Mito_Apisto_302560" totalcount="4" value="CACTAATTGACCTCCCCACCCCCTCCAACATCTCTGCTTGATGAAATTTCG GGTCTCTATTAGGACTATGTTTAATCTCCCAGATCCTAACAGGCTTATTTCTTTCC ATGCACTACACTGCCGACATCAACACAGCTTTTCATCCATCACCCACATCTGCC GAGACGTAAACTACGGATGGCTAATCCGAAATTTACATGCCAACGGAGCATCCTT TTTTTTCATTTGTATCTATTTACACATTGCACGAGGCCTCTATTACGGCTCGTTTCT CTACAAAGAAACATGAAACATCGGGGTAATCCTTCTACTATTAGTAATAATAACC GCTTTTGTAGGCTATGTACTCCCATGAGGACAAATATCGTTTTGGGGGGCTACAG TCATTACCAACCTACTTTCCGCTGTTCCTTACATTGGAAATTCACTAGTTCAATGA ATCTGAGGTGGCTTCTCAGTCGACAATGCCACCCTTACACGATTCTTCGCTATCCA TTTTCTGCTTCCATTTGTTATCGCAGCCCTAACCCTAATTCACCTAATTTTTCTTCA CGAGACAGGATCCACCAACCCAATTGGACTAAACCCAAACACAGACAAAATCTC CTTCCACCCATACTTCTCGTACAAAGACCTTCTCGGCTTCTTAATCCTACTTTTAA CTCTAATGTTTGGTGCCTGAGCTGGTATAGCAGGAACCGCACTAAGCATGCTAAT TCGAGCTGAACTAACTCAGCCCGGTTCCTTTTTTGGGGACGACCAAATCTATAAT GTAATTGTTACTGCACATGCCTTCGTAATAATTTTTTTTATAGTGATGCCAATTAT GATTGGCGGATTTGGTAATTGATTAATTCCACTAATAATCGGTGCCCCAGATATG GCTTTCCCTCGCATGAATAATATAAGCTTTTGACTACTACCCCCCTCCTTCCTCCT CCTCCTTGCCTCCTCCACTGTTGAAGCTGGGGTGGGAACAGGCTGAACTGTGTAC CCCCCCCTCTCAGGAAATTTAGCTCACGACGGCCCATCTGTAGACCTAGCCATCT TCTCCCTTCACCTAGCAGGGGTATCTTCAATTTTAGGAGCAATTAATTTTATCACC ACTATTATCAACATAAAACCTCCAACCACCTCCCTGTATAATGCACCATTATTTAT TTGATCTCTCCTTGTCACGGCTGTTCTTTTACTTCTCTCTCTACCAGTCCTTGCTGC GGGTATCACTATACTTCTAACAGATCgAAACCT"/>
<sequence id="seq_Aeun_Mito_Apisto_302938" taxon="Aeun_Mito_Apisto_302938" totalcount="4" value="CACTAATTGACCTCCCCACCCCCTCCAACATCTCTGCTTGATGAAATTTCG GGTCTCTATTAGGACTATGTTTAATCTCCCAGATCCTAACAGGCTTATTTCTTTCC ATGCACTACACTGCCGACATCAACACAGCTTTTCATCCATCACCCACATCTGCC GAGACGTAAACTACGGATGGCTAATCCGAAATTTACATGCCAACGGAGCATCCTT TTTTTTCATTTGTATCTATTTACACATTGCACGAGGCCTCTATTACGGCTCATTTCT CTACAAAGAAACATGAAACATCGGGGTAATCCTTCTACTATTAGTAATAATAACC GCTTTTGTAGGCTATGTACTCCCATGAGGACAAATATCGTTTTGGGGGGCTACAG TCATTACCAACCTACTTTCCGCTGTTCCTTACATTGGAAATTCACTAGTTCAATGA ATCTGAGGTGGCTTCTCAGTCGACAATGCCACCCTTACACGATTCTTCGCTATCCA TTTTCTGCTTCCATTTGTTATCGCAGCCCTAACCCTAATTCACCTAATTTTTCTTCA CGAGACAGGATCCACCAACCCAATTGGACTAAGCCCAAACACAGACAAAATCTC CTTCCACCCATACTTCTCGTACAAAGACCTTCTCGGCTTCTTAATCCTACTTTTAA CTCTAATGTTTGGTGCCTGAGCTGGTATAGCAGGAACCGCACTAAGCATGCTAAT TCGAGCTGAACTAACTCAGCCCGGTTCCTTTTTTGGGGACGACCAAATCTATAAT GTAATTGTTACTGCACATGCCTTCGTAATAATTTTTTTTATAGTGATGCCAATTAT GATTGGCGGATTTGGTAATTGATTAATTCCACTAATAATCGGTGCCCCAGATATG GCTTTCCCTCGCATGAATAATATAAGCTTTTGACTACTACCCCCCTCCTTCCTCCT CCTCCTTGCCTCCTCCACTGTTGAAGCTGGGGTGGGAACAGGCTGAACTGTGTAC CCCCCCCTCTCAGGAAATTTAGCTCACGACGGCCCATCTGTAGACCTAGCCATCT TCTCCCTTCACCTAGCAGGGGTATCTTCAATTTTAGGAGCAATTAATTTTATCACC ACTATTATCAACATAAAACCTCCAACCACCTCCCTGTATAATGCACCATTATTTAT TTGATCTCTCCTTGTCACGGCTGTTCTTTTACTTCTCTCTCTACCAGTCCTTGCTGC GGGTATCACTATACTTCTAACAGATCGAAACCT"/>

<sequence id="seq_Aeun_Mito_Apisto_302939"
taxon="Aeun_Mito_Apisto_302939" totalcount="4"
value="CACTAATTGACCTCCCCACCCCCTCCAACATCTCTGCTTGATGAAATTTCG
GGTCTCTATTAGGACTATGTTTAATCTCCCAGATCCTAACAGGCTTATTTCTTTCC
ATGCACTACACTGCCGACATCAACACAGCTTTTCATCCATCACCCACATCTGCC
GAGACGTAAACTACGGATGGCTAATCCGAAATTTACATGCCAACGGAGCATCCTT
TTTTTTCATTTGTATCTATTTACACATTGCACGAGGCCTCTATTACGGCTCATTTCT
CTACAAAGAAACATGAAACATCGGGGTAATCCTTCTACTATTAGTAATAATAACC
GCTTTTGTAGGCTATGTACTCCCATGAGGACAAATATCGTTTTGGGGGGCTACAG
TCATTACCAACCTACTTTCCGCTGTTCCTTACATTGGAAATTCACTAGTTCAATGA
ATCTGAGGTGGCTTCTCAGTCGACAATGCCACCCTTACACGATTCTTCGCTATCCA
TTTTCTGCTTCCATTTGTTATCGCAGCCCTAACCCTAATTCACCTAATTTTTCTTCA
CGAGACAGGATCCACCAACCCAATTGGACTAAGCCCAAACACAGACAAAATCTC
CTTCCACCCATACTTCTCGTACAAAGACCTTCTCGGCTTCTTAATCCTACTTTTAA
CTCTAATGTTTGGTGCCTGAGCTGGTATAGCAGGAACCGCACTAAGCATGCTAAT
TCGAGCTGAACTAACTCAGCCCGGTTCCTTTTTTGGGGACGACCAAATCTATAAT
GTAATTGTTACTGCACATGCCTTCGTAATAATTTTTTTTATAGTGATGCCAATTAT
GATTGGCGGATTTGGTAATTGATTAATTCCACTAATAATCGGTGCCCCAGATATG
GCTTTCCCTCGCATGAATAATATAAGCTTTTGACTACTACCCCCCTCCTTCCTCCT
CCTCCTTGCCTCCTCCACTGTTGAAGCTGGGGTGGGAACAGGCTGAACTGTGTAC
CCCCCCCTCTCAGGAAATTTAGCTCACGACGGCCCATCTGTAGACCTAGCCATCT
TCTCCCTTCACCTAGCAGGGGTATCTTCAATTTTAGGAGCAATTAATTTTATCACC
ACTATTATCAACATAAAACCTCCAACCACCTCCCTGTATAATGCACCATTATTTAT
TTGATCTCTCCTTGTCACGGCTGTTCTTTTACTTCTCTCTCTACCAGTCCTTGCTGC
GGGTATCACTATACTTCTAACAGATCGAAACCT"/>
<sequence id="seq_Aeun_Mito_Apisto_302940"
taxon="Aeun_Mito_Apisto_302940" totalcount="4"
value="CACTAATTGACCTCCCCACCCCCTCCAACATCTCTGCTTGATGAAATTTCG
GGTCTCTATTAGGACTATGTTTAATCTCCCAGATCCTAACAGGCTTATTTCTTTCC
ATGCACTACACTGCCGACATCAACACAGCTTTTCATCCATCACCCACATCTGCC
GAGACGTAAACTACGGATGGCTAATCCGAAATTTACATGCCAACGGAGCATCCTT
TTTTTTCATTTGTATCTATTTACACATTGCACGAGGCCTCTATTACGGCTCATTTCT
CTACAAAGAAACATGAAACATCGGGGTAATCCTTCTACTATTAGTAATAATAACC
GCTTTTGTAGGCTATGTACTCCCATGAGGACAAATATCGTTTTGGGGGGCTACAG
TCATTACCAACCTACTTTCCGCTGTTCCTTACATTGGAAATTCACTAGTTCAATGA
ATCTGAGGTGGCTTCTCAGTCGACAATGCCACCCTTACACGATTCTTCGCTATCCA
TTTTCTGCTTCCATTTGTTATCGCAGCCCTAACCCTAATTCACCTAATTTTTCTTCA
CGAGACAGGATCCACCAACCCAATTGGACTAAGCCCAAACACAGACAAAATCTC
CTTCCACCCATACTTCTCGTACAAAGACCTTCTCGGCTTCTTAATCCTACTTTTAA
CTCTAATGTTTGGTGCCTGAGCTGGTATAGCAGGAACCGCACTAAGCATGCTAAT
TCGAGCTGAACTAACTCAGCCCGGTTCCTTTTTTGGGGACGACCAAATCTATAAT
GTAATTGTTACTGCACATGCCTTCGTAATAATTTTTTTTATAGTGATGCCAATTAT
GATTGGCGGATTTGGTAATTGATTAATTCCACTAATAATCGGTGCCCCAGATATG
GCTTTCCCTCGCATGAATAATATAAGCTTTTGACTACTACCCCCCTCCTTCCTCCT
CCTCCTTGCCTCCTCCACTGTTGAAGCTGGGGTGGGAACAGGCTGAACTGTGTAC
CCCCCCCTCTCAGGAAATTTAGCTCACGACGGCCCATCTGTAGACCTAGCCATCT
TCTCCCTTCACCTAGCAGGGGTATCTTCAATTTTAGGAGCAATTAATTTTATCACC
ACTATTATCAACATAAAACCTCCAACCACCTCCCTGTATAATGCACCATTATTTAT
TTGATCTCTCCTTGTCACGGCTGTTCTTTTACTTCTCTCTCTACCAGTCCTTGCTGC
GGGTATCACTATACTTCTAACAGATCgAAACCT"/>

<sequence id="seq_Aeun_Mito_Apisto_301804"
taxon="Aeun_Mito_Apisto_301804" totalcount="4"
value="CACTAATTGACCTCCCCACCCCCTCCAACATCTCTGCTTGATGAAATTTCG
GGTCTCTATTAGGACTATGTTTAATCTCCCAGATCCTAACAGGCTTATTTCTTTCC
ATGCACTACACTGCCGACATCAACACAGCTTTTTCATCCATCACCCACATCTGCC
GAGACGTAAACTACGGATGGCTAATCCGAAATTTACATGCCAACGGAGCATCCTT
TTTTTTCATTTGTATCTATTTACACATTGCACGAGGCCTCTATTACGGCTCATTTCT
CTACAAAGAAACATGAAACATCGGGGTAATCCTTCTACTATTAGTAATAATAACC
GCTTTTGTAGGCTATGTACTCCCATGAGGACAAATATCGTTTTGGGGGGCTACAG
TCATTACCAACCTACTTTCCGCTGTTCCTTACATTGGAAATTCACTAGTTCAATGA
ATCTGAGGTGGCTTCTCAGTCGACAATGCCACCCTTACACGATTCTTCGCTATCCA
TTTTCTGCTTCCATTTGTTATCGCAGCCCTAACCCTAATTCACCTAATTTTTCTTCA
CGAGACAGGATCCACCAACCCAATTGGACTAAGCCCAAACACAGACAAAATCTC
CTTCCACCCATACTTCTCGTACAAAGACCTTCTCGGCTTCTTAATCCTACTTTTAA
CTCTAATGTTTGGTGCCTGAGCTGGTATAGCAGGAACCGCACTAAGCATGCTAAT
TCGAGCTGAACTAACTCAGCCCGGTTCCTTTTTTGGGGACGACCAAATCTATAAT
GTAATTGTTACTGCACATGCCTTCGTAATAATTTTTTTTATAGTGATGCCAATTAT
GATTGGCGGATTTGGTAATTGATTAATTCCACTAATAATCGGTGCCCCAGATATG
GCTTTCCCTCGCATGAATAATATAAGCTTTTGACTACTACCCCCCTCCTTCCTCCT
CCTCCTTGCCTCCTCCACTGTTGAAGCTGGGGTGGGAACAGGCTGAACTGTGTAC
CCCCCCCTCTCAGGAAATTTAGCTCACGACGGCCCATCTGTAGACCTAGCCATCT
TCTCCCTTCACCTAGCAGGGGTATCTTCAATTTTAGGAGCAATTAATTTTATCACC
ACTATTATCAACATAAAACCTCCAACCACCTCCCTGTATAATGCACCATTATTTAT
TTGATCTCTCCTTGTCACGGCTGTTCTTTTACTTCTCTCTCTACCAGTCCTTGCTGC
GGGTATCACTATACTTCTAACAGATCGAAACCT"/>
<sequence id="seq_Ahua_Mito_Apisto_302705"
taxon="Ahua_Mito_Apisto_302705" totalcount="4"
value="CACTAATTGACCTTCCCACCCCCTCCAACATCTCTGCTTGATGAAATTTCG
GGTCTCTATTAGGACTATGCTTAATCTCCCAAGTCCTAACAGGCTTATTTCTTTCC
ATACACTACACTGCCGACATCACCACAGCTTTTTCATCCATCACTCACATCTGCCG
AGATGTAAACTATGGATGGCTAATCCGAAATTTACATGCCAACGGAGCATCCTTT
TTTTTCATTTGTATCTATTTACACATTGCACGGGGCCTCTATTACGGCTCATTTCTC
TACAAAGAAACATGAAGCATCGGGGTAATCCTTCTACTACTAGTAATAATAACCG
CTTTTGTAGGCTATGTACTCCCATGAGGACAAATATCGTTTTGAGGGGCTACAGT
CATTACCAACCTACTCTCCGCTGTTCCCTACATTGGAAATTCACTAGTTCAATGAA
TCTGAGGTGGCTTCTCAGTCGACAATGCCACCCTTACACGATTCTTCGCTATCCAT
TTTCTGCTTCCATTTGTTATCGCAGCCCTAACCCTAATTCACCTAATTTTTCTTCAT
GAAACAGGATCTACCAACCCAATTGGACTAAACCCAAACACAGACAAAATCTCC
TTCCACCCATACTTCTCTTACAAAGACCTTCTCGGTTTCTTAATCCTACTTTTAATT
TTAATGTTCGGTGCCTGAGCTGGTATAGCAGGAACCGCACTAAGCATGCTAATTC
GAGCTGAGCTAACTCAGCCCGGTTCCTTTCTTGGGGACGACCAAATCTATAATGT
AATTGTTACTGCACATGCCTTCGTAATAATTTTTTTTATAGTGATGCCAATTATGA
TTGGCGGATTTGGTAATTGATTAATTCCACTAATAATTGGTGCCCCAGATATGGCT
TTCCCTCGCATAAATAATATAAGCTTCTGACTACTACCCCCCTCCTTCCTCCTCCT
CCTTGCCTCCTCCACTGTTGAAGCTGGGGTGGGGACAGGCTGAACTGTATACCCC
CCCCTCTCAGGGAATTTAGCCCACGACGGCCCATCTGTAGACCTGGCCATCTTCT
CCCTTCACTTAGCAGGGGTATCTTCAATTTTGGGTGCAATTAATTTTATCACCACT
ATTATCAACATAAAACCTCCAACCACCTCCCTGTATAATGCACCATTATTTATTTG
ATCTCTCCTTGTCACGGCTGTTCTTCTACTTCTCTCTCTACCAGTCCTTGCTGCAGG
TATCACTATACTTCTAACAGATCGAAACCT"/>

<sequence id="seq_Ahua_Mito_Apisto_302706"
taxon="Ahua_Mito_Apisto_302706" totalcount="4"
value="CACTAATTGACCTTCCCACCCCCTCCAACATCTCTGCTTGATGAAATTTCG
GGTCTCTATTAGGACTATGCTTAATCTCCCAAGTCCTAACAGGCTTATTTCTTTCC
ATACACTACACTGCCGACATCACCACAGCTTTTCATCCATCACTCACATCTGCCG
AGATGTAAACTATGGATGGCTAATCCGAAATTTACATGCCAACGGAGCATCCTTT
TTTTTCATTTGTATCTATTTACACATTGCACGGGGCCTCTATTACGGCTCATTTCTC
TACAAAGAAACATGAAGCATCGGGGTAATCCTTCTACTACTAGTAATAATAACCG
CTTTTGTAGGCTATGTACTCCCATGAGGACAAATATCGTTTTGAGGGGCTACAGT
CATTACCAACCTACTCTCCGCTGTTCCCTACATTGGAAATTCACTAGTTCAATGAA
TCTGAGGTGGCTTCTCAGTCGACAATGCCACCCTTACACGATTCTTCGCTATCCAT
TTTCTGCTTCCATTTGTTATCGCAGCCCTAACCCTAATTCACCTAATTTTTCTTCAT
GAAACAGGATCTACCAACCCAATTGGACTAAACCCAAACACAGACAAAATCTCC
TTCCACCCATACTTCTCTTACAAAGACCTTCTCGGTTTCTTAATCCTACTTTTAATT
TTAATGTTCGGTGCCTGAGCTGGTATAGCAGGAACCGCACTAAGCATGCTAATTC
GAGCTGAGCTAACTCAGCCCGGTTCCTTTCTTGGGGACGACCAAATCTATAATGT
AATTGTTACTGCACATGCCTTCGTAATAATTTTTTTATAGTGATGCCAATTATGA
TTGGCGGATTTGGTAATTGATTAATTCCACTAATAATTGGTGCCCCAGATATGGCT
TTCCCTCGCATAAATAATATAAGCTTCTGACTACTACCCCCCTCCTTCCTCCTCCT
CCTTGCCTCCTCCACTGTTGAAGCTGGGGTGGGGACAGGCTGAACTGTATACCCC
CCCCTCTCAGGGAATTTAGCCCACGACGGCCCATCTGTAGACCTGGCCATCTTCT
CCCTTCACTTAGCAGGGGTATCTTCAATTTTGGGTGCAATTAATTTTATCACCACT
ATTATCAACATAAAACCTCCAACCACCTCCCTGTATAATGCACCATTATTTATTTG
ATCTCTCCTTGTCACGGCTGTTCTTCTACTTCTCTCTCTACCAGTCCTTGCTGCAGG
TATCACTATACTTCTAACAGATCGAAACCT"/>
<sequence id="seq_Ahua_Mito_Apisto_302707"
taxon="Ahua_Mito_Apisto_302707" totalcount="4"
value="CACTAATTGACCTTCCCACCCCCTCCAACATCTCTGCTTGATGAAATTTCG
GGTCTCTATTAGGACTATGCTTAATCTCCCAAGTCCTAACAGGCTTATTTCTTTCC
ATACACTACACTGCCGACATCACCACAGCTTTTCATCCATCACTCACATCTGCCG
AGATGTAAACTATGGATGGCTAATCCGAAATTTACATGCCAACGGAGCATCCTTT
TTTTTCATTTGTATCTATTTACACATTGCACGGGGCCTCTATTACGGCTCATTTCTC
TACAAAGAAACATGAAGCATCGGGGTAATCCTTCTACTACTAGTAATAATAACCG
CTTTTGTAGGCTATGTACTCCCATGAGGACAAATATCGTTTTGAGGGGCTACAGT
CATTACCAACCTACTCTCCGCTGTTCCCTACATTGGAAATTCACTAGTTCAATGAA
TCTGAGGTGGCTTCTCAGTCGACAATGCCACCCTTACACGATTCTTCGCTATCCAT
TTTCTGCTTCCATTTGTTATCGCAGCCCTAACCCTAATTCACCTAATTTTTCTTCAT
GAAACAGGATCTACCAACCCAATTGGACTAAACCCAAACACAGACAAAATCTCC
TTCCACCCATACTTCTCTTACAAAGACCTTCTCGGTTTCTTAATCCTACTTTTAATT
TTAATGTTCGGTGCCTGAGCTGGTATAGCAGGAACCGCACTAAGCATGCTAATTC
GAGCTGAGCTAACTCAGCCCGGTTCCTTTCTTGGGGACGACCAAATCTATAATGT
AATTGTTACTGCACATGCCTTCGTAATAATTTTTTTATAGTGATGCCAATTATGA
TTGGCGGATTTGGTAATTGATTAATTCCACTAATAATTGGTGCCCCAGATATGGCT
TTCCCTCGCATAAATAATATAAGCTTCTGACTACTACCCCCCTCCTTCCTCCTCCT
CCTTGCCTCCTCCACTGTTGAAGCTGGGGTGGGGACAGGCTGAACTGTATACCCC
CCCCTCTCAGGGAATTTAGCCCACGACGGCCCATCTGTAGACCTGGCCATCTTCT
CCCTTCACTTAGCAGGGGTATCTTCAATTTTGGGTGCAATTAATTTTATCACCACT
ATTATCAACATAAAACCTCCAACCACCTCCCTGTATAATGCACCATTATTTATTTG
ATCTCTCCTTGTCACGGCTGTTCTTCTACTTCTCTCTCTACCAGTCCTTGCTGCAGG
TATCACTATACTTCTAACAGATCGAAACCT"/>

<sequence id="seq_Ahua_Mito_Apisto_302708"
taxon="Ahua_Mito_Apisto_302708" totalcount="4"
value="CACTAATTGACCTTCCCACCCCCTCCAACATCTCTGCTTGATGAAATTTCG
GGTCTCTATTAGGACTATGCTTAATCTCCCAAGTCCTAACAGGCTTATTTCTTTCC
ATACACTACACTGCCGACATCACCACAGCTTTTTCATCCATCACTCACATCTGCCG
AGATGTAAACTATGGATGGCTAATCCGAAATTTACATGCCAACGGAGCATCCTTT
TTTTTCATTTGTATCTATTTACACATTGCACGGGGCCTCTATTACGGCTCATTTCTC
TACAAAGAAACATGAAGCATCGGGGTAATCCTTCTACTACTAGTAATAATAACCG
CTTTTGTAGGCTATGTACTCCCATGAGGACAAATATCGTTTTGAGGGGCTACAGT
CATTACCAACCTACTCTCCGCTGTTCCCTACATTGGAAATTCACTAGTTCAATGAA
TCTGAGGTGGCTTCTCAGTCGACAATGCCACCCTTACACGATTCTTCGCTATCCAT
TTTCTGCTTCCATTTGTTATCGCAGCCCTAACCCTAATTCACCTAATTTTTCTTCAT
GAAACAGGATCTACCAACCCAATTGGACTAAACCCAAACACAGACAAAATCTCC
TTCCACCCATACTTCTCTTACAAAGACCTTCTCGGTTTCTTAATCCTACTTTTAATT
TTAATGTTCGGTGCCTGAGCTGGTATAGCAGGAACCGCACTAAGCATGCTAATTC
GAGCTGAGCTAACTCAGCCCGGTTCCTTTCTTGGGGACGACCAAATCTATAATGT
AATTGTTACTGCACATGCCTTCGTAATAATTTTTTTATAGTGATGCCAATTATGA
TTGGCGGATTTGGTAATTGATTAATTCCACTAATAATTGGTGCCCCAGATATGGCT
TTCCCTCGCATAAATAATATAAGCTTCTGACTACTACCCCCCTCCTTCCTCCTCCT
CCTTGCCTCCTCCACTGTTGAAGCTGGGGTGGGGACAGGCTGAACTGTATACCCC
CCCCTCTCAGGGAATTTAGCCCACGACGGCCCATCTGTAGACCTGGCCATCTTCT
CCCTTCACTTAGCAGGGGTATCTTCAATTTTGGGTGCAATTAATTTTATCACCACT
ATTATCAACATAAAACCTCCAACCACCTCCCTGTATAATGCACCATTATTTATTTG
ATCTCTCCTTGTCACGGCTGTTCTTCTACTTCTCTCTCTACCAGTCCTTGCTGCAGG
TATCACTATACTTCTAACAGATCGAAACCT"/>
<sequence id="seq_Ahua_Mito_Apisto_302709"
taxon="Ahua_Mito_Apisto_302709" totalcount="4"
value="CACTAATTGACCTTCCCACCCCCTCCAACATCTCTGCTTGATGAAATTTCG
GGTCTCTATTAGGACTATGCTTAATCTCCCAAGTCCTAACAGGCTTATTTCTTTCC
ATACACTACACTGCCGACATCACCACAGCTTTTTCATCCATCACTCACATCTGCCG
AGATGTAAACTATGGATGGCTAATCCGAAATTTACATGCCAACGGAGCATCCTTT
TTTTTCATTTGTATCTATTTACACATTGCACGGGGCCTCTATTACGGCTCATTTCTC
TACAAAGAAACATGAAGCATCGGGGTAATCCTTCTACTACTAGTAATAATAACCG
CTTTTGTAGGCTATGTACTCCCATGAGGACAAATATCGTTTTGAGGGGCTACAGT
CATTACCAACCTACTCTCCGCTGTTCCCTACATTGGAAATTCACTAGTTCAATGAA
TCTGAGGTGGCTTCTCAGTCGACAATGCCACCCTTACACGATTCTTCGCTATCCAT
TTTCTGCTTCCATTTGTTATCGCAGCCCTAACCCTAATTCACCTAATTTTTCTTCAT
GAAACAGGATCTACCAACCCAATTGGACTAAACCCAAACACAGACAAAATCTCC
TTCCACCCATACTTCTCTTACAAAGACCTTCTCGGTTTCTTAATCCTACTTTTAATT
TTAATGTTCGGTGCCTGAGCTGGTATAGCAGGAACCGCACTAAGCATGCTAATTC
GAGCTGAGCTAACTCAGCCCGGTTCCTTTCTTGGGGACGACCAAATCTATAATGT
AATTGTTACTGCACATGCCTTCGTAATAATTTTTTTATAGTGATGCCAATTATGA
TTGGCGGATTTGGTAATTGATTAATTCCACTAATAATTGGTGCCCCAGATATGGCT
TTCCCTCGCATAAATAATATAAGCTTCTGACTACTACCCCCCTCCTTCCTCCTCCT
CCTTGCCTCCTCCACTGTTGAAGCTGGGGTGGGGACAGGCTGAACTGTATACCCC
CCCCTCTCAGGGAATTTAGCCCACGACGGCCCATCTGTAGACCTGGCCATCTTCT
CCCTTCACTTAGCAGGGGTATCTTCAATTTTGGGTGCAATTAATTTTATCACCACT
ATTATCAACATAAAACCTCCAACCACCTCCCTGTATAATGCACCATTATTTATTTG
ATCTCTCCTTGTCACGGCTGTTCTTCTACTTCTCTCTCTACCAGTCCTTGCTGCAGG
TATCACTATACTTCTAACAGATCGAAACCT"/>

<sequence id="seq_Ajur_Mito_Apisto_302878"
taxon="Ajur_Mito_Apisto_302878" totalcount="4"
value="CGCTAGTCGACCTTCCCACCCCCTCTAACATCTCCGCCTGATGAAATCTTG
GATCCCTTCTAGGCCTCTGCCTAGTCTCTCAAATCTTAACAGGCCTATTCCTTTCA
ATACATTATACTGCCGATACCACAACAGCTTTTTCATCCGTCGCTCATATTTGCCG
AGATGTAAACTACGGATGACTAATCCGCAATCTACATGCTAACGGAGCATCCTTC
TTCTTCATCTGCATTTACCTACACATCGGACGGGGCCTCTACTTCGGCTCTTACCT
TTATAAACAAACATGGTACGTCGGGGTGGTACTCCTACTGCTAGTTATAATAACC
GCTTTCGTGGGCTACGTCCTACCATGAGGACAAATATCGTTCTGAGGGGCCACCG
TTATCACCAACCTTCTGTCAGCAGCCCCTTACATCGGGGACTCCATAGTCCAATG
AATTTGAGGCGGCTTCTCAGTCGACAACCCCACCCTAACCCGATTCTTTGCCATCC
ACTTCCTGCTTCCCTTCACCATCGCAGCCCTCACCTTAATCCACCTAATTTTTCTCC
ACGAAACAGGCTCCACAAACCCCATTGGGCTAAACCCAAACTCAGATAAAATTT
CTTTCCACCCATTTTTCTCCCTCAAAGACCTCTTAGGATTCCTAGTTCTACTCACA
ACCCTAATATTTGGGGCTTGAGCTGGACTAGTAGGTACCGCATTAAGCACGCTAA
TTCGAGCAGAGCTCACCCAACCCGGCTCCTTTTTCGGAAACGACCAGCTTTATAA
TGTAGTCGTGACTGCACACGCCTTCGTAATAATCTTTTTCATGGTGATACCAATCA
TAATCGGAGGATTTGGCAATTGACTAATCCCGCTGATACTTGGCGCTCCAGACAT
GGCCTTCCCCCGTATGAACAATATGAGCTTCTGACTGCTCCCCCCATCTTTCCTCC
TACTCTTTGTCTCCTCCGCTATCGAAGCTGGCGTCGGGACGGGCTGAACTGTCTAC
CCCCCCCTCTCCGGAAATTTGGCCCATGACGGACCATCCGTAGACCTGGCTATTTT
TTCCCTCCACTTAGCAGGGGTATCCTCAATTTTAGGCGCAATTAACTTTATTACCA
CCATCGTTAACATGAAACCCCCAGCCACCTCTATGTACAATGCGCCTTTGTTTATC
TGATCCCTTCTCATCACCGCTGTGCTGCTGCTTCTCTCCTTGCCAGTACTTGCTGCT
GGTATCACCATACTTCTAACAGACCGaAACCT"/>
<sequence id="seq_Ajur_Mito_Apisto_302879"
taxon="Ajur_Mito_Apisto_302879" totalcount="4"
value="CGCTAGTCGACCTTCCCACCCCCTCTAACATCTCCGCCTGATGAAATCTTG
GATCCCTTCTAGGCCTCTGCCTAATCTCTCAAATCTTAACAGGCCTATTCCTTTCA
ATACATTATACTGCCGATACCACAACAGCTTTTTCATCCGTCGCTCATATTTGCCG
AGATGTAAACTACGGATGACTAATCCGCAATCTACATGCTAACGGAGCATCCTTC
TTCTTCATCTGCATTTACCTACACATCGGACGGGGCCTCTACTTCGGCTCTTACCT
TTATAAACAAACATGGTACGTCGGGGTGGTACTCCTACTGCTAGTTATAATAACC
GCTTTCGTGGGCTACGTCCTACCATGAGGACAAATATCGTTCTGAGGGGCCACCG
TTATCACCAACCTTCTGTCAGCAGCCCCTTACATCGGGGACTCCATAGTCCAATG
AATTTGAGGCGGCTTCTCAGTCGACAACCCCACCCTAACCCGATTCTTTGCCATCC
ACTTCCTGCTTCCCTTCACCATCGCAGCCCTCACCTTAATCCACCTAATTTTTCTCC
ACGAAACAGGCTCCACAAACCCCATTGGGCTAAACCCAAACTCAGATAAAATTT
CTTTCCACCCATTTTTCTCCCTCAAAGACCTCTTAGGATTCCTAGTTCTACTCACA
ACCCTAATATTTGGGGCTTGAGCTGGACTAGTAGGTACCGCATTAAGCACGCTAA
TTCGAGCAGAGCTCACCCAACCCGGCTCCTTTTTCGGAAACGACCAGCTTTATAA
TGTAGTCGTGACTGCACACGCCTTCGTAATAATCTTTTTCATGGTGATACCAATCA
TAATCGGAGGATTTGGCAATTGACTAATCCCGCTGATACTTGGCGCTCCAGACAT
GGCCTTCCCCCGTATGAACAATATGAGCTTCTGACTGCTCCCCCCATCTTTCCTCC
TACTCTTTGTCTCCTCCGCTATCGAAGCTGGCGTCGGGACGGGCTGAACTGTCTAC
CCCCCCCTCTCCGGAAATTTGGCCCATGACGGACCATCCGTAGACCTGGCTATTTT
TTCCCTCCACTTAGCAGGGGTATCCTCAATTTTAGGCGCAATTAACTTTATTACCA
CCATCGTTAACATGAAACCCCCAGCCACCTCTATGTACAATGCGCCTTTGTTTATC
TGATCCCTTCTCATCACCGCTGTGCTGCTGCTTCTCTCCTTGCCAGTACTTGCTGCT
GGTATCACCATACTTCTAACAGACCGaAACCT"/>

<sequence id="seq_Ajur_Mito_Apisto_302880”
taxon="Ajur_Mito_Apisto_302880” totalcount="4"
value="CGCTAGTCGACCTTCCCACCCCCTCTAACATCTCCGCCTGATGAAATCTTG
GATCCCTTCTAGGCCTCTGCCTAGTCTCTCAAATCTTAACAGGCCTATTCCTTTCA
ATACATTATACTGCCGATACCACAACAGCTTTTTCATCCGTCGCTCATATTTGCCG
AGATGTAAACTACGGATGACTAATCCGCAATCTACATGCTAACGGAGCATCCTTC
TTCTTCATCTGCATTTACCTACACATCGGACGGGGCCTCTACTTCGGCTCTTACCT
TTATAAACAAACATGGTACGTCGGGGTGGTACTCCTACTGCTAGTTATAATAACC
GCTTTCGTGGGCTACGTCCTACCATGAGGACAAATATCGTTCTGAGGGGCCACCG
TTATCACCAACCTTCTGTCAGCAGCCCCTTACATCGGGGACTCCATAGTCCAATG
AATTTGAGGCGGCTTCTCAGTCGACAACCCCACCCTAACCCGATTCTTTGCCATCC
ACTTCCTGCTTCCCTTCACCATCGCAGCCCTCACCTTAATCCACCTAATTTTTCTCC
ACGAAACAGGCTCCACAAACCCCATTGGGCTAAACCCAAACTCAGATAAAATTT
CTTTCCACCCATTTTTCTCCCTCAAAGACCTCTTAGGATTCCTAGTTCTACTCACA
ACCCTAATATTTGGGGCTTGAGCTGGAcTAGtAGGTACCGCATTAAGCACGCTAAT
TCGAGCAGAGCTCACcCAACCCGGCTCCTTTTTcGGAAACGACCAgCTTTATAATG
TAGTcGTGACTGCACACGCCTTCGTAATAATcTTTTTCATGGTGATACCAATCATA
ATCGGAGGATTTGGCAATTGAcTAATcCCGCTGATAcTTGGCGCTCCAGACATGGC
CTTCCCCCGTATGAACAATATGAGCTTcTGACTGCTCCCCCCATCTTTCCTCCTACT
CTTTGTCTCCTCCGCTATCGAAGCTGGcGTcGGGACGGGCTGAACTGTCTACCCCC
CCCTCTCCGGAAATTTGGCCCATGACGGACCATCCGTAGACCTGGCtATTTTTTCC
CTcCACTTAGCAGGGGTATCcTCAATTTTAGGcGCAATTAAcTTTATtACCACcATcgT
TAACATGAAACCcCCAGCCACCTCTATGTACAATGCGCCtTTGTTTATcTGATCCCT
TCTCATCACcGCTGTgCTGCTgCTTCTCTCCTTGCCAGTACTTGCTGCTGGTATCAC
CATACTTCTAACAGACCGAAACCT"/>
<sequence id="seq_Ameg_Mito_Apisto_302869"
taxon="Ameg_Mito_Apisto_302869" totalcount="4"
value="CCCTGATTGATCTTCCTACCCCCTCCAACATTTCCTCCTGATGAAATTTTGG
ATCCCTACTAGGCCTCTGCTTAATTTCCCAAATTCTTACAGGCCTATTTCTTTCCAT
ACACTACTCTGCTGACATCAACACAGCTTTTTCATCCGTCGCCCACATCTCTCGAG
ATGTAAACTACGGATGGCTAATCCGCAATTTACATGCTAACGGAGCATCTTTTTTT
TTTATTTGCATCTACCTTCACATCGGACGGGGCCTTTACTTCGGGTCTTATCTCTA
CAAAGAGACATGAAACATTGGAGTAGTACTTCTACTTCTAGTTATAATAACCGCT
TTTGTGGGCTACGTCCTCCCTTGGGGCCAAATATCGTTTTGAGGAGCTACCGTCAT
CACTAACCTACTGTCAGCGATCCCCTACATTGGAAATTCCCTAGTCCAATGAATTT
GAGGCGGCTTCTCAGTTGACAACGCTACCCTAACCCGATTCTTTGCCATCCACTTC
CTACTTCCATTCATCATCGCAGCCATAACCCTAATACACCTAATTTTTCTCCACGA
AACAGGCTCCACCAACCCAATCGGACTGACCCCCAACACGGATAAAATTTCTTTT
CACCCATTTTTCTCCCTCAAAGACCTCCTCGGATTCCTAATTTTACTTATAACTCT
GATATTTGGTGCTTGAGCTGGAATAGCAGGTACCGCATTAAGCATGCTAATTCGA
GCAGAACTTACCCAGCCCGGCTCCTTTTTTGGGGACGATCAGGTATATAATGTAA
TCGTAACTGCGCACGCTTTCGTAATAATCTTCTTTATAGTAATACCAATCATAATT
GGTGGATTTGGTAACTGACTAATTCCACTAATAATCGGCGCCCCAGACATGGCTT
TCCCTCGTATGAACAACATAAGCTTTTGACTGCTCCCCCCATCTTTCCTCCTCCTC
CTCGCTTCCTCAACTGTTGAAGCCGGGGTAGGGACAGGCTGGACTGTGTACCCGC
CTCTTTCCGGGAATTTAGCTCACGATGGCCCATCCGTGGACCTGGCCATCTTTTCC
CTCCACTTAGCGGGGGTATCCTCAATTTTAGGTGCAATCAACTTTATTACTACTAT
CATTAACATGAAACCTCCAGCCACCTCTTTATACAGTACCCCCCTATTCATCTGAT
CCCTCCTCGTCACTGCTGTACTTCTACTTCTTTCACTACCAGTACTTGCTGCTGGTA
TCACTATACTTCTAACGGACCGaAACCT"/>

<sequence id="seq_Ameg_Mito_Apisto_302870"
taxon="Ameg_Mito_Apisto_302870" totalcount="4"
value="CCCTGATTGATCTTCCCACCCCCTCCAACATTTCCTCCTGATGAAATTTTGG
GTCCCTACTAGGCCTCTGCTTAATTTCCCAGATTCTTACAGGCCTATTTCTTTCCAT
ACACTACTCTGCTGACATCAACACAGCTTTTTCATCCGTCGCCCACATCTCTCGAG
ATGTAAACTACGGATGGCTGATCCGCAATTTACATGCTAACGGAGCATCCTTTTT
TTTCATTTGCATCTATCTTCACATCGGACGGGGCCTTTACTTCGGGTCTTATCTCT
ACAAAGAGACATGAAACATTGGAGTAGTACTTCTACTTCTAGTTATAATAACCGC
TTTTGTGGGCTACGTCCTCCCTTGAGGCCAAATATCGTTTTGAGGAGCTACCGTCA
TCACTAACCTACTGTCAGCGATCCCCTACATTGGAAATTCCCTAGTCCAATGAATT
TGAGGCGGCTTCTCAGTTGACAACGCTACCCTAACCCGATTCTTTGCCATCCACTT
CCTACTTCCATTCATCATCGCAGCCATAACCCTAATACACCTAATTTTTCTCCACG
AAACAGGCTCCACCAACCCTATCGGGCTAACCTCCAACACGGATAAAATTTCTTT
CCACCCATTTTTCTCCCTCAAAGACCTCCTCGGATTCCTAATTTTACTTATAACTCT
GATATTTGGTGCTTGAGCTGGAATAGCAGGTACCGCATTAAGCATGCTAATTCGA
GCAGAACTTACCCAGCCCGGCTCCTTTTTTGGGGACGATCAGGTATATAATGTAA
TCGTAACTGCACACGCTTTCGTAATAATCTTCTTTATAGTAATACCAATCATAATT
GGTGGATTTGGTAACTGACTAATTCCGCTAATAATCGGCGCCCCAGACATGGCTT
TCCCTCGTATGAACAACATAAGCTTTTGACTGCTCCCCCCATCTTTCCTCCTCCTC
CTCGCTTCCTCAACTGTTGAAGCCGGGGTAGGAACAGGCTGAACTGTATACCCGC
CTCTTTCCGGGAATTTAGCTCACGACGGCCCATCCGTGGACCTGGCCATCTTTTCC
CTCCACTTAGCGGGAGTATCCTCAATTTTAGGTGCAATCAACTTTATTACTACTAT
CATTAACATGAAACCTCCAGCCACCTCTTTATACAGTACCCCCCTATTTATCTGAT
CCCTCCTCGTCACTGCTGTACTTCTACTTCTTTCACTACCAGTACTTGCTGCTGGTA
TCACTATACTTCTAACGGACCGAAACCT"/>
<sequence id="seq_Ameg_Mito_Apisto_302871"
taxon="Ameg_Mito_Apisto_302871" totalcount="4"
value="CCCTGATTGATCTTCCCACCCCCTCCAACATTTCCTCCTGATGAAATTTTGG
GTCCCTACTAGGCCTCTGCTTAATTTCCCAGATTCTTACAGGCCTATTTCTTTCCAT
ACACTACTCTGCTGACATCAACACAGCTTTTTCATCCGTCGCCCACATCTCTCGAG
ATGTAAACTACGGATGGCTGATCCGCAATTTACATGCTAACGGAGCATCCTTTTT
TTTCATTTGCATCTATCTTCACATCGGACGGGGCCTTTACTTCGGGTCTTATCTCT
ACAAAGAGACATGAAACATTGGAGTAGTACTTCTACTTCTAGTTATAATAACCGC
TTTTGTGGGCTACGTCCTCCCTTGAGGCCAAATATCGTTTTGAGGAGCTACCGTCA
TCACTAACCTACTGTCAGCGATCCCCTACATTGGAAATTCCCTAGTCCAATGAATT
TGAGGCGGCTTCTCAGTTGACAACGCTACCCTAACCCGATTCTTTGCCATCCACTT
CCTACTTCCATTCATCATCGCAGCCATAACCCTAATACACCTAATTTTTCTCCACG
AAACAGGCTCCACCAACCCTATCGGGCTAACCTCCAACACGGATAAAATTTCTTT
CCACCCATTTTTCTCCCTCAAAGACCTCCTCGGATTCCTAATTTTACTTATAACTCT
GATATTTGGTGCTTGAGCTGGAATAGCAGGTACCGCATTAAGCATGCTAATTCGA
GCAGAACTTACCCAGCCCGGCTCCTTTTTTGGGGACGATCAGGTATATAATGTAA
TCGTAACTGCACACGCTTTCGTAATAATCTTCTTTATAGTAATACCAATCATAATT
GGTGGATTTGGTAACTGACTAATTCCGCTAATAATCGGCGCCCCAGACATGGCTT
TCCCTCGTATGAACAACATAAGCTTTTGACTGCTCCCCCCATCTTTCCTCCTCCTC
CTCGCTTCCTCAACTGTTGAAGCCGGGGTAGGAACAGGCTGAACTGTATACCCGC
CTCTTTCCGGGAATTTAGCTCACGACGGCCCATCCGTGGACCTGGCCATCTTTTCC
CTCCACTTAGCGGGAGTATCCTCAATTTTAGGTGCAATCAACTTTATTACTACTAT
CATTAACATGAAACCTCCAGCCACCTCTTTATACAGTACCCCCCTATTTATCTGAT
CCCTCCTCGTCACTGCTGTACTTCTACTTCTTTCACTACCAGTACTTGCTGCTGGTA
TCACTATACTTCTAACGGACCGAAACCT"/>

<sequence id="seq_Ameg_Mito_Apisto_302872"
taxon="Ameg_Mito_Apisto_302872" totalcount="4"
value="CCCTGATTGATCTTCCTACCCCCTCCAACATTTCCTCCTGATGAAATTTTGG
ATCCCTACTAGGCCTCTGCTTAATTTCCCAAATTCTTACAGGCCTATTTCTTTCCAT
ACACTACTCTGCTGACATCAACACAGCTTTTTCATCCGTCGCCCACATCTCTCGAG
ATGTAAACTACGGATGGCTAATCCGCAATTTACATGCTAACGGAGCATCTTTTTTT
TTTATTTGCATCTACCTTCACATCGGACGGGGCCTTTACTTCGGGTCTTATCTCTA
CAAAGAGACATGAAACATTGGAGTAGTACTTCTACTTCTAGTTATAATAACCGCT
TTTGTGGGCTACGTCCTCCCTTGGGGCCAAATATCGTTTTGAGGAGCTACCGTCAT
CACTAACCTACTGTCAGCGATCCCCTACATTGGAAATTCCCTAGTCCAATGAATTT
GAGGCGGCTTCTCAGTTGACAACGCTACCCTAACCCGATTCTTTGCCATCCACTTC
CTACTTCCATTCATCATCGCAGCCATAACCCTAATACACCTAATTTTTCTCCACGA
AACAGGCTCCACCAACCCAATCGGACTGACCCCCAACACGGATAAAATTTCTTTT
CACCCATTTTTCTCCCTCAAAGACCTCCTCGGATTCCTAATTTTACTTATAACTCT
GATATTTGGTGCTTGAGCTGGAATAGCAGGTACCGCATTAAGCATGCTAATTCGA
GCAGAACTTACCCAGCCCGGCTCCTTTTTTGGGGACGATCAGGTATATAATGTAA
TCGTAACTGCGCACGCTTTCGTAATAATCTTCTTTATAGTAATACCAATCATAATT
GGTGGATTTGGTAACTGACTAATTCCACTAATAATCGGCGCCCCAGACATGGCTT
TCCCTCGTATGAACAACATAAGCTTTTGACTGCTCCCCCCATCTTTCCTCCTCCTC
CTCGCTTCCTCAACTGTTGAAGCCGGGGTAGGGACAGGCTGGACTGTGTACCCGC
CTCTTTCCGGGAATTTAGCTCACGATGGCCCATCCGTGGACCTGGCCATCTTTTCC
CTCCACTTAGCGGGGGTATCCTCAATTTTAGGTGCAATCAACTTTATTACTACTAT
CATTAACATGAAACCTCCAGCCACCTCTTTATACAGTACCCCCCTATTCATCTGAT
CCCTCCTCGTCACTGCTGTACTTCTACTTCTTTCACTACCAGTACTTGCTGCTGGTA
TCACTATACTTCTAACGGACCGAAACCT"/>
<sequence id="seq_Ameg_Mito_Apisto_302873"
taxon="Ameg_Mito_Apisto_302873" totalcount="4"
value="CCCTGATTGATCTTCCTACCCCCTCCAACATTTCCTCCTGATGAAATTTTGG
ATCCCTACTAGGCCTCTGCTTAATTTCCCAAATTCTTACAGGCCTATTTCTTTCCAT
ACACTACTCTGCTGACATCAACACAGCTTTTTCATCCGTCGCCCACATCTCTCGAG
ATGTAAACTACGGATGGCTAATCCGCAATTTACATGCTAACGGAGCATCTTTTTTT
TTTATTTGCATCTACCTTCACATCGGACGGGGCCTTTACTTCGGGTCTTATCTCTA
CAAAGAGACATGAAACATTGGAGTAGTACTTCTACTTCTAGTTATAATAACCGCT
TTTGTGGGCTACGTCCTCCCTTGGGGCCAAATATCGTTTTGAGGAGCTACCGTCAT
CACTAACCTACTGTCAGCGATCCCCTACATTGGAAATTCCCTAGTCCAATGAATTT
GAGGCGGCTTCTCAGTTGACAACGCTACCCTAACCCGATTCTTTGCCATCCACTTC
CTACTTCCATTCATCATCGCAGCCATAACCCTAATACACCTAATTTTTCTCCACGA
AACAGGCTCCACCAACCCAATCGGACTGACCCCCAACACGGATAAAATTTCTTTT
CACCCATTTTTCTCCCTCAAAGACCTCCTCGGATTCCTAATTTTACTTATAACTCT
GATATTTGGTGCTTGAGCTGGAATAGCAGGTACCGCATTAAGCATGCTAATTCGA
GCAGAACTTACCCAGCCCGGCTCCTTTTTTGGGGACGATCAGGTATATAATGTAA
TCGTAACTGCGCACGCTTTCGTAATAATCTTCTTTATAGTAATACCAATCATAATT
GGTGGATTTGGTAACTGACTAATTCCACTAATAATCGGCGCCCCAGACATGGCTT
TCCCTCGTATGAACAACATAAGCTTTTGACTGCTCCCCCCATCTTTCCTCCTCCTC
CTCGCTTCCTCAACTGTTGAAGCCGGGGTAGGGACAGGCTGGACTGTGTACCCGC
CTCTTTCCGGGAATTTAGCTCACGATGGCCCATCCGTGGACCTGGCCATCTTTTCC
CTCCACTTAGCGGGGGTATCCTCAATTTTAGGTGCAATCAACTTTATTACTACTAT
CATTAACATGAAACCTCCAGCCACCTCTTTATACAGTACCCCCCTATTCATCTGAT
CCCTCCTCGTCACTGCTGTACTTCTACTTCTTTCACTACCAGTACTTGCTGCTGGTA
TCACTATACTTCTAACGGACCGAAACCT"/>

<sequence id="seq_Amel_Mito_Apisto_301639”
taxon="Amel_Mito_Apisto_301639” totalcount="4"
value="CACTAATTGACCTCCCCACCCCCTCCAACATCTCTGCTTGATGAAATTTCG
GATCTCTATTAGGACTATGTTTAATCTCCCAGATCCTAACAGGCTTATTTCTTTCC
ATACACTACACTGCCGATATCAGCACAGCTTTTTCATCCATCACCCACATCTGCCG
AGACGTAAACTACGGATGGCTAATTCGAAATTTACATGCCAACGGAGCATCCTTT
TTTTTCATTTGTATCTATTTACACATTGCACGAGGCCTCTATTACGGCTCATTTCTC
TACAAAGAAACATGAAACATCGGAGTAATCCTTCTACTATTAGTAATAATAACCG
CTTTTGTAGGCTATGTACTCCCATGAGGACAAATATCGTTTTGAGGGGCTACAGT
CATTACCAACCTACTTTCCGCTGTTCCTTACATTGGAAACTCACTAGTTCAATGAA
TCTGAGGTGGCTTCTCAATCGACAATGCCACCCTCACACGATTCTTCGCTATCCAT
TTTCTGCTTCCATTTGTTATCGCAGCCCTAACCCTAATTCACCTAATTTTTCTTCAT
GAGACAGGATCCACCAACCCAATTGGACTAAGCCCAAACACAGACAAAATCCCC
TTCCACCCATACTTCTCGTACAAAGACCTTCTCGGCTTCTTAATCCTACTTTTAAC
TTTAATGTTTGGTGCCTGAACTGGTATAGCAGGAACCGCACTAAGCATGCTAATT
CGAGCTGAACTAACTCAGCCCGGTTCCTTTTTTGGGGACGACCAAATCTATAATG
TAATTGTTACTGCACATGCCTTCGTAATAATTTTTTTTATAGTGATGCCAATTATG
ATCGGCGGATTTGGTAATTGATTAATTCCACTAATAATTGGTGCCCCAGATATGG
CTTTCCCTCGCATGAATAATATAAGCTTTTGACTACTACCCCCCTCCTTCCTCCTC
CTCCTTGCCTCCTCCACTGTTGAAGCTGGGGTAGGAACAGGTTGAACTGTGTACC
CCCCCCTTTCAGGAAATTTAGCTCACGACGGCCCATCTGTAGACCTAGCCATCTTC
TCCCTTCACCTAGCAGGGGTATCTTCAATTTTGGGGGCAATTAATTTTATCACCAC
TATTATCAACATAAAACCTCCAACCACCTCCCTGTATAATGCACCATTATTTATTT
GATCTCTCCTTGTCACGGCTGTTCTTTTACTTCTCTCTCTACCAGTCCTTGCTGCAG
GTATCACCATACTTCTAACAGACCGAAACCT"/>
<sequence id="seq_Amel_Mito_Apisto_301640"
taxon="Amel_Mito_Apisto_301640" totalcount="4"
value="CACTAATTGACCTCCCCACCCCCTCCAACATCTCTGCTTGATGAAATTTCG
GATCTCTATTAGGACTATGTTTAATCTCCCAGATCCTAACAGGCTTATTTCTTTCC
ATACACTACACTGCCGATATCAGCACAGCTTTTTCATCCATCACCCACATCTGCCG
AGACGTAAACTACGGATGGCTAATTCGAAATTTACATGCCAACGGAGCATCCTTT
TTTTTCATTTGTATCTATTTACACATTGCACGAGGCCTTTATTACGGCTCATTTCTC
TACAAAGAAACATGAAACATCGGAGTAATCCTTCTACTATTAGTAATAATAACCG
CTTTTGTAGGCTATGTACTCCCATGAGGACAAATATCGTTTTGAGGGGCTACAGT
CATTACCAACCTACTTTCCGCTGTTCCTTACATTGGAAACTCACTAGTTCAATGAA
TCTGAGGTGGCTTCTCAATCGACAATGCCACCCTCACACGATTCTTCGCTATCCAT
TTTCTGCTTCCATTTGTTATCGCAGCCCTAACCCTAATTCACCTAATTTTTCTTCAT
GAGACAGGATCCACCAACCCAATTGGACTAAGCCCAAACACAGACAAAATCCCC
TTCCACCCATACTTCTCGTACAAAGACCTTCTCGGCTTCTTAATCCTACTTTTAAC
TTTAATGTTTGGTGCCTGAACTGGTATAGCAGGAACCGCACTAAGCATGCTAATT
CGAGCTGAACTAACTCAGCCCGGTTCCTTTTTTGGGGACGACCAAATCTATAATG
TAATTGTTACTGCACATGCCTTCGTAATAATTTTTTTTATAGTGATGCCAATTATG
ATTGGCGGATTTGGTAATTGATTAATTCCACTAATAATTGGTGCCCCAGATATGG
CTTTCCCTCGCATGAATAATATAAGCTTTTGACTACTACCCCCCTCCTTCCTCCTC
CTCCTTGCCTCCTCCACTGTTGAAGCTGGAGTAGGAACAGGTTGAACTGTGTACC
CCCCCCTTTCAGGAAATTTAGCTCACGACGGCCCATCTGTAGACCTAGCCATCTTC
TCCCTTCACCTAGCAGGGGTATCTTCAATTTTGGGGGCAATTAATTTTATCACCAC
TATTATCAACATAAAACCTCCAACCACCTCCCTGTATAATGCACCATTATTTATTT
GATCTCTCCTTGTCACGGCTGTTCTTTTACTTCTCTCTCTACCAGTCCTTGCTGCGG
GTATCACCATACTTCTAACAGACCGAAACCT"/>

<sequence id="seq_Amel_Mito_Apisto_301641"
taxon="Amel_Mito_Apisto_301641" totalcount="4"
value="CACTAATTGACCTCCCCACCCCCTCCAACATCTCTGCTTGATGAAATTTCG
GATCTCTATTAGGACTATGTTTAATCTCCCAGATCCTAACAGGCTTATTTCTTTCC
ATACACTACACTGCCGATATCAGCACAGCTTTTTCATCCATCACCCACATCTGCCG
AGACGTAAACTACGGATGGCTAATTCGAAATTTACATGCCAACGGAGCATCCTTT
TTTTTCATTTGTATCTATTTACACATTGCACGAGGCCTTTATTACGGCTCATTTCTC
TACAAAGAAACATGAAACATCGGAGTAATCCTTCTACTATTAGTAATAATAACCG
CTTTTGTAGGCTATGTACTCCCATGAGGACAAATATCGTTTTGAGGGGCTACAGT
CATTACCAACCTACTTTCCGCTGTTCCTTACATTGGAAACTCACTAGTTCAATGAA
TCTGAGGTGGCTTCTCAATCGACAATGCCACCCTCACACGATTCTTCGCTATCCAT
TTTCTGCTTCCATTTGTTATCGCAGCCCTAACCCTAATTCACCTAATTTTTCTTCAT
GAGACAGGATCCACCAACCCAATTGGACTAAGCCCAAACACAGACAAAATCCCC
TTCCACCCATACTTCTCGTACAAAGACCTTCTCGGCTTCTTAATCCTACTTTTAAC
TTTAATGTTTGGTGCCTGAACTGGTATAGCAGGAACCGCACTAAGCATGCTAATT
CGAGCTGAACTAACTCAGCCCGGTTCCTTTTTTGGGGACGACCAAATCTATAATG
TAATTGTTACTGCACATGCCTTCGTAATAATTTTTTTATAGTGATGCCAATTATG
ATTGGCGGATTTGGTAATTGATTAATTCCACTAATAATTGGTGCCCCAGATATGG
CTTTCCCTCGCATGAATAATATAAGCTTTTGACTACTACCCCCCTCCTTCCTCCTC
CTCCTTGCCTCCTCCACTGTTGAAGCTGGAGTAGGAACAGGTTGAACTGTGTACC
CCCCCCTTTCAGGAAATTTAGCTCACGACGGCCCATCTGTAGACCTAGCCATCTTC
TCCCTTCACCTAGCAGGGGTATCTTCAATTTTGGGGGCAATTAATTTTATCACCAC
TATTATCAACATAAAACCTCCAACCACCTCCCTGTATAATGCACCATTATTTATTT
GATCTCTCCTTGTCACGGCTGTTCTTTTACTTCTCTCTCTACCAGTCCTTGCTGCGG
GTATCACCATACTTCTAACAGACCGAAACCT"/>
<sequence id="seq_Amel_Mito_Apisto_301649”
taxon="Amel_Mito_Apisto_301649” totalcount="4"
value="CACTAATTGACCTCCCCACCCCCTCCAACATCTCTGCTTGATGAAATTTCG
GATCTCTATTAGGACTATGTTTAATCTCCCAGATCCTAACAGGCTTATTTCTTTCC
ATACACTACACTGCCGATATCAGCACAGCTTTTTCATCCATCACCCACATCTGCCG
AGACGTAAACTACGGATGGCTAATTCGAAATTTACATGCCAACGGAGCATCCTTT
TTTTTCATTTGTATCTATTTACACATTGCACGAGGCCTTTATTACGGCTCATTTCTC
TACAAAGAAACATGAAACATCGGAGTAATCCTTCTACTATTAGTAATAATAACCG
CTTTTGTAGGCTATGTACTCCCATGAGGACAAATATCGTTTTGAGGGGCTACAGT
CATTACCAACCTACTTTCCGCTGTTCCTTACATTGGAAACTCACTAGTTCAATGAA
TCTGAGGTGGCTTCTCAATCGACAATGCCACCCTCACACGATTCTTCGCTATCCAT
TTTCTGCTTCCATTTGTTATCGCAGCCCTAACCCTAATTCACCTAATTTTTCTTCAT
GAGACAGGATCCACCAACCCAATTGGACTAAGCCCAAACACAGACAAAATCCCC
TTCCACCCATACTTCTCGTACAAAGACCTTCTCGGCTTCTTAATCCTACTTTTAAC
TTTAATGTTTGGTGCCTGAACTGGTATAGCAGGAACCGCACTAAGCATGCTAATT
CGAGCTGAACTAACTCAGCCCGGTTCCTTTTTTGGGGACGACCAAATCTATAATG
TAATTGTTACTGCACATGCCTTCGTAATAATTTTTTTATAGTGATGCCAATTATG
ATTGGCGGATTTGGTAATTGATTAATTCCACTAATAATTGGTGCCCCAGATATGG
CTTTCCCTCGCATGAATAATATAAGCTTTTGACTACTACCCCCCTCCTTCCTCCTC
CTCCTTGCCTCCTCCACTGTTGAAGCTGGAGTAGGAACAGGTTGAACTGTGTACC
CCCCCCTTTCAGGAAATTTAGCTCACGACGGCCCATCTGTAGACCTAGCCATCTTC
TCCCTTCACCTAGCAGGGGTATCTTCAATTTTGGGGGCAATTAATTTTATCACCAC
TATTATCAACATAAAACCTCCAACCACCTCCCTGTATAATGCACCATTATTTATTT
GATCTCTCCTTGTCACGGCTGTTCTTTTACTTCTCTCTCTACCAGTCCTTGCTGCGG
GTATCACCATACTTCTAACAGACCGAAACCT"/>

<sequence id="seq_Amel_Mito_Apisto_301650"
taxon="Amel_Mito_Apisto_301650" totalcount="4"
value="CACTAATTGACCTCCCCACCCCCTCCAACATCTCTGCTTGATGAAATTTCG
GATCTCTATTAGGACTATGTTTAATCTCCCAGATCCTAACAGGCTTATTTCTTTCC
ATACACTACACTGCCGATATCAGCACAGCTTTTTCATCCATCACCCACATCTGCCG
AGACGTAAACTACGGATGGCTAATTCGAAATTTACATGCCAACGGAGCATCCTTT
TTTTTCATTTGTATCTATTTACACATTGCACGAGGCCTTTATTACGGCTCATTTCTC
TACAAAGAAACATGAAACATCGGAGTAATCCTTCTACTATTAGTAATAATAACCG
CTTTTGTAGGCTATGTACTCCCATGAGGACAAATATCGTTTTGAGGGGCTACAGT
CATTACCAACCTACTTTCCGCTGTTCCTTACATTGGAAACTCACTAGTTCAATGAA
TCTGAGGTGGCTTCTCAATCGACAATGCCACCCTCACACGATTCTTCGCTATCCAT
TTTCTGCTTCCATTTGTTATCGCAGCCCTAACCCTAATTCACCTAATTTTTCTTCAT
GAGACAGGATCCACCAACCCAATTGGACTAAGCCCAAACACAGACAAAATCCCC
TTCCACCCATACTTCTCGTACAAAGACCTTCTCGGCTTCTTAATCCTACTTTTAAC
TTTAATGTTTGGTGCCTGAACTGGTATAGCAGGAACCGCACTAAGCATGCTAATT
CGAGCTGAACTAACTCAGCCCGGTTCCTTTTTTGGGGACGACCAAATCTATAATG
TAATTGTTACTGCACATGCCTTCGTAATAATTTTTTTTATAGTGATGCCAATTATG
ATTGGCGGATTTGGTAATTGATTAATTCCACTAATAATTGGTGCCCCAGATATGG
CTTTCCCTCGCATGAATAATATAAGCTTTTGACTACTACCCCCCTCCTTCCTCCTC
CTCCTTGCCTCCTCCACTGTTGAAGCTGGAGTAGGAACAGGTTGAACTGTGTACC
CCCCCCTTTCAGGAAATTTAGCTCACGACGGCCCATCTGTAGACCTAGCCATCTTC
TCCCTTCACCTAGCAGGGGTATCTTCAATTTTGGGGGCAATTAATTTTATCACCAC
TATTATCAACATAAAACCTCCAACCACCTCCCTGTATAATGCACCATTATTTATTT
GATCTCTCCTTGTCACGGCTGTTCTTTTACTTCTCTCTCTACCAGTCCTTGCTGCGG
GTATCACCATACTTCTAACAGACCGAAACCT"/>
<sequence id="seq_Amoa_Mito_Apisto_302864"
taxon="Amoa_Mito_Apisto_302864" totalcount="4"
value="CACTAATTGATCTCCCCACCCCCTCCAGCATCTCTGCTTGATGAAATTTCG
GGTCTCTATTAGGACTATGCCTGATTTCCCAAATCCTAACAGGCTTATTTCTTTCC
ATGCACTATACTGCTGACATCAACACAGCTTTCTCATCCATCACTCACATCTGCCG
GGATGTAAACTACGGATGGCTTATCCGTAATTTACATGCCAATGGAGCATCCTTT
TTTTTCATTTGTATCTATTTACACATTGCACGAGGCCTCTATTACGGCTCATTTCTC
CACAAAAAGACATGAAACATTGGGGTAATCCTCCTACTATTAGTCATAATAACCG
CTTTCGTAGGTTACGTACTCCCATGAGGGCAAATATCGTTTTGGGGGGCCACAGT
CATTACCAATTTACTTTCCGCTGTCCCTTACATTGGAAACTCACTAGTCCAATGAA
TCTGAGGTGGCTTCTCAGTCGACAATGCCACCCTTACACGATTCTTCGCTATCCAT
TTTCTGCTCCCCTTTGTTATTGCAGCCCTAACCCTAATTCACCTAATTTTTCTTCAT
GAAACAGGGTCCACTAACCCAATTGGACTAACCCCAAACACAGACAAAATCCCC
TTTCACCCATATTTCTCTTACAAAGACCTTCTCGGTTTCTTAATCCTACTTTTAACT
TTAATGTTCGGAGCCTGAGCTGGTATGGCAGGAACCGCACTAAGCATACTAATTC
GAGCTGAACTAACTCAGCCCGGCTCCTTTTTTGGGGACGATCAAATCTACAATGT
AATTGTCACTGCACATGCCTTTGTAATAATTTTTTTTATGGTGATGCCAATTATAA
TTGGCGGATTTGGTAACTGGTTAATCCCACTAATAATTGGTGCCCCGGATATGGC
TTTCCCTCGCATAAATAACATAAGCTTCTGACTATTACCCCCCTCCTTCCTCCTCC
TCCTTGCCTCCTCCACTGTTGAAGCTGGGGTAGGGACAGGCTGAACTGTATACCC
CCCGCTCTCAGGGAATTTAGCCCACAACGGCCCATCTGTAGACCTAGCCATCTTC
TCCCTACACCTAGCAGGGGCATCTTCAATTTTAGGCGCAATTAATTTTATCACCAC
TATTATTAACATAAAACCTCCAGCTACCTCCCTGTATAATGCACCATTATTCATCT
GATCCCTCCTTGTCACGGCTGTCCTTTTACTTCTTTCCTTACCAGTCCTTGCTGCAG
GTATCACTATACTTCTAACAGATCGAAACCT"/>

<sequence id="seq_Amoa_Mito_Apisto_302865"
taxon="Amoa_Mito_Apisto_302865" totalcount="4"
value="CACTAATTGATCTCCCCACCCCCTCCAGCATCTCTGCTTGATGAAATTTCG
GGTCTCTATTAGGACTATGCCTGATTTCCCAAATCCTAACAGGCTTATTTCTTTCC
ATGCACTATACTGCTGACATCAACACAGCTTTCTCATCCATCACTCACATCTGCCG
GGATGTAAACTACGGATGGCTTATCCGTAATTTACATGCCAATGGAGCATCCTTT
TTTTTCATTTGTATCTATTTACACATTGCACGAGGCCTCTATTACGGCTCATTTCTC
CACAAAAAGACATGAAACATTGGGGTAATCCTCCTACTATTAGTCATAATAACCG
CTTTCGTAGGTTACGTACTCCCATGAGGGCAAATATCGTTTTGGGGGGCCACAGT
CATTACCAATTTACTTTCCGCTGTCCCTTACATTGGAAACTCACTAGTCCAATGAA
TCTGAGGTGGCTTCTCAGTCGACAATGCCACCCTTACACGATTCTTCGCTATCCAT
TTTCTGCTCCCCTTTGTTATTGCAGCCCTAACCCTAATTCACCTAATTTTTCTTCAT
GAAACAGGGTCCACTAACCCAATTGGACTAACCCCAAACACAGACAAAATCCCC
TTTCACCCATATTTCTCTTACAAAGACCTTCTCGGTTTCTTAATCCTACTTTTAACT
TTAATGTTCGGAGCCTGAGCTGGTATGGCAGGAACCGCACTAAGCATACTAATTC
GAGCTGAACTAACTCAGCCCGGCTCCTTTTTTGGGGACGATCAAATCTACAATGT
AATTGTCACTGCACATGCCTTTGTAATAATTTTTTTTATGGTGATGCCAATTATAA
TTGGCGGATTTGGTAACTGGTTAATCCCACTAATAATTGGTGCCCCGGATATGGC
TTTCCCTCGCATAAATAACATAAGCTTCTGACTATTACCCCCCCTCCTTCCTCCTCC
TCCTTGCCTCCTCCACTGTTGAAGCTGGGGTAGGGACAGGCTGAACTGTATACCC
CCCGCTCTCAGGGAATTTAGCCCACAACGGCCCATCTGTAGACCTAGCCATCTTC
TCCCTACACCTAGCAGGGGCATCTTCAATTTTAGGCGCAATTAATTTTATCACCAC
TATTATTAACATAAAACCTCCAGCTACCTCCCTGTATAATGCACCATTATTCATCT
GATCCCTCCTTGTCACGGCTGTCCTTTTACTTCTTTCCTTACCAGTCCTTGCTGCAG
GTATCACTATACTTCTAACAGATCGAAACCT"/>
<sequence id="seq_Amoa_Mito_Apisto_302866"
taxon="Amoa_Mito_Apisto_302866" totalcount="4"
value="CACTAATTGATCTCCCCACCCCCTCCAGCATCTCTGCTTGATGAAATTTCG
GGTCTCTATTAGGACTATGCCTGATTTCCCAAATCCTAACAGGCTTATTTCTTTCC
ATGCACTATACTGCTGACATCAACACAGCTTTCTCATCCATCACTCACATCTGCCG
GGATGTAAACTACGGATGGCTTATCCGTAATTTACATGCCAATGGAGCATCCTTT
TTTTTCATTTGTATCTATTTACACATTGCACGAGGCCTCTATTACGGCTCATTTCTC
CACAAAAAGACATGAAACATTGGGGTAATCCTCCTACTATTAGTCATAATAACCG
CTTTCGTAGGTTACGTACTCCCATGAGGGCAAATATCGTTTTGGGGGGCCACAGT
CATTACCAATTTACTTTCCGCTGTCCCTTACATTGGAAACTCACTAGTCCAATGAA
TCTGAGGTGGCTTCTCAGTCGACAATGCCACCCTTACACGATTCTTCGCTATCCAT
TTTCTGCTCCCCTTTGTTATTGCAGCCCTAACCCTAATTCACCTAATTTTTCTTCAT
GAAACAGGGTCCACTAACCCAATTGGACTAACCCCAAACACAGACAAAATCCCC
TTTCACCCATATTTCTCTTACAAAGACCTTCTCGGTTTCTTAATCCTACTTTTAACT
TTAATGTTCGGAGCCTGAGCTGGTATGGCAGGAACCGCACTAAGCATACTAATTC
GAGCTGAACTAACTCAGCCCGGCTCCTTTTTTGGGGACGATCAAATCTACAATGT
AATTGTCACTGCACATGCCTTTGTAATAATTTTTTTTATGGTGATGCCAATTATAA
TTGGCGGATTTGGTAACTGGTTAATCCCACTAATAATTGGTGCCCCGGATATGGC
TTTCCCTCGCATAAATAACATAAGCTTCTGACTATTACCCCCCCTCCTTCCTCCTCC
TCCTTGCCTCCTCCACTGTTGAAGCTGGGGTAGGGACAGGCTGAACTGTATACCC
CCCGCTCTCAGGGAATTTAGCCCACAACGGCCCATCTGTAGACCTAGCCATCTTC
TCCCTACACCTAGCAGGGGCATCTTCAATTTTAGGCGCAATTAATTTTATCACCAC
TATTATTAACATAAAACCTCCAGCTACCTCCCTGTATAATGCACCATTATTCATCT
GATCCCTCCTTGTCACGGCTGTCCTTTTACTTCTTTCCTTACCAGTCCTTGCTGCAG
GTATCACTATACTTCTAACAGATCGAAACCT"/>

<sequence id="seq_Amoa_Mito_Apisto_302867"
taxon="Amoa_Mito_Apisto_302867" totalcount="4"
value="CACTAATTGATCTCCCCACCCCCTCCAGCATCTCTGCTTGATGAAATTTCG
GGTCTCTATTAGGACTATGCCTGATTTCCCAAATCCTAACAGGCTTATTTCTTTCC
ATGCACTATACTGCTGACATCAACACAGCTTTCTCATCCATCACCCACATCTGCCG
GGATGTAAACTACGGATGGCTTATCCGTAATTTACATGCCAATGGAGCATCCTTT
TTTTTCATTTGTATCTATTTACACATTGCACGAGGCCTCTATTACGGCTCATTTCTC
CACAAAAAGACATGAAACATTGGGGTAATCCTCCTACTATTAGTCATAATAACCG
CTTTCGTAGGTTACGTACTCCCATGAGGCAAATATCGTTTTGGGGGGCCACAGT
CATTACCAATTTACTTTCCGCTGTCCCTTACATTGGAAACTCACTAGTCCAATGAA
TCTGAGGTGGCTTCTCAGTCGACAATGCCACCCTTACACGATTCTTCGCTATCCAT
TTTCTGCTCCCCTTTGTTATTGCAGCCCTAACCCTAATTCACCTAATTTTTCTTCAT
GAAACAGGGTCCACTAACCCAATTGGACTAACCCCAAACACAGACAAAATCCCC
TTTCACCCATATTTCTCTTACAAAGACCTTCTCGGTTTCTTAATCCTACTTTTAACT
TTAATGTTCGGAGCCTGAGCTGGTATGGCAGGAACCGCACTAAGCATACTAATTC
GAGCTGAACTAACTCAGCCCGGCTCCTTTTTTGGGGACGATCAAATCTACAATGT
AATTGTCACTGCACATGCCTTTGTAATAATTTTTTTTATGGTGATGCCAATTATAA
TTGGCGGATTTGGTAACTGGTTAATCCCACTAATAATTGGTGCCCCGGATATGGC
TTTCCCTCGCATAAATAACATAAGCTTCTGACTATTACCCCCCTCCTTCCTCCTCC
TCCTTGCCTCCTCCACTGTTGAAGCTGGGGTAGGGACAGGCTGAACTGTATACCC
CCCGCTCTCAGGGAATTTAGCCCACAACGGCCCATCTGTAGACCTAGCCATCTTC
TCCCTACACCTAGCAGGGGCATCTTCAATTTTAGGCGCAATTAATTTTATCACCAC
TATTATTAACATAAAACCTCCAGCTACCTCCCTGTATAATGCACCATTATTCATCT
GATCCCTCCTTGTCACGGCTGTCCTTTTACTTCTTTCCTTACCAGTCCTTGCTGCAG
GTATCACTATACTTCTAACAGATCGAAACCT"/>
<sequence id="seq_Amoa_Mito_Apisto_302868"
taxon="Amoa_Mito_Apisto_302868" totalcount="4"
value="CACTAATTGATCTCCCCACCCCCTCCAGCATCTCTGCTTGATGAAATTTCG
GGTCTCTATTAGGACTATGCCTGATTTCCCAAATCCTAACAGGCTTATTTCTTTCC
ATGCACTATACTGCTGACATCAACACAGCTTTCTCATCCATCACCCACATCTGCCG
GGATGTAAACTACGGATGGCTTATCCGTAATTTACATGCCAATGGAGCATCCTTT
TTTTTCATTTGTATCTATTTACACATTGCACGAGGCCTCTATTACGGCTCATTTCTC
CACAAAAAGACATGAAACATTGGGGTAATCCTCCTACTATTAGTCATAATAACCG
CTTTCGTAGGTTACGTACTCCCATGAGGCAAATATCGTTTTGGGGGGCCACAGT
CATTACCAATTTACTTTCCGCTGTCCCTTACATTGGAAACTCACTAGTCCAATGAA
TCTGAGGTGGCTTCTCAGTCGACAATGCCACCCTTACACGATTCTTCGCTATCCAT
TTTCTGCTCCCCTTTGTTATTGCAGCCCTAACCCTAATTCACCTAATTTTTCTTCAT
GAAACAGGGTCCACTAACCCAATTGGACTAACCCCAAACACAGACAAAATCCCC
TTTCACCCATATTTCTCTTACAAAGACCTTCTCGGTTTCTTAATCCTACTTTTAACT
TTAATGTTCGGAGCCTGAGCTGGTATGGCAGGAACCGCACTAAGCATACTAATTC
GAGCTGAACTAACTCAGCCCGGCTCCTTTTTTGGGGACGATCAAATCTACAATGT
AATTGTCACTGCACATGCCTTTGTAATAATTTTTTTTATGGTGATGCCAATTATAA
TTGGCGGATTTGGTAACTGGTTAATCCCACTAATAATTGGTGCCCCGGATATGGC
TTTCCCTCGCATAAATAACATAAGCTTCTGACTATTACCCCCCTCCTTCCTCCTCC
TCCTTGCCTCCTCCACTGTTGAAGCTGGGGTAGGGACAGGCTGAACTGTATACCC
CCCGCTCTCAGGGAATTTAGCCCACAACGGCCCATCTGTAGACCTAGCCATCTTC
TCCCTACACCTAGCAGGGGCATCTTCAATTTTAGGCGCAATTAATTTTATCACCAC
TATTATTAACATAAAACCTCCAGCTACCTCCCTGTATAATGCACCATTATTCATCT
GATCCCTCCTTGTCACGGCTGTCCTTTTACTTCTTTCCTTACCAGTCCTTGCTGCAG
GTATCACTATACTTCTAACAGATCgaAACCT"/>

<sequence id="seq_Amor_Mito_Apisto_301601”
taxon="Amor_Mito_Apisto_301601” totalcount="4"
value="CACTAATTGACCTCCCCACCCCCTCCAACATCTCTGCTTGATGAAATTTCG
GGTCTCTATTAGGACTATGTTTAATCTCCCAAATCCTAACAGGCTTATTTCTTTCC
ATACACTACACTGCCGATATCAACACAGCTTTTTCATCCATCACCCACATCTGCCG
AGACGTAAACTACGGATGGCTAATCCGAAATTTACATGCCAACGGAGCATCCTTT
TTTTTCATTTGTCTCTATTTACACATTGCACGAGGCCTCTATTACGGCTCATTTCTC
TACAAAGAAACATGAAGCATCGGGGTAATCCTTCTACTATTAGTAATAATAACCG
CTTTTGTAGGCTATGTACTCCCATGAGGGCAAATATCGTTTTGAGGGGCTACAGT
CATTACCAACCTACTTTCCGCTGTTCCCTACATTGGAAACTCACTAGTTCAATGAA
TCTGAGGTGGCTTCTCAGTCGACAATGCCACCCTCACACGATTCTTCGCTATCCAT
TTTCTGCTTCCATTTGTTATCGCAGCCCTAACCCTAATTCACCTAATTTTTCTTCAT
GAGACAGGATCCACCAACCCAATTGGACTAAGCCCAAACACAGACAAAATCTCC
TTCCACCCATACTTCTCGTACAAAGACCTTCTCGGCTTCTTAATCCTACTTTTAAC
TTTAATGTTTGGTGCCTGAGCTGGTATAGCAGGAACCGCACTAAGCATGCTAATT
CGAGCTGAACTAACTCAGCCCGGTTCCTTTTTTGGGGACGACCAAATCTATAATG
TGATTGTTACTGCACATGCCTTCGTAATAATTTTTTTTATAGTGATGCCAATTATG
ATCGGCGGATTTGGTAATTGATTAATTCCACTAATAATTGGTGCCCCAGATATGG
CTTTCCCTCGCATGAATAATATAAGCTTTTGACTACTACCCCCCTCCTTCCTCCTC
CTCCTTGCCTCCTCCACTGTTGAAGCTGGGGTGGGAACAGGCTGAACTGTGTACC
CCCCCCTCTCAGGAAATTTAGCTCACGACGGCCCATCTGTAGACCTAGCCATCTT
CTCCCTTCACCTAGCAGGGGTATCTTCAATTTTGGGGGCAATTAATTTTATCACCA
CTATTATCAACATAAAACCTCCAACCACCTCCCTGTATAATGCACCATTATTTATT
TGATCTCTCCTTGTCACGGCTGTTCTTTTACTTCTCTCTCTACCAGTCCTTGCTGCA
GGTATCACCATACTTCTAACAGATCGAAACCT"/>
<sequence id="seq_Amor_Mito_Apisto_302772"
taxon="Amor_Mito_Apisto_302772" totalcount="4"
value="CACTAATTGACCTCCCCACCCCCTCCAACATCTCTGCTTGATGAAATTTCG
GGTCTCTATTAGGACTATGTTTAATCTCCCAAATCCTAACAGGCTTATTTCTTTCC
ATACACTACACTGCCGATATCAACACAGCTTTTTCATCCATCACCCACATCTGCCG
AGACGTAAACTACGGATGGCTAATCCGAAATTTACATGCCAACGGAGCATCCTTT
TTTTTCATTTGTCTCTATTTACACATTGCACGAGGCCTCTATTACGGCTCATTTCTC
TACAAAGAAACATGAAGCATCGGGGTAATCCTTCTACTATTAGTAATAATAACCG
CTTTTGTAGGCTATGTACTCCCATGAGGACAAATATCGTTTTGAGGGGCTACAGT
CATTACCAACCTACTTTCCGCTGTTCCCTACATTGGAAACTCACTAGTTCAATGAA
TCTGAGGTGGCTTCTCAGTCGACAATGCCACCCTCACACGATTCTTCGCTATCCAT
TTTCTGCTTCCATTTGTTATCGCAGCCCTAACCCTAATTCACCTAATTTTTCTTCAT
GAGACAGGATCCACCAACCCAATTGGACTAAGCCCAAACACAGACAAAATCTCC
TTCCACCCATACTTCTCGTACAAAGACCTTCTCGGCTTCTTAATCCTACTTTTAAC
TTTAATGTTTGGTGCCTGAGCTGGTATAGCAGGAACCGCACTAAGCATGCTAATT
CGAGCTGAACTAACTCAGCCCGGTTCCTTTTTTGGGGACGACCAAATCTATAATG
TGATTGTTACTGCACATGCCTTCGTAATAATTTTTTTTATAGTGATGCCAATTATG
ATCGGCGGATTTGGTAATTGATTAATTCCACTAATAATTGGTGCCCCAGATATGG
CTTTCCCTCGCATGAATAATATAAGCTTTTGACTACTACCCCCCTCCTTCCTCCTC
CTCCTTGCCTCCTCCACTGTTGAAGCTGGGGTGGGAACAGGCTGAACTGTGTACC
CCCCCCTCTCAGGAAATTTAGCTCACGACGGCCCATCTGTAGACCTAGCCATCTT
CTCCCTTCACCTAGCAGGGGTATCTTCAATTTTGGGGGCAATTAATTTTATCACCA
CTATTATCAACATAAAACCTCCAACCACCTCCCTGTATAATGCACCATTATTTATT
TGATCTCTCCTTGTCACGGCTGTTCTTTTACTTCTCTCTCTACCAGTCCTTGCTGCA
GGTATCACCATACTTCTAACAGATCGAAACCT"/>

<sequence id="seq_Amor_Mito_Apisto_302773"
taxon="Amor_Mito_Apisto_302773" totalcount="4"
value="CACTAATTGACCTCCCCACCCCCTCCAACATCTCTGCTTGATGAAATTTCG
GGTCTCTATTAGGACTATGTTTAATCTCCCAAATCCTAACAGGCTTATTTCTTTCC
ATACACTACACTGCCGATATCAACACAGCTTTTTCATCCATCACCCACATCTGCCG
AGACGTAAACTACGGATGGCTAATCCGAAATTTACATGCCAACGGAGCATCCTTT
TTTTTCATTTGTCTCTATTTACACATTGCACGAGGCCTCTATTACGGCTCATTTCTC
TACAAAGAAACATGAAGCATCGGGGTAATCCTTCTACTATTAGTAATAATAACCG
CTTTTGTAGGCTATGTACTCCCATGAGGACAAATATCGTTTTGAGGGGCTACAGT
CATTACCAACCTACTTTCCGCTGTTCCCTACATTGGAAACTCACTAGTTCAATGAA
TCTGAGGTGGCTTCTCAGTCGACAATGCCACCCTCACACGATTCTTCGCTATCCAT
TTTCTGCTTCCATTTGTTATCGCAGCCCTAACCCTAATTCACCTAATTTTTCTTCAT
GAGACAGGATCCACCAACCCAATTGGACTAAGCCCAAACACAGACAAAATCTCC
TTCCACCCATACTTCTCGTACAAAGACCTTCTCGGCTTCTTAATCCTACTTTTAAC
TTTAATGTTTGGTGCCTGAGCTGGTATAGCAGGAACCGCACTAAGCATGCTAATT
CGAGCTGAACTAACTCAGCCCGGTTCCTTTTTTGGGGACGACCAAATCTATAATG
TGATTGTTACTGCACATGCCTTCGTAATAATTTTTTTTATAGTGATGCCAATTATG
ATCGGCGGATTTGGTAATTGATTAATTCCACTAATAATTGGTGCCCCAGATATGG
CTTTCCCTCGCATGAATAATATAAGCTTTTGACTACTACCCCCCTCCTTCCTCCTC
CTCCTTGCCTCCTCCACTGTTGAAGCTGGGGTGGGAACAGGCTGAACTGTGTACC
CCCCCCTCTCAGGAAATTTAGCTCACGACGGCCCATCTGTAGACCTAGCCATCTT
CTCCCTTCACCTAGCAGGGGTATCTTCAATTTTGGGGGCAATTAATTTTATCACCA
CTATTATCAACATAAAACCTCCAACCACCTCCCTGTATAATGCACCATTATTTATT
TGATCTCTCCTTGTCACGGCTGTTCTTTTACTTCTCTCTCTACCAGTCCTTGCTGCA
GGTATCACCATACTTCTAACAGATCGAAACCT"/>
<sequence id="seq_Amor_Mito_Apisto_302774"
taxon="Amor_Mito_Apisto_302774" totalcount="4"
value="CACTAATTGACCTCCCCACCCCCTCCAACATCTCTGCTTGATGAAATTTCG
GGTCTCTATTAGGACTATGTTTAATCTCCCAAATCCTAACAGGCTTATTTCTTTCC
ATACACTACACTGCCGATATCAACACAGCTTTTTCATCCATCACCCACATCTGCCG
AGACGTAAACTACGGATGGCTAATCCGAAATTTACATGCCAACGGAGCATCCTTT
TTTTTCATTTGTCTCTATTTACACATTGCACGAGGCCTCTATTACGGCTCATTTCTC
TACAAAGAAACATGAAGCATCGGGGTAATCCTTCTACTATTAGTAATAATAACCG
CTTTTGTAGGCTATGTACTCCCATGAGGACAAATATCGTTTTGAGGGGCTACAGT
CATTACCAACCTACTTTCCGCTGTTCCCTACATTGGAAACTCACTAGTTCAATGAA
TCTGAGGTGGCTTCTCAGTCGACAATGCCACCCTCACACGATTCTTCGCTATCCAT
TTTCTGCTTCCATTTGTTATCGCAGCCCTAACCCTAATTCACCTAATTTTTCTTCAT
GAGACAGGATCCACCAACCCAATTGGACTAAGCCCAAACACAGACAAAATCTCC
TTCCACCCATACTTCTCGTACAAAGACCTTCTCGGCTTCTTAATCCTACTTTTAAC
TTTAATGTTTGGTGCCTGAGCTGGTATAGCAGGAACCGCACTAAGCATGCTAATT
CGAGCTGAACTAACTCAGCCCGGTTCCTTTTTTGGGGACGACCAAATCTATAATG
TGATTGTTACTGCACATGCCTTCGTAATAATTTTTTTTATAGTGATGCCAATTATG
ATCGGCGGATTTGGTAATTGATTAATTCCACTAATAATTGGTGCCCCAGATATGG
CTTTCCCTCGCATGAATAATATAAGCTTTTGACTACTACCCCCCTCCTTCCTCCTC
CTCCTTGCCTCCTCCACTGTTGAAGCTGGGGTGGGAACAGGCTGAACTGTGTACC
CCCCCCTCTCAGGAAATTTAGCTCACGACGGCCCATCTGTAGACCTAGCCATCTT
CTCCCTTCACCTAGCAGGGGTATCTTCAATTTTGGGGGCAATTAATTTTATCACCA
CTATTATCAACATAAAACCTCCAACCACCTCCCTGTATAATGCACCATTATTTATT
TGATCTCTCCTTGTCACGGCTGTTCTTTTACTTCTCTCTCTACCAGTCCTTGCTGCA
GGTATCACCATACTTCTAACAGATCGAAACCT"/>

<sequence id="seq_Amor_Mito_Apisto_302775"
taxon="Amor_Mito_Apisto_302775" totalcount="4"
value="CACTAATTGACCTCCCCACCCCCTCCAACATCTCTGCTTGATGAAATTTCG
GGTCTCTATTAGGACTATGTTTAATCTCCCAAATCCTAACAGGCTTATTTCTTTCC
ATACACTACACTGCCGATATCAACACAGCTTTTCATCCATCACCCACATCTGCCG
AGACGTAAACTACGGATGGCTAATCCGAAATTTACATGCCAACGGAGCATCCTTT
TTTTTCATTTGTCTCTATTTACACATTGCACGAGGCCTCTATTACGGCTCATTTCTC
TACAAAGAAACATGAAGCATCGGGGTAATCCTTCTACTATTAGTAATAATAACCG
CTTTTGTAGGCTATGTACTCCCATGAGGACAAATATCGTTTTGAGGGGCTACAGT
CATTACCAACCTACTTTCCGCTGTTCCCTACATTGGAAACTCACTAGTTCAATGAA
TCTGAGGTGGCTTCTCAGTCGACAATGCCACCCTCACACGATTCTTCGCTATCCAT
TTTCTGCTTCCATTTGTTATCGCAGCCCTAACCCTAATTCACCTAATTTTTCTTCAT
GAGACAGGATCCACCAACCCAATTGGACTAAGCCCAAACACAGACAAAATCTCC
TTCCACCCATACTTCTCGTACAAAGACCTTCTCGGCTTCTTAATCCTACTTTTAAC
TTTAATGTTTGGTGCCTGAGCTGGTATAGCAGGAACCGCACTAAGCATGCTAATT
CGAGCTGAACTAACTCAGCCCGGTTCCTTTTTTGGGGACGACCAAATCTATAATG
TGATTGTTACTGCACATGCCTTCGTAATAATTTTTTTTATAGTGATGCCAATTATG
ATCGGCGGATTTGGTAATTGATTAATTCCACTAATAATTGGTGCCCCAGATATGG
CTTTCCCTCGCATGAATAATATAAGCTTTTGACTACTACCCCCCTCCTTCCTCCTC
CTCCTTGCCTCCTCCACTGTTGAAGCTGGGGTGGGAACAGGCTGAACTGTGTACC
CCCCCCTCTCAGGAAATTTAGCTCACGACGGCCCATCTGTAGACCTAGCCATCTT
CTCCCTTCACCTAGCAGGGGTATCTTCAATTTTGGGGGCAATTAATTTTATCACCA
CTATTATCAACATAAAACCTCCAACCACCTCCCTGTATAATGCACCATTATTTATT
TGATCTCTCCTTGTCACGGCTGTTCTTTTACTTCTCTCTCTACCAGTCCTTGCTGCA
GGTATCACCATACTTCTAACAGATCGAAACCT"/>
<sequence id="seq_Amor_Mito_Apisto_302776"
taxon="Amor_Mito_Apisto_302776" totalcount="4"
value="CACTAATTGACCTCCCCACCCCCTCCAACATCTCTGCTTGATGAAATTTCG
GGTCTCTATTAGGACTATGTTTAATCTCCCAAATCCTAACAGGCTTATTTCTTTCC
ATACACTACACTGCCGATATCAACACAGCTTTTCATCCATCACCCACATCTGCCG
AGACGTAAACTACGGATGGCTAATCCGAAATTTACATGCCAACGGAGCATCCTTT
TTTTTCATTTGTCTCTATTTACACATTGCACGAGGCCTCTATTACGGCTCATTTCTC
TACAAAGAAACATGAAGCATCGGGGTAATCCTTCTACTATTAGTAATAATAACCG
CTTTTGTAGGCTATGTACTCCCATGAGGACAAATATCGTTTTGAGGGGCTACAGT
CATTACCAACCTACTTTCCGCTGTTCCCTACATTGGAAACTCACTAGTTCAATGAA
TCTGAGGTGGCTTCTCAGTCGACAATGCCACCCTCACACGATTCTTCGCTATCCAT
TTTCTGCTTCCATTTGTTATCGCAGCCCTAACCCTAATTCACCTAATTTTTCTTCAT
GAGACAGGATCCACCAACCCAATTGGACTAAGCCCAAACACAGACAAAATCTCC
TTCCACCCATACTTCTCGTACAAAGACCTTCTCGGCTTCTTAATCCTACTTTTAAC
TTTAATGTTTGGTGCCTGAGCTGGTATAGCAGGAACCGCACTAAGCATGCTAATT
CGAGCTGAACTAACTCAGCCCGGTTCCTTTTTTGGGGACGACCAAATCTATAATG
TGATTGTTACTGCACATGCCTTCGTAATAATTTTTTTTATAGTGATGCCAATTATG
ATCGGCGGATTTGGTAATTGATTAATTCCACTAATAATTGGTGCCCCAGATATGG
CTTTCCCTCGCATGAATAATATAAGCTTTTGACTACTACCCCCCTCCTTCCTCCTC
CTCCTTGCCTCCTCCACTGTTGAAGCTGGGGTGGGAACAGGCTGAACTGTGTACC
CCCCCCTCTCAGGAAATTTAGCTCACGACGGCCCATCTGTAGACCTAGCCATCTT
CTCCCTTCACCTAGCAGGGGTATCTTCAATTTTGGGGGCAATTAATTTTATCACCA
CTATTATCAACATAAAACCTCCAACCACCTCCCTGTATAATGCACCATTATTTATT
TGATCTCTCCTTGTCACGGCTGTTCTTTTACTTCTCTCTCTACCAGTCCTTGCTGCA
GGTATCACCATACTTCTAACAGATCGAAACCT"/>

<sequence id="seq_Amor_Mito_Apisto_302816"
taxon="Amor_Mito_Apisto_302816" totalcount="4"
value="CACTAATTGACCTCCCCACCCCCTCCAACATCTCTGCTTGATGAAATTTCG
GGTCTCTATTAGGACTATGTTTAATCTCCCAAATCCTAACAGGCTTATTTCTTTCC
ATACACTACACTGCCGATATCAACACAGCTTTTTCATCCATCACCCACATCTGCCG
AGACGTAAACTACGGATGGCTAATCCGAAATTTACATGCCAACGGAGCATCCTTT
TTTTTCATTTGTCTCTATTTACACATTGCACGAGGCCTCTATTACGGCTCATTTCTC
TACAAAGAAACATGAAGCATCGGGGTAATCCTTCTACTATTAGTAATAATAACCG
CTTTTGTAGGCTATGTACTCCCATGAGGACAAATATCGTTTTGAGGGGCTACAGT
CATTACCAACCTACTTTCCGCTGTTCCCTACATTGGAAACTCACTAGTTCAATGAA
TCTGAGGTGGCTTCTCAGTCGACAATGCCACCCTCACACGATTCTTCGCTATCCAT
TTTCTGCTTCCATTTGTTATCGCAGCCCTAACCCTAATTCACCTAATTTTTCTTCAT
GAGACAGGATCCACCAACCCAATTGGACTAAGCCCAAACACAGACAAAATCTCC
TTCCACCCATACTTCTCGTACAAAGACCTTCTCGGCTTCTTAATCCTACTTTTAAC
TTTAATGTTTGGTGCCTGAGCTGGTATAGCAGGAACCGCACTAAGCATGCTAATT
CGAGCTGAACTAACTCAGCCCGGTTCCTTTTTTGGGGACGACCAAATCTATAATG
TGATTGTTACTGCACATGCCTTCGTAATAATTTTTTTTATAGTGATGCCAATTATG
ATCGGCGGATTTGGTAATTGATTAATTCCACTAATAATTGGTGCCCCAGATATGG
CTTTCCCTCGCATGAATAATATAAGCTTTTGACTACTACCCCCCTCCTTCCTCCTC
CTCCTTGCCTCCTCCACTGTTGAAGCTGGGGTGGGAACAGGCTGAACTGTGTACC
CCCCCCTCTCAGGAAATTTAGCTCACGACGGCCCATCTGTAGACCTAGCCATCTT
CTCCCTTCACCTAGCAGGGGTATCTTCAATTTTGGGGGCAATTAATTTTATCACCA
CTATTATCAACATAAAACCTCCAACCACCTCCCTGTATAATGCACCATTATTTATT
TGATCTCTCCTTGTCACGGCTGTTCTTTTACTTCTCTCTCTACCAGTCCTTGCTGCA
GGTATCACCATACTTCTAACAGATCGAAACCT"/>
<sequence id="seq_Amor_Mito_Apisto_302817"
taxon="Amor_Mito_Apisto_302817" totalcount="4"
value="CACTAATTGACCTCCCCACCCCCTCCAACATCTCTGCTTGATGAAATTTCG
GGTCTCTATTAGGACTATGTTTAATCTCCCAAATCCTAACAGGCTTATTTCTTTCC
ATACACTACACTGCCGATATCAACACAGCTTTTTCATCCATCACCCACATCTGCCG
AGACGTAAACTACGGATGGCTAATCCGAAATTTACATGCCAACGGAGCATCCTTT
TTTTTCATTTGTCTCTATTTACACATTGCACGAGGCCTCTATTACGGCTCATTTCTC
TACAAAGAAACATGAAGCATCGGGGTAATCCTTCTACTATTAGTAATAATAACCG
CTTTTGTAGGCTATGTACTCCCATGAGGACAAATATCGTTTTGAGGGGCTACAGT
CATTACCAACCTACTTTCCGCTGTTCCCTACATTGGAAACTCACTAGTTCAATGAA
TCTGAGGTGGCTTCTCAGTCGACAATGCCACCCTCACACGATTCTTCGCTATCCAT
TTTCTGCTTCCATTTGTTATCGCAGCCCTAACCCTAATTCACCTAATTTTTCTTCAT
GAGACAGGATCCACCAACCCAATTGGACTAAGCCCAAACACAGACAAAATCTCC
TTCCACCCATACTTCTCGTACAAAGACCTTCTCGGCTTCTTAATCCTACTTTTAAC
TTTAATGTTTGGTGCCTGAGCTGGTATAGCAGGAACCGCACTAAGCATGCTAATT
CGAGCTGAACTAACTCAGCCCGGTTCCTTTTTTGGGGACGACCAAATCTATAATG
TGATTGTTACTGCACATGCCTTCGTAATAATTTTTTTTATAGTGATGCCAATTATG
ATCGGCGGATTTGGTAATTGATTAATTCCACTAATAATTGGTGCCCCAGATATGG
CTTTCCCTCGCATGAATAATATAAGCTTTTGACTACTACCCCCCTCCTTCCTCCTC
CTCCTTGCCTCCTCCACTGTTGAAGCTGGGGTGGGAACAGGCTGAACTGTGTACC
CCCCCCTCTCAGGAAATTTAGCTCACGACGGCCCATCTGTAGACCTAGCCATCTT
CTCCCTTCACCTAGCAGGGGTATCTTCAATTTTGGGGGCAATTAATTTTATCACCA
CTATTATCAACATAAAACCTCCAACCACCTCCCTGTATAATGCACCATTATTTATT
TGATCTCTCCTTGTCACGGCTGTTCTTTTACTTCTCTCTCTACCAGTCCTTGCTGCA
GGTATCACCATACTTCTAACAGATCGAAACCT"/>

<sequence id="seq_Amor_Mito_Apisto_302818"
taxon="Amor_Mito_Apisto_302818" totalcount="4"
value="CACTAATTGACCTCCCCACCCCCTCCAACATCTCTGCTTGATGAAATTTCG
GGTCTCTATTAGGACTATGTTTAATCTCCCAAATCCTAACAGGCTTATTTCTTTCC
ATACACTACACTGCCGATATCAACACAGCTTTTTCATCCATCACCCACATCTGCCG
AGACGTAAACTACGGATGGCTAATCCGAAATTTACATGCCAACGGAGCATCCTTT
TTTTTCATTTGTCTCTATTTACACATTGCACGAGGCCTCTATTACGGCTCATTTCTC
TACAAAGAAACATGAAGCATCGGGGTAATCCTTCTACTATTAGTAATAATAACCG
CTTTTGTAGGCTATGTACTCCCATGAGGACAAATATCGTTTTGAGGGGCTACAGT
CATTACCAACCTACTTTCCGCTGTTCCCTACATTGGAAACTCACTAGTTCAATGAA
TCTGAGGTGGCTTCTCAGTCGACAATGCCACCCTCACACGATTCTTCGCTATCCAT
TTTCTGCTTCCATTTGTTATCGCAGCCCTAACCCTAATTCACCTAATTTTTCTTCAT
GAGACAGGATCCACCAACCCAATTGGACTAAGCCCAAACACAGACAAAATCTCC
TTCCACCCATACTTCTCGTACAAAGACCTTCTCGGCTTCTTAATCCTACTTTTAAC
TTTAATGTTTGGTGCCTGAGCTGGTATAGCAGGAACCGCACTAAGCATGCTAATT
CGAGCTGAACTAACTCAGCCCGGTTCCTTTTTTGGGGACGACCAAATCTATAATG
TGATTGTTACTGCACATGCCTTCGTAATAATTTTTTTTATAGTGATGCCAATTATG
ATCGGCGGATTTGGTAATTGATTAATTCCACTAATAATTGGTGCCCCAGATATGG
CTTTCCCTCGCATGAATAATATAAGCTTTTGACTACTACCCCCCTCCTTCCTCCTC
CTCCTTGCCTCCTCCACTGTTGAAGCTGGGGTGGGAACAGGCTGAACTGTGTACC
CCCCCCTCTCAGGAAATTTAGCTCACGACGGCCCATCTGTAGACCTAGCCATCTT
CTCCCTTCACCTAGCAGGGGTATCTTCAATTTTGGGGGCAATTAATTTTATCACCA
CTATTATCAACATAAAACCTCCAACCACCTCCCTGTATAATGCACCATTATTTATT
TGATCTCTCCTTGTCACGGCTGTTCTTTTACTTCTCTCTCTACCAGTCCTTGCTGCA
GGTATCACCATACTTCTAACAGATCGAAACCT"/>
<sequence id="seq_Amor_Mito_Apisto_302819"
taxon="Amor_Mito_Apisto_302819" totalcount="4"
value="CACTAATTGACCTCCCCACCCCCTCCAACATCTCTGCTTGATGAAATTTCG
GGTCTCTATTAGGACTATGTTTAATCTCCCAAATCCTAACAGGCTTATTTCTTTCC
ATACACTACACTGCCGATATCAACACAGCTTTTTCATCCATCACCCACATCTGCCG
AGACGTAAACTACGGATGGCTAATCCGAAATTTACATGCCAACGGAGCATCCTTT
TTTTTCATTTGTCTCTATTTACACATTGCACGAGGCCTCTATTACGGCTCATTTCTC
TACAAAGAAACATGAAGCATCGGGGTAATCCTTCTACTATTAGTAATAATAACCG
CTTTTGTAGGCTATGTACTCCCATGAGGACAAATATCGTTTTGAGGGGCTACAGT
CATTACCAACCTACTTTCCGCTGTTCCCTACATTGGAAACTCACTAGTTCAATGAA
TCTGAGGTGGCTTCTCAGTCGACAATGCCACCCTCACACGATTCTTCGCTATCCAT
TTTCTGCTTCCATTTGTTATCGCAGCCCTAACCCTAATTCACCTAATTTTTCTTCAT
GAGACAGGATCCACCAACCCAATTGGACTAAGCCCAAACACAGACAAAATCTCC
TTCCACCCATACTTCTCGTACAAAGACCTTCTCGGCTTCTTAATCCTACTTTTAAC
TTTAATGTTTGGTGCCTGAGCTGGTATAGCAGGAACCGCACTAAGCATGCTAATT
CGAGCTGAACTAACTCAGCCCGGTTCCTTTTTTGGGGACGACCAAATCTATAATG
TGATTGTTACTGCACATGCCTTCGTAATAATTTTTTTTATAGTGATGCCAATTATG
ATCGGCGGATTTGGTAATTGATTAATTCCACTAATAATTGGTGCCCCAGATATGG
CTTTCCCTCGCATGAATAATATAAGCTTTTGACTACTACCCCCCTCCTTCCTCCTC
CTCCTTGCCTCCTCCACTGTTGAAGCTGGGGTGGGAACAGGCTGAACTGTGTACC
CCCCCCTCTCAGGAAATTTAGCTCACGACGGCCCATCTGTAGACCTAGCCATCTT
CTCCCTTCACCTAGCAGGGGTATCTTCAATTTTGGGGGCAATTAATTTTATCACCA
CTATTATCAACATAAAACCTCCAACCACCTCCCTGTATAATGCACCATTATTTATT
TGATCTCTCCTTGTCACGGCTGTTCTTTTACTTCTCTCTCTACCAGTCCTTGCTGCA
GGTATCACCATACTTCTAACAGATCGAAACCT"/>

<sequence id="seq_Amor_Mito_Apisto_300564"
taxon="Amor_Mito_Apisto_300564" totalcount="4"
value="CACTAATTGACCTCCCCACCCCCTCCAACATCTCTGCTTGATGAAATTTCG
GGTCTCTATTAGGACTATGTTTAATCTCCCAAATCCTAACAGGCTTATTTCTTTCC
ATACACTACACTGCCGATATCAACACAGCTTTTTCATCCATCACCCACATCTGCCG
AGACGTAAACTACGGATGGCTAATCCGAAATTTACATGCCAACGGAGCATCCTTT
TTTTTCATTTGTCTCTATTTACACATTGCACGAGGCCTCTATTACGGCTCATTTCTC
TACAAAGAAACATGAAGCATCGGGGTAATCCTTCTACTATTAGTAATAATAACCG
CTTTTGTAGGCTATGTACTCCCATGAGGACAAATATCGTTTTGAGGGGCTACAGT
CATTACCAACCTACTTTCCGCTGTTCCCTACATTGGAAACTCACTAGTTCAATGAA
TCTGAGGTGGCTTCTCAGTCGACAATGCCACCCTCACACGATTCTTCGCTATCCAT
TTTCTGCTTCCATTTGTTATCGCAGCCCTAACCCTAATTCACCTAATTTTTCTTCAT
GAGACAGGATCCACCAACCCAATTGGACTAAGCCCAAACACAGACAAAATCTCC
TTCCACCCATACTTCTCGTACAAAGACCTTCTCGGCTTCTTAATCCTACTTTTAAC
TTTAATGTTTGGTGCCTGAGCTGGTATAGCAGGAACCGCACTAAGCATGCTAATT
CGAGCTGAACTAACTCAGCCCGGTTCCTTTTTTGGGGACGACCAAATCTATAATG
TGATTGTTACTGCACATGCCTTCGTAATAATTTTTTTTATAGTGATGCCAATTATG
ATCGGCGGATTTGGTAATTGATTAATTCCACTAATAATTGGTGCCCCAGATATGG
CTTTCCCTCGCATGAATAATATAAGCTTTTGACTACTACCCCCCTCCTTCCTCCTC
CTCCTTGCCTCCTCCACTGTTGAAGCTGGGGTGGGAACAGGCTGAACTGTGTACC
CCCCCCTCTCAGGAAATTTAGCTCACGACGGCCCATCTGTAGACCTAGCCATCTT
CTCCCTTCACCTAGCAGGGGTATCTTCAATTTTGGGGGCAATTAATTTTATCACCA
CTATTATCAACATAAAACCTCCAACCACCTCCCTGTATAATGCACCATTATTTATT
TGATCTCTCCTTGTCACGGCTGTTCTTTTACTTCTCTCTCTACCAGTCCTTGCTGCA
GGTATCACCATACTTCTAACAGATCGAAACCT"/>
<sequence id="seq_Amor_Mito_Apisto_300577"
taxon="Amor_Mito_Apisto_300577" totalcount="4"
value="CACTAATTGACCTCCCCACCCCCTCCAACATCTCTGCTTGATGAAATTTCG
GGTCTCTATTAGGACTATGTTTAATCTCCCAAATCCTAACAGGCTTATTTCTTTCC
ATACACTACACTGCCGATATCAACACAGCTTTTTCATCCATCACCCACATCTGCCG
AGACGTAAACTACGGATGGCTAATCCGAAATTTACATGCCAACGGAGCATCCTTT
TTTTTCATTTGTCTCTATTTACACATTGCACGAGGCCTCTATTACGGCTCATTTCTC
TACAAAGAAACATGAAGCATCGGGGTAATCCTTCTACTATTAGTAATAATAACCG
CTTTTGTAGGCTATGTACTCCCATGAGGACAAATATCGTTTTGAGGGGCTACAGT
CATTACCAACCTACTTTCCGCTGTTCCCTACATTGGAAACTCACTAGTTCAATGAA
TCTGAGGTGGCTTCTCAGTCGACAATGCCACCCTCACACGATTCTTCGCTATCCAT
TTTCTGCTTCCATTTGTTATCGCAGCCCTAACCCTAATTCACCTAATTTTTCTTCAT
GAGACAGGATCCACCAACCCAATTGGACTAAGCCCAAACACAGACAAAATCTCC
TTCCACCCATACTTCTCGTACAAAGACCTTCTCGGCTTCTTAATCCTACTTTTAAC
TTTAATGTTTGGTGCCTGAGCTGGTATAGCAGGAACCGCACTAAGCATGCTAATT
CGAGCTGAACTAACTCAGCCCGGTTCCTTTTTTGGGGACGACCAAATCTATAATG
TGATTGTTACTGCACATGCCTTCGTAATAATTTTTTTTATAGTGATGCCAATTATG
ATCGGCGGATTTGGTAATTGATTAATTCCACTAATAATTGGTGCCCCAGATATGG
CTTTCCCTCGCATGAATAATATAAGCTTTTGACTACTACCCCCCTCCTTCCTCCTC
CTCCTTGCCTCCTCCACTGTTGAAGCTGGGGTGGGAACAGGCTGAACTGTGTACC
CCCCCCTCTCAGGAAATTTAGCTCACGACGGCCCATCTGTAGACCTAGCCATCTT
CTCCCTTCACCTAGCAGGGGTATCTTCAATTTTGGGGGCAATTAATTTTATCACCA
CTATTATCAACATAAAACCTCCAACCACCTCCCTGTATAATGCACCATTATTTATT
TGATCTCTCCTTGTCACGGCTGTTCTTTTACTTCTCTCTCTACCAGTCCTTGCTGCA
GGTATCACCATACTTCTAACAGATCGAAACCT"/>

<sequence id="seq_Amor_Mito_Apisto_300578"
taxon="Amor_Mito_Apisto_300578" totalcount="4"
value="CACTAATTGACCTCCCCACCCCCTCCAACATCTCTGCTTGATGAAATTTCG
GGTCTCTATTAGGACTATGTTTAATCTCCCAAATCCTAACAGGCTTATTTCTTTCC
ATACACTACACTGCCGATATCAACACAGCTTTTTCATCCATCACCCACATCTGCCG
AGACGTAAACTACGGATGGCTAATCCGAAATTTACATGCCAACGGAGCATCCTTT
TTTTTCATTTGTCTCTATTTACACATTGCACGAGGCCTCTATTACGGCTCATTTCTC
TACAAAGAAACATGAAGCATCGGGGTAATCCTTCTACTATTAGTAATAATAACCG
CTTTTGTAGGCTATGTACTCCCATGAGGACAAATATCGTTTTGAGGGGCTACAGT
CATTACCAACCTACTTTCCGCTGTTCCCTACATTGGAAACTCACTAGTTCAATGAA
TCTGAGGTGGCTTCTCAGTCGACAATGCCACCCTCACACGATTCTTCGCTATCCAT
TTTCTGCTTCCATTTGTTATCGCAGCCCTAACCCTAATTCACCTAATTTTTCTTCAT
GAGACAGGATCCACCAACCCAATTGGACTAAGCCCAAACACAGACAAAATCTCC
TTCCACCCATACTTCTCGTACAAAGACCTTCTCGGCTTCTTAATCCTACTTTTAAC
TTTAATGTTTGGTGCCTGAGCTGGTATAGCAGGAACCGCACTAAGCATGCTAATT
CGAGCTGAACTAACTCAGCCCGGTTCCTTTTTTGGGGACGACCAAATCTATAATG
TGATTGTTACTGCACATGCCTTCGTAATAATTTTTTTTATAGTGATGCCAATTATG
ATCGGCGGATTTGGTAATTGATTAATTCCACTAATAATTGGTGCCCCAGATATGG
CTTTCCCTCGCATGAATAATATAAGCTTTTGACTACTACCCCCCTCCTTCCTCCTC
CTCCTTGCCTCCTCCACTGTTGAAGCTGGGGTGGGAACAGGCTGAACTGTGTACC
CCCCCCTCTCAGGAAATTTAGCTCACGACGGCCCATCTGTAGACCTAGCCATCTT
CTCCCTTCACCTAGCAGGGGTATCTTCAATTTTGGGGGCAATTAATTTTATCACCA
CTATTATCAACATAAAACCTCCAACCACCTCCCTGTATAATGCACCATTATTTATT
TGATCTCTCCTTGTCACGGCTGTTCTTTTACTTCTCTCTCTACCAGTCCTTGCTGCA
GGTATCACCATACTTCTAACAGATCGAAACCT"/>
<sequence id="seq_Amor_Mito_Apisto_300581"
taxon="Amor_Mito_Apisto_300581" totalcount="4"
value="CACTAATTGACCTCCCCACCCCCTCCAACATCTCTGCTTGATGAAATTTCG
GGTCTCTATTAGGACTATGTTTAATCTCCCAAATCCTAACAGGCTTATTTCTTTCC
ATACACTACACTGCCGATATCAACACAGCTTTTTCATCCATCACCCACATCTGCCG
AGACGTAAACTACGGATGGCTAATCCGAAATTTACATGCCAACGGAGCATCCTTT
TTTTTCATTTGTCTCTATTTACACATTGCACGAGGCCTCTATTACGGCTCATTTCTC
TACAAAGAAACATGAAGCATCGGGGTAATCCTTCTACTATTAGTAATAATAACCG
CTTTTGTAGGCTATGTACTCCCATGAGGACAAATATCGTTTTGAGGGGCTACAGT
CATTACCAACCTACTTTCCGCTGTTCCCTACATTGGAAACTCACTAGTTCAATGAA
TCTGAGGTGGCTTCTCAGTCGACAATGCCACCCTCACACGATTCTTCGCTATCCAT
TTTCTGCTTCCATTTGTTATCGCAGCCCTAACCCTAATTCACCTAATTTTTCTTCAT
GAGACAGGATCCACCAACCCAATTGGACTAAGCCCAAACACAGACAAAATCTCC
TTCCACCCATACTTCTCGTACAAAGACCTTCTCGGCTTCTTAATCCTACTTTTAAC
TTTAATGTTTGGTGCCTGAGCTGGTATAGCAGGAACCGCACTAAGCATGCTAATT
CGAGCTGAACTAACTCAGCCCGGTTCCTTTTTTGGGGACGACCAAATCTATAATG
TGATTGTTACTGCACATGCCTTCGTAATAATTTTTTTTATAGTGATGCCAATTATG
ATCGGCGGATTTGGTAATTGATTAATTCCACTAATAATTGGTGCCCCAGATATGG
CTTTCCCTCGCATGAATAATATAAGCTTTTGACTACTACCCCCCTCCTTCCTCCTC
CTCCTTGCCTCCTCCACTGTTGAAGCTGGGGTGGGAACAGGCTGAACTGTGTACC
CCCCCCTCTCAGGAAATTTAGCTCACGACGGCCCATCTGTAGACCTAGCCATCTT
CTCCCTTCACCTAGCAGGGGTATCTTCAATTTTGGGGGCAATTAATTTTATCACCA
CTATTATCAACATAAAACCTCCAACCACCTCCCTGTATAATGCACCATTATTTATT
TGATCTCTCCTTGTCACGGCTGTTCTTTTACTTCTCTCTCTACCAGTCCTTGCTGCA
GGTATCACCATACTTCTAACAGATCGAAACCT"/>

<sequence id="seq_Amor_Mito_Apisto_301724"
taxon="Amor_Mito_Apisto_301724" totalcount="4"
value="CACTAATTGACCTCCCCACCCCCTCCAACATCTCTGCTTGATGAAATTTCG
GGTCTCTATTAGGACTATGTTTAATCTCCCAAATCCTAACAGGCTTATTTCTTTCC
ATACACTACACTGCCGATATCAACACAGCTTTTTCATCCATCGCCCACATCTGCCG
AGACGTAAACTACGGATGGCTAATCCGAAATTTACATGCCAACGGAGCATCCTTT
TTTTTCATTTGTCTCTATTTACACATTGCACGAGGCCTCTATTACGGCTCATTTCTC
TACAAAGAAACATGAAACATCGGGGTAATCCTTCTACTATTAGTAATAATAACCG
CTTTTGTAGGCTATGTACTCCCATGAGGACAAATATCGTTTTGAGGGGCTACAGT
CATTACCAACCTACTTTCCGCTGTTCCTTACATTGGAAACTCACTAGTTCAATGAA
TCTGAGGTGGCTTCTCAGTCGACAATGCCACCCTCACACGATTCTTCGCTATCCAT
TTTCTGCTTCCATTTGTTATCGCAGCCCTAACCCTAATTCACCTAATTTTTCTTCAT
GAGACAGGATCCACCAACCCAATTGGACTAAGCCCAAACACAGACAAAATCTCC
TTCCACCCATACTTCTCGTACAAAGACCTTCTCGGCTTCTTAATCCTACTTTTAAC
TTTAATGTTTGGTGCCTGAGCTGGTATAGCAGGAACCGCACTAAGCATGCTAATT
CGAGCTGAACTAACTCAGCCCGGTTCCTTTTTTGGGGACGACCAAATCTATAATG
TAATTGTTACTGCACATGCCTTCGTAATAATTTTTTTTATAGTGATGCCAATTATG
ATCGGCGGATTTGGTAATTGATTAATTCCACTAATAATTGGTGCCCCAGATATGG
CTTTCCCTCGCATGAATAATATAAGCTTTTGACTTCTACCCCCCTCCTTCCTCCTCC
TCCTTGCCTCCTCCACTGTTGAAGCTGGGGTGGGAACAGGCTGAACTGTGTACCC
CCCCCTCTCAGGAAATTTAGCTCACGACGGCCCATCTGTAGACCTAGCCATCTTCT
CCCTTCACCTAGCAGGGGTATCTTCAATTTTGGGGGCAATTAATTTTATCACCACT
ATTATCAACATAAAACCTCCAACCACCTCCCTGTATAATGCACCATTATTTATTTG
ATCTCTCCTTGTCACGGCTGTTCTTTTACTTCTCTCTCTACCAGTCCTTGCTGCAGG
TATCACCATACTTCTAACAGATCGAAACCT"/>
<sequence id="seq_Amor_Mito_Apisto_301725”"
taxon="Amor_Mito_Apisto_301725”" totalcount="4" value="-------------------------------------
----
ACTATGTTTAATcTCCCAaAtCCTAaCAGGCTTATTTCtTTCCaTACACTACACTGCcG
ATATCAACACAGCTTTTTCATCCATcGCCCACATCTACCGAGACGTAAACTACGG
ATGGCTAATCCGAAATTtACATGCCAACGGAGCATCCTTTTTTTTCATTTGTCTCTA
TTTACACATTGCACGAGGCCTCTATTACGGCTCATTTCTCTACAAAGAAACATGA
AACATCGGGGTAATCCTTCTACTATTAGTAATAATAACCGCTTTTGTAGGCTATGT
ACTCCCATGAGGACAAATATCGTTTTGAGGGGCTACAGTCATTACCAACCTACTT
TCCGCTGTTCCTTACATTGGAAACTCACTAGTTCAATGAATCTGAGGTGGCTTCTC
AGTCGACAATGCCaCCCTCACACGATTCTTCGCTATCCATTTTCTGCTTCCATTTGT
TATCGCAGCCCTAACCCTAATTCACCTAATTTTTCTTCATGAGACAgGATCCACCA
ACCCAATTGGACTAAGCCCAAACACaGACAAAATCTCC----------------------------------
----
TGTTTGGAGCCTGAGCTGGTATAGCAGGAACCGCACTAAGCATGCTAATTCGAGC
TGAACTAACTCAGCCCGGTTCCTTTTTTGGGGACGACCAAATCTATAATGTAATT
GTTACTGCACATGCCTTCGTAATAATTTTTTTTATAGTGATGCCAATTATGATCGG
CGGATTTGGTAATTGATTAATTCCACTAATAATTGGTGCCCCAGATATGGCTTTCC
CTCGCATGAATAATATAAGCTTTTGACTTCTACCCCCCTCCTTCCTCCTCCTCCTTG
CCTCCTCCACTGTTGAAGCTGGGGTGGGAACAGGCTGAACTGTGTACCCCCCCCT
CTCAGGAAATTTAGCTCACGACGGCCCATCTGTAGACCTAGCCATCTTCTCCCTTC
ACCTAGCAGGGGTATCTTCAATTTTGGGGGCAATTAATTTTATCACCACTATTATC
AACATAAAACCTCCAACCACCTCCCTGTATAATGCACCATTATTTATTTGATCTCT
CCTTGTCACGGCTGTTCTTTTACTTCTCTCTCTACCAGTCCTTGCTGCAGGTATCAC
CATACTTCTAACAGATCGAAACCT"/>

<sequence id="seq_Amor_Mito_Apisto_301726"
taxon="Amor_Mito_Apisto_301726" totalcount="4"
value="CACTAATTGACCTCCCCACCCCCTCCAACATCTCTGCTTGATGAAATTTCG
GGTCTCTATTAGGACTATGTTTAATCTCCCAAATCCTAACAGGCTTATTTCTTTCC
ATACACTACACTGCCGATATCAACACAGCTTTTCATCCATCACCCACATCTGCCG
AGACGTAAACTACGGATGGCTAATCCGAAATTTACATGCCAACGGAGCATCCTTT
TTTTTCATTTGTCTCTATTTACACATTGCACGAGGCCTCTATTACGGCTCATTTCTC
TACAAAGAAACATGAAGCATCGGGGTAATCCTTCTACTATTAGTAATAATAACCG
CTTTTGTAGGCTATGTACTCCCATGAGGGCAAATATCGTTTTGAGGGGCTACAGT
CATTACCAACCTACTTTCCGCTGTTCCCTACATTGGAAACTCACTAGTTCAATGAA
TCTGAGGTGGCTTCTCAGTCGACAATGCCACCCTCACACGATTCTTCGCTATCCAT
TTTCTGCTTCCATTTGTTATCGCAGCCCTAACCCTAATTCACCTAATTTTTCTTCAT
GAGACAGGATCCACCAACCCAATTGGACTAAGCCCAAACACAGACAAAATCTCC
TTCCACCCATACTTCTCGTACAAAGACCTTCTCGGCTTCTTAATCCTACTTTTAAC
TTTAATGTTTGGTGCCTGAGCTGGTATAGCAGGAACCGCACTAAGCATGCTAATT
CGAGCTGAACTAACTCAGCCCGGTTCCTTTTTTGGGGACGACCAAATCTATAATG
TGATTGTTACTGCACATGCCTTCGTAATAATTTTTTTTATAGTGATGCCAATTATA
ATCGGCGGATTTGGTAATTGATTAATTCCACTAATAATTGGTGCCCCAGATATGG
CTTTCCCTCGCATGAATAATATAAGCTTTTGACTACTACCCCCCTCCTTCCTCCTC
CTCCTTGCCTCCTCCACTGTTGAAGCTGGGGTGGGAACAGGCTGAACTGTGTACC
CCCCCCTCTCAGGAAATTTAGCTCACGACGGCCCATCTGTAGACCTAGCCATCTT
CTCCCTTCACCTAGCAGGGGTATCTTCAATTTTGGGGGCAATTAATTTTATCACCA
CTATTATCAACATAAAACCTCCAACCACCTCCCTGTATAATGCACCATTATTTATT
TGATCTCTCCTTGTCACGGCTGTTCTTTTACTTCTCTCTCTACCAGTCCTTGCTGCA
GGTATCACCATACTTCTAACAGATCGAAACCT"/>
<sequence id="seq_Amor_Mito_Apisto_301727"
taxon="Amor_Mito_Apisto_301727" totalcount="4"
value="CACTAATTGACCTCCCCACCCCCTCCAACATCTCTGCTTGATGAAATTTCG
GGTCTCTATTAGGACTATGTTTAATCTCCCAAATCCTAACAGGCTTATTTCTTTCC
ATACACTACACTGCCGATATCAACACAGCTTTTCATCCATCGCCCACATCTACCG
AGACGTAAACTACGGATGGCTAATCCGAAATTTACATGCCAACGGAGCATCCTTT
TTTTTCATTTGTCTCTATTTACACATTGCACGAGGCCTCTATTACGGCTCATTTCTC
TACAAAGAAACATGAAACATCGGGGTAATCCTTCTACTATTAGTAATAATAACCG
CTTTTGTAGGCTATGTACTCCCATGAGGACAAATATCGTTTTGAGGGGCTACAGT
CATTACCAACCTACTTTCCGCTGTTCCTTACATTGGAAACTCACTAGTTCAATGAA
TCTGAGGTGGCTTCTCAGTCGACAATGCCACCCTCACACGATTCTTCGCTATCCAT
TTTCTGCTTCCATTTGTTATCGCAGCCCTAACCCTAATTCACCTAATTTTTCTTCAT
GAGACAGGATCCACCAACCCAATTGGACTAAGCCCAAACACAGACAAAATCTCC
TTCCACCCATACTTCTCGTACAAAGACCTTCTCGGCTTCTTAATCCTACTTTTAAC
TTTAATGTTTGGAGCCTGAGCTGGTATAGCAGGAACCGCACTAAGCATGCTAATT
CGAGCTGAACTAACTCAGCCCGGTTCCTTTTTTGGGGACGACCAAATCTATAATG
TAATTGTTACTGCACATGCCTTCGTAATAATTTTTTTTATAGTGATGCCAATTATG
ATCGGCGGATTTGGTAATTGATTAATTCCACTAATAATTGGTGCCCCAGATATGG
CTTTCCCTCGCATGAATAATATAAGCTTTTGACTTCTACCCCCCTCCTTCCTCCTCC
TCCTTGCCTCCTCCACTGTTGAAGCTGGGGTGGGAACAGGCTGAACTGTGTACCC
CCCCCTCTCAGGAAATTTAGCTCACGACGGCCCATCTGTAGACCTAGCCATCTTCT
CCCTTCACCTAGCAGGGGTATCTTCAATTTTGGGGGCAATTAATTTTATCACCACT
ATTATCAACATAAAACCTCCAACCACCTCCCTGTATAATGCACCATTATTTATTTG
ATCTCTCCTTGTCACGGCTGTTCTTTTACTTCTCTCTCTACCAGTCCTTGCTGCAGG
TATCACCATACTTCTAACAGATCGAAACCT"/>

<sequence id="seq_Amor_Mito_Apisto_301730"
taxon="Amor_Mito_Apisto_301730" totalcount="4"
value="CACTAATTGACCTCCCCACCCCCTCCAACATCTCTGCTTGATGAAATTTCG
GGTCTCTATTAGGACTATGTTTAATCTCCCAAATCCTAACAGGCTTATTTCTTTCC
ATACACTACACTGCCGATATCAACACAGCTTTTTCATCCATCACCCACATCTGCCG
AGACGTAAACTACGGATGGCTAATCCGAAATTTACATGCCAACGGAGCATCCTTT
TTTTTCATTTGTCTCTATTTACACATTGCACGAGGCCTCTATTACGGCTCATTTCTC
TACAAAGAAACATGAAGCATCGGGGTAATCCTTCTACTATTAGTAATAATAACCG
CTTTTGTAGGCTATGTACTCCCATGAGGACAAATATCGTTTTGAGGGGCTACAGT
CATTACCAACCTACTTTCCGCTGTTCCCTACATTGGAAACTCACTAGTTCAATGAA
TCTGAGGTGGCTTCTCAGTCGACAATGCCACCCTCACACGATTCTTCGCTATCCAT
TTTCTGCTTCCATTTGTTATCGCAGCCCTAACCCTAATTCACCTAATTTTTCTTCAT
GAGACAGGATCCACCAACCCAATTGGACTAAGCCCAAACACAGACAAAATCTCC
TTCCACCCATACTTCTCGTACAAAGACCTTCTCGGCTTCTTAATCCTACTTTTAAC
TTTAATGTTTGGTGCCTGAGCTGGTATAGCAGGAACCGCACTAAGCATGCTAATT
CGAGCTGAACTAACTCAGCCCGGTTCCTTTTTTGGGGACGACCAAATCTATAATG
TGATTGTTACTGCACATGCCTTCGTAATAATTTTTTTTATAGTGATGCCAATTATG
ATCGGCGGATTTGGTAATTGATTAATTCCACTAATAATTGGTGCCCCAGATATGG
CTTTCCCTCGCATGAATAATATAAGCTTTTGACTACTACCCCCCTCCTTCCTCCTC
CTCCTTGCCTCCTCCACTGTTGAAGCTGGGGTGGGAACAGGCTGAACTGTGTACC
CCCCCCTCTCAGGAAATTTAGCTCACGACGGCCCATCTGTAGACCTAGCCATCTT
CTCCCTTCACCTAGCAGGGGTATCTTCAATTTTGGGGGCAATTAATTTTATCACCA
CTATTATCAACATAAAACCTCCAACCACCTCCCTGTATAATGCACCATTATTTATT
TGATCTCTCCTTGTCACGGCTGTTCTTTTACTTCTCTCTCTACCAGTCCTTGCTGCA
GGTATCACCATACTTCTAACAGATCGAAACCT"/>
<sequence id="seq_Amor_Mito_Apisto_301792"
taxon="Amor_Mito_Apisto_301792" totalcount="4"
value="CAATAATTGACCTCCCCACCCCCTCCAACATCTCTGCTTGATGAAATTTCG
GGTCTCTATTAGGACTATGTTTAATCTCCCAAATCCTAACAGGCTTATTTCTTTCC
ATACACTACACTGCCGATATCAACACAGCTTTTTCATCCATCACCCACATCTGCCG
AGACGTAAACTACGGATGGCTAATCCGAAATTTACATGCCAACGGAGCATCCTTT
TTTTTCATTTGTCTCTATTTACACATTGCACGAGGCCTCTATTACGGCTCATTTCTC
TACAAAGAAACATGAAGCATCGGGGTAATCCTTCTACTATTAGTAATAATAACCG
CTTTTGTAGGCTATGTACTCCCATGAGGACAAATATCGTTTTGAGGGGCTACAGT
CATTACCAACCTACTTTCCGCTGTTCCCTACATTGGAAACTCACTAGTTCAATGAA
TCTGAGGTGGCTTCTCAGTCGACAATGCCACCCTCACACGATTCTTCGCTATCCAT
TTTCTGCTTCCATTTGTTATCGCAGCCCTAACCCTAATTCACCTAATTTTTCTTCAT
GAGACAGGATCCACCAACCCAATTGGACTAAGCCCAAACACAGACAAAATCTCC
TTCCACCCATACTTCTCGTACAAAGACCTTCTCGGCTTCTTAATCCTACTTTTAAC
TTTAATGTTTGGTGCCTGAGCTGGTATAGCAGGAACCGCACTAAGCATGCTAATT
CGAGCTGAACTAACTCAGCCCGGTTCCTTTTTTGGGGACGACCAAATCTATAATG
TGATTGTTACTGCACATGCCTTCGTAATAATTTTTTTTATAGTGATGCCAATTATG
ATCGGCGGATTTGGTAATTGATTAATTCCACTAATAATTGGTGCCCCAGATATGG
CTTTCCCTCGCATGAATAATATAAGCTTTTGACTACTACCCCCCTCCTTCCTCCTC
CTCCTTGCCTCCTCCACTGTTGAAGCTGGGGTGGGAACAGGCTGAACTGTGTACC
CCCCCCTCTCAGGAAATTTAGCTCACGACGGCCCATCTGTAGACCTAGCCATCTT
CTCCCTTCACCTAGCAGGGGTATCTTCAATTTTGGGGGCAATTAATTTTATCACCA
CTATTATCAACATAAAACCTCCAACCACCTCCCTGTATAATGCACCATTATTTATT
TGATCTCTCCTTGTCACGGCTGTTCTTTTACTTCTCTCTCTACCAGTCCTTGCTGCA
GGTATCACCATACTTCTAACAGATCGAAACCT"/>

<sequence id="seq_Amor_Mito_Apisto_301599"
taxon="Amor_Mito_Apisto_301599" totalcount="4"
value="CACTAATTGACCTCCCCACCCCCTCCAACATCTCTGCTTGATGAAATTTCG
GGTCTCTATTAGGACTATGTTTAATCTCCCAAATCCTAACAGGCTTATTTCTTTCC
ATACACTACACTGCCGATATCAACACAGCTTTTTCATCCATCACCCACATCTGCCG
AGACGTAAACTACGGATGGCTAATCCGAAATTTACATGCCAACGGAGCATCCTTT
TTTTTCATTTGTCTCTATTTACACATTGCACGAGGCCTCTATTACGGCTCATTTCTC
TACAAAGAAACATGAAGCATCGGGGTAATCCTTCTACTATTAGTAATAATAACCG
CTTTTGTAGGCTATGTACTCCCATGAGGGCAAATATCGTTTTGAGGGGCTACAGT
CATTACCAACCTACTTTCCGCTGTTCCCTACATTGGAAACTCACTAGTTCAATGAA
TCTGAGGTGGCTTCTCAGTCGACAATGCCACCCTCACACGATTCTTCGCTATCCAT
TTTCTGCTTCCATTTGTTATCGCAGCCCTAACCCTAATTCACCTAATTTTTCTTCAT
GAGACAGGATCCACCAACCCAATTGGACTAAGCCCAAACACAGACAAAATCTCC
TTCCACCCATACTTCTCGTACAAAGACCTTCTCGGCTTCTTAATCCTACTTTTAAC
TTTAATGTTTGGTGCCTGAGCTGGTATAGCAGGAACCGCACTAAGCATGCTAATT
CGAGCTGAACTAACTCAGCCCGGTTCCTTTTTTGGGGACGACCAAATCTATAATG
TGATTGTTACTGCACATGCCTTCGTAATAATTTTTTTTATAGTGATGCCAATTATG
ATCGGCGGATTTGGTAATTGATTAATTCCACTAATAATTGGTGCCCCAGATATGG
CTTTCCCTCGCATGAATAATATAAGCTTTTGACTACTACCCCCCTCCTTCCTCCTC
CTCCTTGCCTCCTCCACTGTTGAAGCTGGGGTGGGAACAGGCTGAACTGTGTACC
CCCCCCTCTCAGGAAATTTAGCTCACGACGGCCCATCTGTAGACCTAGCCATCTT
CTCCCTTCACCTAGCAGGGGTATCTTCAATTTTGGGGGCAATTAATTTTATCACCA
CTATTATCAACATAAAACCTCCAACCACCTCCCTGTATAATGCACCATTATTTATT
TGATCTCTCCTTGTCACGGCTGTTCTTTTACTTCTCTCTCTACCAGTCCTTGCTGCA
GGTATCACCATACTTCTAACAGATCGAAACCT"/>
<sequence id="seq_Amor_Mito_Apisto_301600"
taxon="Amor_Mito_Apisto_301600" totalcount="4"
value="CACTAATTGACCTCCCCACCCCCTCCAACATCTCTGCTTGATGAAATTTCG
GGTCTCTATTAGGACTATGTTTAATCTCCCAAATCCTAACAGGCTTATTTCTTTCC
ATACACTACACTGCCGATATCAACACAGCTTTTTCATCCATCACCCACATCTGCCG
AGACGTAAACTACGGATGGCTAATCCGAAATTTACATGCCAACGGAGCATCCTTT
TTTTTCATTTGTCTCTATTTACACATTGCACGAGGCCTCTATTACGGCTCATTTCTC
TACAAAGAAACATGAAGCATCGGGGTAATCCTTCTACTATTAGTAATAATAACCG
CTTTTGTAGGCTATGTACTCCCATGAGGGCAAATATCGTTTTGAGGGGCTACAGT
CATTACCAACCTACTTTCCGCTGTTCCCTACATTGGAAACTCACTAGTTCAATGAA
TCTGAGGTGGCTTCTCAGTCGACAATGCCACCCTCACACGATTCTTCGCTATCCAT
TTTCTGCTTCCATTTGTTATCGCAGCCCTAACCCTAATTCACCTAATTTTTCTTCAT
GAGACAGGATCCACCAACCCAATTGGACTAAGCCCAAACACAGACAAAATCTCC
TTCCACCCATACTTCTCGTACAAAGACCTTCTCGGCTTCTTAATCCTACTTTTAAC
TTTAATGTTTGGTGCCTGAGCTGGTATAGCAGGAACCGCACTAAGCATGCTAATT
CGAGCTGAACTAACTCAGCCCGGTTCCTTTTTTGGGGACGACCAAATCTATAATG
TGATTGTTACTGCACATGCCTTCGTAATAATTTTTTTTATAGTGATGCCAATTATG
ATCGGCGGATTTGGTAATTGATTAATTCCACTAATAATTGGTGCCCCAGATATGG
CTTTCCCTCGCATGAATAATATAAGCTTTTGACTACTACCCCCCTCCTTCCTCCTC
CTCCTTGCCTCCTCCACTGTTGAAGCTGGGGTGGGAACAGGCTGAACTGTGTACC
CCCCCCTCTCAGGAAATTTAGCTCACGACGGCCCATCTGTAGACCTAGCCATCTT
CTCCCTTCACCTAGCAGGGGTATCTTCAATTTTGGGGGCAATTAATTTTATCACCA
CTATTATCAACATAAAACCTCCAACCACCTCCCTGTATAATGCACCATTATTTATT
TGATCTCTCCTTGTCACGGCTGTTCTTTTACTTCTCTCTCTACCAGTCCTTGCTGCA
GGTATCACCATACTTCTAACAGATCGAAACCA"/>

<sequence id="seq_Anij_Mito_Apisto_300304"
taxon="Anij_Mito_Apisto_300304" totalcount="4"
value="CACTAATTGACCTTCCCGCCCCCTCCAACATCTCCTCTTGATGAAATTTCG
GGTCCCTACTAGGCCTCTGCTTAATTTCCCAAATCCTAACAGGCTTATTTCTTTCC
ATACATTACACTGCTAACATCGACACAGCTTTCTCATCCGTTGCCCACATCTGCCG
AGATGTAAACTACGGATGACTAATTCGAAATCTACACGCTAACGGAGCATCTTTC
TTCTTTATTTGCATCTACCTGCACATCGGACGGGGCCTTTACTTCGGCTCTTACCT
TTACAAAGAAACATGAAACATTGGGGTGGTACTCCTACTGCTAGTTATAATAACC
GCTTTTGTAGGCTATGTCCTCCCATGAGGACAAATATCTTTTTGAGGGGCCACCGT
CATTACCAACTTACTATCAGCCATCCCCTACATCGGGGACTTCCTAGTCCAATGA
ATTTGAGGCGGCTTCTCAGTCGACAACGCTACCCTGACCCGATTCTTTGCCATCCA
CTTCCTCCTCCCATTTGTCATCACAGCCATAACCTTAATACACCTAATTTTCCTCC
ACGAAACAGGCTCTACAAACCCCATCGGACTAAACCCAAACACAGATAAAATCT
CTTTCCACCCATTCTACTCCCTCAAAGACCTCCTTGGCTTCCTAATTTTACTCACA
GCTTTTATATTTGGTGCGCCCCTTTTATTATTATTTGGCGCCCAGCTTGGT---AAC--
-CCCCTCATAACACATGCT---
TTTTTTTTCGGAGACGATCAAGCCTATAATGTAGTCGAAACCGCACACGCCTTCG
TAATAATCTTCTTTATAGTTATACCAATTATGATTGGTGGGTTTGGTAATTGACTA
ATCCCGCTAATAATCGGCGCCCCGGACATGGCTTTCCCTCGAATAAATAAAATAA
GCTTTTGACTACTCCCCCCATCTTTCCTCCTCCTCCTAGCCTCCTCTACTGTTGAAG
CTGGTGTAGGAACGGGCTGAACTGTATACCCACCCCTTTCCGGAAATTTAGCCCA
CGACGGCCCATCCGTGGACCTGGCCATTTTCTCCCTTCATTTAGCAGGAATCTCCT
CCATTCTTGGTGCAATCAACTTTATCACCACCATCATTAACATGAAACCTCCAGCC
ATCTCCATATACACTACACCCCTGTTTATCTGGTCCCTCCTCATTACTGCTGTACTT
CTACTTCTCTCACTACCAGTTCTTGCTGCTGGCATCACCATACTTCTCACAGACCG
TAACTT"/>
<sequence id="seq_Anij_Mito_Apisto_300306"
taxon="Anij_Mito_Apisto_300306" totalcount="4"
value="CACTAATTGACCTTCCCGCCCCCTCCAACATCTCCTCTTGATGAAATTTCG
GGTCCCTACTAGGCCTCTGCTTAATTTCCCAAATCCTAACAGGCTTATTTCTTTCC
ATACATTACACTGCTAACATCGACACAGCTTTCTCATCCGTTGCCCACATCTGCCG
AGATGTAAACTACGGATGACTAATTCGAAATCTACACGCTAACGGAGCATCTTTC
TTCTTTATTTGCATCTACCTGCACATCGGACGGGGCCTTTACTTCGGCTCTTACCT
TTACAAAGAAACATGAAACATTGGGGTGGTACTCCTACTGCTAGTTATAATAACC
GCTTTTGTAGGCTATGTCCTCCCATGAGGACAAATATCTTTTTGAGGGGCCACCGT
CATTACCAACTTACTATCAGCCATCCCCTACATCGGGGACTTCCTAGTCCAATGA
ATTTGAGGCGGCTTCTCAGTCGACAACGCTACCCTGACCCGATTCTTTGCCATCCA
CTTCCTCCTCCCATTTGTCATCACAGCCATAACCTTAATACACCTAATTTTCCTCC
ACGAAACAGGCTCTACAAACCCCATCGGACTAAACCCAAACACAGATAAAATCT
CTTTCCACCCATTCTACTCCCTCAAAGACCTCCTTGGCTTCCTAATTTTACTCACA
GCTTTTATATTTGGTGCGCCCCTTTTATTATTATTTGGCGCCCAGCTTGGT---AAC--
-CCCCTCATAACACATGCT---
TTTTTTTTCGGAGACGATCAAGCCTATAATGTAGTCGTAACTGCACACGCCTTCGT
AATAATCTTCTTTATAGTTATACCAATTATGATTGGTGGGTTTGGTAATTGACTAA
TCCCGCTAATAATCGGCGCCCCGGACATGGCTTTCCCTCGAATAAATAAAATAAG
CTTTTGACTACTCCCCCCATCTTTCCTCCTCCTCCTAGCCTCCTCAACTGTTGAAGC
TGGTGTAGGAACGGGCTGAACTGTATACCCACCCCTTTCCGGAAATTTAGCCCAC
GACGGCCCATCCGTGGACCTGGCCATTTTCTCCCTTCATTTAGCAGGAATCTCCTC
CATTCTAGGTGCAATCAACTTTATCACCACCATCATTAACATGAAACCTCCAGCC
ATCTCCATATACACTACACCCCTGTTTATCTGGTCCCTCCTCATTACTGCTGTACTT

CTACTTCTCTCACTACCAGTTCTTGCTGCTGGCATCACCATACTTCTCACAGACCG
TAACTT"/>
<sequence id="seq_Anij_Mito_Apisto_300328"
taxon="Anij_Mito_Apisto_300328" totalcount="4"
value="CACTAATTGACCTTCCCGCCCCCTCCAACATCTCCTCTTGATGAAATTTCG
GGTCCCTACTAGGCCTCTGCTTAATTTCCCAAATCCTAACAGGCTTATTTCTTTCC
ATACATTACACTGCTAACATCGACCCAGCTTTCTCATCCGTTGCCCACATCTGCCG
AGATGTAAACTACGGATGACTAATTCGAAATCTACACGCTAACGGAGCATCTTTC
TTCTTTATTTGCATCTACCTGCACATCGGACGGGGCCTTTACTTCGGCTCTTACCT
TTACAAAGAAACATGAAACATTGGGGTGGTACTCCTACTGCTAGTTATAATAACC
GCTTTTGTAGGCTATGTCCTCCCATGAGGACAAATATCTTTTTGAGGGGCCACCGT
CATTACCAACTTACTATCAGCCATCCCCTACATCGGGGACTTCCTAGTCCAATGA
ATTTGAGGCGGCTTCTCAGTCGACAACGCTACCCTGACCCGATTCTTTGCCATCCA
CTTCCTCCTCCCATTTGTCATCACAGCCATAACCTTAATACACCTAATTTTCCTCC
ACGAAACAGGCTCTACAAACCCCATCGGACTAAACCCAAACACAGATAAAATCT
CTTTCCACCCATTCTACTCCCTCAAAGACCTCCTTGGCTTCCTAATTTTACTCACA
GCTTTTATATTTGGTGCGCCCCTTTTATTATTATTTGGCGCCCAGCTTGGT---AAC--
-CCCCTCATAACACATGCT---
TTTTTTTTCGGAGACGATCAAGCCTATAATGTAGTCGTAACTGCACACGCCTTCGT
AATAATCTTCTTTATAGTTATACCAATTATGATTGGTGGGTTTGGTAATTGACTAA
TCCCGCTAATAATCGGCGCCCCGGACATGGCTTTCCCTCGAATAAATAAAATAAG
CTTTTGACTACTCCCCCCATCTTTCCTCCTCCTCCTAGCCTCCTCAACTGTTGAAGC
TGGTGTAGGAACGGGCTGAACTGTATACCCACCCCTTTCCGGAAATTTAGCCCAC
GACGGCCCATCCGTGGACCTGGCCATTTTCTCCCTTCATTTAGCAGGAATCTCCTC
CATTCTAGGTGCAATCAACTTTATCACCACCATCATTAACATGAAACCTCCAGCC
ATCTCCATATACACTACACCCCTGTTTATCTGGTCCCTCCTCATTACTGCTGTACTT
CTACTTCTCTCACTACCAGTTCTTGCTGCTGGCATCACCATACTTCTCACAGACCG
TAACTT"/>
<sequence id="seq_Aore_Mito_Apisto_302891"
taxon="Aore_Mito_Apisto_302891" totalcount="4"
value="CACTAATTGATCTTCCCGCTCCCTCCAACATCTCCTCTTGATGAAATTTTGG
GTCCCTATTGGGCCTCTGCTTAATTTCCCAAATCCTAACAGGCTTATTTCTTTCCA
TACATTATACTGCTGACATCAACACAGCTTTCTCATCCATTACCCACATCTGCCGA
GATGTAAATTACGGATGACTAATTCGAAATCTACATGCTAACGGAGCATCCTTTT
TCTTCATTTGCATCTACCTGCACATCGGACGAGGCCTTTACTTCGGCTCTTACCTC
TACAAAGAAACATGAAACATTGGGGTGGTACTCCTGCTGCTAGTAATAATAACCG
CTTTTGTAGGTTACGTCCTCCCGTGAGGACAAATATCGTTTTGAGGGGCTACCGTC
ATCACCAACTTGCTGTCGGCGGTCCCCTACATTGGAGACTCCCTGGTCCAATGAA
TTTGAGGCGGCTTCTCAGTTGACAACGCTACCCTAACTCGATTCTTTGCCATCCAC
TTCCTTCTTCCGTTCATCATCGCAGCCTTAACCTTAATACACCTAATTTTCCTCCAC
GAGACAGGTTCCACAAACCCAATCGGACTAAGCCCAAATACAGATAAAATTTCTT
TCCACCCATTCTACTCCCTCAAAGACCTCCTCGGCTTCCTAATTTTACTTACAACT
CTATTATTTGGTGCcTGAGCCGGAATGGCAGGTACCGCaCTAAGCATGcTAATTCG
AGCAGAACTTACTCAACCCGGCTCCCTTTTTGGAGACGaCCAAGCcTATAATGTAA
TCGTAACTGCACACGCCTTCGTAATAATTTTCTTCATAGTTATACCAATTAtGATC
GGCGGGtTTGGTAATTGACTAATCCCGCTAATAATCGGCGCCCCGGATATGGCTTT
CCCCCGTATAAATAAcAtAagCTTTTGACTACTCCCCCCGTCTTTCCTCCTCCTCCTA
gCCTCCTCGACTGTTGAAGCTGGTGTGGGGACaGGCTGAACTGTATACCCGCCCCT
TTCCGGAAATTTAGCCCACGACGGCCCATCCGTGGACCTGGCCATTTTCTCCCTTC
ATTTAACAGGGgTCTCCTCCATTCTAGGTGCAATCAACTTTATCACCACAATCATT

AACATAAAACCCCCAGCCATTTCCATGTACACCACACCCCTATTCATCTGATCTCT
CCTCATTACTGCTGTGCTCCTACTTCTCTCACTACCAGTACTTGCTGCTGGAATTA
CCATGCTTCTCACAGACCGaAACCT"/>
<sequence id="seq_Aore_Mito_Apisto_302892"
taxon="Aore_Mito_Apisto_302892" totalcount="4"
value="CACTAATTGATCTTCCCGCTCCCTCCAACATCTCCTCTTGATGAAATTTTGG
GTCCCTATTGGGCCTCTGCTTAATTTCCCAAATCCTAACAGGCTTATTTCTTTCCA
TACATTATACTGCTGACATCAACACAGCTTTCTCATCCATTACCCACATCTGCCGA
GATGTAAATTACGGATGACTAATTCGAAATCTACATGCTAACGGAGCATCCTTTT
TCTTCATTTGCATCTACCTGCACATCGGACGAGGCCTTTACTTCGGCTCTTACCTC
TACAAAGAAACATGAAACATTGGGGTGGTACTCCTGCTGCTAGTAATAATAACCG
CTTTTGTAGGTTACGTCCTCCCGTGAGGACAAATATCGTTTTGAGGGGCTACCGTC
ATCACCAATTTGCTGTCGGCGGTCCCCTACATTGGAGACTCCCTGGTCCAATGAA
TTTGAGGCGGCTTCTCAGTTGACAACGCTACCCTAACTCGATTCTTTGCCATCCAC
TTCCTTCTTCCGTTCATCATCGCAGCCTTAACCTTAATACACCTAATTTTCCTCCAC
GAGACAGGTTCCACAAACCCAATCGGACTAAGCCCAAATACAGATAAAATTTCTT
TCCACCCATTCTACTCCCTCAAAGACCTCCTCGGCTTCCTAATTTTACTTACAACT
CTTATatTTGGtGCTTGAGCcGGAaTGGCAGGTACCGCACTAAGCATGCTAATTCGA
GCaGAACTTACTCAACCCGGcTCCcTTTTGGAGACGACCAAgcCTATAATGTAATc
GTAACTGCACAcGCCTTCGTAATAATTTTCTTCATAGTTATACCAATTATGATCGG
CGGGTTTGGTAATTGACTAATCCCGCTAATAATCGGCGCCCCGGATATGGCTTTC
CCCCGTATAAATAACATAAGCTTTTGACTACTCCCCCCGTCTTTCCTCCTCCTCCT
AgCCTCCTCgACTGTTGAAgCTGGTGTGGGGACAGGCTGAACTGTATACCCGCCCC
TTTCCGGAAATTTAGCCCACGACGGCCCATCCGTGGACCTGGCCATTTTCTCCCTT
CATTTAGCAGGGGTCTCCTCCATTCTAGGTGCAATCAACTTTATCACCACGATCAT
TAACATAAAACCCCCAgCCATTTCCATGTACACCAcACCCCTATTCATCTGATCCC
TCCTCAtTACTGCTGTGCTCCTACTTCTCTCACTACCAGTACTTGCTGCTGGAATTA
CCATGCTTCTCACAgACCGAAACCT"/>
<sequence id="seq_Aore_Mito_Apisto_302893"
taxon="Aore_Mito_Apisto_302893" totalcount="4"
value="CACTAATTGATCTTCCCGCTCCCTCCAACATCTCCTCTTGATGAAATTTTGG
GTCCCTATTGGGCCTCTGCTTAATTTCCCAAATCCTAACAGGCTTATTTCTTTCCA
TACATTATACTGCTGACATCAACACAGCTTTCTCATCCATTACCCACATCTGCCGA
GATGTAAATTACGGATGACTAATTCGAAATCTACATGCTAACGGAGCATCCTTTT
TCTTCATTTGCATCTACCTGCACATCGGACGAGGCCTTTACTTCGGCTCTTACCTC
TACAAAGAAACATGAAACATTGGGGTGGTACTCCTGCTGCTAGTAATAATAACCG
CTTTTGTAGGTTACGTCCTCCCGTGAGGACAAATATCGTTTTGAGGGGCTACCGTC
ATCACCAACTTGCTGTCGGCGGTCCCCTACATTGGAGACTCCCTGGTCCAATGAA
TTTGAGGCGGCTTCTCAGTTGACAACGCTACCCTAACTCGATTCTTTGCCATCCAC
TTCCTTCTTCCGTTCATCATCGCAGCCTTAACCTTAATACACCTAATTTTCCTCCAC
GAGACAGGTTCCACAAACCCAATCGGACTAAGCCCAAATACAGATAAAATTTCTT
TCCACCCATTCTACTCCCTCAAAGACCTCCTCGGCTTCCTAATTTTACTTACAACT
CTTAtaTTTGGTGCtTGAGCcGGAATGGCAGGTACCGCaCTAAGCATGCTAATTCGA
GCaGAACTTACTCAACCCGGcTCCcTTTTGGAGACGACCAAgcCTATAATGTAATC
GTAACTGCACACGCCTTCgTAATAATTTTCTTCATAGTTATACCAATTATGATCGG
CGGGtTTGGTAATTGACTAATCCCGCTAATAATCGGCGCCCCGGATATGGCTTTCC
CCCGTATAAATAACATAagCTTTTGACTACTCCCCCCGTCTTTCCTCCTCCTCCTAg
CCTCCTCgACTGTTGAAGCTGGTGtGGgGACAGGCTGAACTGTATACCCGCCCCTT
TCCGGAAATTTAgCCCACGACGGCCCATCCGTGGACCTGGCCATTTTCTCCCTTCA
TTTAgCAGGGgTCTCCTCCATTCTAGGTGCAATCAACTTTATCACCACgATCATTAA

CATAAAACCCCCAgCCATTTCCATGTACACCACACCCCTATTCATCTGATCCCTCC
TCATTACTGCTGTGCTCCTACTTCTCTCACTACCAGTACTTGCTGCTGGAATTACC
ATGCTTCTCACAGACCGaAACCT"/>
<sequence id="seq_Aore_Mito_Apisto_302894"
taxon="Aore_Mito_Apisto_302894" totalcount="4"
value="CACTAATTGATCTTCCCGCTCCCTCCAACATCTCCTCTTGATGAAATTTTGG
GTCCCTATTGGGCCTCTGCTTAATTTCCCAAATCCTAACAGGCTTATTTCTTTCCA
TACATTATACTGCTGACATCAACACAGCTTTCTCATCCATTACCCACATCTGCCGA
GATGTAAATTACGGATGACTAATTCGAAATCTACATGCTAACGGAGCATCCTTTT
TCTTCATTTGCATCTACCTGCACATCGGACGAGGCCTTTACTTCGGCTCTTACCTC
TACAAAGAAACATGAAACATTGGGGTGGTACTCCTGCTGCTAGTAATAATAACCG
CTTTTGTAGGTTACGTCCTCCCGTGAGGACAAATATCGTTTTGAGGGGCTACCGTC
ATCACCAACTTGCTGTCGGCGGTCCCCTACATTGGAGACTCCCTGGTCCAATGAA
TTTGAGGCGGCTTCTCAGTTGACAACGCTACCCTAACTCGATTCTTTGCCATCCAC
TTCCTTCTTCCGTTCATCATCGCAGCCTTAACCTTAATACACCTAATTTTCCTCCAC
GAGACAGGTTCCACAAACCCAATCGGACTAAGCCCAAATACAGATAAAATTTCTT
TCCACCCATTCTACTCCCTCAAAGACCTCCTCGGCTTCCTAATTTTACTTACAACT
CTTATATTTGGTGCtTGAGCcGGAATGGCAGGTACCGCACTAAGCATGCTAATTCG
AGCaGAACTTACTCAACCCGGCTCCcTTTTTGGAGACGACCAAGcCTATAATGTAA
TcGTaACTGCACAcGCCTTCGTAATAATTTTCTTCATAGTTATACCAATTATGATCG
GCGGGTTTGGTAATTGACTAATCCCGCTAATAATCGGCGCCCCGGATATGGCTTT
CCCCCGTATAAATAAcATAAGCTTTTGACTACTCCCCCCGTCTTTCCTCCTCCTCCT
AgcCTCCTCgACTGTTGAAGCTGGTGTGGGGACAGGCTGAACTGtATACCCGCCCC
TTTCCGGAAATTTAGCCCACGACGGCCCATCCGTGGACCTGGCCATTTTCTCCCTT
CATTTAgCAGGGgTCTCCTCCATTCTAGGTGCAATCAACTTTATCACCACGATCAT
TAACATAAAACCCCCAgCCATTTCCATGTACACCACACCCCTATTCATCTGATCCC
TCCTCAtTACTGCTGTGCTCCTACTTCTCTCACTACCAGTACTTGCTGCTGGAATTA
CCATGCTTCTCACAGACCGAAACCT"/>
<sequence id="seq_Aore_Mito_Apisto_302895"
taxon="Aore_Mito_Apisto_302895" totalcount="4"
value="CACTAATTGATCTTCCCGCTCCCTCCAACATCTCCTCTTGATGAAATTTTGG
GTCCCTATTGGGCCTCTGCTTAATTTCCCAAATCCTAACAGGCTTATTTCTTTCCA
TACATTATACTGCTGACATCAACACAGCTTTCTCATCCATTACCCACATCTGCCGA
GATGTAAATTACGGATGACTAATTCGAAATCTACATGCTAACGGAGCATCCTTTT
TCTTCATTTGCATCTACCTGCACATCGGACGAGGCCTTTACTTCGGCTCTTACCTC
TACAAAGAAACATGAAACATTGGGGTGGTACTCCTGCTGCTAGTAATAATAACCG
CTTTTGTAGGTTACGTCCTCCCGTGAGGACAAATATCGTTTTGAGGGGCTACCGTC
ATCACCAACTTGCTGTCGGCGGTCCCCTACATTGGAGACTCCCTGGTCCAATGAA
TTTGAGGCGGCTTCTCAGTTGACAACGCTACCCTAACTCGATTCTTTGCCATCCAC
TTCCTTCTTCCGTTCATCATCGCAGCCTTAACCTTAATACACCTAATTTTCCTCCAC
GAGACAGGTTCCACAAACCCAATCGGACTAAGCCCAAATACAGATAAAATTTCTT
TCCACCCATTCTACTCCCTCAAAGACCTCCTCGGCTTCCTAATTTTACTTACAACT
CTTATATTTGGTGCtTGAGCcGGAATGGCAGGTACCGCACTAAGCATGCTAATTCG
AGCaGAACTTACTCAACCCGGCTCCcTTTTTGGAGACGACCAAGcCTATAATGTAA
TcGTaACTGCACAcGCCTTCGTAATAATTTTCTTCATAGTTATACCAATTATGATCG
GCGGGTTTGGTAATTGACTAATCCCGCTAATAATCGGCGCCCCGGATATGGCTTT
CCCCCGTATAAATAACATAAGCTTTTGACTACTCCCCCCGTCTTTCCTCCTCCTCC
TAgcCTCCTCgACTGTTGAAGCTGGTGTGGGGACAGGCTGAACTGtATACCCGCCC
CTTTCCGGAAATTTAGCCCACGACGGCCCATCCGTGGACCTGGCCATTTTCTCCCT
TCATTTAgCAGGGGTCTCCTCCATTCTAGGTGCAATCAACTTTATCACCACGATCA

TTAACATAAAACCCCCAgCCATTTCCATGTACACCACACCCCTATTCATCTGATCC
CTCCTCATTACTGCTGtGCTCCTACTTCTCTCACTACCAGTACTTGCTGCTGGAATT
ACCATGCTTCTCACAgACCGAAACCT"/>
<sequence id="seq_Apap_Mito_Apisto_301773"
taxon="Apap_Mito_Apisto_301773" totalcount="4"
value="CACTAATTGACCTCCCCACCCCCTCCAACATCTCTGCTTGATGAAATTTCG
GGTCTCTATTAGGACTATGTTTAATTTCCCAAATCCTAACAGGCTTATTTCTTTCC
ATACACTACACTGCCGATATCAACACAGCTTTTTCATCCATCGCCCACATCTACCG
AGATGTAAACTACGGATGACTAATCCGAAATTTACATGCTAACGGAGCATCCTTT
TTTTTCATTTGTATCTATTTACACATTGCACGAGGCCTCTATTACGGCTCATTTCTC
TACAAAGAAACATGAAACATCGGAGTAATCCTTCTACTATTAGTAATAATAACCG
CTTTTGTAGGCTATGTACTCCCATGAGGACAAATATCGTTTTGAGGGGCTACAGT
CATTACCAACCTACTTTCCGCTGTTCCTTACATTGGAAACTCACTAGTTCAATGAA
TCTGAGGTGGCTTCTCAGTCGACAATGCCACCCTCACACGATTCTTCGCTATCCAT
TTTCTACTTCCATTTGTTATCGCAGCCCTAACCCTAATTCACCTAATTTTTCTTCAT
GAGACAGGATCCACCAACCCAATTGGACTAAGCCCAAACACAGACAAAATCTCC
TTCCACCCATACTTCTCGTACAAAGACCTTCTCGGCTTCTTAATCCTACTTTTAAC
TTTAATGTTTGGTGCCTGAGCTGGTATGGCAGGAACCGCACTAAGCATGCTAATT
CGAGCTGAGCTAACTCAGCCCGGTTCCTTTTTTGGGGACGACCAAATCTATAATG
TAATTGTTACTGCACATGCCTTCGTAATAATTTTTTTTATAGTGATACCAATTATG
ATCGGCGGATTTGGTAATTGATTAATTCCACTAATAATTGGTGCCCCAGATATGG
CTTTCCCTCGCATGAATAATATAAGCTTTTGACTACTACCCCCCTCCTTCCTCCTC
CTCCTTGCCTCCTCCACTGTTGAAGCTGGGGTGGGAACAGGCTGAACTGTGTACC
CCCCCCTCTCAGGAAATTTAGCTCACGACGGCCCATCTGTAGACCTAGCCATCTT
CTCCCTTCACCTAGCAGGGGTATCTTCAATTTTGGGGGCCATTAATTTTATCACCA
CTATTATCAACATAAAACCTCCAACCACCTCCCTGTATAATGCACCACTATTTATT
TGATCTCTCCTTGTCACGGCTGTTCTTTACTTCTCTCTCTACCAGTCCTTGCTGCG
GGTATCACCATACTTCTAACAGATCGAAACCT"/>
<sequence id="seq_Apap_Mito_Apisto_301774"
taxon="Apap_Mito_Apisto_301774" totalcount="4"
value="CACTAATTGACCTCCCCACCCCCTCCAACATCTCTGCTTGATGAAATTTCG
GGTCTCTATTAGGACTATGTTTAATTTCCCAAATCCTAACAGGCTTATTTCTTTCC
ATACACTACACTGCCGATATCAACACAGCTTTTTCATCCATCGCCCACATCTACCG
AGATGTAAACTACGGATGACTAATCCGAAATTTACATGCTAACGGAGCATCCTTT
TTTTTCATTTGTATCTATTTACACATTGCACGAGGCCTCTATTACGGCTCATTTCTC
TACAAAGAAACATGAAACATCGGAGTAATCCTTCTACTATTAGTAATAATAACCG
CTTTTGTAGGCTATGTACTCCCATGAGGACAAATATCGTTTTGAGGGGCTACAGT
CATTACCAACCTACTTTCCGCTGTTCCTTACATTGGAAACTCACTAGTTCAATGAA
TCTGAGGTGGCTTCTCAGTCGACAATGCCACCCTCACACGATTCTTCGCTATCCAT
TTTCTACTTCCATTTGTTATCGCAGCCCTAACCCTAATTCACCTAATTTTTCTTCAT
GAGACAGGATCCACCAACCCAATTGGACTAAGCCCAAACACAGACAAAATCTCC
TTCCACCCATACTTCTCGTACAAAGACCTTCTCGGCTTCTTAATCCTACTTTTAAC
TTTAATGTTTGGTGCCTGAGCTGGTATGGCAGGAACCGCACTAAGCATGCTAATT
CGAGCTGAGCTAACTCAGCCCGGTTCCTTTTTTGGGGACGACCAAATCTATAATG
TAATTGTTACTGCACATGCCTTCGTAATAATTTTTTTTATAGTGATACCAATTATG
ATCGGCGGATTTGGTAATTGATTAATTCCACTAATAATTGGTGCCCCAGATATGG
CTTTCCCTCGCATGAATAATATAAGCTTTTGACTACTACCCCCCTCCTTCCTCCTC
CTCCTTGCCTCCTCCACTGTTGAAGCTGGGGTGGGAACAGGCTGAACTGTGTACC
CCCCCCTCTCAGGAAATTTAGCTCACGACGGCCCATCTGTAGACCTAGCCATCTT
CTCCCTTCACCTAGCAGGGGTATCTTCAATTTTGGGGGCCATTAATTTTATCACCA

CTATTATCAACATAAAACCTCCAACCACCTCCCTGTATAATGCACCACTATTTATT
TGATCTCTCCTTGTCACGGCTGTTCTTTACTTCTCTCTCTACCAGTCCTTGCTGCG
GGTATCACCATACTTCTAACAGATCGAAACCT"/>
<sequence id="seq_Apap_Mito_Apisto_301766"
taxon="Apap_Mito_Apisto_301766" totalcount="4"
value="CACTAATTGACCTCCCCACCCCCTCCAACATCTCTGCTTGATGAAATTTCG
GGTCTCTATTAGGACTATGTTTAATTTCCCAAATCCTAACAGGCTTATTTCTTTCC
ATACACTACACTGCCGATATCAACACAGCTTTTTCATCCATCGCCCACATCTACCG
AGATGTAAACTACGGATGACTAATCCGAAATTTACATGCTAACGGAGCATCCTTT
TTTTTCATTTGTATCTATTTACACATTGCACGAGGCCTCTATTACGGCTCATTTCTC
TACAAAGAAACATGAAACATCGGAGTAATCCTTCTACTATTAGTAATAATAACCG
CTTTTGTAGGCTATGTACTCCCATGAGGACAAATATCGTTTTGAGGGGCTACAGT
CATTACCAACCTACTTTCCGCTGTTCCTTACATTGGAAACTCACTAGTTCAATGAA
TCTGAGGTGGCTTCTCAGTCGACAATGCCACCCTCACACGATTCTTCGCTATCCAT
TTTCTGCTTCCATTTGTTATCGCAGCCCTAACCCTAATTCACCTAATTTTTCTTCAT
GAGACAGGATCCACCAACCCAATTGGACTAAGCCCAAACACAGACAAAATCTCC
TTCCACCCATACTTCTCGTACAAAGACCTTCTCGGCTTCTTAATCCTACTTTTAAC
TTTAATGTTTGGTGCCTGAGCTGGTATGGCAGGAACCGCACTAAGCATGCTAATT
CGAGCTGAGCTAACTCAGCCCGGTTCCTTTTTTGGGGACGACCAAATCTATAATG
TAATTGTTACTGCACATGCCTTCGTAATAATTTTTTTTATAGTGATACCAATTATG
ATCGGCGGATTTGGTAATTGATTAATTCCACTAATAATTGGTGCCCCAGATATGG
CTTTCCCTCGCATGAATAATATAAGCTTTTGACTACTACCCCCCTCCTTCCTCCTC
CTCCTTGCCTCCTCCACTGTTGAAGCTGGGGTGGGAACAGGCTGAACTGTGTACC
CCCCCCTCTCAGGAAATTTAGCTCACGACGGCCCATCTGTAGACCTAGCCATCTT
CTCCCTTCACCTAGCAGGGGTATCTTCAATTTTGGGGGCCATTAATTTTATCACCA
CTATTATCAACATAAAACCTCCAACCACCTCCCTGTATAATGCACCACTATTTATT
TGATCTCTCCTTGTCACGGCTGTTCTTTACTTCTCTCTCTACCAGTCCTTGCTGCG
GGTATCACCATACTTCTAACAGATCGAAACCT"/>
<sequence id="seq_Apap_Mito_Apisto_301768"
taxon="Apap_Mito_Apisto_301768" totalcount="4"
value="CACTAATTGACCTCCCCACCCCCTCCAACATCTCTGCTTGATGAAATTTCG
GGTCTCTATTAGGACTATGTTTAATTTCCCAAATCCTAACAGGCTTATTTCTTTCC
ATACACTACACTGCCGATATCAACACAGCTTTTTCATCCATCGCCCACATCTACCG
AGATGTAAACTACGGATGACTAATCCGAAATTTACATGCTAACGGAGCATCCTTT
TTTTTCATTTGTATCTATTTACACATTGCACGAGGCCTCTATTACGGCTCATTTCTC
TACAAAGAAACATGAAACATCGGAGTAATCCTTCTACTATTAGTAATAATAACCG
CTTTTGTAGGCTATGTACTCCCATGAGGACAAATATCGTTTTGAGGGGCTACAGT
CATTACCAACCTACTTTCCGCTGTTCCTTACATTGGAAACTCACTAGTTCAATGAA
TCTGAGGTGGCTTCTCAGTCGACAATGCCACCCTCACACGATTCTTCGCTATCCAT
TTTCTACTTCCATTTGTTATCGCAGCCCTAACCCTAATTCACCTAATTTTTCTTCAT
GAGACAGGATCCACCAACCCAATTGGACTAAGCCCAAACACAGACAAAATCTCC
TTCCACCCATACTTCTCGTACAAAGACCTTCTCGGCTTCTTAATCCTACTTTTAAC
TTTAATGTTTGGTGCCTGAGCTGGTATGGCAGGAACCGCACTAAGCATGCTAATT
CGAGCTGAGCTAACTCAGCCCGGTTCCTTTTTTGGGGACGACCAAATCTATAATG
TAATTGTTACTGCACATGCCTTCGTAATAATTTTTTTTATAGTGATACCAATTATG
ATCGGCGGATTTGGTAATTGATTAATTCCACTAATAATTGGTGCCCCAGATATGG
CTTTCCCTCGCATGAATAATATAAGCTTTTGACTACTACCCCCCTCCTTCCTCCTC
CTCCTTGCCTCCTCCACTGTTGAAGCTGGGGTGGGAACAGGCTGAACTGTGTACC
CCCCCCTCTCAGGAAATTTAGCTCACGACGGCCCATCTGTAGACCTAGCCATCTT
CTCCCTTCACCTAGCAGGGGTATCTTCAATTTTGGGGGCCATTAATTTTATCACCA

CTATTATCAACATAAAACCTCCAACCACCTCCCTGTATAATGCACCACTATTTATT
TGATCTCTCCTTGTCACGGCTGTTCTTTACTTCTCTCTCTACCAGTCCTTGCTGCG
GGTATCACCATACTTCTAACAGATCGAAACCT"/>
<sequence id="seq_Apap_Mito_Apisto_301769"
taxon="Apap_Mito_Apisto_301769" totalcount="4"
value="CACTAATTGACCTCCCCACCCCCTCCAACATCTCTGCTTGATGAAATTTCG
GGTCTCTATTAGGACTATGTTTAATTTCCCAAATCCTAACAGGCTTATTTCTTTCC
ATACACTACACTGCCGATATCAACACAGCTTTTTCATCCATCGCCCACATCTACCG
AGATGTAAACTACGGATGACTAATCCGAAATTTACATGCTAACGGAGCATCCTTT
TTTTTCATTTGTATCTATTTACACATTGCACGAGGCCTCTATTACGGCTCATTTCTC
TACAAAGAAACATGAAACATCGGAGTAATCCTTCTACTATTAGTAATAATAACCG
CTTTTGTAGGCTATGTACTCCCATGAGGACAGATATCGTTTTGAGGGGCTACAGT
CATTACCAACCTACTTTCCGCTGTTCCTTACATTGGAAACTCACTAGTTCAATGAA
TCTGAGGTGGCTTCTCAGTCGACAATGCCACCCTCACACGATTCTTCGCTATCCAT
TTTCTACTTCCATTTGTTATCTCAGCCCTAACCCTAATTCACCTAATTTTTCTTCAT
GAGACAGGATCCACCAACCCAATTGGACTAAGCCCAAACACAGACAAAATCTCC
TTCCACCCATACTTCTCGTACAAAGACCTTCTCGGCTTCTTAATCCTACTTTTAAC
TTTAATGTTTGGTGCCTGAGCTGGTATGGCAGGAACCGCACTAAGCATGCTAATT
CGAGCTGAGCTAACTCAGCCCGGTTCCTTTTTTGGGGACGACCAAATCTATAATG
TAATTGTTACTGCACATGCCTTCGTAATAATTTTTTTTATAGTGATACCAATTATG
ATCGGCGGATTTGGTAATTGATTAATTCCACTAATAATTGGTGCCCCAGATATGG
CTTTCCCTCGCATGAATAATATAAGCTTTTGACTACTACCCCCCTCCTTCCTCCTC
CTCCTTGCCTCCTCCACTGTTGAAGCTGGGGTGGGAACAGGCTGAACTGTGTACC
CCCCCCTCTCAGGAAATTTAGCTCACGACGGCCCATCTGTAGACCTAGCCATCTT
CTCCCTTCACCTGGCAGGGGTATCTTCAATTTTGGGGGCCATTAATTTTATCACCA
CTATTATCAACATAAAACCTCCAACCACCTCCCTGTATAATGCACCACTATTTATT
TGATCTCTCCTTGTCACGGCTGTTCTTTACTTCTCTCTCTACCAGTCCTTGCTGCG
GGTATCACCATACTTCTAACAGATCGAAACCT"/>
<sequence id="seq_Apap_Mito_Apisto_301770"
taxon="Apap_Mito_Apisto_301770" totalcount="4"
value="CACTAATTGACCTCCCCACCCCCTCCAACATCTTTGCTGGATGAAATTTCG
GGTCTGTATTAGGACTATGTTTAATTTCCCAAATCCTAACAGGCTTATTTCTTTCC
ATACACTACACTGCCGACATCAACACAGCTTTTTCATCCATCGCCCACATCTGCC
GAGACGTAAACTACGGATGGCTAATCCGAAATTTACATGCCAACGGAGCATCCTT
TTTTTTCATTTGTATCTATTTACACATTGCACGAGGCCTCTATTACGGCTCATTTCT
CTACAAAGAAACATGAAACATTGGGGTAATCCTTCTACTATTAGTAATAATAACC
GCTTTTGTAGGCTATGTACTCCCATGAGGACAAATATCGTTTTGAGGGGCTACAG
TCATTACCAACCTACTTTCCGCTGTTCCTTACATTGGAAACTCACTAGTTCAATGA
ATCTGAGGTGGCTTCTCAGTCGACAATGCCACCCTCACACGATTCTTCGCTATCCA
TTTTCTGCTTCCATTTGTTATCGCAGCCCTAACCCTAATTCACCTAATTTTTCTTCA
TGAGACAGGATCCACCAACCCAATTGGACTAAGCCCAAACACAGACAAAATCTC
CTTCCACCCATACTTCTCGTACAAAGACCTTCTCGGCTTCTTAATCCTACTTTTAA
CTTTAATGTTTGGTGCCTGAGCTGGTATGGCAGGAACCGCACTAAGCATGCTAAT
TCGAGCTGAGCTAACTCAGCCCGGTTCCTTTTTTGGGGACGACCAAATCTATAAT
GTAATTGTTACTGCACATGCCTTCGTAATAATTTTTTTTATAGTGATACCAATTAT
GATCGGCGGATTTGGTAATTGATTAATTCCACTAATAATTGGTGCCCCAGATATG
GCTTTCCCTCGCATGAATAATATAAGCTTTTGACTACTACCCCCCTCCTTCCTCCT
CCTCCTTGCCTCCTCCACTGTTGAAGCTGGGGTGGGAACAGGCTGAACTGTGTAC
CCCCCCCTCTCAGGAAATTTAGCTCACGACGGCCCATCTGTAGACCTAGCCATCT
TCTCCCTTCACCTAGCAGGGGTATCTTCAATTTTGGGGGCCATTAATTTTATCACC

ACTATTATCAACATAAAACCTCCAACCACCTCCCTGTATAATGCACCACTATTTAT
TTGATCTCTCCTTGTCACGGCTGTTCTTTTACTTCTCTCTCTACCAGTCCTTGCTGC
GGGTATCACCATACTTCTAACAGATCGAAACCT"/>
<sequence id="seq_Apaul_Mito_Apisto_300387"
taxon="Apaul_Mito_Apisto_300387" totalcount="4"
value="CACTAATTAATCTCCCCGCTCCCTCCAACATCTCTGCTTGATGGAACTTCG
GATCTCTATTAGGTCTATGCTTAATTTCCCAAATCCTAACAGGCTTATTTCTTTCT
ATACACTACACTGCCGACATCAATACAGCTTTTTCATCCATCACCCACATCTGCCG
AGATGTAAACTACGGATGACTAATCCGAAATTTACATGCCAATGGAGCATCCTTT
TTCTTCATTTGTGTTTACTTACACATTGCACGAGGCCTCTATTACGGCTCATTTCTC
TACAAAGAAACATGAAGCATTGGAGTAGCCCTTCTACTATTAGTTATAATAACCG
CTTTTGTAGGCTATGTTCTCCCGTGAGGACAAATATCGTTTTGAGGGGCCACAGT
CATCACCAACCTACTTTCCGCTGTCCCTTACATTGGAAACTCACTAGTTCAATGAA
TCTGAGGTGGCTTCTCAATCGACAATGCCACCCTTACCCGATTCTTCGCTATCCAT
TTCCTGCTTCCATTTGCTATCGCAGCCCTAACCCTAATTCACCTAATTTTTCTTCAT
GAAACAGGGTCCACTAACCCAATTGGTTTAAACTCAAACACAGATAAAATTTCCT
TCCACCCATATTTCTCCTTCAAAGACATTCTCGGTTTCTTAGCCCTACTCCTGGCTT
TAATGTTCGGTGCCTGAGCTGGGATAGCAGGAACCGCGCTAAGTATACTAATTCG
AGCTGAGCTGACTCAGCCCGGCTCCTTTTTTGGAGACGACCAAATCTATAATGTA
ATCGTTACTGCACATGCCTTCGTAATAATCTTCTTTATAGTAATACCCATTATAAT
TGGCGGGTTCGGTAATTGATTAATTCCACTAATAATTGGTGCCCCAGACATGGCT
TTCCCTCGCATAAATAATATAAGCTTTTGACTACTACCCCCCTCCTTCCTCCTCCTT
CTCGCCTCTTCAACTGTTGAAGCGGGTGTCGGGACAGGCTGAACCGTGTACCCCC
CCCTCTCAGGGAATTTAGCCCACGATGGCCCATCTGTAGACCTGGCCATCTTCTCC
CTCCACCTGGCGGGAGTATCTTCAATTTTAGGTGCAATCAATTTCATTACCACTAT
TATTAACATAAAACCTCCAACCACCTCCCTGTATAATGCACCATTATTTATCTGAT
CTCTCCTTGTCACGGCTGTTCTTCTACTTCTCTCCTTACCAGTCCTTGCTGCAGGTA
TCACTATACTTCTAACAGATCGAAACCT"/>
<sequence id="seq_Apaul_Mito_Apisto_300391"
taxon="Apaul_Mito_Apisto_300391" totalcount="4"
value="CACTAATTAATCTCCCCGCTCCCTCCAACATCTCTGCTTGATGGAACTTCG
GATCTCTATTAGGTCTATGCTTAATTTCCCAAATCCTAACAGGCTTATTTCTTTCT
ATACACTACACTGCCGACATCAATACAGCTTTTTCATCCATCACCCACATCTGCCG
AGATGTAAACTACGGATGACTAATCCGAAATTTACATGCCAATGGAGCATCCTTT
TTCTTCATTTGTGTTTACTTACACATTGCACGAGGCCTCTATTACGGCTCATTTCTC
TACAAAGAAACATGAAGCATTGGAGTAGCCCTTCTACTATTAGTTATAATAACCG
CTTTTGTAGGCTATGTTCTCCCGTGAGGACAAATATCGTTTTGAGGGGCCACAGT
CATCACCAACCTACTTTCCGCTGTCCCTTACATTGGAAACTCACTAGTTCAATGAA
TCTGAGGTGGCTTCTCAATCGACAATGCCACCCTTACCCGATTCTTCGCTATCCAT
TTCCTGCTTCCATTTGCTATCGCAGCCCTAACCCTAATTCACCTAATTTTTCTTCAT
GAAACAGGGTCCACTAACCCAATTGGTTTAAACTCAAACACAGATAAAATTTCCT
TCCACCCATATTTCTCCTTCAAAGACATTCTCGGTTTCTTAGCCCTACTCCTGGCTT
TAATGTTCGGTGCCTGAGCTGGGATAGCAGGAACCGCGCTAAGTATACTAATTCG
AGCTGAGCTGACTCAGCCCGGCTCCTTTTTTGGAGACGACCAAATCTATAATGTA
ATCGTTACTGCACATGCCTTCGTAATAATCTTCTTTATAGTAATACCCATTATAAT
TGGCGGGTTCGGTAATTGATTAATTCCACTAATAATTGGTGCCCCAGACATGGCT
TTCCCTCGCATAAATAATATAAGCTTTTGACTACTACCCCCCTCCTTCCTCCTCCTT
CTCGCCTCTTCAACTGTTGAAGCGGGTGTCGGGACAGGCTGAACCGTGTACCCCC
CCCTCTCAGGGAATTTAGCCCACGATGGCCCATCTGTAGACCTGGCCATCTTCTCC
CTCCACCTGGCGGGAGTATCTTCAATTTTAGGTGCAATCAATTTCATTACCACTAT

TATTAACATAAAACCTCCAACCACCTCCCTGTATAATGCACCATTATTTATcTGAT
CTCTCCTTGTCACGGCTGTTcTTCTACTTCTCTCCTTACCAGTCCTTGCTGCAGGTA
TCACTATACTTCTAACAGATCgAAACCT"/>
<sequence id="seq_Apaul_Mito_Apisto_300393"
taxon="Apaul_Mito_Apisto_300393" totalcount="4"
value="CACTAATTAATCTCCCCGCTCCCTCCAACATCTCTGCTTGATGGAACTTCG
GATCTCTATTAGGTCTATGCTTAATTTCCCAAATCCTAACAGGCTTATTTCTTTCT
ATACACTACACTGCCGACATCAATACAGCTTTTTCATCCATCACCCACATCTGCCG
AGATGTAAACTACGGATGACTAATCCGAAATTTACATGCCAATGGAGCATCCTTT
TTCTTCATTTGTGTTTACTTACACATTGCACGAGGCCTCTATTACGGCTCATTTCTC
TACAAAGAAACATGAAGCATTGGAGTAGCCCTTCTACTATTAGTTATAATAACCG
CTTTTGTAGGCTATGTTCTCCCGTGAGGACAAATATCGTTTTGAGGGGCCACAGT
CATCACCAACCTACTTTCCGCTGTCCCTTACATTGGAAACTCACTAGTTCAATGAA
TCTGAGGTGGCTTCTCAATCGACAATGCCACCCTTACCCGATTCTTCGCTATCCAT
TTCCTGCTTCCATTTGCTATCGCAGCCCTAACCCTAATTCACCTAATTTTTCTTCAT
GAAACAGGGTCCACTAACCCAATTGGTTTAAACTCAAACACAGATAAAATTTCCT
TCCACCCATATTTCTCCTTCAAAGACATTCTCGGTTTCTTAGCCCTACTCCTGGCTT
TAATGTTCGGTGCCTGAGCTGGGATAGCAGGAACCGCGCTAAGTATACTAATTCG
AGCTGAGCTGACTCAGCCCGGCTCCTTTTTTGGAGACGACCAAATCTATAATGTA
ATCGTTACTGCACATGCCTTCGTAATAATCTTCTTTATAGTAATACCCATTATAAT
TGGCGGGTTCGGTAATTGATTAATTCCACTAATAATTGGTGCCCCAGACATGGCT
TTCCCTCGCATAAATAATATAAGCTTTTGACTACTACCCCCCTCCTTCCTCCTCCTT
CTCGCCTCTTCAACTGTTGAAGCGGGTGTCGGGACAGGCTGAACCGTGTACCCCC
CCCTCTCAGGGAATTTAGCCCACGATGGCCCATCTGTAGACCTGGCCATCTTCTCC
CTCCACCTGGCGGGAGTATCTTCAATTTTAGGTGCAATCAATTTCATTACCACTAT
TATTAACATAAAACCTCCAACCACCTCCCTGTATAATGCACCATTATTTATCTGAT
CTCTCCTTGTCACGGCTGTTCTTCTACTTCTCTCCTTACCAGTCCTTGCTGCAGGTA
TCACTATACTTCtAAcAGATCGaAACCT"/>
<sequence id="seq_Apaul_Mito_Apisto_302881"
taxon="Apaul_Mito_Apisto_302881" totalcount="4"
value="CACTAATTAATCTCCCCGCTCCCTCCAACATCTCTGCTTGATGGAACTTCG
GATCTCTATTAGGTCTATGCTTAATTTCCCAAATCCTAACAGGCTTATTTCTTTCT
ATACACTACACTGCCGACATCAATACAGCTTTTTCATCCATCACCCACATCTGCCG
AGATGTAAACTACGGATGACTAATCCGAAATTTACATGCCAATGGAGCATCCTTT
TTCTTCATTTGTGTTTACTTACACATTGCACGAGGCCTCTATTACGGCTCATTTCTC
TACAAAGAAACATGAAGCATTGGAGTAGCCCTTCTACTATTAGTTATAATAACCG
CTTTTGTAGGCTATGTTCTCCCGTGAGGACAAATATCGTTTTGAGGGGCCACAGT
CATCACCAACCTACTTTCCGCTGTCCCTTACATTGGAAACTCACTAGTTCAATGAA
TCTGAGGTGGCTTCTCAATCGACAATGCCACCCTTACCCGATTCTTCGCTATCCAT
TTCCTGCTTCCATTTGCTATCGCAGCCCTAACCCTAATTCACCTAATTTTTCTTCAT
GAAACAGGGTCCACTAACCCAATTGGTTTAAACTCAAACACAGATAAAATTTCCT
TCCACCCATATTTCTCCTTCAAAGACATTCTCGGTTTCTTAGCCCTACTCCTGGCTT
TAATGTTCGGTGCCTGAGCTGGGATAGCAGGAACCGCGCTAAGTATACTAATTCG
AGCTGAGCTGACTCAGCCCGGCTCCTTTTTTGGAGACGACCAAATCTATAATGTA
ATCGTTACTGCACATGCCTTCGTAATAATCTTCTTTATAGTAATACCCATTATAAT
TGGCGGGTTCGGTAATTGATTAATTCCACTAATAATTGGTGCCCCAGACATGGCT
TTCCCTCGCATAAATAATATAAGCTTTTGACTACTACCCCCCTCCTTCCTCCTCCTT
CTCGCCTCTTCAACTGTTGAAGCGGGTGTCGGGACAGGCTGAACCGTGTACCCCC
CCCTCTCAGGGAATTTAGCCCACGATGGCCCATCTGTAGACCTGGCCATCTTCTCC
CTCCACCTGGCGGGAGTATCTTCAATTTTAGGTGCAATCAATTTCATTACCACTAT

TATTAACATAAAACCTCCAACCACCTCCCTGTATAATGCACCATTATTTATCTGAT
CTCTCCTTGTCACGGCTGTTCTTCTACTTCTCTCCTTACCAGTCCTTGCTGCAGGTA
TCACTATACTTCTAACAGATCGaAACCT"/>
<sequence id="seq_Apaul_Mito_Apisto_302882"
taxon="Apaul_Mito_Apisto_302882" totalcount="4"
value="CACTAATTAATCTCCCCGCTCCCTCCAACATCTCTGCTTGATGGAACTTCG
GATCTCTATTAGGTCTATGCTTAATTTCCCAAATCCTAACAGGCTTATTTCTTTCT
ATACACTACACTGCCGACATCAATACAGCTTTTCATCCATCACCCACATCTGCCG
AGATGTAAACTACGGATGACTAATCCGAAATTTACATGCCAATGGAGCATCCTTT
TTCTTCATTTGTGTTTACTTACACATTGCACGAGGCCTCTATTACGGCTCATTTCTC
TACAAAGAAACATGAAGCATTGGAGTAGCCCTTCTACTATTAGTTATAATAACCG
CTTTTGTAGGCTATGTTCTCCCGTGAGGACAAATATCGTTTTGAGGGGCCACAGT
CATCACCAACCTACTTTCCGCTGTCCCTTACATTGGAAACTCACTAGTTCAATGAA
TCTGAGGTGGCTTCTCAATCGACAATGCCACCCTTACCCGATTCTTCGCTATCCAT
TTCCTGCTTCCATTTGCTATCGCAGCCCTAACCCTAATTCACCTAATTTTTCTTCAT
GAAACAGGGTCCACTAACCCAATTGGTTTAAACTCAAACACAGATAAAATTTCCT
TCCACCCATATTTCTCCTTCAAAGACATTCTCGGTTTCTTAGCCCTACTCCTGGCTT
TAATGTTCGGTGCCTGAGCTGGGATAGCAGGAACCGCGCTAAGTATACTAATTCG
AGCTGAGCTGACTCAGCCCGGCTCCTTTTTTGGAGACGACCAAATCTATAATGTA
ATCGTTACTGCACATGCCTTCGTAATAATCTTCTTTATAGTAATACCCATTATAAT
TGGCGGGTTCGGTAATTGATTAATTCCACTAATAATTGGTGCCCCAGACATGGCT
TTCCCTCGCATAAATAATATAAGCTTTTGACTACTACCCCCCTCCTTCCTCCTCCTT
CTCGCCTCTTCAACTGTTGAAGCGGGTGTCGGGACAGGCTGAACCGTGTACCCCC
CCCTCTCAGGGAATTTAGCCCACGATGGCCCATCTGTAGACCTGGCCATCTTCTCC
CTCCACCTGGCGGGAGTATCTTCAATTTTAGGTGCAATCAATTTCATTACCACTAT
TATTAACATAAAACCTCCAACCACCTCCCTGTATAATGCACCATTATTTATCTGAT
CTCTCCTTGTCACGGCTGTTCTTCTACTTCTCTCCTTACCAGTCCTTGCTGCAGGTA
TCACTATACTTCTAACAGATCGAAACCT"/>
<sequence id="seq_Apaul_Mito_Apisto_302883"
taxon="Apaul_Mito_Apisto_302883" totalcount="4"
value="CACTAATTAATCTCCCCGCTCCCTCCAACATCTCTGCTTGATGGAACTTCG
GATCTCTATTAGGTCTATGCTTAATTTCCCAAATCCTAACAGGCTTATTTCTTTCT
ATACACTACACTGCCGACATCAATACAGCTTTTCATCCATCACCCACATCTGCCG
AGATGTAAACTACGGATGACTAATCCGAAATTTACATGCCAATGGAGCATCCTTT
TTCTTCATTTGTGTTTACTTACACATTGCACGAGGCCTCTATTACGGCTCATTTCTC
TACAAAGAAACATGAAGCATTGGAGTAGCCCTTCTACTATTAGTTATAATAACCG
CTTTTGTAGGCTATGTTCTCCCGTGAGGACAAATATCGTTTTGAGGGGCCACAGT
CATCACCAACCTACTTTCCGCTGTCCCTTACATTGGAAACTCACTAGTTCAATGAA
TCTGAGGTGGCTTCTCAATCGACAATGCCACCCTTACCCGATTCTTCGCTATCCAT
TTCCTGCTTCCATTTGCTATCGCAGCCCTAACCCTAATTCACCTAATTTTTCTTCAT
GAAACAGGGTCCACTAACCCAATTGGTTTAAACTCAAACACAGATAAAATTTCCT
TCCACCCATATTTCTCCTTCAAAGACATTCTCGGTTTCTTAGCCCTACTCCTGGCTT
TAATGTTCGGTGCCTGAGCTGGGATAGCAGGAACCGCGCTAAGTATACTAATTCG
AGCTGAGCTGACTCAGCCCGGCTCCTTTTTTGGAGACGACCAAATCTATAATGTA
ATCGTTACTGCACATGCCTTCGTAATAATCTTCTTTATAGTAATACCCATTATAAT
TGGCGGGTTCGGTAATTGATTAATTCCACTAATAATTGGTGCCCCAGACATGGCT
TTCCCTCGCATAAATAATATAAGCTTTTGACTACTACCCCCCTCCTTCCTCCTCCTT
CTCGCCTCTTCAACTGTTGAAGCGGGTGTCGGGACAGGCTGAACCGTGTACCCCC
CCCTCTCAGGGAATTTAGCCCACGATGGCCCATCTGTAGACCTGGCCATCTTCTCC
CTCCACCTGGCGGGAGTATCTTCAATTTTAGGTGCAATCAATTTCATTACCACTAT

TATTAACATAAAACCTCCAACCACCTCCCTGTATAATGCACCATTATTTATCTGAT
CTCTCCTTGTCACGGCTGTTCTTCTACTTCTCTCCTTACCAGTCCTTGCTGCAGGTA
TCACTATACTTCTAACAGATCgAAACCT"/>
<sequence id="seq_Apaul_Mito_Apisto_302885"
taxon="Apaul_Mito_Apisto_302885" totalcount="4"
value="CACTAATTAATCTCCCCGCTCCCTCCAACATCTCTGCTTGATGGAACTTCG
GATCTCTATTAGGTCTATGCTTAATTTCCCAAATCCTAACAGGCTTATTTCTTTCT
ATACACTACACTGCCGACATCAATACAGCTTTTCATCCATCACCCACATCTGCCG
AGATGTAAACTACGGATGACTAATCCGAAATTTACATGCCAATGGAGCATCCTTT
TTCTTCATTTGTGTTTACTTACACATTGCACGAGGCCTCTATTACGGCTCATTTCTC
TACAAAGAAACATGAAGCATTGGAGTAGCCCTTCTACTATTAGTTATAATAACCG
CTTTTGTAGGCTATGTTCTCCCGTGAGGACAAATATCGTTTTGAGGGGCCACAGT
CATCACCAACCTACTTTCCGCTGTCCCTTACATTGGAAACTCACTAGTTCAATGAA
TCTGAGGTGGCTTCTCAATCGACAATGCCACCCTTACCCGATTCTTCGCTATCCAT
TTCCTGCTTCCATTTGCTATCGCAGCCCTAACCCTAATTCACCTAATTTTTCTTCAT
GAAACAGGGTCCACTAACCCAATTGGTTTAAACTCAAACACAGATAAAATTTCCT
TCCACCCATATTTCTCCTTCAAAGACATTCTCGGTTTCTTAGCCCTACTCCTGGCTT
TAATGTTCGGTGCCTGAGCTGGGATAGCAGGAACCGCGCTAAGTATACTAATTCG
AGCTGAGCTGACTCAGCCCGGCTCCTTTTTTGGAGACGACCAAATCTATAATGTA
ATCGTTACTGCACATGCCTTCGTAATAATCTTCTTTATAGTAATACCCATTATAAT
TGGCGGGTTCGGTAATTGATTAATTCCACTAATAATTGGTGCCCCAGACATGGCT
TTCCCTCGCATAAATAATATAAGCTTTTGACTACTACCCCCCTCCTTCCTCCTCCTT
CTCGCCTCTTCAACTGTTGAAGCGGGTGTCGGGACAGGCTGAACCGTGTACCCCC
CCCTCTCAGGGAATTTAGCCCACGATGGCCCATCTGTAGACCTGGCCATCTTCTCC
CTCCACCTGGCGGGAGTATCTTCAATTTTAGGTGCAATCAATTTCATTACCACTAT
TATTAACATAAAACCTCCAACCACCTCCCTGTATAATGCACCATTATTTATCTGAT
CTCTCCTTGTCACGGCTGTTCTTCTACTTCTCTCCTTACCAGTCCTTGCTGCAGGTA
TCACTATACTTCTAACAGATCgAAACCT"/>
<sequence id="seq_Apeb_Mito_Apisto_302561"
taxon="Apeb_Mito_Apisto_302561" totalcount="4"
value="CACTAATTGACCTCCCCACCCCCTCCAACATCTCTGCTTGATGAAATTTCG
GGTCTCTACTAGGGCTATGCTTAATCTCCCAAATCCTAACAGGCTTATTTCTTTCC
ATACACTATACTGCCGACATCAACACAGCTTTTCATCCATCACTCACATCTGCCG
AGACGTAAACTACGGATGGCTAATCCAAAATCTACATGCCAACGGAGCATCCTTT
TTTTTCATTTGTATCTACTTACACATTGCACGAGGCCTCTATTACGGCTCATTTCTC
TACAAAGAAACATGAAACATCGGAGTAATCCTTCTACTATTAGTAATAATAACCG
CTTTTGTAGGCTACGTACTCCCATGAGGACAAATATCGTTTTGAGGGGCTACAGT
CATTACCAACCTACTTTCCGCTGTTCCTTACATTGGAAACTCACTAGTTCAATGAA
TCTGAGGTGGCTTCTCAGTCGACAGTGCCACCCTTACACGATTCTTCGCTATCCAT
TTTCTGCTTCCATTTGTTATTGCAGCCCTAACCCTAATTCACCTAATCTTTCTTCAT
GAGACAGGATCCACCAACCCAATTGGACTAAGCCCAAACACAGACAAAATCTCC
TTCCACCCATACTTCTCTTACAAAGACCTTCTAGGTTTCTTAATCCTACTTTTAACT
CTAGTGTTCGGTGCCTGGGCTGGTATAGCAGGAACCGCACTAAGCATGCTTATTC
GAGCTGAACTAACTCAGCCCGGTTCCTTTTTTGGGGACGACCAAATCTATAATGT
AATTGTTACTGCACATGCCTTCGTAATAATTTTTTTTATGGTAATGCCAATTATAA
TTGGCGGATTTGGTAATTGATTAATTCCACTAATAATTGGTGCCCCAGATATGGC
CTTCCCTCGCATAAATAATATAAGCTTTTGACTACTACCCCCCTCCTTCCTCCTCC
TCCTTGCCTCCTCCACTGTTGAAGCTGGGGTGGGAACAGGCTGAACTGTGTACCC
CCCCCTCTCAGGGAATTTAGCTCACGACGGCCCATCTGTAGACCTAGCCATCTTCT
CCCTTCACCTAGCAGGGGTATCTTCAATTTTGGGTGCAATTAATTTTATCACCACT

ATTATTAACATAAAACCCCCAACCACCTCCCTATATAACGCACCATTATTTATCTG
ATCTCTCCTTGTCACGGCTGTTCTTCTACTTCTCTCTCTACCAGTCCTTGCTGCAGG
TATCACTATACTTCTAACAGATCGAAACCT"/>
<sequence id="seq_Apeb_Mito_Apisto_302562"
taxon="Apeb_Mito_Apisto_302562" totalcount="4"
value="CACTAATTGACCTCCCCACCCCCTCCAACATCTCTGCTTGATGAAATTTCG
GGTCTCTACTAGGGCTATGCTTAATCTCCCAAATCCTAACAGGCTTATTTCTTTCC
ATACACTATACTGCCGACATCAACACAGCTTTTTCATCCATCACTCACATCTGCCG
AGACGTAAACTACGGATGGCTAATCCAAAATCTACATGCCAACGGAGCATCCTTT
TTTTTCATTTGTATCTACTTACACATTGCACGAGGCCTCTATTACGGCTCATTTCTC
TACAAAGAAACATGAAACATCGGAGTAATCCTTCTACTATTAGTAATAATAACCG
CTTTTGTAGGCTACGTACTCCCATGAGGACAAATATCGTTTTGAGGGGCTACAGT
CATTACCAACCTACTTTCCGCTGTTCCTTACATTGGAAACTCACTAGTTCAATGAA
TCTGAGGTGGCTTCTCAGTCGACAGTGCCACCCTTACACGATTCTTCGCTATCCAT
TTTCTGCTTCCATTTGTTATTGCAGCCCTAACCCTAATTCACCTAATCTTTCTTCAT
GAGACAGGATCCACCAACCCAATTGGACTAAGCCCAAACACAGACAAAATCTCC
TTCCACCCATACTTCTCTTACAAAGACCTTCTAGGTTTCTTAATCCTACTTTTAACT
CTAGTGTTCGGTGCCTGGGCTGGTATAGCAGGAACCGCACTAAGCATGCTTATTC
GAGCTGAACTAACTCAGCCCGGTTCCTTTTTTGGGGACGACCAAATCTATAATGT
AATTGTTACTGCACATGCCTTCGTAATAATTTTTTTTATGGTAATGCCAATTATAA
TTGGCGGATTTGGTAATTGATTAATTCCACTAATAATTGGTGCCCCAGATATGGC
CTTCCCTCGCATAAATAATATAAGCTTTTGACTACTACCCCCCTCCTTCCTCCTCC
TCCTTGCCTCCTCCACTGTTGAAGCTGGGGTGGGAACAGGCTGAACTGTGTACCC
CCCCCTCTCAGGGAATTTAGCTCACGACGGCCCATCTGTAGACCTAGCCATCTTCT
CCCTTCACCTAGCAGGGGTATCTTCAATTTTGGGTGCAATTAATTTTATCACCACT
ATTATTAACATAAAACCCCCAACCACCTCCCTATATAACGCACCATTATTTATCTG
ATCTCTCCTTGTCACGGCTGTTCTTCTACTTCTCTCTCTACCAGTCCTTGCTGCAGG
TATCACTATACTTCTAACAGATCGAAACCT"/>
<sequence id="seq_Apeb_Mito_Apisto_302563"
taxon="Apeb_Mito_Apisto_302563" totalcount="4"
value="CACTAATTGACCTCCCCACCCCCTCCAACATCTCTGCTTGATGAAATTTCG
GGTCTCTACTAGGGCTATGCTTAATCTCCCAAATCCTAACAGGCTTATTTCTTTCC
ATACACTATACTGCCGACATCAACACAGCTTTTTCATCCATCACTCACATCTGCCG
AGACGTAAACTACGGATGGCTAATCCAAAATCTACATGCCAACGGAGCATCCTTT
TTTTTCATTTGTATCTACTTACACATTGCACGAGGCCTCTATTACGGCTCATTTCTC
TACAAAGAAACATGAAACATCGGAGTAATCCTTCTACTATTAGTAATAATAACCG
CTTTTGTAGGCTACGTACTCCCATGAGGACAAATATCGTTTTGAGGGGCTACAGT
CATTACCAACCTACTTTCCGCTGTTCCTTACATTGGAAACTCACTAGTTCAATGAA
TCTGAGGTGGCTTCTCAGTCGACAGTGCCACCCTTACACGATTCTTCGCTATCCAT
TTTCTGCTTCCATTTGTTATTGCAGCCCTAACCCTAATTCACCTAATCTTTCTTCAT
GAGACAGGATCCACCAACCCAATTGGACTAAGCCCAAACACAGACAAAATCTCC
TTCCACCCATACTTCTCTTACAAAGACCTTCTAGGTTTCTTAATCCTACTTTTAACT
CTAGTGTTCGGTGCCTGGGCTGGTATAGCAGGAACCGCACTAAGCATGCTTATTC
GAGCTGAACTAACTCAGCCCGGTTCCTTTTTTGGGGACGACCAAATCTATAATGT
AATTGTTACTGCACATGCCTTCGTAATAATTTTTTTTATGGTAATGCCAATTATAA
TTGGCGGATTTGGTAATTGATTAATTCCACTAATAATTGGTGCCCCAGATATGGC
CTTCCCTCGCATAAATAATATAAGCTTTTGACTACTACCCCCCTCCTTCCTCCTCC
TCCTTGCCTCCTCCACTGTTGAAGCTGGGGTGGGAACAGGCTGAACTGTGTACCC
CCCCCTCTCAGGGAATTTAGCTCACGACGGCCCATCTGTAGACCTAGCCATCTTCT
CCCTTCACCTAGCAGGGGTATCTTCAATTTTGGGTGCAATTAATTTTATCACCACT

ATTATTAACATAAAACCCCCAACCACCTCCCTATATAACGCACCATTATTTATCTG
ATCTCTCCTTGTCACGGCTGTTCTTCTACTTCTCTCTCTACCAGTCCTTGCTGCAGG
TATCACTATACTTCTAACAGATCGAAACCT"/>
<sequence id="seq_Apeb_Mito_Apisto_302564"
taxon="Apeb_Mito_Apisto_302564" totalcount="4"
value="CACTAATTGACCTCCCCACCCCCTCCAACATCTCTGCTTGATGAAATTTCG
GGTCTCTACTAGGGCTATGCTTAATCTCCCAAATCCTAACAGGCTTATTTCTTTCC
ATACACTATACTGCCGACATCAACACAGCTTTTTCATCCATCACTCACATCTGCCG
AGACGTAAACTACGGATGGCTAATCCAAAATCTACATGCCAACGGAGCATCCTTT
TTTTTCATTTGTATCTACTTACACATTGCACGAGGCCTCTATTACGGCTCATTTCTC
TACAAAGAAACATGAAACATCGGAGTAATCCTTCTACTATTAGTAATAATAACCG
CTTTTGTAGGCTACGTACTCCCATGAGGACAAATATCGTTTTGAGGGGCTACAGT
CATTACCAACCTACTTTCCGCTGTTCCTTACATTGGAAACTCACTAGTTCAATGAA
TCTGAGGTGGCTTCTCAGTCGACAGTGCCACCCTTACACGATTCTTCGCTATCCAT
TTTCTGCTTCCATTTGTTATTGCAACCCTAACCCTAATTCACCTAATCTTTCTTCAT
GAGACAGGATCCACCAACCCAATTGGACTAAGCCCAAACACAGACAAAATCTCC
TTCCACCCATACTTCTCTTACAAAGACCTTCTAGGTTTCTTAATCCTACTTTTAACT
CTAGTGTTCGGTGCCTGGGCTGGTATAGCAGGAACCGCACTAAGCATGCTTATTC
GAGCTGAACTAACTCAGCCCGGTTCCTTTTTTGGGGACGACCAAATCTATAATGT
AATTGTTACTGCACATGCCTTCGTAATAATTTTTTTTATGGTAATGCCAATTATAA
TTGGCGGATTTGGTAATTGATTAATTCCACTAATAATTGGTGCCCCAGATATGGC
CTTCCCTCGCATAAATAATATAAGCTTTTGACTACTACCCCCCTCCTTCCTCCTCC
TCCTTGCCTCCTCCACTGTTGAAGCTGGGGTGGGAACAGGCTGAACTGTGTACCC
CCCCCTCTCAGGGAATTTAGCTCACGACGGCCCATCTGTAGACCTAGCCATCTTCT
CCCTTCACCTAGCAGGGGTATCTTCAATTTTGGGTGCAATTAATTTTATCACCACT
ATTATTAACATAAAACCCCCAACCACCTCCCTATATAACGCACCATTATTTATCTG
ATCTCTCCTTGTCACGGCTGTTCTTCTACTTCTCTCTCTACCAGTCCTTGCTGCAGG
TATCACTATACTTCTAACAGATCGAAACCT"/>
<sequence id="seq_Apeb_Mito_Apisto_302565"
taxon="Apeb_Mito_Apisto_302565" totalcount="4"
value="CACTAATTGACCTCCCCACCCCCTCCAACATCTCTGCTTGATGAAATTTCG
GGTCTCTACTAGGGCTATGCTTAATCTCCCAAATCCTAACAGGCTTATTTCTTTCC
ATACACTATACTGCCGACATCAACACAGCTTTTTCATCCATCACTCACATCTGCCG
AGACGTAAACTACGGATGGCTAATCCAAAATCTACATGCCAACGGAGCATCCTTT
TTTTTCATTTGTATCTACTTACACATTGCACGAGGCCTCTATTACGGCTCATTTCTC
TACAAAGAAACATGAAACATCGGAGTAATCCTTCTACTATTAGTAATAATAACCG
CTTTTGTAGGCTACGTACTCCCATGAGGACAAATATCGTTTTGAGGGGCTACAGT
CATTACCAACCTACTTTCCGCTGTTCCTTACATTGGAAACTCACTAGTTCAATGAA
TCTGAGGTGGCTTCTCAGTCGACAGTGCCACCCTTACACGATTCTTCGCTATCCAT
TTTCTGCTTCCATTTGTTATTGCAGCCCTAACCCTAATTCACCTAATCTTTCTTCAT
GAGACAGGATCCACCAACCCAATTGGACTAAGCCCAAACACAGACAAAATCTCC
TTCCACCCATACTTCTCTTACAAAGACCTTCTAGGTTTCTTAATCCTACTTTTAACT
CTAGTGTTCGGTGCCTGGGCTGGTATAGCAGGAACCGCACTAAGCATGCTTATTC
GAGCTGAACTAACTCAGCCCGGTTCCTTTTTTGGGGACGACCAAATCTATAATGT
AATTGTTACTGCACATGCCTTCGTAATAATTTTTTTTATGGTAATGCCAATTATAA
TTGGCGGATTTGGTAATTGATTAATTCCACTAATAATTGGTGCCCCAGATATGGC
CTTCCCTCGCATAAATAATATAAGCTTTTGACTACTACCCCCCTCCTTCCTCCTCC
TCCTTGCCTCCTCCACTGTTGAAGCTGGGGTGGGAACAGGCTGAACTGTGTACCC
CCCCCTCTCAGGGAATTTAGCTCACGACGGCCCATCTGTAGACCTAGCCATCTTCT
CCCTTCACCTAGCAGGGGTATCTTCAATTTTGGGTGCAATTAATTTTATCACCACT

ATTATTAACATAAAACCCCCAACCACCTCCCTATATAACGCACCATTATTTATCTG
ATCTCTCCTTGTCACGGCTGTTCTTCTACTTCTCTCTCTACCAGTCCTTGCTGCAGG
TATCACTATACTTCTAACAGATCGAAACCT"/>
<sequence id="seq_Aper_Mito_Apisto_301543"
taxon="Aper_Mito_Apisto_301543" totalcount="4"
value="CACTAATTAATCTCCCCGCCCCCTCCAACATCTCTGCTTGATGGAACTTCG
GATCTCTATTAGGTCTATGCTTAATTTCCCAAGTCCTAACAGGCTTATTTCTTTCC
ATACACTACACTGCCGACATCAATACAGCTTTTCATCCATCACCCACATCTGCCG
AGATGTAAACTACGGATGACTAATCCGAAATTTACATGCCAATGGAGCATCCTTT
TTCTTCATTTGTGTTTACTTACACATTGCACGAGGCCTCTATTACGGCTCATTTCTC
TACAAAGAAACATGAGGCATTGGAGTAGTCCTTCTACTATTAGTTATAATAACCG
CTTTTGTAGGCTATGTTCTCCCGTGAGGACAAATATCGTTTTGAGGGGCCACAGT
CATCACCAACCTACTTTCCGCTGTCCCTTACATTGGAAACTCACTAGTCCAATGAA
TCTGAGGTGGCTTCTCAGTCGACAATGCCACCCTTACCCGATTCTTCGCTATCCAT
TTCCTGCTTCCATTTGTTATCGCAGCCCTAACCCTAATTCACCTAATTTTTCTTCAT
GAAACAGGATCCACTAACCCAATTGGTTTAAACTCAAACACAGATAAAATTTCCT
TCCACCCATATTTCTCCTACAAAGACATTCTCGGTTTCTTAGCCCTACTCCTGGCT
TTAATgTTtGGTGCcTGAGCTGGtATAGCAGGAACCGCAcTAAGCATGCTAATTCGA
GCtGAaCTaACTCAGCCCGGTTCCTTTtTTGGGGACGAcCAAATaTATAATGTAATTG
TtACTGCACAtGCCTTCGTAATAATTTTTTTTATAGTgATgCCAATTATGATtGGCGG
ATTTGGtAATTGATTAATTCCACTAATAATTGGtGCCCCAGAtATGGCTTTCCCTCG
CATgAAtAAtATAAGCTTTTGACTaCTaCCCCCcTCcTTCCTCCTCCTCCTTGCCTCCT
CCACTGTTGAAGCtGGGGTgGGGACAGGCTGAACTGTgTACCCCCCcCTcTCAGGG
AATTTAGCCCACGACGGCCCATCtGTcGACCTGGCCATCTTcTCCCTTCACTTAGCA
GGGGTATCTTCAATTTTGGGtGCAATtAATTTTATCACCACTATtATCAACATAAAA
CCTCCAACCACCTCCCTGTATAATGCACCATTATTTATTTGATCTCTCCTTGTCAC
GGCTGTTCTTTTACTTCTCTCTCTACCAGTCCTTGCTGCAGGTATCACCATACTTCT
AACAGATCGAAACCT"/>
<sequence id="seq_Aros_Mito_Apisto_301032”
taxon="Aros_Mito_Apisto_301032” totalcount="4"
value="CACTAATTGACCTTCCCGCCCCCTCCAACATCTCCTCTTGATGAAATTTCG
GGTCCCTACTAGGCCTCTGCTTAATTTCCCAAATCCTAACAGGCTTATTCCTTTCC
ATACATTACACTGCTGACATCAACACAGCTTTCTCATCCGTTGCCCACATCTGCCG
AGATGTGAACTACGGATGACTAATTCGAAATCTACATGCTAACGGAGCATCTTTT
TTCTTTATTTGCATCTACCTGCACATCGGACGGGGCCTTTACTTCGGCTCTTACCT
CTACAAAGAAACATGAAACATTGGGGTAGTGCTCCTACTGCTAGTTATAATAACC
GCTTTTGTAGGCTATGTCCTCCCATGAGGACAAATATCATTTTGAGGGGCCACCG
TCATTACCAACTTGCTATCAGCCATCCCCTACATCGGGGACTTCCTAGTCCAATGA
ATTTGAGGCGGCTTCTCAATTGACAACGCTACCCTAACCCGATTCTTTGCCATCCA
CTTCCTTCTCCCGTTCGTCATCACAGCCATAACCTTGATACACCTAATTTTCCTCC
ACGAAACAGGCTCTACAAACCCCATCGGACTAAACCCAAACACAGATAAAATTT
CTTTCCACCCATTCTACTCCCTCAAAGACCTCCTTGGCTTCCTAATTTTACTCACA
ACTCTTATATTTGGAGCCTGAGCTGGGATAGCAGGCACCGCATTAAGCATACTAA
TTCGAGCAGAACTTACTCAACCCGGCTCCTTTTTCGGAGACGATCAAGCTTATAA
TGTAGTCGTAACTGCACACGCCTTTGTAATAATCTTTTTTATAGTTATACCAATTA
TGATTGGCGGGTTTGGTAATTGACTAATCCCACTAATACTCGGCGCCCCAGACAT
GGCTTTCCCTCGAATAAATAATATAAGCTTTTGATTACTCCCCCCATCTTTCCTCC
TCCTCCTCGCCTCCTCAACTGTTGAAGCTGGTGTAGGAACGGGCTGAACTGTATA
CCCACCCCTTTCCGGGAATTTAGCCCATGATGGCCCATCCGTGGACCTGGCCATTT
TCTCCCTCCACTTGGCAGGAATCTCCTCCATTCTAGGTGCAATCAACTTTATCACC

ACCATCATTAACATGAAACCTCCAGCCATCTCCATATACACTACACCCCTGTTTAT
CTGATCCCTCCTCATTACTGCTGTACTTCTACTTCTTTCGCTACCAGTTCTTGCTGC
TGGCATCACCATACTTCTCACAGACCGAAACTT"/>
<sequence id="seq_Aros_Mito_Apisto_301034”
taxon="Aros_Mito_Apisto_301034” totalcount="4"
value="CACTAATTGACCTTCCCGCCCCCTCCAACATCTCCTCTTGATGAAATTTCG
GGTCCCTACTAGGCCTCTGCTTAATTTCCCAAATCCTAACAGGCTTATTCCTTTCC
ATACATTACACTGCTGACATCAACACAGCTTTCTCATCCGTTGCCCACATCTGCCG
AGATGTGAACTACGGATGACTAATTCGAAATCTACATGCTAACGGAGCATCTTTT
TTCTTTATTTGCATCTACCTGCACATCGGACGGGGCCTTTACTTCGGCTCTTACCT
CTACAAAGAAACATGAAACATTGGGGTAGTGCTCCTACTGCTAGTTATAATAACC
GCTTTTGTAGGCTATGTCCTCCCATGAGGACAAATATCATTTTGAGGGGCCACCG
TCATTACCAACTTGCTATCAGCCATCCCCTACATCGGGGACTTCCTAGTCCAATGA
ATTTGAGGCGGCTTCTCAATTGACAACGCTACCCTAACCCGATTCTTTGCCATCCA
CTTCCTTCTCCCGTTCGTCATCACAGCCATAACCTTGATACACCTAATTTTCCTCC
ACGAAACAGGCTCTACAAACCCCATCGGACTAAACCCAAACACAGATAAAATTT
CTTTCCACCCATTCTACTCCCTCAAAGACCTCCTTGGCTTCCTAATTTTACTCACA
ACTCTTATATTTGGAGCCTGAGCTGGGATAGCAGGCACCGCATTAAGCATACTAA
TTCGAGCAGAACTTACTCAACCCGGCTCCTTTTTCGGAGACGATCAAGCTTATAA
TGTAGTCGTAACTGCACACGCCTTTGTAATAATCTTTTTTATAGTTATACCAATTA
TGATTGGCGGGTTTGGTAATTGACTAATCCCACTAATACTCGGCGCCCCAGACAT
GGCTTTCCCTCGAATAAATAATATAAGCTTTTGATTACTCCCCCCATCTTTCCTCC
TCCTCCTCGCCTCCTCAACTGTTGAAGCTGGTGTAGGAACGGGCTGAACTGTATA
CCCACCCCTTTCCGGGAATTTAGCCCATGATGGCCCATCCGTGGACCTGGCCATTT
TCTCCCTCCACTTGGCAGGAATCTCCTCCATTCTAGGTGCAATCAACTTTATCACC
ACCATCATTAACATGAAACCTCCAGCCATCTCCATATACACTACACCCCTGTTTAT
CTGATCCCTCCTCATTACTGCTGTACTTCTACTTCTTTCGCTACCAGTTCTTGCTGC
TGGCATCACCATACTTCTCACAGACCGAAACTT"/>
<sequence id="seq_Aros_Mito_Apisto_301035”
taxon="Aros_Mito_Apisto_301035” totalcount="4"
value="CACTAATTGACCTTCCCGCCCCCTCCAACATCTCCTCTTGATGAAATTTCG
GGTCCCTACTAGGCCTCTGCTTAATTTCCCAAATCCTAACAGGCTTATTCCTTTCC
ATACATTACACTGCTGACATCAACACAGCTTTCTCATCCGTTGCCCACATCTGCCG
AGATGTGAACTACGGATGACTAATTCGAAATCTACATGCTAACGGAGCATCTTTT
TTCTTTATTTGCATCTACCTGCACATCGGACGGGGCCTTTACTTCGGCTCTTACCT
CTACAAAGAAACATGAAACATTGGGGTAGTGCTCCTACTGCTAGTTATAATAACC
GCTTTTGTAGGCTATGTCCTCCCATGAGGACAAATATCATTTTGAGGGGCCACCG
TCATTACCAACTTGCTATCAGCCATCCCCTACATCGGGGACTTCCTAGTCCAATGA
ATTTGAGGCGGCTTCTCAATTGACAACGCTACCCTAACCCGATTCTTTGCCATCCA
CTTCCTTCTCCCGTTCGTCATCACAGCCATAACCTTGATACACCTAATTTTCCTCC
ACGAAACAGGCTCTACAAACCCCATCGGACTAAACCCAAACACAGATAAAATTT
CTTTCCACCCATTCTACTCCCTCAAAGACCTCCTTGGCTTCCTAATTTTACTCACA
ACTCTTATATTTGGAGCCTGAGCTGGGATAGCAGGCACCGCATTAAGCATACTAA
TTCGAGCAGAACTTACTCAACCCGGCTCCTTTTTCGGAGACGATCAAGCTTATAA
TGTAGTCGTAACTGCACACGCCTTTGTAATAATCTTTTTTATAGTTATACCAATTA
TGATTGGCGGGTTTGGTAATTGACTAATCCCACTAATACTCGGCGCCCCAGACAT
GGCTTTCCCTCGAATAAATAATATAAGCTTTTGATTACTCCCCCCATCTTTCCTCC
TCCTCCTCGCCTCCTCAACTGTTGAAGCTGGTGTAGGAACGGGCTGAACTGTATA
CCCACCCCTTTCCGGGAATTTAGCCCATGATGGCCCATCCGTGGACCTGGCCATTT
TCTCCCTCCACTTGGCAGGAATCTCCTCCATTCTAGGTGCAATCAACTTTATCACC

ACCATCATTAACATGAAACCTCCAGCCATCTCCATATACACTACACCCCTGTTTAT
CTGATCCCTCCTCATTACTGCTGTACTTCTACTTCTTTCGCTACCAGTTCTTGCTGC
TGGCATCACCATACTTCTCACAGACCGAAACTT"/>
<sequence id="seq_Aros_Mito_Apisto_301037”
taxon="Aros_Mito_Apisto_301037” totalcount="4"
value="CACTAATTGACCTTCCCGCCCCCTCCAACATCTCCTCTTGATGAAATTTCG
GGTCCCTACTAGGCCTCTGCTTAATTTCCCAAATCCTAACAGGCTTATTCCTTTCC
ATACATTACACTGCTGACATCAACACAGCTTTCTCATCCGTTGCCCACATCTGCCG
AGATGTGAACTACGGATGACTAATTCGAAATCTACATGCTAACGGAGCATCTTTT
TTCTTTATTTGCATCTACCTGCACATCGGACGGGGCCTTTACTTCGGCTCTTACCT
CTACAAAGAAACATGAAACATTGGGGTAGTGCTCCTACTGCTAGTTATAATAACC
GCTTTTGTAGGCTATGTCCTCCCATGAGGACAAATATCATTTTGAGGGGCCACCG
TCATTACCAACTTGCTATCAGCCATCCCCTACATCGGGGACTTCCTAGTCCAATGA
ATTTGAGGCGGCTTCTCAATTGACAACGCTACCCTAACCCGATTCTTTGCCATCCA
CTTCCTTCTCCCGTTCGTCATCACAGCCATAACCTTGATACACCTAATTTTCCTCC
ACGAAACAGGCTCTACAAACCCCATCGGACTAAACCCAAACACAGATAAAATTT
CTTTCCACCCATTCTACTCCCTCAAAGACCTCCTTGGCTTCCTAATTTTACTCACA
ACTCTTATATTTGGAGCCTGAGCTGGGATAGCAGGCACCGCATTAAGCATACTAA
TTCGAGCAGAACTTACTCAACCCGGCTCCTTTTTCGGAGACGATCAAGCTTATAA
TGTAGTCGTAACTGCACACGCCTTTGTAATAATCTTTTTTATAGTTATACCAATTA
TGATTGGCGGGTTTGGTAATTGACTAATCCCACTAATACTCGGCGCCCCAGACAT
GGCTTTCCCTCGAATAAATAATATAAGCTTTTGATTACTCCCCCCATCTTTCCTCC
TCCTCCTCGCCTCCTCAACTGTTGAAGCTGGTGTAGGAACGGGCTGAACTGTATA
CCCACCCCTTTCCGGGAATTTAGCCCATGATGGCCCATCCGTGGACCTGGCCATTT
TCTCCCTCCACTTGGCAGGAATCTCCTCCATTCTAGGTGCAATCAACTTTATCACC
ACCATCATTAACATGAAACCTCCAGCCATCTCCATATACACTACACCCCTGTTTAT
CTGATCCCTCCTCATTACTGCTGTACTTCTACTTCTTTCGCTACCAGTTCTTGCTGC
TGGCATCACCATACTTCTCACAGACCGAAACTT"/>
<sequence id="seq_Asp2_Mito_Apisto_301290"
taxon="Asp2_Mito_Apisto_301290" totalcount="4"
value="CACTAATTGACCTCCCCACCCCCTCCAACATCTCTGCTTGATGGAATTTTG
GGTCTCTATTAGGATTATGCTTAATTTCCCAAATCCTAACAGGCTTATTTCTTTCC
ATACACTACACTGCCGACATCAATACAGCTTTTTCATCCATCACTCACATCTCCCG
AGATGTAAACTACGGATGACTAATCCGAAATTTACATGCCAACGGAGCATCCTTT
TTCTTCATTTGCATCTACCTACACATTGCACGAGGCCTCTATTACAGCTCATTTCT
CTACAAAGAAACATGAAACATTGGAGTAGCCCTTCTACTATTAGTTATAATAACC
GCTTTTGTAGGCTATGTCCTCCCGTGAGGGCAAATATCATTTTGAGGGGCCACAG
TCATCACCAATCTACTTTCCGCTGTCCCTTACATTGGAAACTCGCTAGTCCAATGA
ATCTGGGGTGGCTTCTCAGTCGACAATGCCACTCTTACCCGATTCTTCGCTATCCA
TTTCCTGCTTCCATTTGTTATCGCAGCCCTAACTCTAATTCACCTAATTTTTCTTCA
TGAAACAGGGTCCACTAACCCAATTGGGCTAAACTCAAACACAGACAAAATTTC
CTTTCACCCATACTTCTCTTACAAAGACCTTCTCGGTTTCTTAATCCTACTCTTGGC
TTTAATATTCGGAGCCTGAGCTGGGATAGCAGGGACCGCGCTAAGCATACTAATT
CGAGCTGAACTAACTCAGCCCGGCTCCTTTTTTGGGGACGACCAAATCTATAATG
TAATCGTTACTGCACATGCCTTCGTAATAATCTTCTTTATAGTAATGCCAATTATA
ATTGGCGGGTTTGGTAATTGATTAATCCCACTCATAATTGGTGCCCCAGACATGG
CTTTCCCTCGTATAAATAACATAAGCTTTTGACTACTACCCCCCTCCTTCCTCCTC
CTCCTCGCCTCTTCAACTGTTGAAGCCGGAGTGGGGACAGGCTGGACCGTGTACC
CCCCTCTCTCAGGGAACTTAGCCCACGATGGCCCATCGGTAGACCTAGCCATCTT
CTCCCTCCACCTAGCGGGAGTATCTTCAATCTTAGGTGCAATCAATTTCATTACCA

CTATTATTAACATAAAACCTCCAACCACCTCCCTGTATAATGCACCATTATTTATT
TGATCTCTTCTTGTCACGGCTGTTCTCCTACTTCTTTCCCTACCAGTCCTTGCTGCA
GGTATTACCATACTTCTAACAGATCGAAACCT"/>
<sequence id="seq_Asp2_Mito_Apisto_301291"
taxon="Asp2_Mito_Apisto_301291" totalcount="4"
value="CACTAATTGACCTCCCCACCCCCTCCAACATCTCTGCTTGATGGAATTTTG
GGTCTCTATTAGGATTATGCTTAATTTCCCAAATCCTAACAGGCTTATTTCTTTCC
ATACACTACACTGCCGACATCAATACAGCTTTTTCATCCATCACTCACATCTCCCG
AGATGTAAACTACGGATGACTAATCCGAAATTTACATGCCAACGGAGCATCCTTT
TTCTTCATTTGCATCTACCTACACATTGCACGAGGCCTCTATTACAGCTCATTTCT
CTACAAAGAAACATGAAACATTGGAGTAGCCCTTCTACTATTAGTTATAATAACC
GCTTTTGTAGGCTATGTCCTCCCGTGAGGGCAAATATCATTTTGAGGGGCCACAG
TCATCACCAATCTACTTTCCGCTGTCCCTTACATTGGAAACTCGCTAGTCCAATGA
ATCTGGGGTGGCTTCTCAGTCGACAATGCCACTCTTACCCGATTCTTCGCTATCCA
TTTCCTGCTTCCATTTGTTATCGCAGCCCTAACTCTAATTCACCTAATTTTTCTTCA
TGAAACAGGGTCCACTAACCCAATTGGGCTAAACTCAAACACAGACAAAATTTC
CTTTCACCCATACTTCTCTTACAAAGACCTTCTCGGTTTCTTAATCCTACTCTTGGC
TTTAATATTCGGAGCCTGAGCTGGGATAGCAGGGACCGCGCTAAGCATACTAATT
CGAGCTGAACTAACTCAGCCCGGCTCCTTTTTTGGGGACGACCAAATCTATAATG
TAATCGTTACTGCACATGCCTTCGTAATAATCTTCTTTATAGTAATGCCAATTATA
ATTGGCGGGTTTGGTAATTGATTAATCCCACTCATAATTGGTGCCCCAGACATGG
CTTTCCCTCGTATAAATAACATAAGCTTTTGACTACTACCCCCCTCCTTCCTCCTC
CTCCTCGCCTCTTCAACTGTTGAAGCCGGAGTGGGGACAGGCTGGACCGTGTACC
CCCCTCTCTCAGGGAACTTAGCCCACGATGGCCCATCGGTAGACCTAGCCATCTT
CTCCCTCCACCTAGCGGGAGTATCTTCAATCTTAGGTGCAATCAATTTCATTACCA
CTATTATTAACATAAAACCTCCAACCACCTCCCTGTATAATGCACCATTATTTATT
TGATCTCTTCTTGTCACGGCTGTTCTCCTACTTCTTTCCCTACCAGTCCTTGCTGCA
GGTATTACCATACTTCTAACAGATCGAAACCT"/>
<sequence id="seq_Asp2_Mito_Apisto_301292"
taxon="Asp2_Mito_Apisto_301292" totalcount="4"
value="CACTAATTGACCTCCCCACCCCCTCCAACATCTCTGCTTGATGGAATTTTG
GGTCTCTATTAGGATTATGCTTAATTTCCCAAATCCTAACAGGCTTATTTCTTTCC
ATACACTACACTGCCGACATCAATACAGCTTTTTCATCCATCACTCACATCTCCCG
AGATGTAAACTACGGATGACTAATCCGAAATTTACATGCCAACGGAGCATCCTTT
TTCTTCATTTGCATCTACCTACACATTGCACGAGGCCTCTATTACAGCTCATTTCT
CTACAAAGAAACATGAAACATTGGAGTAGCCCTTCTACTATTAGTTATAATAACC
GCTTTTGTAGGCTATGTCCTCCCGTGAGGGCAAATATCATTTTGAGGGGCCACAG
TCATCACCAATCTACTTTCCGCTGTCCCTTACATTGGAAACTCGCTAGTCCAATGA
ATCTGGGGTGGCTTCTCAGTCGACAATGCCACTCTTACCCGATTCTTCGCTATCCA
TTTCCTGCTTCCATTTGTTATCGCAGCCCTAACTCTAATTCACCTAATTTTTCTTCA
TGAAACAGGGTCCACTAACCCAATTGGGCTAAACTCAAACACAGACAAAATTTC
CTTTCACCCATACTTCTCTTACAAAGACCTTCTCGGTTTCTTAATCCTACTCTTGGC
TTTAATATTCGGAGCCTGAGCTGGGATAGCAGGGACCGCGCTAAGCATACTAATT
CGAGCTGAACTAACTCAGCCCGGCTCCTTTTTTGGGGACGACCAAATCTATAATG
TAATCGTTACTGCACATGCCTTCGTAATAATCTTCTTTATAGTAATGCCAATTATA
ATTGGCGGGTTTGGTAATTGATTAATCCCACTCATAATTGGTGCCCCAGACATGG
CTTTCCCTCGTATAAATAACATAAGCTTTTGACTACTACCCCCCTCCTTCCTCCTC
CTCCTCGCCTCTTCAACTGTTGAAGCCGGAGTGGGGACAGGCTGGACCGTGTACC
CCCCTCTCTCAGGGAACTTAGCCCACGATGGCCCATCGGTAGACCTAGCCATCTT
CTCCCTCCACCTAGCGGGAGTATCTTCAATCTTAGGTGCAATCAATTTCATTACCA

CTATTATTAACATAAAACCTCCAACCACCTCCCTGTATAATGCACCATTATTTATT
TGATCTCTTCTTGTCACGGCTGTTCTCCTACTTCTTTCCCTACCAGTCCTTGCTGCA
GGTATTACCATACTTCTAACAGATCGAAACCT"/>
<sequence id="seq_Awol_Mito_Apisto_302976"
taxon="Awol_Mito_Apisto_302976" totalcount="4"
value="CTCTAATTGACCTTCCCGCTCCCTCTAACATCTCCTCTTGATGAAATTTCGG
GTCCCTACTGGGCCTCTGCTTAATTTCCCAGATCCTAACAGGCTTATTCCTTTCCA
TGCATTACACTGCTGACATCAGCACAGCTTTCTCATCCGTTGCCCACATCTGCCGA
GATGTAAACTACGGATGACTAATTCGAAATCTTCATGCCAACGGAGCATCTTTCT
TCTTTATTTGCATCTACCTACACATCGGACGGGGCCTCTACTTCGGCTCTTACCTC
TACAAAGAAACATGAAACATTGGGGTAGTGCTCCTACTACTAGTTATAATAACCG
CTTTTGTAGGCTATGTCCTCCCATGAGGACAAATATCGTTTTGGGGGGCCACCGT
CATTACCAACTTGCTATCAGCCATCCCCTACATCGGGGACTTCCTAGTCCAATGA
ATTTGAGGAGGCTTCTCAGTCGACAATGCTACCCTAACCCGATTCTTTGCCATCCA
TTTCCTCCTCCCGTTCATCATCACAGCCATAACCTTGATACACCTAATCTTCCTCC
ACGAAACAGGCTCTACAAACCCCATCGGACTAAGTTCAAACACAGATAAAATTT
CCTTCCATCCATTCTACTCCCTCAAAGACCTCCTTGGCTTTTTACTCTTACTCATAA
CTCTTATATTTGGtGCCTGagcTGGGatgGCAGGCACCGCATTAAGCatgcTAATTcGAG
CAGAAcTTAcCCAACCCGGcTTTTTTTTcGGGGACGATCAAGCCTATAATGTAATcG
TAACTGCACACGCCTTCGTAATAATTTTcTTTATAGTTATGCCAATTATGATTGGtG
GGTTTGGTAATTGAcTAATCCCGCTAATAATcGGCGCCCCGGACATGGCcTTCCCT
CGAATAAATAATATAAGCTTTTGACTACTcCCCCCaTCtTTCCTCCTCCTCCTAGCC
TCCTCaACTGTTGAAGCTGGtGTaGGAACGGGCTGAACTGTATACCCaCCCCTtTCcG
GAAATTTAGCcCACGACGGCCCATCCGTGGACCTGGCCATtTTCTCTCTTCATCTG
GCAGGAGTCTCCTCCATTTTAGGTGCAATCAACTTTATCACCACCATCgTTAACAT
GAAACCtCCAgCTATCTCCaTATACACTACACCTTTATTTATCTGATCCCTCCTCaTT
ACtGCTGTACTTCTACTTCTCTCACTGCCAGTTCTTGCTGCTGGcATCACTATACTT
CTCACAGAcCGAAACCT"/>
<sequence id="seq_Bio_Mito_Biotodoma" taxon="Bio_Mito_Biotodoma"
totalcount="4"
value="CCCTAATTGATCTTCCCGCACCCTCTAACATTTCCGTCTGATGAAATTTTG
GCTCTCTACTAGGACTTTGCCTCGCCACTCAAATCCTTACAGGACTATTCCTCGCG
ATACATTACACTTCAGACATTTCCACAGCCTTCTCCTCCGTAGCTCATATTTGCCG
AGATGTCAACTACGGATGACTTATTCGAAACATGCATGCTAACGGCGCATCCTTC
TTCTTCATCTGCATTTATCTCCATATTGGACGAGGCCTCTATTACGGCTCATATCT
TTACAAAGAGACATGAAACATTGGAGTAATCCTCCTCCTCCTAGTTATAATAACC
GCATTTGTAGGGTATGTCCTTCCCTGAGGACAAATATCCTTTTGAGGCGCAACCG
TTATCACTAACCTTCTCTCTGCAATCCCTTACATCGGCAACTCCCTCGTCCAATGG
ATCTGAGGCGGATTTTCAGTAGACAACGCCACCCTAACTCGATTCTTTGCCTTCCA
CTTTCTCTTCCCATTTGTCATTGCAGCTATAACCATAATTCACCTGATCTTTCTTCA
CGAAACCGGATCAACAAACCCAACAGGACTTAACTCAGACACAGACAAAATCCC
CTTCCACCCTTACTTTTCCTACAAAGATCTCTTAGGTTTTATCGTGCTACTAATTTC
CCTAATGTTTGGTGCCTGGGCCGGATTAGTAGGCACTGCGTTAAGTCTGTTAATTC
GGGCAGAACTAAGTCAACCCGGCACCCTCCTTGGAGAAGACCAAATCTATAACG
TTATTGTAACTGCACACGCCTTTGTGATAATCTTTTTTATAGTAATACCCGTAATG
ATCGGAGGTTTCGGTAATTGACTAATCCCCCTAATAATTGGAGCCCCCGATATAG
CCTTCCCCCGAATAAACAATATGAGCTTTTGACTCCTCCCCCCCGCACTTCTCCTC
CTCCTAGCCTCCGCAAGCGTGGAGGCAGGGGCAGGAACAGGATGAACTGTTTAT
CCCCCGCTAGCAGGAAACCTA-CACAC-C---
ACCCTCCGTTGACCTAGCAATCTTCTCTCTTCACTTAGCCGGTGCCTCCTCAATCT

TGGGGGCAATCAACTTTATCACCACTATTATTAACATAAAACCACCAGCCATTAC
TATGCTTAGTCTCCCCTTATTCGTGTGAGCACTCTTTATCACAGCCATTCTCCTCCT
ACTTTCTTTGCCAATTCTTGCTGCCAGCATCACTATGCTTCTAACCGATC-------"/>
<sequence id="seq_Cre_Mito_Crenicara" taxon="Cre_Mito_Crenicara"
totalcount="4"
value="CTCTAATTGATCTTCCCACACCCTCAAACATCTCTGCCTGGTGGAACTTTG
GATCCCTCCTAGGTCTTTGTCTTGCTACCCAAATCCTTACTGGCCTCTTCCTCGCG
ATACACTACACCCCTGACATCACCTTGGCTTTCTCTTCCACTGTTCACATCTGCCG
GGATGTAAACTATGGCTGACTCATCCGAAACCTCCACGCCAACGGCGCATCCTTC
TTTTTTATCTGTATCTATCTCCACATCGGACGAGGGCTCTACTATGGCTCCTATCT
CTATAAAGAGACCTGAAATGTCGGCATTATCCTCCTCCTTCTAATCATAATGACC
GCATTCGTAGGTTATGTACTTCCATGAGGCCAAATATCCCTCTGAGGGGCAACCG
TTATTACTAACCTCCTCTCTACAACCCCTTACGTTGGCAACTCCCTAGTCCAATGA
ATCTGAGGCGGGTTCTCAGTAGATAATGCCACCCTTACTCGATTCTTCACCTTTCA
TTTTCTCCTCCCCTTCACCGCCGCTGCCATAGCCTTAGTTCACCTCATCCTCCTCCA
CGAAACTGGCTCAACAAACCCCGCTGGATTAAACTCGGACTCAGACAAAATCTCC
TTCCACCCCTACTTCTCCTTCAAAGATCTTCTCGGATTCTTAGCCCTACTTACTAC
GTTAGTATTTGGTGCCTGAGCTGGGATAGCGGGCACTGCCCTGAGCCTCATAATT
CGAGCAGAGCTAACTCAACCTGGCTCCCTTCTTGGGGACGACCAAATTTATAATG
TTGTCGTCACCGCACACGCTTTTGTAATAATCTTTTTTATAGTAATACCCATCATA
ATCGGGGGGTTTGGCAACTGACTTATTCCCCTGATAATTGGCGCCCCTGATATAG
CCTTCCCCCGTATAAATAACATAAGCTTCTGACTTCTCCCCCCCTCATTCCTCCTC
CTCCTATCCTCTTCAGGAGTCGAAGCCGGAGTGGGCACAGGATGAACCGTGTACC
CCCCATTGGCAGGCAACCTTGCACATGCAGGCCCATCCGTAGACTTAGCCATCTT
TTCGCTCCATTTAGCAGGTATTTCCTCAATCTTAGGGGCAATCAATTTTATTACTA
CCATCATTAATATAAAACCCCCGGCCATTTGTATGCACCAGCTACCACTATTTATC
TGATCACTCTCCATCACCGCTGTTCTTCTTCTGCTGTCCCTTCCAGTTCTTGCTGCC
AGCATTACCATACTCCTCACTGACCGAAATTT"/>
<sequence id="seq_Geo_Mito_Geophagus" taxon="Geo_Mito_Geophagus"
totalcount="4"
value="CCCTAATTGACCTCCCCACACCCTCTAACATCTCTGTCTGATGAAACTTCG
GCTCCCTTCTAGGACTCTGCCTAGTTGCCCAAATCCTAACAGGCCTATTTCTTGCA
ATACACTATACCTCCGACATCGCCACAGCCTTTTCATCCGTCGCCCACATCTGTCG
AGATGTAAACTACGGCTGACTCATCCGAAACATACATGCCAACGGCGCATCCTTT
TTCTTTATCTGCATCTACCTCCATATTGGACGAGGCCTTTACTATGGCTCCTATCT
ATACAAAGAAACCTGAAACATCGGAGTAATTCTCCTACTTTTAGTCATGATAACT
GCATTTGTAGGCTACGTTCTTCCATGAGGACAAATATCATTCTGAGGGGCAACCG
TCATCACCAACCTCCTTTCCGCAATCCCCTACATCGGCAATTCCCTCGTACAATGA
ATCTGAGGCGGCTTCTCAGTAGACAACGCCACCCTAACCCGATTCTTTGCCTTCC
ACTTCCTCTTTCCATTCATTATTGCAGCCATAACCATGATTCACCTCATTTTCCTCC
ACGAAACCGGATCAACAAACCCAACAGGACTAAACTCAGACGCAGACAAAATCT
CCTTCCACCCATACTTCTCTTATAAAGATCTACTAGGCTTTGCCACTCTACTAATT
GCCCTAATATTTGGTGCCTGAGCTGGAATAGTGGGCACTGCTTTAAGCCTACTAA
TTCGAGCAGAACTAAGCCAACCTGGCTCCCTCCTTGGAGACGACCAAATTTATAA
TGTCATTGTTACTGCACACGCCTTTGTAATAATTTTCTTTATAGTAATGCCAGTTA
TAATTGGGGGCTTCGGCAACTGACTGGTACCACTAATAATTGGTGCTCCTGACAT
AGCCTTTCCTCGAATAAACAACATGAGTTTTTGACTCCTGCCCCCCTCATTTCTTC
TCCTCCTGGCCTCCTCAGGTGTTGAGGCGGGGGTGGGTACAGGATGAACTGTATA
CCCCCCATTAGCAGGTAATCTGGCACATGCCGGCCCATCTGTTGATTTAGCCATTT
TTTCCCTCCACCTGGCCGGGGTTTCCTCAATCTTGGGAGCAATCAATTTTATTACC

ACCATTATTAACATAAAACCCCCAGCCACCTCCCAATACCAAACACCCCTATTTG
TTTGATCAGTTCTTATTACCGCCATTCTTCTGCTTCTGTCCCTTCCAGTTCTTGCCG
CCGGCATTACTATACTTTTAACCGACCGAAACCT"/>
<sequence id="seq_Ggym_Mito_Gymnogymno"
taxon="Ggym_Mito_Gymnogymno" totalcount="4"
value="CATTAGTTGATCTTCCCACACCCTCAAATATTTCTGTCTGATGAAACTTTG
GTTCCCTACTAGGACTCTGTCTCGCCGCACAAATCCTGACGGGCCTATTCCTTGCC
ATACATTACACCTCTGACATTGCCACAGCCTTCTCCTCAGTTGCTCACATCTGCCG
AGATGTAAATTACGGCTGGCTAATTCGAAACATACATGCCAACGGCGCATCCTTC
TTCTTCATCTGTATCTATCTTCACATTGGGCGAGGACTTTATTACGGTTCTTACTTG
TTCAAAGAGACATGAAACATTGGAGTAATTCTTCTCCTCCTAGTAATAATAACTG
CATTTGTGGGCTACGTACTGCCATGAGGACAAATGTCCTTCTGAGGGGCCACCGT
CATTACAAATCTCCTCTCCGCCATCCCTTACATCGGTAACTCCCTCGTCCAATGAA
TTTGAGGCGGCTTCTCAGTAGACAACGCCACCCTAACCCGATTCTTTGCCTTTCAC
TTCCTTTTCCCCTTTATTATTGCAGCCATAACAGTTATTCATCTTATCTTTCTCCAT
GAAACCGGATCCACGAACCCAACAGGATTAAACTCAGACGCAGATAAAATCTCG
TTCCACCCCTACTTCTCCTATAAAGACCTCCTAGGCTTCGCCATCCTGCTAATTGC
TTTAATATTTGGTGCTTGAGCTGGAATAGTAGGCACAGCATTGAGCCTAATAATT
CGAGCAGAACTGAGCCAACCGGGCTCTCTCCTTGGAGATGATCAAATTTATAATG
TAATTGTTACTGCACACGCCTTTGTAATAATTTTCTTTATAGTCATACCAATTATA
ATTGGAGGTTTTGGCAACTGACTTATCCCCTTGATGATTGGCGCTCCTGATATAGC
TTTTCCCCGAATAAACAACATGAGTTTTTGACTCTTACCCCCATCATTTTTACTCC
TTCTATCCTCTTCAGGAGTTGAAGCTGGTGCGGGGACAGGGTGAACTGTATATCC
TCCGCTAGCGGGCAACCTTGCACACGCGGGCCCATCCGTCGATTTAGCCATCTTT
TCCCTTCACTTAGCCGGCGTGTCCTCTATTTTAGGAGCAATTAATTTTATTACGAC
CATCATTAACATAAAACCCCCAGCCATTTCTCAATATCAAACACCCTTATTTGTAT
GGGCAGTGCTCATTACCGCTGTTCTCCTTCTCCTGTCCCTCCCAGTTCTTGCTGCA
GGCATTACCATACTATTAACAGACC-------"/>
<sequence id="seq_Gmer_Mito_Gymnomer" taxon="Gmer_Mito_Gymnomer"
totalcount="4" value="---

GGCCTTTTCCTTGCCATACACTATACCTCTGATATTGCCACAGCCTTCTCCTCAGT
GGCCCACATTTGCCGAGATGTAAATTACGGCTGGCTTATCCGGAACATACACGCC
AACGGCGCGTCCTTCTTCTTCATCTGTATCTACCTCCACATTGGACGAGGACTTTA
CTACGGCTCCTACTTATATAAAGAGACATGAAACATTGGGGTGATTCTCCTCCTT
CTAGTAATAATAACTGCATTTGTAGGCTACGTATTACCATGAGGACAAATATCCT
TCTGAGGGGCCACCGTCATTACAAATCTCCTCTCCGCCATCCCCTACATCGGTAA
CTCCCTCGTCCAATGAATCTGAGGCGGCTTCTCAGTAGACAACGCTACCCTGACC
CGATTCTTTGCCTTCCACTTCCTCTTTCCCTTCATTATTGCAGCCATAACACTGATT
CATCTTATTTTCCTTCATGAAACAGGATCCACAAACCCAACAGGGCTAAACTCAG
ACGCAGACAAGATTTCCTTTCACCCCTACTTCTCCTATAAAGACCTCCTAGGATTC
GCCATTCTACTAATTGATTTAGTATTCGGTGCTTGAGCCGGAATAGTAGGCACAG
CATTAAGCCTGATAATTCGAGCAGAACTGAGCCAACCAGGCTCCCTCCTTGGAGA
TGACCAAATTTATAATGTAATTGTTACTGCACACGCCTTTGTAATAATTTTCTTTA
TAGTTATACCAGTCATGATTGGGGGCTTTGGTAACTGACTTGTCCCCCTGATGATT
GGCGCTCCTGATATAGCTTTCCCCCGAATAAACAACATGAGTTTTTGACTTCTACC
CCCCTCATTCTTACTCCTTCTGTCCTCTTCAGGCGTCGAAGCTGGTGCAGGGACAG
GGTGAACTGTATATCCCCCGCTAGCGGGCAATCTCGCACACGCCGGCCCATCGGT
TGATTTAGCCATCTTCTCCCTCCACTTGGCCGGTGTTTCCTCTATTTTAGGGGCAA
TTAATTTTATTACTACCATCATTAACATAAAACCCCCGGCCATCTCTCAATATCAA

```
ACACCACTATTTGTGTGATCAGTTCTCATTACCGCTGTTCTACTCCTCCTGTCCCTT
CCAGTTCTTGCTGCAGGCATCACCATACTATTAACAGACCGAAACCT"/>
                <sequence id="seq_Sat_Mito_Satanoperca" taxon="Sat_Mito_Satanoperca"
totalcount="4"
value="CACTAATTGATCTCCCCGCACCCTCAAACATTTCAGTCTGATGAAACTTTG
GCTCCCTCTTAGGACTCTGCCTACTCGCCCAAATCCTAACAGGCCTCTTCCTTGCA
ATACACTACACCTCAGATATCTCCATGGCCTTCTCATCCGTCGCCCACATCTGTCG
AGATGTAAACTACGGCTGACTTATCCGAAACATACATGCCAACGGCGCATCCTTC
TTTTTCATCTGCATTTACCTACACATCGGCCGAGGCCTTTACTACGGTTCCTACCT
CTACAAAGAAACATGAAACATTGGAGTAATTCTCCTCCTCCTAGTTATAATAACT
GCATTTGTAGGGTACGTCCTTCCATGAGGACAAATGTCATTTTGAGGGGCTACTG
TCATCACCAACCTTCTCTCCGCAATCCCCTACATCGGAAACTCTCTAGTCCAATGA
ATTTGAGGCGGCTTCTCAGTTGACAACGCCACCCTAACCCGATTTTTTGCCTTCCA
CTTCCTATTTCCATTCATCATTGCAGCCATAACCATAATTCACCTAATTTTTCTTCA
CGAGACCGGGTCAACAAACCCAACAGGACTAAACTCAGACGCAGACAAAATCTC
CTTCCACCCTTACTTCTCCTACAAAGATCTCCTGGGCTTTCTAGCCCTACTAATTG
CCCTCATATTTGGTGCTTGAGCTGGAATAGTAGGCACCGCATTGAGCCTAGTTAT
TCGAGCAGAACTTAGTCAGCCTGGCTCTCTCCTTGGAGACGATCAAATTTATAAT
GTAGTTGTTACTGCACATGCCTTTGTAATAATTTTCTTTATAGTCATGCCCATCAT
AATTGGAGGGTTTGGCAACTGACTTATCCCACTAATAATTGGCGCCCCAGACATA
GCATTCCCCCGAATAAATAATATAAGCTTTTGACTATTGCCCCCTTCATTTCTTCT
CCTTTTAGCCTCCTCCGGAGTTGAAGCTGGAGTCGGAACAGGATGAACCGTCTAC
CCACCTCTAGCAGGGAACCTAGCACACGCTGGCCCATCCGTTGATCTAGCTATCT
TCTCACTTCACCTAGCTGGGGTTTCCTCGATTTTAGGGGCAATTAACTTCATTACT
ACAATTATTAATATGAAACCCCCAGCTATTTCCCAATATCAAACACCTTTATTTAT
TTGAGCACTTTTAATTACCGCTATTCTTCTATTATTGTCCCTACCAGTTCTCGCTGC
CGGCATCACAATACTTTTAACGGACCGAAACTT"/>
            </data>

<map name="Uniform" >beast.math.distributions.Uniform</map>
<map name="Exponential" >beast.math.distributions.Exponential</map>
<map name="LogNormal" >beast.math.distributions.LogNormalDistributionModel</map>
<map name="Normal" >beast.math.distributions.Normal</map>
<map name="Beta" >beast.math.distributions.Beta</map>
<map name="Gamma" >beast.math.distributions.Gamma</map>
<map name="LaplaceDistribution" >beast.math.distributions.LaplaceDistribution</map>
<map name="prior" >beast.math.distributions.Prior</map>
<map name="InverseGamma" >beast.math.distributions.InverseGamma</map>
<map name="OneOnX" >beast.math.distributions.OneOnX</map>


<run id="mcmc" spec="MCMC" chainLength="100000000" storeEvery="5000">
    <state id="state" storeEvery="5000">
        <stateNode id="Tree.t:Species" spec="starbeast2.SpeciesTree">
            <taxonset id="taxonsuperset" spec="TaxonSet">
                <taxon id="Apaul" spec="TaxonSet">
                    <taxon id="Apaul_Mito_Apisto_300393" spec="Taxon"/>
                    <taxon id="Apaul_Mito_Apisto_300391" spec="Taxon"/>
                    <taxon id="Apaul_Mito_Apisto_300387" spec="Taxon"/>
                    <taxon id="Apaul_Mito_Apisto_302882" spec="Taxon"/>
```

```
    <taxon id="Apaul_Mito_Apisto_302881" spec="Taxon"/>
    <taxon id="Apaul_Mito_Apisto_302885" spec="Taxon"/>
    <taxon id="Apaul_Mito_Apisto_302883" spec="Taxon"/>
    <taxon id="Apaul_Nuc_Apisto_302885" spec="Taxon"/>
    <taxon id="Apaul_Nuc_Apisto_302883" spec="Taxon"/>
    <taxon id="Apaul_Nuc_Apisto_302882" spec="Taxon"/>
    <taxon id="Apaul_Nuc_Apisto_302881" spec="Taxon"/>
    <taxon id="Apaul_Nuc_Apisto_300393" spec="Taxon"/>
    <taxon id="Apaul_Nuc_Apisto_300391" spec="Taxon"/>
    <taxon id="Apaul_Nuc_Apisto_300387" spec="Taxon"/>
</taxon>
<taxon id="Amoa" spec="TaxonSet">
    <taxon id="Amoa_Mito_Apisto_302868" spec="Taxon"/>
    <taxon id="Amoa_Mito_Apisto_302866" spec="Taxon"/>
    <taxon id="Amoa_Mito_Apisto_302867" spec="Taxon"/>
    <taxon id="Amoa_Mito_Apisto_302864" spec="Taxon"/>
    <taxon id="Amoa_Mito_Apisto_302865" spec="Taxon"/>
    <taxon id="Amoa_Nuc_Apisto_302864" spec="Taxon"/>
    <taxon id="Amoa_Nuc_Apisto_302867" spec="Taxon"/>
    <taxon id="Amoa_Nuc_Apisto_302865" spec="Taxon"/>
    <taxon id="Amoa_Nuc_Apisto_302866" spec="Taxon"/>
    <taxon id="Amoa_Nuc_Apisto_302868" spec="Taxon"/>
</taxon>
<taxon id="Aeun" spec="TaxonSet">
    <taxon id="Aeun_Mito_Apisto_302940" spec="Taxon"/>
    <taxon id="Aeun_Mito_Apisto_302939" spec="Taxon"/>
    <taxon id="Aeun_Mito_Apisto_302938" spec="Taxon"/>
    <taxon id="Aeun_Nuc_Apisto_302938" spec="Taxon"/>
    <taxon id="Aeun_Nuc_Apisto_302940" spec="Taxon"/>
    <taxon id="Aeun_Mito_Apisto_301804" spec="Taxon"/>
    <taxon id="Aeun_Mito_Apisto_302558" spec="Taxon"/>
    <taxon id="Aeun_Mito_Apisto_302557" spec="Taxon"/>
    <taxon id="Aeun_Mito_Apisto_302556" spec="Taxon"/>
    <taxon id="Aeun_Mito_Apisto_302555" spec="Taxon"/>
    <taxon id="Aeun_Mito_Apisto_302554" spec="Taxon"/>
    <taxon id="Aeun_Mito_Apisto_302560" spec="Taxon"/>
    <taxon id="Aeun_Mito_Apisto_302559" spec="Taxon"/>
    <taxon id="Aeun_Nuc_Apisto_302556" spec="Taxon"/>
    <taxon id="Aeun_Nuc_Apisto_302557" spec="Taxon"/>
    <taxon id="Aeun_Nuc_Apisto_302554" spec="Taxon"/>
    <taxon id="Aeun_Nuc_Apisto_302555" spec="Taxon"/>
    <taxon id="Aeun_Nuc_Apisto_301804" spec="Taxon"/>
    <taxon id="Aeun_Nuc_Apisto_302558" spec="Taxon"/>
    <taxon id="Aeun_Nuc_Apisto_302559" spec="Taxon"/>
    <taxon id="Aeun_Nuc_Apisto_302560" spec="Taxon"/>
</taxon>
<taxon id="Aaga" spec="TaxonSet">
    <taxon id="Aaga_Nuc_Apisto_302958" spec="Taxon"/>
    <taxon id="Aaga_Nuc_Apisto_302959" spec="Taxon"/>
    <taxon id="Aaga_Mito_Apisto_300492" spec="Taxon"/>
```

```
    <taxon id="Aaga_Mito_Apisto_300494" spec="Taxon"/>
    <taxon id="Aaga_Nuc_Apisto_303077" spec="Taxon"/>
    <taxon id="Aaga_Nuc_Apisto_303078" spec="Taxon"/>
    <taxon id="Aaga_Mito_Apisto_302186" spec="Taxon"/>
    <taxon id="Aaga_Mito_Apisto_302185" spec="Taxon"/>
    <taxon id="Aaga_Mito_Apisto_302307" spec="Taxon"/>
    <taxon id="Aaga_Mito_Apisto_302306" spec="Taxon"/>
    <taxon id="Aaga_Mito_Apisto_302918" spec="Taxon"/>
    <taxon id="Aaga_Mito_Apisto_302911" spec="Taxon"/>
    <taxon id="Aaga_Mito_Apisto_302920" spec="Taxon"/>
    <taxon id="Aaga_Mito_Apisto_302902" spec="Taxon"/>
    <taxon id="Aaga_Nuc_Apisto_302186" spec="Taxon"/>
    <taxon id="Aaga_Nuc_Apisto_302185" spec="Taxon"/>
    <taxon id="Aaga_Nuc_Apisto_300494" spec="Taxon"/>
    <taxon id="Aaga_Nuc_Apisto_300492" spec="Taxon"/>
    <taxon id="Aaga_Nuc_Apisto_302918" spec="Taxon"/>
    <taxon id="Aaga_Nuc_Apisto_302920" spec="Taxon"/>
    <taxon id="Aaga_Nuc_Apisto_302902" spec="Taxon"/>
    <taxon id="Aaga_Nuc_Apisto_302911" spec="Taxon"/>
    <taxon id="Aaga_Mito_Apisto_302959" spec="Taxon"/>
    <taxon id="Aaga_Mito_Apisto_302958" spec="Taxon"/>
    <taxon id="Aaga_Mito_Apisto_303078" spec="Taxon"/>
    <taxon id="Aaga_Mito_Apisto_303077" spec="Taxon"/>
</taxon>
<taxon id="Abit3" spec="TaxonSet">
    <taxon id="Abit3_Nuc_Apisto_303098" spec="Taxon"/>
    <taxon id="Abit3_Mito_Apisto_303098" spec="Taxon"/>
</taxon>
<taxon id="Abit1" spec="TaxonSet">
    <taxon id="Abit1_Mito_Apisto_300440" spec="Taxon"/>
    <taxon id="Abit1_Mito_Apisto_300438" spec="Taxon"/>
    <taxon id="Abit1_Nuc_Apisto_300438" spec="Taxon"/>
    <taxon id="Abit1_Nuc_Apisto_300440" spec="Taxon"/>
</taxon>
<taxon id="Sat" spec="TaxonSet">
    <taxon id="Sat_Nuc_Satanoperca" spec="Taxon"/>
    <taxon id="Sat_Mito_Satanoperca" spec="Taxon"/>
</taxon>
<taxon id="Abit4" spec="TaxonSet">
    <taxon id="Abit4_Mito_Apisto_302612" spec="Taxon"/>
    <taxon id="Abit4_Mito_Apisto_302614" spec="Taxon"/>
    <taxon id="Abit4_Nuc_Apisto_302612" spec="Taxon"/>
    <taxon id="Abit4_Nuc_Apisto_302614" spec="Taxon"/>
</taxon>
<taxon id="Asp2" spec="TaxonSet">
    <taxon id="Asp2_Mito_Apisto_301291" spec="Taxon"/>
    <taxon id="Asp2_Mito_Apisto_301292" spec="Taxon"/>
    <taxon id="Asp2_Mito_Apisto_301290" spec="Taxon"/>
    <taxon id="Asp2_Nuc_Apisto_301292" spec="Taxon"/>
    <taxon id="Asp2_Nuc_Apisto_301291" spec="Taxon"/>
```

<taxon id="Asp2_Nuc_Apisto_301290" spec="Taxon"/>
</taxon>
<taxon id="Abit5" spec="TaxonSet">
<taxon id="Abit5_Nuc_Apisto_302952" spec="Taxon"/>
<taxon id="Abit5_Nuc_Apisto_302951" spec="Taxon"/>
<taxon id="Abit5_Mito_Apisto_302926" spec="Taxon"/>
<taxon id="Abit5_Mito_Apisto_302928" spec="Taxon"/>
<taxon id="Abit5_Nuc_Apisto_302926" spec="Taxon"/>
<taxon id="Abit5_Mito_Apisto_302951" spec="Taxon"/>
<taxon id="Abit5_Mito_Apisto_302952" spec="Taxon"/>
</taxon>
<taxon id="Ahua" spec="TaxonSet">
<taxon id="Ahua_Mito_Apisto_302709" spec="Taxon"/>
<taxon id="Ahua_Mito_Apisto_302705" spec="Taxon"/>
<taxon id="Ahua_Mito_Apisto_302706" spec="Taxon"/>
<taxon id="Ahua_Mito_Apisto_302707" spec="Taxon"/>
<taxon id="Ahua_Mito_Apisto_302708" spec="Taxon"/>
<taxon id="Ahua_Nuc_Apisto_302708" spec="Taxon"/>
<taxon id="Ahua_Nuc_Apisto_302705" spec="Taxon"/>
<taxon id="Ahua_Nuc_Apisto_302706" spec="Taxon"/>
<taxon id="Ahua_Nuc_Apisto_302707" spec="Taxon"/>
<taxon id="Ahua_Nuc_Apisto_302709" spec="Taxon"/>
</taxon>
<taxon id="Gmer" spec="TaxonSet">
<taxon id="Gmer_Mito_Gymnomer" spec="Taxon"/>
<taxon id="Gmer_Nuc_Gymnomer" spec="Taxon"/>
</taxon>
<taxon id="Aore" spec="TaxonSet">
<taxon id="Aore_Nuc_Apisto_302894" spec="Taxon"/>
<taxon id="Aore_Nuc_Apisto_302893" spec="Taxon"/>
<taxon id="Aore_Nuc_Apisto_302895" spec="Taxon"/>
<taxon id="Aore_Nuc_Apisto_302892" spec="Taxon"/>
<taxon id="Aore_Nuc_Apisto_302891" spec="Taxon"/>
<taxon id="Aore_Mito_Apisto_302893" spec="Taxon"/>
<taxon id="Aore_Mito_Apisto_302892" spec="Taxon"/>
<taxon id="Aore_Mito_Apisto_302891" spec="Taxon"/>
<taxon id="Aore_Mito_Apisto_302895" spec="Taxon"/>
<taxon id="Aore_Mito_Apisto_302894" spec="Taxon"/>
</taxon>
<taxon id="Apap" spec="TaxonSet">
<taxon id="Apap_Mito_Apisto_301770" spec="Taxon"/>
<taxon id="Apap_Mito_Apisto_301769" spec="Taxon"/>
<taxon id="Apap_Mito_Apisto_301768" spec="Taxon"/>
<taxon id="Apap_Mito_Apisto_301766" spec="Taxon"/>
<taxon id="Apap_Nuc_Apisto_301773" spec="Taxon"/>
<taxon id="Apap_Nuc_Apisto_301774" spec="Taxon"/>
<taxon id="Apap_Nuc_Apisto_301768" spec="Taxon"/>
<taxon id="Apap_Nuc_Apisto_301770" spec="Taxon"/>
<taxon id="Apap_Nuc_Apisto_301769" spec="Taxon"/>
<taxon id="Apap_Nuc_Apisto_301766" spec="Taxon"/>

<taxon id="Apap_Mito_Apisto_301774" spec="Taxon"/>
<taxon id="Apap_Mito_Apisto_301773" spec="Taxon"/>
</taxon>
<taxon id="Aper" spec="TaxonSet">
<taxon id="Aper_Nuc_Apisto_301543" spec="Taxon"/>
<taxon id="Aper_Mito_Apisto_301543" spec="Taxon"/>
</taxon>
<taxon id="Aalg" spec="TaxonSet">
<taxon id="Aalg_Mito_Apisto_300788" spec="Taxon"/>
<taxon id="Aalg_Mito_Apisto_300787" spec="Taxon"/>
<taxon id="Aalg_Nuc_Apisto_300788" spec="Taxon"/>
<taxon id="Aalg_Nuc_Apisto_300787" spec="Taxon"/>
</taxon>
<taxon id="Aere" spec="TaxonSet">
<taxon id="Aere_Mito_Apisto_300403" spec="Taxon"/>
<taxon id="Aere_Mito_Apisto_300405" spec="Taxon"/>
<taxon id="Aere_Mito_Apisto_300412" spec="Taxon"/>
<taxon id="Aere_Mito_Apisto_300401" spec="Taxon"/>
<taxon id="Aere_Mito_Apisto_300402" spec="Taxon"/>
<taxon id="Aere_Nuc_Apisto_300403" spec="Taxon"/>
<taxon id="Aere_Nuc_Apisto_300402" spec="Taxon"/>
<taxon id="Aere_Nuc_Apisto_300401" spec="Taxon"/>
<taxon id="Aere_Nuc_Apisto_300412" spec="Taxon"/>
<taxon id="Aere_Nuc_Apisto_300405" spec="Taxon"/>
</taxon>
<taxon id="Bio" spec="TaxonSet">
<taxon id="Bio_Mito_Biotodoma" spec="Taxon"/>
<taxon id="Bio_Nuc_Biotodoma" spec="Taxon"/>
</taxon>
<taxon id="Aros" spec="TaxonSet">
<taxon id="Aros_Mito_Apisto_301037” spec="Taxon"/>
<taxon id="Aros_Mito_Apisto_301034” spec="Taxon"/>
<taxon id="Aros_Mito_Apisto_301035” spec="Taxon"/>
<taxon id="Aros_Nuc_Apisto_301032” spec="Taxon"/>
<taxon id="Aros_Nuc_Apisto_301034” spec="Taxon"/>
<taxon id="Aros_Nuc_Apisto_301035” spec="Taxon"/>
<taxon id="Aros_Nuc_Apisto_301037” spec="Taxon"/>
<taxon id="Aros_Mito_Apisto_301032” spec="Taxon"/>
</taxon>
<taxon id="Acar" spec="TaxonSet">
<taxon id="Acar_Mito_Apisto_302365" spec="Taxon"/>
<taxon id="Acar_Mito_Apisto_302364" spec="Taxon"/>
<taxon id="Acar_Mito_Apisto_302367" spec="Taxon"/>
<taxon id="Acar_Mito_Apisto_302366" spec="Taxon"/>
<taxon id="Acar_Nuc_Apisto_302365" spec="Taxon"/>
<taxon id="Acar_Nuc_Apisto_302366" spec="Taxon"/>
<taxon id="Acar_Nuc_Apisto_302367" spec="Taxon"/>
<taxon id="Acar_Nuc_Apisto_302364" spec="Taxon"/>
</taxon>
<taxon id="Amel" spec="TaxonSet">

<taxon id="Amel_Nuc_Apisto_301641" spec="Taxon"/>
<taxon id="Amel_Nuc_Apisto_301649” spec="Taxon"/>
<taxon id="Amel_Nuc_Apisto_301639” spec="Taxon"/>
<taxon id="Amel_Nuc_Apisto_301640" spec="Taxon"/>
<taxon id="Amel_Nuc_Apisto_301650" spec="Taxon"/>
<taxon id="Amel_Mito_Apisto_301649” spec="Taxon"/>
<taxon id="Amel_Mito_Apisto_301641" spec="Taxon"/>
<taxon id="Amel_Mito_Apisto_301640" spec="Taxon"/>
<taxon id="Amel_Mito_Apisto_301639” spec="Taxon"/>
<taxon id="Amel_Mito_Apisto_301650" spec="Taxon"/>
</taxon>
<taxon id="Awol" spec="TaxonSet">
<taxon id="Awol_Mito_Apisto_302976" spec="Taxon"/>
<taxon id="Awol_Nuc_Apisto_302976" spec="Taxon"/>
</taxon>
<taxon id="Abar" spec="TaxonSet">
<taxon id="Abar_Mito_Apisto_300549" spec="Taxon"/>
<taxon id="Abar_Mito_Apisto_300542" spec="Taxon"/>
<taxon id="Abar_Mito_Apisto_300554" spec="Taxon"/>
<taxon id="Abar_Mito_Apisto_300550" spec="Taxon"/>
<taxon id="Abar_Mito_Apisto_300557" spec="Taxon"/>
<taxon id="Abar_Nuc_Apisto_300554" spec="Taxon"/>
<taxon id="Abar_Nuc_Apisto_300549" spec="Taxon"/>
<taxon id="Abar_Nuc_Apisto_300550" spec="Taxon"/>
<taxon id="Abar_Nuc_Apisto_300542" spec="Taxon"/>
<taxon id="Abar_Nuc_Apisto_300557" spec="Taxon"/>
<taxon id="Abar_Nuc_Apisto_302256" spec="Taxon"/>
<taxon id="Abar_Nuc_Apisto_302258" spec="Taxon"/>
<taxon id="Abar_Nuc_Apisto_302257" spec="Taxon"/>
<taxon id="Abar_Nuc_Apisto_302259" spec="Taxon"/>
<taxon id="Abar_Mito_Apisto_302259" spec="Taxon"/>
<taxon id="Abar_Mito_Apisto_302258" spec="Taxon"/>
<taxon id="Abar_Mito_Apisto_302257" spec="Taxon"/>
<taxon id="Abar_Mito_Apisto_302256" spec="Taxon"/>
<taxon id="Abar_Nuc_Apisto_302897" spec="Taxon"/>
<taxon id="Abar_Nuc_Apisto_302896" spec="Taxon"/>
<taxon id="Abar_Nuc_Apisto_302899" spec="Taxon"/>
<taxon id="Abar_Mito_Apisto_302900" spec="Taxon"/>
<taxon id="Abar_Nuc_Apisto_302900" spec="Taxon"/>
<taxon id="Abar_Mito_Apisto_302897" spec="Taxon"/>
<taxon id="Abar_Mito_Apisto_302896" spec="Taxon"/>
<taxon id="Abar_Mito_Apisto_302899" spec="Taxon"/>
</taxon>
<taxon id="Amor" spec="TaxonSet">
<taxon id="Amor_Nuc_Apisto_302816" spec="Taxon"/>
<taxon id="Amor_Nuc_Apisto_302817" spec="Taxon"/>
<taxon id="Amor_Nuc_Apisto_302818" spec="Taxon"/>
<taxon id="Amor_Nuc_Apisto_302819" spec="Taxon"/>
<taxon id="Amor_Nuc_Apisto_302773" spec="Taxon"/>
<taxon id="Amor_Nuc_Apisto_302775" spec="Taxon"/>

<taxon id="Amor_Nuc_Apisto_302776" spec="Taxon"/>
<taxon id="Amor_Nuc_Apisto_301730" spec="Taxon"/>
<taxon id="Amor_Nuc_Apisto_301792" spec="Taxon"/>
<taxon id="Amor_Nuc_Apisto_300578" spec="Taxon"/>
<taxon id="Amor_Nuc_Apisto_300577" spec="Taxon"/>
<taxon id="Amor_Nuc_Apisto_300564" spec="Taxon"/>
<taxon id="Amor_Nuc_Apisto_300581" spec="Taxon"/>
<taxon id="Amor_Nuc_Apisto_302772" spec="Taxon"/>
<taxon id="Amor_Nuc_Apisto_301727" spec="Taxon"/>
<taxon id="Amor_Nuc_Apisto_301726" spec="Taxon"/>
<taxon id="Amor_Nuc_Apisto_301724" spec="Taxon"/>
<taxon id="Amor_Mito_Apisto_300577" spec="Taxon"/>
<taxon id="Amor_Mito_Apisto_300564" spec="Taxon"/>
<taxon id="Amor_Mito_Apisto_300578" spec="Taxon"/>
<taxon id="Amor_Mito_Apisto_300581" spec="Taxon"/>
<taxon id="Amor_Mito_Apisto_302819" spec="Taxon"/>
<taxon id="Amor_Mito_Apisto_302817" spec="Taxon"/>
<taxon id="Amor_Mito_Apisto_302818" spec="Taxon"/>
<taxon id="Amor_Mito_Apisto_302773" spec="Taxon"/>
<taxon id="Amor_Mito_Apisto_302776" spec="Taxon"/>
<taxon id="Amor_Mito_Apisto_302816" spec="Taxon"/>
<taxon id="Amor_Mito_Apisto_302774" spec="Taxon"/>
<taxon id="Amor_Mito_Apisto_302775" spec="Taxon"/>
<taxon id="Amor_Mito_Apisto_302772" spec="Taxon"/>
<taxon id="Amor_Mito_Apisto_301601” spec="Taxon"/>
<taxon id="Amor_Nuc_Apisto_301599” spec="Taxon"/>
<taxon id="Amor_Nuc_Apisto_301600” spec="Taxon"/>
<taxon id="Amor_Nuc_Apisto_301601” spec="Taxon"/>
<taxon id="Amor_Mito_Apisto_301792" spec="Taxon"/>
<taxon id="Amor_Mito_Apisto_301730" spec="Taxon"/>
<taxon id="Amor_Mito_Apisto_301600” spec="Taxon"/>
<taxon id="Amor_Mito_Apisto_301599” spec="Taxon"/>
<taxon id="Amor_Mito_Apisto_301725”" spec="Taxon"/>
<taxon id="Amor_Mito_Apisto_301726" spec="Taxon"/>
<taxon id="Amor_Mito_Apisto_301724" spec="Taxon"/>
<taxon id="Amor_Mito_Apisto_301727" spec="Taxon"/>
</taxon>
<taxon id="Geo" spec="TaxonSet">
<taxon id="Geo_Mito_Geophagus" spec="Taxon"/>
<taxon id="Geo_Nuc_Geophagus" spec="Taxon"/>
</taxon>
<taxon id="Anij" spec="TaxonSet">
<taxon id="Anij_Mito_Apisto_300306" spec="Taxon"/>
<taxon id="Anij_Mito_Apisto_300304" spec="Taxon"/>
<taxon id="Anij_Mito_Apisto_300328" spec="Taxon"/>
<taxon id="Anij_Nuc_Apisto_300306" spec="Taxon"/>
<taxon id="Anij_Nuc_Apisto_300328" spec="Taxon"/>
<taxon id="Anij_Nuc_Apisto_300304" spec="Taxon"/>
</taxon>
<taxon id="Aata" spec="TaxonSet">

<taxon id="Aata_Mito_Apisto_300411" spec="Taxon"/>
<taxon id="Aata_Nuc_Apisto_302107" spec="Taxon"/>
<taxon id="Aata_Nuc_Apisto_302106" spec="Taxon"/>
<taxon id="Aata_Mito_Apisto_302107" spec="Taxon"/>
<taxon id="Aata_Mito_Apisto_302106" spec="Taxon"/>
<taxon id="Aata_Nuc_Apisto_301983" spec="Taxon"/>
<taxon id="Aata_Nuc_Apisto_301990" spec="Taxon"/>
<taxon id="Aata_Nuc_Apisto_301991" spec="Taxon"/>
<taxon id="Aata_Nuc_Apisto_300411" spec="Taxon"/>
<taxon id="Aata_Mito_Apisto_301983" spec="Taxon"/>
<taxon id="Aata_Mito_Apisto_301991" spec="Taxon"/>
<taxon id="Aata_Mito_Apisto_301990" spec="Taxon"/>
</taxon>
<taxon id="Abit" spec="TaxonSet">
<taxon id="Abit_Nuc_Apisto_302613" spec="Taxon"/>
<taxon id="Abit_Nuc_Apisto_302616" spec="Taxon"/>
<taxon id="Abit_Nuc_Apisto_302615" spec="Taxon"/>
<taxon id="Abit_Mito_Apisto_302615" spec="Taxon"/>
<taxon id="Abit_Mito_Apisto_302616" spec="Taxon"/>
<taxon id="Abit_Mito_Apisto_302613" spec="Taxon"/>
</taxon>
<taxon id="Apeb" spec="TaxonSet">
<taxon id="Apeb_Nuc_Apisto_302564" spec="Taxon"/>
<taxon id="Apeb_Nuc_Apisto_302565" spec="Taxon"/>
<taxon id="Apeb_Nuc_Apisto_302562" spec="Taxon"/>
<taxon id="Apeb_Nuc_Apisto_302563" spec="Taxon"/>
<taxon id="Apeb_Nuc_Apisto_302561" spec="Taxon"/>
<taxon id="Apeb_Mito_Apisto_302565" spec="Taxon"/>
<taxon id="Apeb_Mito_Apisto_302564" spec="Taxon"/>
<taxon id="Apeb_Mito_Apisto_302563" spec="Taxon"/>
<taxon id="Apeb_Mito_Apisto_302562" spec="Taxon"/>
<taxon id="Apeb_Mito_Apisto_302561" spec="Taxon"/>
</taxon>
<taxon id="Ggym" spec="TaxonSet">
<taxon id="Ggym_Nuc_Gymnogymno" spec="Taxon"/>
<taxon id="Ggym_Mito_Gymnogymno" spec="Taxon"/>
</taxon>
<taxon id="Ameg" spec="TaxonSet">
<taxon id="Ameg_Mito_Apisto_302872" spec="Taxon"/>
<taxon id="Ameg_Nuc_Apisto_302870" spec="Taxon"/>
<taxon id="Ameg_Mito_Apisto_302871" spec="Taxon"/>
<taxon id="Ameg_Nuc_Apisto_302871" spec="Taxon"/>
<taxon id="Ameg_Mito_Apisto_302870" spec="Taxon"/>
<taxon id="Ameg_Nuc_Apisto_302872" spec="Taxon"/>
<taxon id="Ameg_Mito_Apisto_302869" spec="Taxon"/>
<taxon id="Ameg_Nuc_Apisto_302873" spec="Taxon"/>
<taxon id="Ameg_Mito_Apisto_302873" spec="Taxon"/>
<taxon id="Ameg_Nuc_Apisto_302869" spec="Taxon"/>
</taxon>
<taxon id="Cre" spec="TaxonSet">

```
    <taxon id="Cre_Nuc_Crenicara" spec="Taxon"/>
    <taxon id="Cre_Mito_Crenicara" spec="Taxon"/>
</taxon>
<taxon id="Acin" spec="TaxonSet">
    <taxon id="Acin_Mito_Apisto_301619” spec="Taxon"/>
    <taxon id="Acin_Mito_Apisto_301628” spec="Taxon"/>
    <taxon id="Acin_Mito_Apisto_301616" spec="Taxon"/>
    <taxon id="Acin_Mito_Apisto_301618” spec="Taxon"/>
    <taxon id="Acin_Mito_Apisto_301629” spec="Taxon"/>
    <taxon id="Acin_Nuc_Apisto_301616" spec="Taxon"/>
    <taxon id="Acin_Nuc_Apisto_301618” spec="Taxon"/>
    <taxon id="Acin_Nuc_Apisto_301619” spec="Taxon"/>
    <taxon id="Acin_Nuc_Apisto_301628” spec="Taxon"/>
    <taxon id="Acin_Nuc_Apisto_301629” spec="Taxon"/>
    <taxon id="Acin_Nuc_Apisto_300374" spec="Taxon"/>
    <taxon id="Acin_Nuc_Apisto_300371" spec="Taxon"/>
    <taxon id="Acin_Nuc_Apisto_300378" spec="Taxon"/>
    <taxon id="Acin_Nuc_Apisto_300379" spec="Taxon"/>
    <taxon id="Acin_Nuc_Apisto_300380" spec="Taxon"/>
    <taxon id="Acin_Mito_Apisto_300380" spec="Taxon"/>
    <taxon id="Acin_Mito_Apisto_300379" spec="Taxon"/>
    <taxon id="Acin_Mito_Apisto_300378" spec="Taxon"/>
    <taxon id="Acin_Mito_Apisto_300374" spec="Taxon"/>
    <taxon id="Acin_Mito_Apisto_300371" spec="Taxon"/>
</taxon>
<taxon id="Ajur" spec="TaxonSet">
    <taxon id="Ajur_Nuc_Apisto_302879" spec="Taxon"/>
    <taxon id="Ajur_Nuc_Apisto_302878" spec="Taxon"/>
    <taxon id="Ajur_Nuc_Apisto_302880” spec="Taxon"/>
    <taxon id="Ajur_Mito_Apisto_302878" spec="Taxon"/>
    <taxon id="Ajur_Mito_Apisto_302879" spec="Taxon"/>
    <taxon id="Ajur_Mito_Apisto_302880” spec="Taxon"/>
</taxon>
<taxon id="Abae" spec="TaxonSet">
    <taxon id="Abae_Mito_Apisto_301679" spec="Taxon"/>
    <taxon id="Abae_Mito_Apisto_301678" spec="Taxon"/>
    <taxon id="Abae_Mito_Apisto_301677" spec="Taxon"/>
    <taxon id="Abae_Mito_Apisto_301676" spec="Taxon"/>
    <taxon id="Abae_Mito_Apisto_301654" spec="Taxon"/>
    <taxon id="Abae_Nuc_Apisto_301678" spec="Taxon"/>
    <taxon id="Abae_Nuc_Apisto_301677" spec="Taxon"/>
    <taxon id="Abae_Nuc_Apisto_301676" spec="Taxon"/>
    <taxon id="Abae_Nuc_Apisto_301654" spec="Taxon"/>
    <taxon id="Abae_Nuc_Apisto_301679" spec="Taxon"/>
</taxon>
<taxon id="Acac" spec="TaxonSet">
    <taxon id="Acac_Mito_Apisto_302196" spec="Taxon"/>
    <taxon id="Acac_Mito_Apisto_302195" spec="Taxon"/>
    <taxon id="Acac_Mito_Apisto_302303" spec="Taxon"/>
    <taxon id="Acac_Mito_Apisto_302197" spec="Taxon"/>
```

```
<taxon id="Acac_Mito_Apisto_302302" spec="Taxon"/>
<taxon id="Acac_Nuc_Apisto_300709" spec="Taxon"/>
<taxon id="Acac_Nuc_Apisto_300690" spec="Taxon"/>
<taxon id="Acac_Nuc_Apisto_301422" spec="Taxon"/>
<taxon id="Acac_Nuc_Apisto_301162" spec="Taxon"/>
<taxon id="Acac_Mito_Apisto_300690" spec="Taxon"/>
<taxon id="Acac_Mito_Apisto_301422" spec="Taxon"/>
<taxon id="Acac_Mito_Apisto_300709" spec="Taxon"/>
<taxon id="Acac_Mito_Apisto_301162" spec="Taxon"/>
<taxon id="Acac_Mito_Apisto_301505" spec="Taxon"/>
<taxon id="Acac_Nuc_Apisto_301505" spec="Taxon"/>
<taxon id="Acac_Nuc_Apisto_302196" spec="Taxon"/>
<taxon id="Acac_Nuc_Apisto_302195" spec="Taxon"/>
<taxon id="Acac_Nuc_Apisto_302197" spec="Taxon"/>
</taxon>
</taxonset>
</stateNode>
<parameter id="speciationRate.t:Species" lower="0.0"
name="stateNode">1.0</parameter>
<tree id="Tree.t:Apisto_Combi_Nuc" name="stateNode">
<taxonset id="TaxonSet.Apisto_Combi_Nuc" spec="TaxonSet">
<alignment idref="Apisto_Combi_Nuc"/>
</taxonset>
</tree>
<tree id="Tree.t:Apisto_Combi_Mito" name="stateNode">
<taxonset id="TaxonSet.Apisto_Combi_Mito" spec="TaxonSet">
<alignment idref="Apisto_Combi_Mito"/>
</taxonset>
</tree>
<parameter id="tipPopSizes.Species" lower="0.0" name="stateNode">1.0</parameter>
<parameter id="topPopSizes.Species" dimension="0" lower="0.0"
name="stateNode">1.0</parameter>
<parameter id="lwcrPopScale.Species" lower="0.0"
name="stateNode">1.0</parameter>
<parameter id="freqParameter.s:Apisto_Combi_Mito" dimension="4" lower="0.0"
name="stateNode" upper="1.0">0.25</parameter>
<parameter id="rateAC.s:Apisto_Combi_Mito" lower="0.001"
name="stateNode">1.0</parameter>
<parameter id="rateAG.s:Apisto_Combi_Mito" lower="0.001"
name="stateNode">1.0</parameter>
<parameter id="rateAT.s:Apisto_Combi_Mito" lower="0.001"
name="stateNode">1.0</parameter>
<parameter id="rateCG.s:Apisto_Combi_Mito" lower="0.001"
name="stateNode">1.0</parameter>
<parameter id="rateGT.s:Apisto_Combi_Mito" lower="0.001"
name="stateNode">1.0</parameter>
<parameter id="mutationRate.s:Apisto_Combi_Mito"
name="stateNode">1.0</parameter>
<parameter id="kappa.s:Apisto_Combi_Nuc" lower="0.0"
name="stateNode">2.0</parameter>
```

```
        <parameter id="proportionInvariant.s:Apisto_Combi_Mito" lower="0.0"
name="stateNode" upper="1.0">0.0</parameter>
        <parameter id="proportionInvariant.s:Apisto_Combi_Nuc" lower="0.0"
name="stateNode" upper="1.0">0.0</parameter>
        <parameter id="mutationRate.s:Apisto_Combi_Nuc"
name="stateNode">1.0</parameter>
        <parameter id="uclnClockRate.c:Apisto_Combi_Mito" lower="0.0"
name="stateNode">1.0</parameter>
        <stateNode id="uclnBranchRates.c:Apisto_Combi_Mito"
spec="parameter.IntegerParameter" dimension="354">1</stateNode>
        <parameter id="uclnClockRate.c:Apisto_Combi_Nuc" lower="0.0"
name="stateNode">1.0</parameter>
        <stateNode id="uclnBranchRates.c:Apisto_Combi_Nuc"
spec="parameter.IntegerParameter" dimension="338">1</stateNode>
        <parameter id="uclnBranchRatesStdev.c:Apisto_Combi_Nuc" lower="0.01"
name="stateNode">0.3</parameter>
    </state>

    <init id="SBI" spec="starbeast2.StarBeastInitializer"
birthRate="@speciationRate.t:Species" estimate="false" speciesTree="@Tree.t:Species">
        <geneTree idref="Tree.t:Apisto_Combi_Nuc"/>
        <geneTree idref="Tree.t:Apisto_Combi_Mito"/>
        <populationModel id="popModelBridge.Species" spec="starbeast2.PassthroughModel">
            <childModel id="lwcrPopModel.Species" spec="starbeast2.LinearWithConstantRoot"
speciesTree="@Tree.t:Species" tipPopulationSizes="@tipPopSizes.Species"
topPopulationSizes="@topPopSizes.Species"/>
        </populationModel>
    </init>

    <distribution id="posterior" spec="util.CompoundDistribution">
        <distribution id="speciescoalescent" spec="starbeast2.MultispeciesCoalescent">
            <distribution id="geneTree.t:Apisto_Combi_Mito" spec="starbeast2.GeneTree"
ploidy="0.5" populationModel="@popModelBridge.Species" speciesTree="@Tree.t:Species"
tree="@Tree.t:Apisto_Combi_Mito"/>
            <distribution id="geneTree.t:Apisto_Combi_Nuc" spec="starbeast2.GeneTree"
populationModel="@popModelBridge.Species" speciesTree="@Tree.t:Species"
tree="@Tree.t:Apisto_Combi_Nuc"/>
        </distribution>
        <distribution id="prior" spec="util.CompoundDistribution">
            <distribution id="YuleModel.t:Species" spec="beast.evolution.speciation.YuleModel"
birthDiffRate="@speciationRate.t:Species" tree="@Tree.t:Species"/>
            <prior id="KappaPrior.s:Apisto_Combi_Nuc" name="distribution"
x="@kappa.s:Apisto_Combi_Nuc">
                <LogNormal id="LogNormalDistributionModel.2" name="distr">
                    <parameter id="RealParameter.12" estimate="false"
name="M">1.0</parameter>
                    <parameter id="RealParameter.13" estimate="false"
name="S">1.25</parameter>
                </LogNormal>
            </prior>
```

```
        <prior id="lwcrPopScalePrior.Species" name="distribution"
x="@lwcrPopScale.Species">
            <OneOnX id="OneOnX" name="distr"/>
        </prior>
        <prior id="PropInvariantPrior.s:Apisto_Combi_Nuc" name="distribution"
x="@proportionInvariant.s:Apisto_Combi_Nuc">
            <Uniform id="Uniform.3" name="distr"/>
        </prior>
        <prior id="RateACPrior.s:Apisto_Combi_Mito" name="distribution"
x="@rateAC.s:Apisto_Combi_Mito">
            <Gamma id="Gamma.0" name="distr">
                <parameter id="RealParameter.0" estimate="false"
name="alpha">0.05</parameter>
                <parameter id="RealParameter.1" estimate="false"
name="beta">10.0</parameter>
            </Gamma>
        </prior>
        <prior id="RateAGPrior.s:Apisto_Combi_Mito" name="distribution"
x="@rateAG.s:Apisto_Combi_Mito">
            <Gamma id="Gamma.1" name="distr">
                <parameter id="RealParameter.2" estimate="false"
name="alpha">0.05</parameter>
                <parameter id="RealParameter.3" estimate="false"
name="beta">20.0</parameter>
            </Gamma>
        </prior>
        <prior id="RateATPrior.s:Apisto_Combi_Mito" name="distribution"
x="@rateAT.s:Apisto_Combi_Mito">
            <Gamma id="Gamma.2" name="distr">
                <parameter id="RealParameter.4" estimate="false"
name="alpha">0.05</parameter>
                <parameter id="RealParameter.5" estimate="false"
name="beta">10.0</parameter>
            </Gamma>
        </prior>
        <prior id="RateCGPrior.s:Apisto_Combi_Mito" name="distribution"
x="@rateCG.s:Apisto_Combi_Mito">
            <Gamma id="Gamma.3" name="distr">
                <parameter id="RealParameter.6" estimate="false"
name="alpha">0.05</parameter>
                <parameter id="RealParameter.7" estimate="false"
name="beta">10.0</parameter>
            </Gamma>
        </prior>
        <prior id="RateGTPrior.s:Apisto_Combi_Mito" name="distribution"
x="@rateGT.s:Apisto_Combi_Mito">
            <Gamma id="Gamma.5" name="distr">
                <parameter id="RealParameter.10" estimate="false"
name="alpha">0.05</parameter>
```

```
            <parameter id="RealParameter.11" estimate="false"
name="beta">10.0</parameter>
        </Gamma>
    </prior>
    <prior id="speciationRatePrior.t:Species" name="distribution"
x="@speciationRate.t:Species">
        <Uniform id="Uniform.0" name="distr" upper="10000.0"/>
    </prior>
    <prior id="tipPopSizesPrior.Species" name="distribution"
x="@tipPopSizes.Species">
        <Gamma id="Gamma" beta="@lwcrPopScale.Species" name="distr">
            <parameter id="tipPopShape.Species" estimate="false" lower="0.0"
name="alpha">4.0</parameter>
        </Gamma>
    </prior>
    <prior id="topPopSizesPrior.Species" name="distribution"
x="@topPopSizes.Species">
        <Gamma id="Gamma1" beta="@lwcrPopScale.Species" name="distr">
            <parameter id="topPopShape.Species" estimate="false" lower="0.0"
name="alpha">2.0</parameter>
        </Gamma>
    </prior>
    <prior id="uclnBranchRatesStdevPrior.c:Apisto_Combi_Nuc" name="distribution"
x="@uclnBranchRatesStdev.c:Apisto_Combi_Nuc">
        <Exponential id="Exponential.3" name="distr">
            <parameter id="uclnBranchRatesStdevPriorMean.s:Apisto_Combi_Nuc"
estimate="false" lower="0.0" name="mean">1.0</parameter>
        </Exponential>
    </prior>
    <prior id="uclnClockRatePrior.c:Apisto_Combi_Mito" name="distribution"
x="@uclnClockRate.c:Apisto_Combi_Mito">
        <LogNormal id="LogNormalDistributionModel.3" meanInRealSpace="true"
name="distr">
            <parameter id="uclnClockRatePriorMean.s:Apisto_Combi_Mito"
estimate="false" lower="0.0" name="M">1.0</parameter>
            <parameter id="uclnClockRatePriorStdev.s:Apisto_Combi_Mito"
estimate="false" lower="0.0" name="S">0.6</parameter>
        </LogNormal>
    </prior>
    <prior id="uclnClockRatePrior.c:Apisto_Combi_Nuc" name="distribution"
x="@uclnClockRate.c:Apisto_Combi_Nuc">
        <LogNormal id="LogNormalDistributionModel.4" meanInRealSpace="true"
name="distr">
            <parameter id="uclnClockRatePriorMean.s:Apisto_Combi_Nuc"
estimate="false" lower="0.0" name="M">1.0</parameter>
            <parameter id="uclnClockRatePriorStdev.s:Apisto_Combi_Nuc"
estimate="false" lower="0.0" name="S">0.6</parameter>
        </LogNormal>
    </prior>
```

```
        <distribution id="Geophagini.prior" spec="beast.math.distributions.MRCAPrior"
monophyletic="true" tree="@Tree.t:Species">
            <taxonset id="Geophagini" spec="TaxonSet">
                <taxon idref="Aaga"/>
                <taxon idref="Aalg"/>
                <taxon idref="Aata"/>
                <taxon idref="Abae"/>
                <taxon idref="Abar"/>
                <taxon idref="Abit"/>
                <taxon idref="Abit1"/>
                <taxon idref="Abit3"/>
                <taxon idref="Abit4"/>
                <taxon idref="Abit5"/>
                <taxon idref="Acac"/>
                <taxon idref="Acar"/>
                <taxon idref="Acin"/>
                <taxon idref="Aere"/>
                <taxon idref="Aeun"/>
                <taxon idref="Ahua"/>
                <taxon idref="Ajur"/>
                <taxon idref="Ameg"/>
                <taxon idref="Amel"/>
                <taxon idref="Amoa"/>
                <taxon idref="Amor"/>
                <taxon idref="Anij"/>
                <taxon idref="Aore"/>
                <taxon idref="Apap"/>
                <taxon idref="Apaul"/>
                <taxon idref="Apeb"/>
                <taxon idref="Aper"/>
                <taxon idref="Aros"/>
                <taxon idref="Asp2"/>
                <taxon idref="Awol"/>
                <taxon idref="Bio"/>
                <taxon idref="Cre"/>
                <taxon idref="Geo"/>
                <taxon idref="Ggym"/>
                <taxon idref="Gmer"/>
                <taxon idref="Sat"/>
            </taxonset>
            <Normal id="Normal.0" name="distr">
                <parameter id="RealParameter.14" estimate="false"
name="mean">52.0</parameter>
                <parameter id="RealParameter.15" estimate="false"
name="sigma">7.3</parameter>
            </Normal>
        </distribution>
        <distribution id="gymno.prior" spec="beast.math.distributions.MRCAPrior"
monophyletic="true" tree="@Tree.t:Species">
            <taxonset id="gymno" spec="TaxonSet">
```

```
<taxon idref="Ggym"/>
<taxon idref="Gmer"/>
</taxonset>
<Normal id="Normal.1" name="distr">
<parameter id="RealParameter.16" estimate="false"
name="mean">44.5</parameter>
<parameter id="RealParameter.17" estimate="false"
name="sigma">2.0</parameter>
</Normal>
</distribution>
</distribution>
<distribution id="likelihood" spec="util.CompoundDistribution">
<distribution id="treeLikelihood.Apisto_Combi_Mito" spec="TreeLikelihood"
data="@Apisto_Combi_Mito" tree="@Tree.t:Apisto_Combi_Mito" useAmbiguities="true">
<siteModel id="SiteModel.s:Apisto_Combi_Mito" spec="SiteModel"
mutationRate="@mutationRate.s:Apisto_Combi_Mito"
proportionInvariant="@proportionInvariant.s:Apisto_Combi_Mito">
<parameter id="gammaShape.s:Apisto_Combi_Mito" estimate="false"
name="shape">1.0</parameter>
<substModel id="gtr.s:Apisto_Combi_Mito" spec="GTR"
rateAC="@rateAC.s:Apisto_Combi_Mito" rateAG="@rateAG.s:Apisto_Combi_Mito"
rateAT="@rateAT.s:Apisto_Combi_Mito" rateCG="@rateCG.s:Apisto_Combi_Mito"
rateGT="@rateGT.s:Apisto_Combi_Mito">
<parameter id="rateCT.s:Apisto_Combi_Mito" estimate="false"
lower="0.001" name="rateCT">1.0</parameter>
<frequencies id="estimatedFreqs.s:Apisto_Combi_Mito" spec="Frequencies"
frequencies="@freqParameter.s:Apisto_Combi_Mito"/>
</substModel>
</siteModel>
<branchRateModel id="GeneTreeUCLN.c:Apisto_Combi_Mito"
spec="starbeast2.UncorrelatedRates" clock.rate="@uclnClockRate.c:Apisto_Combi_Mito"
rates="@uclnBranchRates.c:Apisto_Combi_Mito" tree="@Tree.t:Apisto_Combi_Mito">
<parameter id="uclnBranchRatesStdev.c:Apisto_Combi_Mito" estimate="false"
lower="0.01" name="stdev">0.3</parameter>
</branchRateModel>
</distribution>
<distribution id="treeLikelihood.Apisto_Combi_Nuc" spec="TreeLikelihood"
data="@Apisto_Combi_Nuc" tree="@Tree.t:Apisto_Combi_Nuc" useAmbiguities="true">
<siteModel id="SiteModel.s:Apisto_Combi_Nuc" spec="SiteModel"
mutationRate="@mutationRate.s:Apisto_Combi_Nuc"
proportionInvariant="@proportionInvariant.s:Apisto_Combi_Nuc">
<parameter id="gammaShape.s:Apisto_Combi_Nuc" estimate="false"
name="shape">1.0</parameter>
<substModel id="hky.s:Apisto_Combi_Nuc" spec="HKY"
kappa="@kappa.s:Apisto_Combi_Nuc">
<frequencies id="equalFreqs.s:Apisto_Combi_Nuc" spec="Frequencies"
data="@Apisto_Combi_Nuc" estimate="false"/>
</substModel>
</siteModel>
```

```
            <branchRateModel id="GeneTreeUCLN.c:Apisto_Combi_Nuc"
spec="starbeast2.UncorrelatedRates" clock.rate="@uclnClockRate.c:Apisto_Combi_Nuc"
rates="@uclnBranchRates.c:Apisto_Combi_Nuc"
stdev="@uclnBranchRatesStdev.c:Apisto_Combi_Nuc"
tree="@Tree.t:Apisto_Combi_Nuc"/>
         </distribution>
      </distribution>
      <distribution id="fossilCalibrations" spec="util.CompoundDistribution"/>
   </distribution>

   <operator id="Reheight.t:Species" spec="starbeast2.NodeReheight2"
taxonset="@taxonsuperset" tree="@Tree.t:Species" weight="75.0">
      <geneTree idref="geneTree.t:Apisto_Combi_Nuc"/>
      <geneTree idref="geneTree.t:Apisto_Combi_Mito"/>
   </operator>

   <operator id="coordinatedUniform.t:Species" spec="starbeast2.CoordinatedUniform"
speciesTree="@Tree.t:Species" weight="15.0">
      <geneTree idref="Tree.t:Apisto_Combi_Nuc"/>
      <geneTree idref="Tree.t:Apisto_Combi_Mito"/>
   </operator>

   <operator id="coordinatedExponential.t:Species"
spec="starbeast2.CoordinatedExponential" speciesTree="@Tree.t:Species"
weight="9.0437">
      <geneTree idref="Tree.t:Apisto_Combi_Nuc"/>
      <geneTree idref="Tree.t:Apisto_Combi_Mito"/>
   </operator>

   <operator id="TreeScaler.t:Species" spec="ScaleOperator" scaleFactor="0.9947"
tree="@Tree.t:Species" weight="3.0"/>

   <operator id="TreeRootScaler.t:Species" spec="ScaleOperator" rootOnly="true"
scaleFactor="0.908" tree="@Tree.t:Species" weight="3.0"/>

   <operator id="UniformOperator.t:Species" spec="Uniform" tree="@Tree.t:Species"
weight="15.0"/>

   <operator id="SubtreeSlide.t:Species" spec="SubtreeSlide" size="1.3658"
tree="@Tree.t:Species" weight="15.0"/>

   <operator id="Narrow.t:Species" spec="Exchange" tree="@Tree.t:Species"
weight="15.0"/>

   <operator id="Wide.t:Species" spec="Exchange" isNarrow="false" tree="@Tree.t:Species"
weight="15.0"/>

   <operator id="WilsonBalding.t:Species" spec="WilsonBalding" tree="@Tree.t:Species"
weight="15.0"/>
```

```
    <operator id="updownAll:Species" spec="UpDownOperator" scaleFactor="0.724"
weight="6.0">
        <up idref="speciationRate.t:Species"/>
        <up idref="uclnClockRate.c:Apisto_Combi_Mito"/>
        <up idref="uclnClockRate.c:Apisto_Combi_Nuc"/>
        <down idref="Tree.t:Species"/>
        <down idref="Tree.t:Apisto_Combi_Nuc"/>
        <down idref="Tree.t:Apisto_Combi_Mito"/>
        <down idref="tipPopSizes.Species"/>
        <down idref="topPopSizes.Species"/>
        <down idref="lwcrPopScale.Species"/>
    </operator>

    <operator id="speciationRateScale.t:Species" spec="ScaleOperator"
parameter="@speciationRate.t:Species" scaleFactor="0.5" weight="1.0"/>

    <operator id="clockUpDownOperator.c:Apisto_Combi_Nuc" spec="UpDownOperator"
scaleFactor="0.9837" weight="3.0">
        <up idref="uclnClockRate.c:Apisto_Combi_Nuc"/>
        <down idref="Tree.t:Apisto_Combi_Nuc"/>
    </operator>

    <operator id="TreeScaler.t:Apisto_Combi_Nuc" spec="ScaleOperator"
scaleFactor="0.9841" tree="@Tree.t:Apisto_Combi_Nuc" weight="3.0"/>

    <operator id="TreeRootScaler.t:Apisto_Combi_Nuc" spec="ScaleOperator"
rootOnly="true" scaleFactor="0.4390" tree="@Tree.t:Apisto_Combi_Nuc" weight="3.0"/>

    <operator id="UniformOperator.t:Apisto_Combi_Nuc" spec="Uniform"
tree="@Tree.t:Apisto_Combi_Nuc" weight="15.0"/>

    <operator id="SubtreeSlide.t:Apisto_Combi_Nuc" spec="SubtreeSlide" size="3.3602"
tree="@Tree.t:Apisto_Combi_Nuc" weight="15.0"/>

    <operator id="Narrow.t:Apisto_Combi_Nuc" spec="Exchange"
tree="@Tree.t:Apisto_Combi_Nuc" weight="15.0"/>

    <operator id="Wide.t:Apisto_Combi_Nuc" spec="Exchange" isNarrow="false"
tree="@Tree.t:Apisto_Combi_Nuc" weight="15.0"/>

    <operator id="WilsonBalding.t:Apisto_Combi_Nuc" spec="WilsonBalding"
tree="@Tree.t:Apisto_Combi_Nuc" weight="15.0"/>

    <operator id="clockUpDownOperator.c:Apisto_Combi_Mito" spec="UpDownOperator"
scaleFactor="0.996" weight="3.0">
        <up idref="uclnClockRate.c:Apisto_Combi_Mito"/>
        <down idref="Tree.t:Apisto_Combi_Mito"/>
    </operator>
```

```
    <operator id="TreeScaler.t:Apisto_Combi_Mito" spec="ScaleOperator"
scaleFactor="0.996" tree="@Tree.t:Apisto_Combi_Mito" weight="3.0"/>

    <operator id="TreeRootScaler.t:Apisto_Combi_Mito" spec="ScaleOperator"
rootOnly="true" scaleFactor="0.9114" tree="@Tree.t:Apisto_Combi_Mito" weight="3.0"/>

    <operator id="UniformOperator.t:Apisto_Combi_Mito" spec="Uniform"
tree="@Tree.t:Apisto_Combi_Mito" weight="15.0"/>

    <operator id="SubtreeSlide.t:Apisto_Combi_Mito" spec="SubtreeSlide" size="0.5432"
tree="@Tree.t:Apisto_Combi_Mito" weight="15.0"/>

    <operator id="Narrow.t:Apisto_Combi_Mito" spec="Exchange"
tree="@Tree.t:Apisto_Combi_Mito" weight="15.0"/>

    <operator id="Wide.t:Apisto_Combi_Mito" spec="Exchange" isNarrow="false"
tree="@Tree.t:Apisto_Combi_Mito" weight="15.0"/>

    <operator id="WilsonBalding.t:Apisto_Combi_Mito" spec="WilsonBalding"
tree="@Tree.t:Apisto_Combi_Mito" weight="15.0"/>

    <operator id="tipPopSizesSwap.Species" spec="SwapOperator"
parameter="@tipPopSizes.Species" weight="3.0"/>

    <operator id="topPopSizesSwap.Species" spec="SwapOperator"
parameter="@topPopSizes.Species" weight="3.0"/>

    <operator id="tipPopSizesScale.Species" spec="ScaleOperator"
parameter="@tipPopSizes.Species" scaleFactor="0.1967" weight="3.0"/>

    <operator id="topPopSizesScale.Species" spec="ScaleOperator"
parameter="@topPopSizes.Species" scaleFactor="0.1414" weight="3.0"/>

    <operator id="lwcrPopScaleScale.Species" spec="ScaleOperator"
parameter="@lwcrPopScale.Species" scaleFactor="0.6764" weight="1.0"/>

    <operator id="FrequenciesExchanger.s:Apisto_Combi_Mito"
spec="DeltaExchangeOperator" delta="0.0396" weight="1.5">
        <parameter idref="freqParameter.s:Apisto_Combi_Mito"/>
    </operator>

    <operator id="RateACScaler.s:Apisto_Combi_Mito" spec="ScaleOperator"
parameter="@rateAC.s:Apisto_Combi_Mito" scaleFactor="0.6355" weight="1.0"/>

    <operator id="RateAGScaler.s:Apisto_Combi_Mito" spec="ScaleOperator"
parameter="@rateAG.s:Apisto_Combi_Mito" scaleFactor="0.7477" weight="1.0"/>

    <operator id="RateATScaler.s:Apisto_Combi_Mito" spec="ScaleOperator"
parameter="@rateAT.s:Apisto_Combi_Mito" scaleFactor="0.5793" weight="1.0"/>
```

```
    <operator id="RateCGScaler.s:Apisto_Combi_Mito" spec="ScaleOperator"
parameter="@rateCG.s:Apisto_Combi_Mito" scaleFactor="0.3950" weight="1.0"/>

    <operator id="RateGTScaler.s:Apisto_Combi_Mito" spec="ScaleOperator"
parameter="@rateGT.s:Apisto_Combi_Mito" scaleFactor="0.4161" weight="1.0"/>

    <operator id="FixMeanMutationRatesOperator" spec="DeltaExchangeOperator"
delta="0.027" weight="2.0">
        <parameter idref="mutationRate.s:Apisto_Combi_Mito"/>
        <parameter idref="mutationRate.s:Apisto_Combi_Nuc"/>
        <weightvector id="weightparameter" spec="parameter.IntegerParameter" dimension="2"
estimate="false" lower="0" upper="0">1252 293</weightvector>
    </operator>

    <operator id="KappaScaler.s:Apisto_Combi_Nuc" spec="ScaleOperator"
parameter="@kappa.s:Apisto_Combi_Nuc" scaleFactor="0.2501" weight="1.0"/>

    <operator id="proportionInvariantScaler.s:Apisto_Combi_Mito" spec="ScaleOperator"
parameter="@proportionInvariant.s:Apisto_Combi_Mito" scaleFactor="0.685"
weight="1.0"/>

    <operator id="proportionInvariantScaler.s:Apisto_Combi_Nuc" spec="ScaleOperator"
parameter="@proportionInvariant.s:Apisto_Combi_Nuc" scaleFactor="0.805"
weight="1.0"/>

    <operator id="uclnClockRateScaler.c:Apisto_Combi_Mito" spec="ScaleOperator"
parameter="@uclnClockRate.c:Apisto_Combi_Mito" scaleFactor="0.8724" weight="3.0"/>

    <operator id="uclnBranchRatesCycle.c:Apisto_Combi_Mito"
spec="starbeast2.DiscreteRateCycle" k="8" optimise="false"
treeRates="@uclnBranchRates.c:Apisto_Combi_Mito" weight="9.0"/>

    <operator id="uclnBranchRatesUniform.c:Apisto_Combi_Mito"
spec="starbeast2.DiscreteRateUniform" k="8" optimise="false"
treeRates="@uclnBranchRates.c:Apisto_Combi_Mito" weight="9.0"/>

    <operator id="uclnClockRateScaler.c:Apisto_Combi_Nuc" spec="ScaleOperator"
parameter="@uclnClockRate.c:Apisto_Combi_Nuc" scaleFactor="0.4807" weight="3.0"/>

    <operator id="uclnBranchRatesCycle.c:Apisto_Combi_Nuc"
spec="starbeast2.DiscreteRateCycle" k="16" optimise="false"
treeRates="@uclnBranchRates.c:Apisto_Combi_Nuc" weight="9.0"/>

    <operator id="uclnBranchRatesUniform.c:Apisto_Combi_Nuc"
spec="starbeast2.DiscreteRateUniform" k="8" optimise="false"
treeRates="@uclnBranchRates.c:Apisto_Combi_Nuc" weight="9.0"/>

    <operator id="uclnBranchRatesStdevScaler.c:Apisto_Combi_Nuc" spec="ScaleOperator"
parameter="@uclnBranchRatesStdev.c:Apisto_Combi_Nuc" scaleFactor="0.75"
weight="3.0"/>
```

```
    <logger id="tracelog" fileName="starbeast_LY.log" logEvery="5000"
model="@posterior" sort="smart">
        <log idref="posterior"/>
        <log idref="likelihood"/>
        <log idref="prior"/>
        <log idref="speciescoalescent"/>
        <log idref="speciationRate.t:Species"/>
        <log idref="YuleModel.t:Species"/>
        <log id="TreeHeight.Species" spec="beast.evolution.tree.TreeHeightLogger"
tree="@Tree.t:Species"/>
        <log id="TreeLength.Species" spec="starbeast2.TreeLengthLogger"
tree="@Tree.t:Species"/>
        <log idref="treeLikelihood.Apisto_Combi_Nuc"/>
        <log id="TreeHeight.t:Apisto_Combi_Nuc"
spec="beast.evolution.tree.TreeHeightLogger" tree="@Tree.t:Apisto_Combi_Nuc"/>
        <log idref="treeLikelihood.Apisto_Combi_Mito"/>
        <log id="TreeHeight.t:Apisto_Combi_Mito"
spec="beast.evolution.tree.TreeHeightLogger" tree="@Tree.t:Apisto_Combi_Mito"/>
        <log idref="lwcrPopScale.Species"/>
        <log idref="freqParameter.s:Apisto_Combi_Mito"/>
        <log idref="rateAC.s:Apisto_Combi_Mito"/>
        <log idref="rateAG.s:Apisto_Combi_Mito"/>
        <log idref="rateAT.s:Apisto_Combi_Mito"/>
        <log idref="rateCG.s:Apisto_Combi_Mito"/>
        <log idref="rateGT.s:Apisto_Combi_Mito"/>
        <log idref="mutationRate.s:Apisto_Combi_Mito"/>
        <log idref="kappa.s:Apisto_Combi_Nuc"/>
        <log idref="proportionInvariant.s:Apisto_Combi_Mito"/>
        <log idref="proportionInvariant.s:Apisto_Combi_Nuc"/>
        <log idref="mutationRate.s:Apisto_Combi_Nuc"/>
        <log idref="uclnClockRate.c:Apisto_Combi_Mito"/>
        <log idref="Geophagini.prior"/>
        <log idref="gymno.prior"/>
        <log idref="uclnClockRate.c:Apisto_Combi_Nuc"/>
        <log idref="uclnBranchRatesStdev.c:Apisto_Combi_Nuc"/>
    </logger>

    <logger id="speciesTreeLogger" fileName="species_LY.trees" logEvery="5000"
mode="tree">
        <log id="SpeciesTreeLoggerX" spec="starbeast2.SpeciesTreeLogger"
populationmodel="@lwcrPopModel.Species" speciesTree="@Tree.t:Species"/>
    </logger>

    <logger id="screenlog" fileName="apisto_combi_LY.txt" logEvery="5000"
model="@posterior">
        <log idref="posterior"/>
        <log id="ESS.0" spec="util.ESS" arg="@posterior"/>
        <log idref="likelihood"/>
        <log idref="prior"/>
```

```
    </logger>

    <logger id="treelog.t:Apisto_Combi_Nuc" fileName="$(tree)_LY.trees" logEvery="5000"
mode="tree">
        <log id="TreeWithMetaDataLogger.t:Apisto_Combi_Nuc"
spec="beast.evolution.tree.TreeWithMetaDataLogger"
branchratemodel="@GeneTreeUCLN.c:Apisto_Combi_Nuc"
tree="@Tree.t:Apisto_Combi_Nuc"/>
    </logger>

    <logger id="treelog.t:Apisto_Combi_Mito" fileName="$(tree)_LY.trees" logEvery="5000"
mode="tree">
        <log id="TreeWithMetaDataLogger.t:Apisto_Combi_Mito"
spec="beast.evolution.tree.TreeWithMetaDataLogger"
branchratemodel="@GeneTreeUCLN.c:Apisto_Combi_Mito"
tree="@Tree.t:Apisto_Combi_Mito"/>
    </logger>

</run>

</beast>
```
